# Epidermal Growth Factor Is Essential for the Maintenance of Novel Prostate Epithelial Cells Isolated From Patient-Derived Organoids

**DOI:** 10.3389/fcell.2020.571677

**Published:** 2020-10-29

**Authors:** Katia Cheaito, Hisham F. Bahmad, Hiba Jalloul, Ola Hadadeh, Hiba Msheik, Albert El-Hajj, Deborah Mukherji, Mohamed Al-Sayegh, Wassim Abou-Kheir

**Affiliations:** ^1^Department of Anatomy, Cell Biology and Physiological Sciences, Faculty of Medicine, American University of Beirut, Beirut, Lebanon; ^2^Division of Urology, Department of Surgery, American University of Beirut Medical Center, Beirut, Lebanon; ^3^Division of Hematology-Oncology, Department of Internal Medicine, American University of Beirut Medical Center, Beirut, Lebanon; ^4^Biology Division, New York University Abu Dhabi, Abu Dhabi, United Arab Emirates

**Keywords:** prostate epithelial cells, prostate cancer, organoids, lineage markers, RNA-seq, EGF

## Abstract

Prostate cancer (PCa) is the second leading cause of cancer-related mortality and morbidity among males worldwide. Deciphering the biological mechanisms and molecular pathways involved in PCa pathogenesis and progression has been hindered by numerous technical limitations mainly attributed to the limited number of cell lines available, which do not recapitulate the diverse phenotypes of clinical disease. Indeed, PCa has proven problematic to establish as cell lines in culture due to its heterogeneity which remains a challenge, despite the various *in vitro* and *in vivo* model systems available. Growth factors have been shown to play a central role in the complex regulation of cell proliferation among hormone sensitive tumors, such as PCa. Here, we report the isolation and characterization of novel patient-derived prostate epithelial (which we named as AUB-PrC) cells from organoids culture system. We also assessed the role of epidermal growth factor (EGF) in culturing those cells. We profiled the AUB-PrC cells isolated from unaffected and tumor patient samples via depicting their molecular and epithelial lineage features through immunofluorescence staining and quantitative real-time PCR (qRT-PCR), as well as through functional assays and transcriptomic profiling through RNA sequencing. In addition, by optimizing a previously established prostate organoids culture system, we were able to grow human prostate epithelial cells using growth medium and EGF only. With these data collected, we were able to gain insight at the molecular architecture of novel human AUB-PrC cells, which might pave the way for deciphering the mechanisms that lead to PCa development and progression, and ultimately improving prognostic abilities and treatments.

## Introduction

Prostate cancer (PCa) is the most commonly diagnosed cancer and second leading cause of cancer-related deaths among males worldwide, with an estimated annual incidence of 191,930 in the United States in 2020, and estimated deaths of 33,330 per year (Siegel et al., 2020). PCa usually contains multifocal lesions (with varying genetic alterations) and is heterogenous at the molecular, cellular and architectural levels (Zhang et al., 2016), which makes obtaining a homogenous material for molecular analysis difficult (Abate-Shen and Shen, 2000; Bahmad et al., 2020b; Daouk et al., 2020). The heterogeneity of this tumor also renders choosing the best therapy for each patient (castration therapy, surgery, radiotherapy, or chemotherapy) very challenging (Karantanos et al., 2013; Zhang et al., 2016).

Understanding the molecular pathways involved in PCa has been hindered by numerous technical limitations. These mainly relate to the limited number of PCa cell lines available, which do not recapitulate the diverse phenotypes of clinical disease (Ziaee et al., 2015). Nonetheless, the need for representative *in vitro* and *in vivo* models that recapitulate different stages of PCa (Daoud et al., 2016; Daouk et al., 2020; Bahmad et al., 2020b), especially castration-resistant prostate cancer (CRPC), has led to numerous attempts to establish cell lines from human prostate carcinomas (Van Bokhoven et al., 2003). Prostate carcinomas, however, have been the most challenging to establish continuous cell lines from Cunningham and You (2015) and Huang et al. (2016). Approximately 30 reported human prostate cell lines have been described and used for research purposes from 1970 to the present (Van Bokhoven et al., 2003). Due to contamination of putative prostate cell lines, those cells turned out to be derivatives of previously established prostate carcinoma cell lines such as DU145 and PC-3 (Chen, 1993; MacLeod et al., 1999; Pan et al., 2001; Van Bokhoven et al., 2001, 2003). It is thus important to select prostate cell lines that accurately depict its molecular features in order to address research questions appropriately, preferably generated from primary human tissue, bearing in mind that generating a “new primary” PCa cell line is very challenging (Sobel and Sadar, 2005).

A novel promising technology has been recently developed to study tissue homeostasis through a three-dimensional (3D) organoid culture system (Koo et al., 2011). These organoids that mimic the structures of tissues *in vivo*, can grow “indefinitely” in culture and remain phenotypically and genetically stable (Sato et al., 2009; Schwank et al., 2013a,b; Drost et al., 2016). It is believed that they stem from single multipotent stem cells or progenitors capable of differentiation and self-organization to form structures morphologically and functionally resembling the corresponding *in vivo* organ (Bartucci et al., 2016; Bahmad et al., 2020a). Currently, organoids are being established from a variety of organs, including the colon, stomach, and prostate among others (Barker et al., 2010; Eiraku et al., 2011; Jung et al., 2011; Sato et al., 2011; Antonica et al., 2012; Huch et al., 2013; Koehler et al., 2013; Lancaster et al., 2013; Stange et al., 2013; Sachs and Clevers, 2014; Taguchi et al., 2014; Takasato et al., 2014; Agarwal et al., 2015; Drost et al., 2016). Karthaus et al. adapted this culture method to PCa and described an R-spondin1-based 3D culture method through which normal human and murine prostate epithelial cells can be cultured indefinitely without genetic manipulation, in an *in vitro* 3D system that models prostate glandular structure (Karthaus et al., 2014).

Herein, we employed the 3D organoid culture system to generate patient-derived prostate epithelial (American University of Beirut-Prostate Cells; AUB-PrC) cells *in vitro* in an attempt to establish new cells without any genetic manipulation. Since EGFR ligands (such as EGF) and other growth factors have been shown to mediate epithelial cell repair of bronchial cells (Barrow et al., 1993; Burgel and Nadel, 2004), breast cancer (Fitzpatrick et al., 1984; Kim et al., 2012), and PCa cells (Peehl et al., 1996; Festuccia et al., 2005), we hypothesized that EGF might have a role in prostate epithelial cell growth in culture as well. This is supported by the notion that human recombinant EGF is known to be essential for the growth of PCa cells cultured in keratinocyte growth media (Bahmad et al., 2018). We characterized the novel generated primary AUB-PrC cells for molecular and epithelial lineage features through immunofluorescence (IF) staining and quantitative real-time PCR (qRT-PCR), as well as through functional assays and transcriptomic profiling through RNA sequencing.

## Materials and Methods

### Patients Selection

Samples from different stages of human prostate adenocarcinomas were obtained from consented treatment-naïve patients undergoing radical prostatectomy at the American University of Beirut Medical Center (AUBMC). Appropriate Institutional Review Board (IRB) approval was obtained. After getting written informed consents from the patients, primary tissue samples collected were used only if this doesn’t compromise the diagnosis or staging. A sample was taken from each patient from the area most likely to be involved with cancer (from the core of the cancerous lesion) and a sample from the unaffected area (far from the tumor site) according to the urologist’s and pathologist’s recommendation.

A total of seven treatment-naïve patients with PCa diagnosis were enrolled in our study and tested for PSA level at the time of operation. Prostate tissue specimens were collected, weighed, and characterized then assigned a Gleason score, International Society of Urological Pathology (ISUP) grade group, and TNM cancer staging by a pathologist at AUBMC. Among the seven patients included, there was no cancer invasion to nearby lymph nodes and the cancer had not metastasized to other parts of the body (Supplementary Table S1).

### Collection, Dissection, and Processing of Patient Prostate Tissue Specimens

The collected fresh prostate tissues (ranging from 3 to 5 mm in size) were directly put in a 50 mL conical tube containing “human prostate growth medium” right after the surgery, sent to the research laboratory, and kept at 4°C until processing (within 6 h to maximize the reliability of organoids generation). Using sterile scalpel blades, prostate tissue fragments were minced into approximately 0.1–0.5 mm diameter pieces and washed with “human prostate growth medium” to get rid of cellular debris. Part of minced fragments were used for organoids culturing and the remaining fragments were used for RNA extraction and sequencing.

Prostate tissue fragments designated “unaffected” (U) and “tumor” (T) and minced using sterile scalpel blades were kept overnight in a humidified incubator containing 5% CO_2_ at 37°C with 5 mL of 5 mg/mL collagenase type II (Gibco^TM^; cat #17101-015) in Advanced DMEM-F12 medium (adDMEM/F12) (Gibco^TM^; cat #12634-010) with ROCK inhibitor (Y-27632) (Santa Cruz; cat #sc-281642A) to digest the tissue. The next day, cells were washed with adDMEM/F12, then centrifuged at 200 g for 5 min at 4°C. The pellet was resuspended in 1 mL TrypLE^TM^ (ThermoFisher; cat #12605-010) with Y-27632 and digested for approximately 15 min at 37°C. The pellet was then washed once with adDMEM/F12 and centrifuged at 200 g for 5 min at 4°C. Digested tissue was placed in ice-cold Matrigel^TM^ (Corning Life Sciences; cat #354230) and pipetted up and down several times to mix. Around 20,000 cells in a 40 μL drop of 90% Matrigel^TM^ were plated into the middle of one well of a 24-wells culture plate which was placed upside down in the 37°C incubator for 15 min to allow the Matrigel^TM^ to solidify. Then, 500 μL of pre-warmed (37°C) human prostate growth medium plus Y-27632 was added gently into each well. Media was replenished every 3 days using human prostate growth medium plus Y-27632. ROCK inhibitor (Y-27632) was added fresh to the culture medium on the same day medium is changed for the first week after plating only.

### Human Prostate Growth Medium Components

“Human prostate growth medium” was prepared using adDMEM/F12 supplemented with 1% Penicillin/Streptomycin (v/v) (Biowest; cat #L0022-100), 0.2% Gentamicin/Amphotericin B (v/v) (Thermo Fisher; cat #R01510), 0.2% plasmocin prophylactic (v/v) (Invivogen; cat #ant-mpp), 10 mM HEPES (Gibco^TM^; cat #15630-056) and 2 mM GlutaMAX (Gibco^TM^; cat #35050-061). For organoids culturing, organoid medium components specified in Supplementary Table S2 were added (Cheaito et al., under review).

### Culturing of Patient-Derived Prostate Epithelial (AUB-PrC) Cells

After passaging the organoids, leftover two-dimensional (2D) cells were detached using TrypLE^TM^ and then transferred to T25 plates previously coated with 1% collagen-I. Cells were supplemented with “human prostate growth medium” plus ROCK inhibitor (Y-27632) and incubated at 37°C in a CO_2_ incubator. These patient-derived 2D cells were split at a ratio of 1:2 every 3–4 days where 50% of cells were frozen down in FBS + 10% DMSO (v/v) and stored in liquid nitrogen, and 50% were maintained in culture using same conditions and medium.

American University of Beirut-Prostate Cells (AUB-PrC) cells from patients (unaffected and tumor parts) were named as follows:

• **AUB-PrC-U#:** Patient # unaffected prostate epithelial cells
• **AUB-PrC-T#:** Patient # tumor prostate epithelial cells

(# designates the patient number from 1 to 7)

Cells were frozen in fetal bovine serum (FBS) (Sigma-Aldrich; cat #F9665) + 10% dimethyl sulfoxide (DMSO) (Scharlau; cat #SU01571000) as P0 cells to serve as a stock of patient’s derived cells for later use. All cells were kept as a stock in liquid nitrogen.

### Immunofluorescence (IF) Analysis of Cells

Indirect immunofluorescence analysis was used to characterize prostate epithelial lineage markers, including CK8 and CK5. Cells were grown on collagen-I coated coverslips. Adherent cells were then fixed using 4% PFA (v/v) in PBS for 20 min, then permeabilized with 0.5% Triton X-100 (v/v) in PBS for 20 min. Non-specific sites were blocked by incubation in blocking buffer [0.1% BSA (v/v), 0.2% Triton X-100 (v/v), 0.05% Tween-20 (v/v) and 10% NGS (v/v] in PBS) for 1 h [bovine serum albumin (BSA) (v/v) (Amresco; cat #0332-100G), normal goat serum (NGS) (v/v) (ThermoFisher; cat #16210064), Tween-20 (Sigma-Aldrich; cat #P1379), and Triton X-100 (v/v) (Sigma-Aldrich; cat #T9284)]. Cells were then incubated overnight with specific primary antibodies at 4°C. After washing with PBS containing 0.1% Tween-20 (v/v), cells were incubated with the corresponding secondary antibodies, then washed gently and mounted with anti-fade reagent Fluoro-gel II with DAPI (Electron Microscopy Sciences; cat #17985-51). Immunofluorescence images were captured using the Carl Zeiss Axio Observer.Z1 with 40 × oil reflector and confocal microscopic analyses was performed using Zeiss LSM710 laser scanning confocal microscope, both utilizing the Carl Zeiss ZEN 2013 image software.

The following antibodies were used: mouse monoclonal anti-CK8 (1/200 dilution; BioLegend; cat #MMS-162P), rabbit polyclonal anti-CK5 (1/200 dilution; BioLegend; cat #PRB-160P), rabbit polyclonal anti-CK14 (1/200 dilution; BioLegend; cat #PRB-155P), rabbit polyclonal anti-Vim (1/50 dilution; Santa Cruz Biotechnology; cat #sc-5565) Alexa 568 goat anti-mouse IgG (ThermoFisher; cat #A-11004), and Alexa 488 goat anti-rabbit IgG (ThermoFisher; cat # A-11034). All secondary Alexa Fluor antibodies were used at 1/200 dilution. Fluoro-gel II with DAPI (Electron Microscopy Sciences, PA) was used for mounting.

### Total RNA Extraction and Purification

Total RNA was extracted from corresponding samples using both TriZol (ThermoFisher; cat #15596026) and RNAeasy Mini Kit (Qiagen; cat #74104) according to manufacturer’s protocols with modifications. Patient tissues, organoids, and organoid-derived AUB-PrC cells were washed once with 1 mL of PBS prior to the addition of 1 mL of TriZol reagent, which were used to isolated total RNA (upper aqueous phase) after the addition of 0.2 mL of Chloroform followed by centrifugation at 12,000 rpm for 15 min at 4°C. Isolated RNA phase were mixed with 70% ethanol with equal volumes followed by purification of RNA using RNAeasy Mini spin column (Qiagen) according to the manufacturer’s protocol. Concentrations and integrity (RNA integrity number—RIN) of isolated RNA were determined using ThermoScientific^TM^ NanoDrop 2000^TM^ and Agilent BioAnalyzer 2100^TM^, respectively.

### Quantitative Real-Time Polymerase Chain Reaction (qRT-PCR) of Cells

For cDNA preparation, the Quantitect Reverse Transcription Kit (Qiagen; cat #205311) was utilized. cDNA was diluted in a 1:10 volume ratio of DEPC. mRNA expression of normal and tumor samples were analyzed by RT-PCR (Bio-rad CFX^TM^ Manager Software; cat #1845000) using the ΔC_t_ method and the SYBR green system. All reactions were performed using 2X SYBR Green master mixes each containing 2 μL template cDNA, 0.5 μL of each primer mix (forward and reverse), 5 μL buffer containing SYBR Green (Applied Biosystems; cat #A46111) and 2.5 μL of RNase free water (Primers used are listed in Supplementary Table S3).

The PCR reaction consisted of a DNA denaturation step at 95°C for 5 min, followed by 40 cycles (denaturation at 95°C for 10 s), then annealing at the appropriate temperature of 60°C for each primer for 30 s, and finally an extension step at 72°C for 10 min. For each experiment, reactions were performed in duplicates and expression of individual genes was normalized to the house keeping gene *GAPDH*. Gene expression was calculated through the following equation: ΔC_t_ = C_t (target)_ − C_t (GAPDH__)_. The amount of endogenous target gene relative to a calibrator (*GAPDH*) became 2^–Δ^ ^Ct^.

### RNA Sequencing of AUB-PrC Cells vs. Their Corresponding Tissue Counterparts

#### RNA-Seq Library Preparation and Sequencing

RNA samples from two patients (patients 4 and 5) with total concentrations of > 0.5 ng/μl and RIN > 8 were used for library preparation. RNA Sequencing (RNA-Seq) libraries were prepared using Illumina^#x00AE;^ TruSeq Stranded mRNA prep kit (Illumina; cat #RS-122-2101) accordingly with the manufacturers LS protocol. Samples were barcoded, multiplexed and sequenced (100 bp pair-end) using the Illumina^®^ Hi-Seq 2500 platform at New York University Abu Dhabi (NYUAD) Genomic Core facility (Abu Dhabi, U.A.E.).

#### Transcriptome Data Computational Analysis (Subject to Change)

DESeq2 computational pipeline was used to estimate the raw count reads aligned to the reference genome (Love et al., 2014). Computing methods were run on a Linux based command system on NYUAD High Performance Computing (HPC) server platform Dalma. Correlation (i.e., Principle Component Analysis—PCA) analysis were generated by RNA-Seq START (Shiny Transcriptome Analysis Resource Tool) application, via the New York University Abu Dhabi Center of Genomic and Systems Biology (NYUAD-CGSB) Bioinformatics Online Analysis and Visualization Portal^1^ (Nelson et al., 2017). The data discussed in this paper have been deposited in NCBI’s Gene Expression Omnibus (Edgar et al., 2002) and are accessible through GEO Series accession number GSE148937^2^.

#### Gene Array Data Analysis

Differentially expressed gene (DEG) features (3,383 and 4,250 significantly differentially expressed transcripts between the AUB-PrC cells and their corresponding tissues in the unaffected and tumor samples, respectively) were subjected to Gene Ontology (GO) term and gene set enrichment analyses using GSEA, Cytoscape, and EnrichmentMap bioinformatics tools (Reimand et al., 2019). The database of pathway gene sets used for pathway enrichment analysis was downloaded from http://baderlab.org/GeneSets and it includes eight data sources: MSigDB (C2 collection) (Subramanian et al., 2005), NCI (Schaefer et al., 2009), Institute of Bioinformatics (IOB), NetPath (Kandasamy et al., 2010), HumanCyc (Romero et al., 2005), Reactome (Croft et al., 2011), GO (Ashburner et al., 2000), MSigDB (C3 collection; Specialty GMTs mirs, transcription factors) (Subramanian et al., 2005), and Panther (Mi et al., 2005; Supplementary Table S4).

### MTT Cell Growth Assay

MTT ([3-(4, 5-dimethylthiazol-2-yl)-2, 5-diphenyltetrazolium bromide]) (Sigma-Aldrich; cat #M5655-1G) cell growth assay was used, according to the manufacturer’s instructions (Mosmann, 1983; Riss et al., 2004; Van Meerloo et al., 2011), to measure the *in vitro* cell proliferation and growth of the unaffected and tumor patient-derived AUB-PrC cells under the three different culturing conditions:

• Condition control “All Factors” was prepared as described in Supplementary Table S2
• Condition “All Factors – EGF” included all other components except EGF
• Condition “EGF alone” included adDMEM/F12 medium + EGF only (10 ng/mL)

AUB-PrC cells were derived from tissue samples of 3 different patients (Patients 5, 6, and 7), including the unaffected and the tumor sample for each. Cells were seeded at a density of 4 × 10^3^ cells/well in 100 μL in triplicates in 96-well culture plates and incubated overnight at 37°C in a humidified incubator containing 5% CO_2_, before being exposed to the different culturing conditions for 72 h. Media was changed at 24 and 48 h. The reduced MTT optical density (OD) was measured by the microplate ELISA reader (Multiscan EX) at a wavelength of 595 nm. The percentage of cell viability was presented as percentage growth using the OD ratio of cells relative to condition “All Factors.” The average percentage cell viability in each condition was derived from the mean of triplicate wells of three independent experiments.

### Cell Viability (Trypan Blue Exclusion Method)

Unaffected and tumor AUB-PrC cells from three patients were seeded, in triplicates, in 12-well plates at a density of 5 × 10^4^ cells per well. Cells were then cultured under the three different culturing conditions used in the MTT assay for up to 72 h. Viable cells were collected and counted using trypan blue dye (Sigma-Aldrich; cat #T8154-100ML) exclusion method after 72 h (Strober, 2001). Cell viability was expressed as percentage growth relative to condition “All Factors.” The data are derived from the mean of triplicates wells.

### Statistical Analyses

Statistical analysis was performed using GraphPad Prism 7 software. Student’s *t*-test was used to analyze gene expression. To determine statistical significance of differences in *in vitro* cell proliferation and viability of the unaffected and tumor patient-derived AUB-PrC cells between the three culturing conditions related to EGF, two-way ANOVA test was performed followed by multiple comparisons using Bonferroni *post hoc* analysis. All *P* < 0.05 were considered significant.

## Results

### Isolation of Patient-Derived Prostate Epithelial (AUB-PrC) Cells From 3D Organoids

**FIGURE 1:**
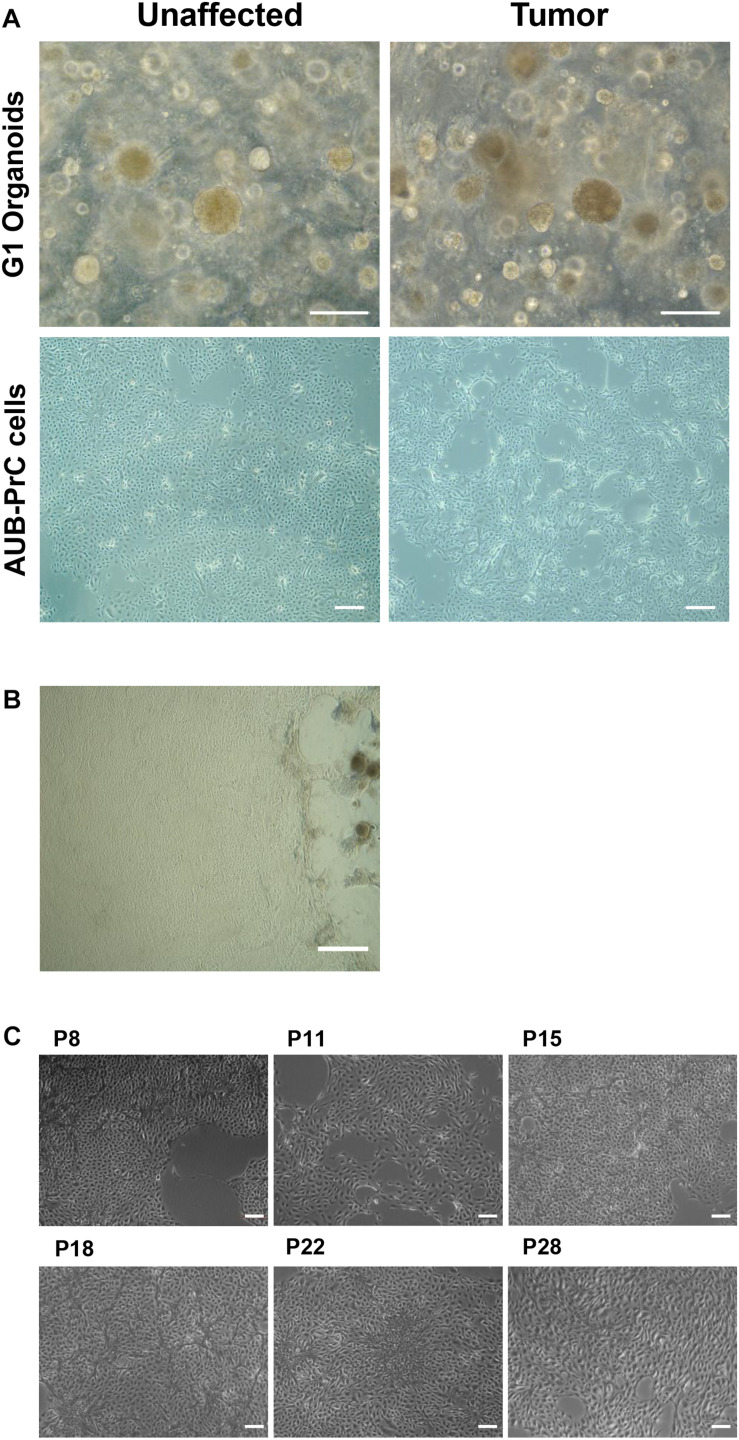
Isolation of patient-derived AUB-PrC cells using organoids culture conditions. Representative bright-field images showing established prostate organoids (generation 1, G1) from unaffected and tumor prostate patient samples {patient 2 with Grade Group 3 [Gleason Score 7(4 + 3)]; patient characteristics in Supplementary Table S1} grown in culture (Scale bar = 200 μm) **(A)**, and AUB-PrC cells established and grown on 1% collagen type-I coated plates (Scale bar = 200 μm) **(B)** maintaining their healthy morphology when propagated for continuous passages (passages P8, P11, P15, P18, P22, and P28 are shown) reaching more than 28 passages with successful repeated freeze-thaw cycles (Scale bar = 100 μm) **(C)**.

Starting from the prostate organoids protocol and using the same culture medium (Cheaito et al., under review), AUB-PrC two-dimensional (2D) cell lines (unaffected and tumor) were successfully generated. After the 1st week of organoids culture (Figure 1A), cells started invading the three-dimensional (3D) Matrigel^TM^ droplet and proliferating in 2D cultures on the bottom of the plates (Figure 1B). Collagen-I allowed the spreading of cells and maintained their healthy morphology when propagated for continuous passages reaching more than 28 passages with successful repeated freeze-thaw cycles (Figure 1C).

### Immunofluorescence Characterization of AUB-PrC Cells for Prostate Epithelial Lineage Markers

**FIGURE 2:**
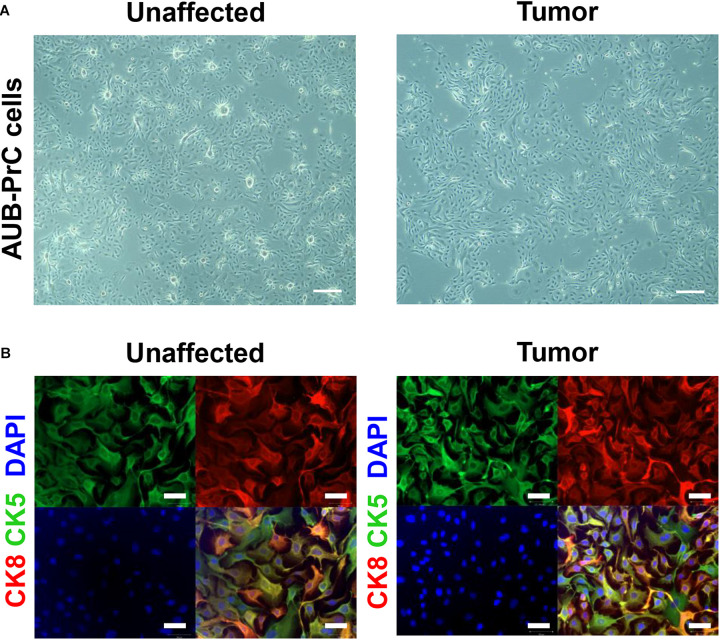
Morphologic and immunofluorescent epithelial lineage characterization of AUB-PrC cells. **(A)** Representative bright-field images of AUB-PrC cells from unaffected and tumor prostate patient samples {patient 1 with Grade Group 5 [Gleason Score 9(5 + 4)]; patient characteristics in Supplementary Table S1}, displaying key characteristics of epithelial cells and showing that when cells are far apart from each other, they form extensions that fill the gaps *in vitro*. Scale bar = 200 μm. **(B)** Representative immunofluorescence images of AUB-PrC cells from unaffected and tumor prostate patient samples {patient 3 with Grade Group 2 [Gleason Score 7(3 + 4)]; patient characteristics in Supplementary Table S1} stained for the lineage epithelial cell markers, CK8 (luminal epithelial cell marker) and CK5 (basal epithelial cell marker), and the nuclear counterstain DAPI illustrating CK8 + /CK5 + intermediate character. Scale bars = 50 μm.

Using immunofluorescence, we characterized AUB-PrC cells derived from three treatment-naïve patients for prostate epithelial lineage markers. AUB-PrC cells displayed key characteristics of epithelial cells, showing that when such cells are further apart from each other, they form extensions that fill the gaps *in vitro*. We also demonstrated that tumor AUB-PrC cells display elongated epithelial cell features compared to their unaffected counterparts (Figure 2A and Supplementary Figure S1). Those key characteristics of epithelial cells show that when such cells are further apart from each other, they tend to form extensions to fill the gaps *in vitro*. Morphological differences were further depicted in immunofluorescent staining of AUB-PrC cells using lineage epithelial cell markers, including CK8 (luminal epithelial cell marker) and CK5 (basal epithelial cell marker). Both unaffected and tumor AUB-PrC cells showed evidence of CK8 + and CK5 + expression (Figure 2B and Supplementary Figure S2A) with no difference in expression noticed between unaffected and tumor cells. Since PCa cells are more prone to lose their epithelial phenotype in favor of a more mesenchymal phenotype, which is a trigger for aggressiveness and metastasis (Cheaito et al., 2019), we employed immunofluorescent staining of tumor AUB-PrC cells using CK8 (luminal epithelial cell marker) and vimentin (VIM; mesenchymal cell marker), showing evidence of VIM + expression (Supplementary Figure S2B).

### Expression of Prostate Epithelial Lineage Genes in AUB-PrC Cells

Next, we sought to characterize the novel patient-derived cell lines with respect to specific primers relative to *GAPDH*, for experimental value *n* = 1, done in technical duplicates, using quantitative reverse transcription-PCR (qRT-PCR) analysis. We assessed mRNA expression levels of several genes including epithelial cell markers (*CDH1* and *CDH2*), prostate luminal epithelial markers (*CK8* and *CK18*), basal epithelial markers (*NKX3.1* and *P63*), and other markers known to be aberrated in the prostate or maintain stemness (*AR* and *CD44*, respectively).

**FIGURE 3:**
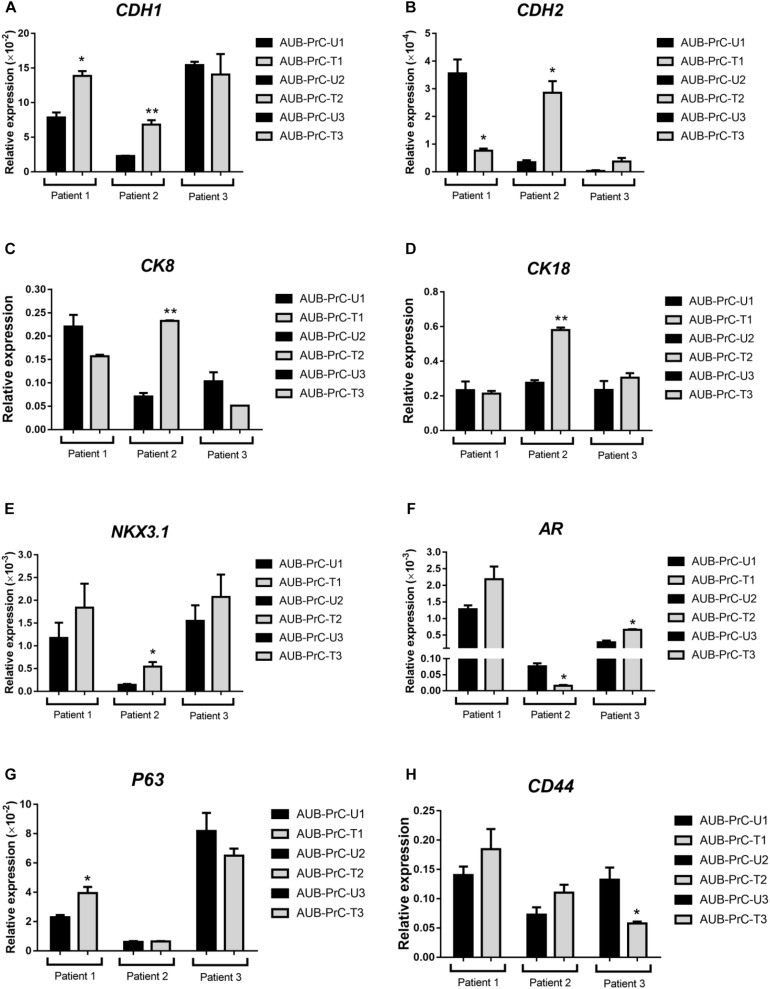
Expression of different prostate epithelial lineage and stem cell markers determined using qRT-PCR analysis. qRT-PCR expression analysis depicted aberrations in epithelial cell markers (**A:** *CDH1* and **B:** *CDH2*), prostate luminal epithelial markers (**C:** *CK8* and **D:** *CK18*), basal epithelial cell marker (**E:** *NKX3.1*), stem cell markers (**G:** *P63* basal stem cell marker and **H:** *CD44*), and other markers know to aberrated in PCa (**F:** *AR*). For each patient {patient 1 with Grade Group 5 [Gleason Score 9(5 + 4)]; patient 2 with Grade Group 3 [Gleason Score 7(4 + 3)]; patient 3 with Grade Group 2 [Gleason Score 7(3 + 4)]; patients characteristics in Supplementary Table S1}, reactions were performed in biological duplicates and expressions of individual genes was normalized to the house keeping gene *GAPDH*. Data were plotted relative to the unaffected cells (AUB-PrC-U) for each patient. Relative expression value are presented as means + SD (two technical replicates) (*P <0.05; **P <0.01; by Student’s *t*-test).

In our study, patient 1 showed significantly increased expression level of E-cadherin (*CDH1*) and decreased levels of N-cadherin (*CDH2*) (Figures 3A,B). Although patient 1 has high ISUP group 5, this does not exclude the possibility that the cancer cells still retain cell adhesion epithelial phenotype. This is consistent with the epithelial behavior of those cells which when grown apart from each other in culture tend to form extensions and fill the gaps *in vitro*, as mentioned previously. Also, we found significantly increased expression of the luminal epithelial cell markers (*CK8* and *CK18*) in patient 2 AUB-PrC cells (Figures 3C,D). Besides, a pathway known to be central to prostate cells proliferation and survival (Song et al., 2009) was found to be dysregulated in the AUB-PrC cells from all three patients, depicting upregulation of *NKX3.1* among those patients and down-regulation of *AR* in patient 2 AUB-PrC cells (Figures 3E,F). Stem cell markers, such as *P63* (basal stem cell marker) and *CD44* were found to be upregulated in AUB-PrC cells from patients 1 and 2 (Figures 3G,H). Notably, stem cell-expressing population of AUB-PrC cells may be responsible for the regenerative potential that allows these patient cells to be maintained in culture for many passages, especially cells derived from tumor samples. It is noteworthy mentioning that since patients might have different genetic backgrounds, it is expected to have them convey different gene expression profiles.

### Whole-Transcriptome Sequence Analysis of AUB-PrC Cells vs. Their Corresponding Tissues

We then sought to study transcriptomic features that signify AUB-PrC cells vs. their corresponding tissues in unaffected and tumor samples. We performed paired-end (100 base pair) RNA-sequencing using the Hi-Seq 2500 Illumina platform to delineate DEG features between patient-derived AUB-PrC cells and their corresponding tissue counterparts (two biological replicates – with technical duplicate for each – in each group).

**FIGURE 4:**
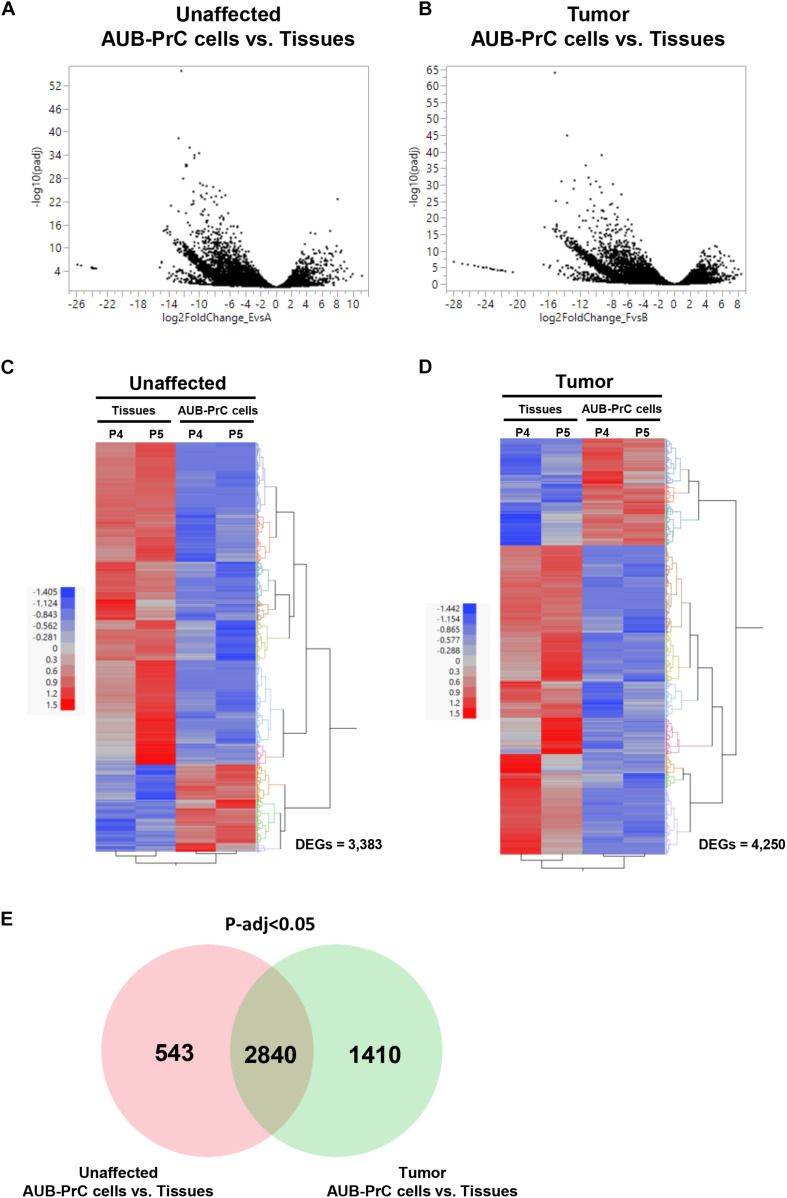
RNA-sequencing of patient-derived AUB-PrC cells relative to their corresponding tissues in unaffected and tumor samples. RNA-Seq was performed using the Hi-Seq 2500 Illumina platform to delineate the differentially expressed genes (DEG) as described in “Materials and Methods” section. Volcano plots **(A,B)** and Venn diagram **(E)** demonstrating an overview of the DEGs. The threshold was set at *p*-adjusted < 0.05. Differentially expressed transcripts (*n* = 3,383 and 4,250 in unaffected and tumor samples) between AUB-PrC cells and tissue counterparts {two biological replicates and two technical duplicates in each group; patient 4 with Grade Group 1 [Gleason Score 6(3 + 3)] and patient 5 with Grade Group 3 [Gleason Score 7(4 + 3)]; patients characteristics in Supplementary Table S1} were identified using statistical criteria detailed in “Materials and Methods” section. **(C,D)** Heatmaps and the hierarchical cluster analyses of the differentially expressed genes for unaffected **(C)** and tumor **(D)** samples. Red represents the upregulated genes and blue represents the downregulated genes.

Based on statistical significance using *p*-adj < 0.05 cut-off, we identified 3,383 and 4,250 transcripts that were significantly differentially expressed between the AUB-PrC cells vs. their corresponding tissue counterparts in each of the unaffected and tumor samples, respectively (722 up-regulated and 2,661 down-regulated in unaffected samples and 1,092 up-regulated and 3,158 down-regulated in tumor samples; Supplementary Tables S5, S6). The volcano plots in Figures 4A,B represent an overview of DEGs with a threshold set at *p*-adj < 0.05. The DEG expression heatmaps for unaffected and tumor samples are presented in Figures 4C,D, and interestingly the venn diagram identified DEG that are uniquely expressed in the Unaffected samples (543) vs. the tumor samples (1410) (Figure 4E and Supplementary Table S7).

### GO Term Analysis Venn Diagram

**FIGURE 5:**
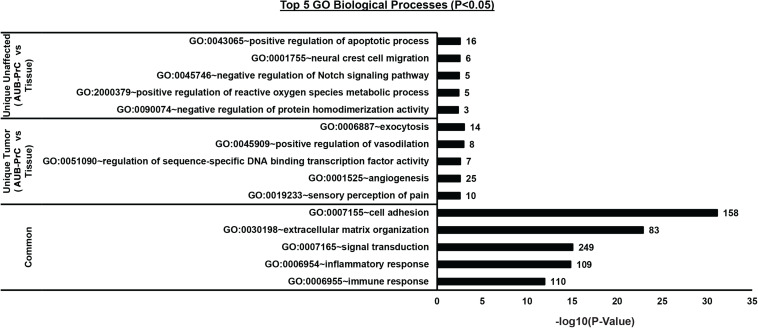
Biological processes Gene Ontology (GO) analysis of the DEGs lists between AUB-PrC cells vs. tissue in unaffected and tumor samples. [Unique genes in unaffected AUB-PrC cells vs. tissue (543 DEGs), unique genes in tumor AUB-PrC cells vs. tissue (1410 DEGs), and common genes between unaffected and tumor (2840 DEGs)] were isolated based on the Venn diagram and further analyzed via DAVID platform (Huang Da et al., 2009) to identify the top 5 GO biological processes.

Gene Ontology (GO) analysis of the DEGs lists [unique genes in unaffected AUB-PrC cells vs. tissue [543 DEGs], unique genes in tumor AUB-PrC cells vs. tissue (1410 DEGs), and common genes between unaffected and tumor (2840 DEGs)] isolated based on the venn diagram were further analyzed via DAVID platform (Huang Da et al., 2009). Focusing solely on biological processes with a cutoff of *p* < 0.05, several terms were identified. In the unaffected AUB-PrC cells vs. tissue, there were 41 terms (Supplementary Table S8) showing top five significant enrichments of GO:0043065∼positive regulation of apoptotic process (16 genes), GO:0001755∼neural crest cell migration (6 genes), GO:0045746∼negative regulation of Notch signaling pathway (5 genes), GO:2000379∼positive regulation of reactive oxygen species metabolic process (5 genes), and GO:0090074∼negative regulation of protein homodimerization activity (3 genes) (Figure 5—top panel). In the tumor AUB-PrC cells vs. tissue, there were 58 term (Supplementary Table S9) showing top 5 significant enrichments GO:0006887∼exocytosis (14 genes), GO:0045909∼positive regulation of vasodilation (8 genes), GO:0051090∼regulation of sequence-specific DNA binding transcription factor activity (7 genes), GO:0001525∼angiogenesis (25 genes), and GO:0019233∼sensory perception of pain (10 genes) (Figure 5—middle panel). Whereas common genes shared between both DEGs list consisted of 414 terms (Supplementary Table S10) that included top 5 significant enrichments of GO:0007155∼cell adhesion (158 genes), GO:0030198∼extracellular matrix organization (83 genes), GO:0007165∼signal transduction (249 genes), GO:0006954∼inflammatory response (109 genes), and GO:0006955∼immune response (109 genes) (Figure 5—lower panel).

### AUB-PrC Cells Demonstrate Upregulation of Prostate Epithelial Lineage mRNA Expression

DEGs genes that characterize prostate basal, luminal, and intermediate epithelia (Wang et al., 2001) along with other genes known to be aberrated in prostate tissue and cancer and growth factors genes were found to be dysregulated in AUB-PrC cells *vs.* tissues in unaffected and tumor samples (Table 1).

**TABLE 1:** List of DEGs genes commonly representing prostate lineage markers and other markers related to prostate diseases along with their expression levels in AUB-PrC cells relative to their corresponding tissue counterparts in unaffected and tumor samples.

Markers	DEGs gene symbols	DEGs gene names	AUB-PrC cells vs. Tissues			
			Unaffected samples		Tumor samples	
			FC (log2)	*p*-adj	FC (log2)	*p*-adj
Basal epithelial	*KRT5*	Keratin 5	4.81	0.00034	4.77	0.00034
	*KRT14*	Keratin 14	5.7	1.51E-09	4.37	6.16E-06
	*TP63*	Tumor protein p63	2.32	0.07827	2.96	0.01257
	*NKX3.1*	NK3 Homeobox 1	–3.3	0.00118	–2.9	0.00498
Luminal epithelial	*KRT8*	Keratin 8	1.48	0.16463	2.99	0.00043
	*KRT13*	Keratin 13	2.78	0.40733	5.12	0.05105
	*KRT18*	Keratin 18	1.04	0.40985	2.35	0.01291
Intermediate epithelial	*KRT19*	Keratin 19	3.57	0.01804	2.99	0.04917
Cadherins	*CDH1*	E-cadherin	1.55	0.27936	2.05	0.09614
	*CDH2*	N-cadherin	–5.08	0.0475	–4.34	0.00446
Prostate cancer related	*AR*	Androgen receptor	–3.12	0.01436	–2.81	0.02722
	*VIM*	Vimentin	–1.99	0.25782	–1.05	0.58212
	*CD44*	CD44 Molecule	2.27	0.00253	3.22	4.37E-06
	*FOXA1*	Forkhead Box A1	0.92	0.58522	1.19	0.39047
	*TWIST1*	Twist Family BHLH Transcription Factor 1	–2.06	0.37524	–4.25	0.01980
	*IL6*	Interleukin 6	–7.78	0.00111	–10.92	9.39E-07
	*TMPRSS2*	Transmembrane Serine Protease 2	–2.90	0.08178	–4.22	0.00371
	*ERG*	ETS Transcription Factor ERG	–3.40	2.49E-05	–3.27	4.73E-05
Growth factors	*FGF10*	Fibroblast Growth Factor 10	–9.38	9.95E-05	–9.55	6.35E-05
	*FGFR1*	Fibroblast growth factor receptor 1	–2.61	0.00081	–3.33	7.76E-06
	*FGF2*	Basic fibroblast growth factor (β-FGF)	–3.44	0.00076	–3.05	0.00242
	*EGFR*	Epidermal growth factor receptor	1.40	0.29082	2.18	0.04582
	*EGF*	Epidermal growth factor	0.84	0.77265	–1.77	0.41791
	*NTF3*	Neurotrophin-3	–6.68	0.00198	–8.20	0.00072

*DEG, differentially expressed gene; FC, fold change.*

Next, we pursued to confirm some of the gene features that were identified by the RNA-Seq analysis to be differentially expressed in AUB-PrC cells relative to their corresponding tissue counterparts. RNA-Seq analysis had revealed the upregulation of the prostate luminal epithelial lineage marker *CK8* and basal stem cell marker *P63* in AUB-PrC cells compared to their tissue counterparts (Table 1). It also showed the downregulation of other genes, such as *AR*, *VIM*, and *TWIST1* in those cells. Consistent with the RNA-Seq results, quantitative real-time PCR analysis of AUB-PrC cells from patient 5 and its tissue counterparts (three technical replicates each) showed upregulation of *CK8* and *P63* genes in AUB-PrC cells compared to their tissue counterparts (Supplementary Figure S3A, upper panels) and downregulation of *AR*, *VIM*, and *TWIST1* (Supplementary Figure S3A, lower panels). Molecular characterization was also performed on AUB-PrC cells and tissue sections from patients 4 and 5 on which RNA-Seq analysis was done. Immunofluorescent staining showed evidence of high CK8 + and CK5 + expression among cells and their counterpart tissues, with low expression of VIM+ (Supplementary Figure S3B). Results are consistent with the RNA-Seq results showing upregulation of *CK8* and *CK5* genes and downregulation of *VIM* (Table 1).

### GSEA Identifies Enrichment of Growth Factor and Epithelial Lineage-Related Signaling Pathways in AUB-PrC Cells Relative to Their Tissue Counterparts

**FIGURE 6:**
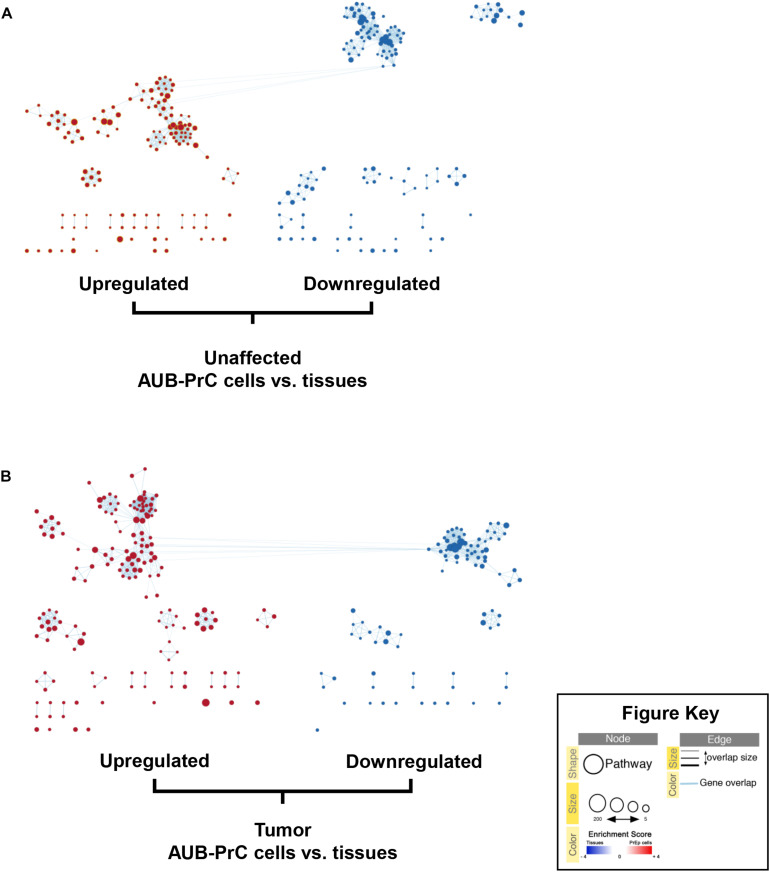
Enrichment maps of pathways enriched in upregulated genes (red) and downregulated genes (blue) in AUB-PrC cells vs. tissues. Enrichment maps of pathways among prostate unaffected **(A)** and tumor **(B)** samples were built using EnrichmentMap analysis on Cytoscape 3.7.2 software. Each node (circle) represents a distinct pathway (red representing upregulated pathways and blue representing downregulated pathways), and edges (lines) represent the number of genes overlapping between two pathways, determined using the similarity coefficient.

We sought to build enrichment maps to evaluate DEGs and their related pathways in our datasets (Figure 6 and Supplementary Tables S11, S12) using Cytoscape 3.7.2 software (EnrichmentMap tool). Using gene set enrichment analysis (GSEA), we identified significantly altered pathways in AUB-PrC cells relative to their corresponding tissue counterparts (Supplementary Tables S13, S14). Results indicated significant differences (FDR < 0.01, NOM *p* < 0.05) in the enrichment of the gene sets database (Human_GOBP_AllPathways_no_GO_iea_April_01_2020_ symbol.gmt; Supplementary Table S4). We selected the 20 most significantly enriched signaling pathways, based on normalized enrichment score (NES) (Supplementary Figures S4, S5). Results indicated the unaffected data set was enriched for cell cycle pathways, E2F signaling, TP53 transcriptional regulation, Rb signaling, mitosis, and epithelial differentiation pathways while the treated data set was enriched for cell cycle pathways, PLK1 signaling, DNA irradiation damage and cellular response via ATR, and epithelial differentiation pathways. Other pathways that are found to be enriched in AUB-PrC cells and are of specific interest in prostate diseases include cell adhesion molecules (CAMs), cholesterol biosynthesis and metabolism pathways, ErbB-2 signaling, c-Myc pathway, and other cancer pathways which can be further explored in future work to look for novel potential therapeutic targets for PCa.

### EGF Is Essential to Grow Patient-Derived AUB-PrC Cells in Culture

**FIGURE 7:**
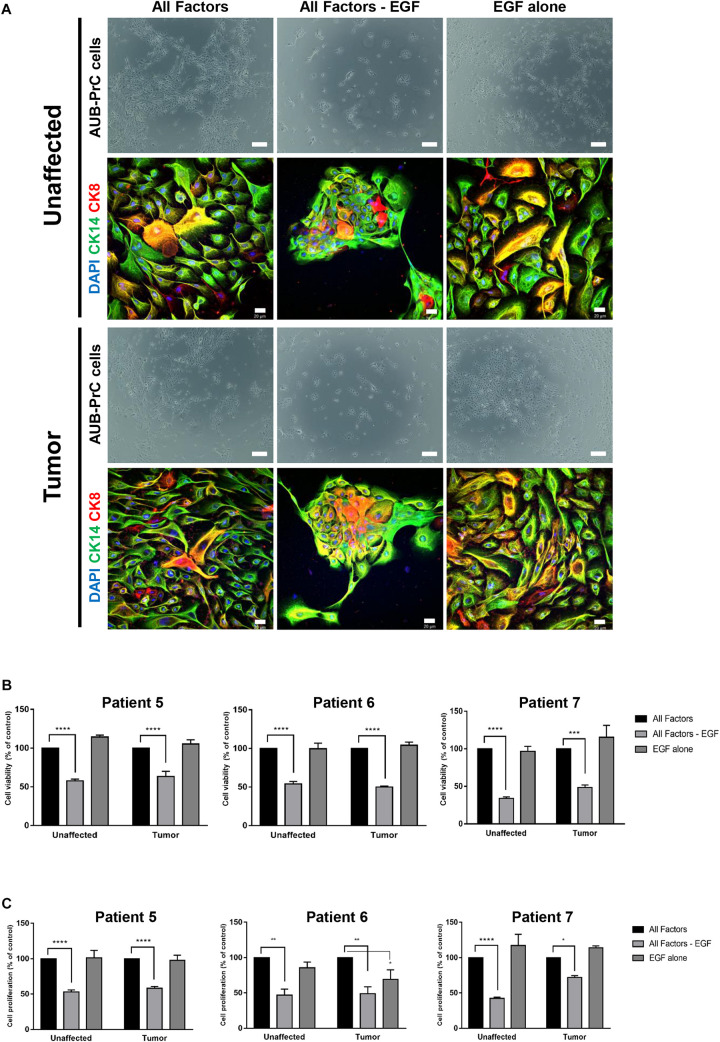
EGF is essential to grow patient-derived AUB-PrC cells in culture. **(A)** Representative bright-field images of AUB-PrC cells established from unaffected and tumor organoids {patient 7 with Grade Group 3 [Gleason Score 7(4 + 3)]; patient characteristics in Supplementary Table S1} and grown under different conditions; condition 1 “All factors” with prostate organoids growth medium, condition 2 “All factors -EGF” with prostate organoids growth medium without EGF, and condition 3 “EGF alone” with adDMEM/F12 with EGF only (10 ng/mL). Scale bar = 200 μm. Representative Immunofluorescent images of AUB-PrC cells {patient 7 with Grade Group 3 [Gleason Score 7(4 + 3)]; patient characteristics in Supplementary Table S1} grown under different conditions as described previously are stained with the prostate lineage epithelial markers CK8 and CK14. The nuclei were stained with anti-fade reagent Fluorogel II with DAPI. The images were acquired using the Zeiss LSM 710 laser scanning confocal microscope (Zeiss), and images were processed using the Carl Zeiss ZEN 2013 image software. Scale bar = 20 μm. **(B)** Cell viability was determined using the trypan blue exclusion assay. **(C)** Cell proliferation was determined in triplicates using the MTT cell proliferation assay {patient 5 with Grade Group 3 [Gleason Score 7(4 + 3)]; patient 6 with Grade Group 2 [Gleason Score 7(3 + 4)]; patient 7 with Grade Group 3 [Gleason Score 7(4 + 3)]; patients characteristics in Supplementary Table S1}. Data represent an average of triplicate measurements and are reported as mean ± SEM. (Two-way ANOVA; *P < 0.05, **P < 0.01, ***P < 0.001, ****P < 0.0001; different conditions compared to condition 1 “All Factors,” Bonferroni’s multiple comparisons test).

Based on an observation made during the organoids’ optimization experiment (Cheaito et al., under review), we noticed that EGF withdrawal from the medium affected the ability to derive AUB-PrC cells negatively (data not shown). So, we further investigated the importance of EGF for the growth of AUB-PrC cells by growing them under 3 conditions; condition 1 includes prostate organoids growth medium (as described in Supplementary Table S2), condition 2 includes prostate organoids growth medium without EGF, and condition 3 includes adDMEM/F12 with EGF only (10 ng/mL) (Figure 7A). AUB-PrC cells derived from tissue samples from 3 different patients (Patients 5, 6, and 7), including the unaffected and the tumor sample, were seeded under the three different conditions. MTT and Trypan Blue assays were performed showing, a significant reduction in cell viability and cell proliferation when EGF was removed from the medium, while EGF alone demonstrated the ability to maintain the growth of AUB-PrC cells. Indeed, there was no significant difference in both cell proliferation and cell viability between condition 1 and condition 3 for all three patients’ derived AUB-PrC cells (Figures 7B,C). To further confirm that condition 3 “EGF alone” can support the growth of both luminal and epithelial cells, AUB-PrC cells growing under 3 conditions described above were immunostained with luminal marker CK8 and basal marker CK14. The results obtained showed similar morphologies and expression patterns of luminal and basal markers in both condition 1 and condition 3, which confirms that EGF alone can substitute the cocktail of 12 components included in condition 1.

## Discussion

Epithelial organ remodeling (such as PCa, breast cancer, and colon cancer) is a major contributing factor to worldwide morbidity and mortality. It is difficult to translate basic epithelial research into clinical therapy due to the lack of relevant preclinical models (Hynds and Giangreco, 2013). The challenges of PCa research include inter- and intra-patient heterogeneity and the scarcity of appropriate *in vitro* and *in vivo* models that depict the vast molecular aberrations that occur in PCa (Van Bokhoven et al., 2003; Tsai et al., 2018). In this context, many genetic aberrations in PCa are poorly studied, and their effects on therapeutic response are not known (Vela and Chen, 2015). Despite its prevalence, PCa has proven very difficult to propagate *in vitro* and is highly underrepresented with very few cell lines available among the thousands of cancer cell lines in public repositories (Gao et al., 2014).

The heterogeneous nature of PCa has made it difficult to understand the factors involved in the onset and progression of the disease (Shen and Abate-Shen, 2010). In the last few years several efforts have been made to delineate the complex genomic landscape of PCa (Baca and Garraway, 2012). Moreover, considering that PCa is fairly indolent, the development of treatment approaches that delay its onset or progression is likely to have a significant impact on outcome. Indeed, the scarcity of human PCa cell lines has always hindered our understanding of the disease etiology and progression, and therefore the need for novel cell lines representing the heterogeneity of the disease is of eminent importance. Along those lines and starting from organoids, we aimed at generating novel patient-derived cell lines representing unaffected and tumor prostate tissues.

Starting from the organoids protocol and using the same culture medium (Cheaito et al., under review), human prostate two-dimensional (2D) cell lines (unaffected and tumor) – which we named as AUB-PrC cells – were successfully generated. After the first week of organoids culture, 2D cells started invading the three-dimensional (3D) Matrigel^TM^ droplet and proliferating on the bottom of the culture plates. These cells were successfully derived whenever organoids were established; nonetheless, their maintenance in culture was very challenging. Consequently, to maintain them in culture, we attempted to optimize the culture conditions by using different matrices. Interestingly, collagen-I allowed the spreading of cells and maintained their healthy morphology when propagated for continuous passages reaching more than 28 passages. The favored adhesion of PCa cells to collagen-I represents a possible explanation for these results. Indeed, the most frequent site of human PCa metastasis is the bone and collagen-I represents the most abundant protein within the skeleton (Buckwalter et al., 1996). In addition, it has been previously demonstrated that collagen-I induces the attachment and proliferation of PCa cells (Kiefer and Farach-Carson, 2001).

We sought to characterize the novel patient-derived AUB-PrC cells using immunofluorescence (IF), qRT-PCR, and RNA-Seq analyses (Table 2). AUB-PrC cells depicted a distinctive epithelial cell morphology expressing CK8 and CK5 prostate epithelial lineage markers. Yet, PCa cells are more prone to lose their epithelial phenotype in favor of a more mesenchymal phenotype, which is a trigger for aggressiveness and metastasization. Indeed, our results showed that tumor AUB-PrC cells demonstrate some vimentin (mesenchymal cell marker) expression as well which further validate our point (Supplementary Figure S2). qRT-PCR results indicated a trend in mRNA expression levels of several genes involved in prostate lineage differentiation and other genes known to aberrated in PCa.

**TABLE 2:** Table summarizing major characteristics of patients and AUB-PrC cell lines generated.

Patient #	Cell lines	Gleason score	ISUP grade group	Immunofluorescent staining relative to unaffected cells			mRNA expression of different prostate epithelial lineage and stem cell markers relative to unaffected cells							
				CK8	CK5	VIM	*CDH1*	*CDH2*	*CK8*	*CK18*	*NKX3.1*	*AR*	*p63*	*CD34*
Patient 1	AUB-PrC-U1 and AUB-PrC-T1	9(5 + 4)	Grade group 5	↑	↑	↓	↑	↓	↓	↓	↑	↑	↑	↑
Patient 2	AUB-PrC-U2 and AUB-PrC-T2	7(4 + 3)	Grade group 3	↑	↑	↑	↑	↑	↑	↑	↑	↓	↑	↑
Patient 3	AUB-PrC-U3 and AUB-PrC-T3	7(3 + 4)	Grade group 2	↑	↑	↓	↓	↑	↓	↑	↑	↑	↓	↓
Patient 4	AUB-PrC-U4 and AUB-PrC-T4	6(3 + 3)	Grade group 1	↑	↑	↑	–	–	–	–	–	–	–	–
Patient 5	AUB–PrC-U5 and AUB-PrC-T5	7(4 + 3)	Grade group 3	↑	↑	↑	–	–	–	–	–	–	–	–
Patient 6	AUB-PrC-U6 and AUB-PrC-T6	7(3 + 4)	Grade group 2	↑	↑	↓	–	–	–	–	–	–	–	–
Patient 7	AUB-PrC-U7 and AUB-PrC-T7	7(4 + 3)	Grade group 3	↑	↑	↑	–	–	–	–	–	–	–	–

*AUB-PrC, American University of Beirut-Prostate Cells; ISUP, International Society of Urological Pathology; ↑, increased; ↓, decreased.*

We also studied the transcriptomic features and delineated the DEGs that signify AUB-PrC cells *vs.* their corresponding tissues in unaffected and tumor samples, followed by gene set enrichment analysis (GSEA), demonstrating upregulation of epithelial differentiation pathways and gene features. Herein, we showed that the AUB-PrC cells that have been isolated from patient-derived organoids cultures are of prostate epithelial lineage based on expression of different markers including CK5, CK8, AR, and Nkx3.1, and hence they represent the tissue of origin. Nevertheless, we expected to see variations in the transcriptomic analysis between the cells and their corresponding tissues because we are comparing cells grown *in vitro* in 2D *vs.* primary cells or tissues. In other words, we are comparing epithelial cells that are growing under selective pressure *in vitro* to those that are nascent non-manipulated tissues that contain intact microenvironment with all its components.

Among the DEGs identified by RNA-sequencing were upregulated ones that include keratins (*KRT5*, *KRT8*, *KRT13*, *KRT14*, *KRT18*, and *KRT19*), *TP63*, *CDH1*, *EGFR*, *CD44*, and *FOXA1*, and other downregulated genes such as *NKX3.1*, *TWIST1*, *IL6*, *TMPRSS2*, *ERG*, *AR*, *CDH2*, and growth factor genes (*FGF10*, *FGF2*, *FGFR1*, *EGF*, and *NTF3*). We sought to validate some of those genes using qRT-PCR and IF analyses and results were indeed consistent with the RNA-Seq data. Interestingly, the observed mRNA expression patterns recapitulate the architecture of prostate tissues where luminal secretory cell layers express prominent levels of *CK8* and *CK18*, underlying basal cell layers express *CK5*, *CK14* and *TP63*, and intermediate epithelial cells express *KRT19* (Wang et al., 2001; Van Leenders and Schalken, 2003; Peehl, 2005; Cheaito et al., 2019). *KRT13*, which was upregulated in AUB-PrC cells, has been also proposed to be a marker of stem/progenitor-like cell state. In PCa, this gene has been shown to be enriched in benign stem-like cells displaying androgen-resistance and was identified in tumors that have the potential to metastasize to the bone (Liu et al., 2016). Likewise, *TWIST1* which plays a role in PCa bone metastasis, was downregulated in AUB-PrC cells in our study (Gajula et al., 2013; Chang et al., 2015).

Intercellular adhesion is a key factor in epithelial tissue morphogenesis and maintenance, and disruption of this adhesion is an important factor in cancer (Balzer and Konstantopoulos, 2012). Cadherins are a family of calcium-dependent cell– CAMs with well-established roles in cell–cell recognition, intercellular junction organization and cell differentiation. The role of cadherins, particularly the epithelial (E)-cadherin, has been studied in detail in relation to metastatic potential and prognosis in carcinoma. In our study, RNA-seq revealed upregulation of epithelial *CDH1* and downregulation of mesenchymal *CDH2*, verifying the epithelial nature of AUB-PrC cells (Tomita et al., 2000).

One of the initiating events in prostate tumorigenesis is downregulation of the homeobox gene *NKX3.1*. It is described as the “gatekeeper” for PCa initiation (Barbieri et al., 2013), and was found to be downregulated in AUB-PrC cells in our study. Chromosomal rearrangements involving the *ETS* family of transcription factors, such as *TMPRSS2-ERG* fusions, are mostly detected after initiation and not as an initial event, thus they are commonly associated with PCa progression (Tomlins et al., 2005; Shen and Abate-Shen, 2010). In our human AUB-PrC cells, those genes were found to be downregulated. Along the line, AUB-PrC cells demonstrated downregulation of growth factor genes including *FGF10*, *FGF2*, *FGFR1*, *EGF*, and *NTF3*, all of which are essential for development and progression of PCa (Polnaszek et al., 2003; Memarzadeh et al., 2007; Corn et al., 2013; Yang et al., 2013; Mandel et al., 2018).

In this same context, and although EGF was designated as an essential component for establishing and maintaining prostate organoids in culture (Karthaus et al., 2014), we were interested in studying its effect on the *in vitro* culturing and growth of AUB-PrC cells. In brief, we investigated the importance of EGF by growing AUB-PrC cells in three different conditions: prostate organoids growth medium (will all 12 factors), prostate organoids growth medium without EGF, and adDMEM/F12 media with EGF only. Remarkably, our results demonstrated enhanced growth and maintenance of those cells in the presence of EGF alone, while a significant reduction in cell viability and proliferation was noticed when EGF was removed from the medium. These data are consistent with the substantial role of EGF in stimulating cell motility and migration of epithelial cells from various tumors, including PCa (Lu and Kang, 2010; Montanari et al., 2017). Further, we stained the cells grown under the three conditions with prostate luminal epithelial marker CK8 and basal epithelial marker CK14, and found similar cell morphologies and expression patterns in conditions 1 (complete organoids media) and 3 (EGF alone), confirming that EGF by itself is sufficient to substitute the cocktail of 12 components included in condition 1.

Lastly, it is important to emphasize that it is very crucial to establish new cell line models of cancers especially when some of those are scarce as in the case of PCa. Cancer cell lines are considered powerful tools for studying the mechanisms of tumorigenesis especially if the cancer harbors heterogeneity features such as in PCa. Those cancer cell lines are considered fundamental pre-clinical models to assess the efficacy of anti-cancer therapeutics. The available cell lines in PCa do not really recapitulate the huge heterogeneity of the disease and data inferred from small number of cell lines cannot be really generalized as a representative of the pathophysiology of that disease. The major PCa cell lines used are of Caucasian origin (LnCap, DU145, PC3, and VcaP) and hence might not genetically represent the different world populations. Our novel cell lines represent a novel cohort of Middle Eastern patients. Importantly, those novel cell lines are derived from treatment-naïve patients and therefore the cancer cells are considered primitive in terms of treatment response. This can shed more light on the etiology of the disease as it will not be masked by different therapeutic modalities.

### Limitations

Our work has several limitations. First, we acknowledge that the sample size might be small, but since we are dealing with patient tissues, it is indeed difficult to obtain large number of prostate tissues to work on just after the surgery. Second, some experiments were not performed on all the seven patients included, and this is due to the fact that obtaining tissue samples from patients is challenging including the small size of the certain samples that we receive and the small number of cells we get. Third, although samples were taken from each patient from the area most likely to be involved with cancer (from the core of the cancerous lesion) and from the unaffected area (far from the tumor site) based on an assessment made by the urologist and pathologist, no definite conclusion can be made to whether the unaffected sample is not genetically modified or might contain niche of cancerous cells. Forth, since PCa starts as an adenocarcinoma (epithelial origin), we tend to refer to the cell lines as epithelial PCa cell lines. However, PCa cells are more prone to lose their epithelial phenotype in favor of a more mesenchymal phenotype, which is a trigger for aggressiveness and metastasization. Herein, our results showed that tumor AUB-PrC cells demonstrate some vimentin (mesenchymal cell marker) expression as well. Fifth, we acknowledge that it is crucial to assess the AUB-PrC cell lines’ ability to engraft in animal models to provide information also about its potential employment *in vivo*, which can be employed in future studies. In addition, 3D culture experiments using Matrigel or Collagen Type I can be performed also to try and distinguish between malignant and non-malignant cells. Also, validating some of the significantly DEGs at a protein level using western blotting is interesting to be addressed in future studies assessing molecular aberration and signaling underlying our newly developed AUB-PrC cell lines. Sixth, our RNA seq results revealed that growth factors are among the DEGs identified in AUB-PrC cells vs. tissues, including *FGF10*, *FGF2*, *FGFR1*, and *NTF3*, all of which are essential for development and progression of PCa. For the scope of this paper, we have only worked on EGF. Nevertheless, it would be very interesting to assess the roles of the other growth factors. Lastly, and as all those newly derived cells are considered biological replicates from unique patients and therefore represent different cell models, it becomes crucial to subject them to targeted sequencing or whole genome sequencing to fully characterize the genomic landscape of each cell line/patient.

## Conclusion

The derivation of novel models to express the diverse array of aberrations seen in PCa is essential in detecting specific stages of the disease, classifying PCa based on specific molecular alterations, and selecting the most appropriate therapy for each patient. In this manuscript, we were able to generate and characterize different cell models representing different PCa patients from Middle-Eastern background and having a common feature of being treatment-naïve. We successfully demonstrated the importance of growth factors in modeling of prostate diseases by showing that the newly isolated prostate cells are capable of growing in culture in the presence of EGF alone. Yet, it is of utmost importance to further analyze the differential transcriptomic features between tumor and unaffected samples to better understand PCa at a subcellular level. Our findings provide a prospect to better understand prostate diseases, especially PCa, and pave the way for deciphering the mechanisms that lead to PCa development and progression, and ultimately improving prognostic abilities and treatments.

## Data Availability Statement

The datasets presented in this study can be found in online repositories. The names of the repository/repositories and accession number(s) can be found in the article/Supplementary Material.

## Ethics Statement

The studies involving human participants were reviewed and approved by Institutional Review Board (IRB) of the American University of Beirut. The patients/participants provided their written informed consent to participate in this study.

## Author Contributions

KC: investigation, methodology, writing- original draft preparation, writing- reviewing and editing, visualization, validation. HB: resources, software, formal analysis, investigation, methodology, data curation, writing- original draft preparation, writing- reviewing and editing, visualization, validation. HJ, OH, and HM: investigation, methodology, writing- reviewing and editing, validation. AE-H and DM: investigation, writing- reviewing and editing, visualization, validation. MA-S: project administration, supervision, software, formal analysis, methodology, data curation, writing- original draft preparation, writing- reviewing and editing, visualization, validation. WA-K: conceptualization, project administration, supervision, writing- original draft preparation, writing- reviewing and editing, validation, visualization, funding acquisition. All authors contributed to the article and approved the submitted version.

## Conflict of Interest

The authors declare that the research was conducted in the absence of any commercial or financial relationships that could be construed as a potential conflict of interest.

## Footnotes

1 http://tsar.abudhabi.nyu.edu/

2 https://www.ncbi.nlm.nih.gov/geo/query/acc.cgi?acc=GSE148937

## Supplementary Material

Supplementary Figure 1 Morphologic analysis of AUB-PrC cells from patients 2 and 3. Representative bright-field images of AUB-PrC-U2 and AUB-PrC-T2 cells **(A)** and AUB-PrC-U3 and AUB-PrC-T3 cells **(B)**, displaying key characteristics of epithelial cells {patient 2 with Grade Group 3 [Gleason Score 7(4 +3)]; patient 3 with Grade Group 1 [Gleason Score 7(3 +4)]; patients characteristics in Supplementary Table S1}. Scale bar 200 μm.

Supplementary Figure 2 Immunofluorescent epithelial lineage characterization of AUB-PrC cells for CK8/CK5 and CK8/VIM **(B)**. Representative immunofluorescence images of AUB-PrC cells from unaffected and tumor prostate patient samples {patient 1 with Grade Group 5 [Gleason Score 9(5 +4)]; patient 2 with Grade Group 3 [Gleason Score 7(4 +3)]; patient 3 with Grade Group 2 [Gleason Score 7(3 +4)]; patient 4 with Grade Group 1 [Gleason Score 6(3 +3)]; patient 6 with Grade Group 2 [Gleason Score 7(3 +4)]; patient characteristics in Supplementary Table S1} stained for the lineage epithelial cell markers, CK8 (luminal epithelial cell marker), CK5 (basal epithelial cell marker), and VIM (mesenchymal cell marker), and the nuclear counterstain DAPI illustrating CK8 +/CK5 **(A)** and CK8+/VIM **(B)** characters. Scale bars 20 μm.

Supplementary Figure 3 Validation of dysregulated gene expression in AUB-PrC cells relative to their tissue counterparts. **(A)** Upregulation of *CK8* and *P63* and downregulation of *AR*, *VIM*, and *TWIST1* in AUB-PrC cells compared to tissues [patient 5 with Grade Group 3 [Gleason Score 7(4 +3)]; patient characteristics in Supplementary Table S1} was validated by qRT-PCR and analyzed using the 2^–Δ^ ^Ct^ method by normalization to *GAPDH*. Reactions were performed in technical triplicates and expression of individual genes was normalized to the house keeping gene *GAPDH*. Data were plotted relative to the tissue counterparts. Relative expression values are presented as means + SD (three technical replicates) (^∗^*P* <0.05; ^∗∗^*P* <0.01; by Student’s *t*-test). **(B)** Representative Immunofluorescent images of AUB-PrC cells {patient 4 with Grade Group 1 [Gleason Score 6(3 +3)] and patient 5 with Grade Group 3 [Gleason Score 7(4 +3)]; patients characteristics in Supplementary Table S1} stained with the prostate lineage epithelial markers CK8, CK5, and VIM. The nuclei were stained with anti-fade reagent Fluorogel II with DAPI. The images were acquired using the Zeiss LSM 710 laser scanning confocal microscope (Zeiss), and images were processed using the Carl Zeiss ZEN 2013 image software. Scale bar 200μm.

Supplementary Figure 4 Gene set enrichment analysis (GSEA) of the top 20 signaling pathways activated in AUB-PrC cells relative to their tissue counterparts among the unaffected samples. Comparison of data sets indicated unaffected AUB-PrC cells had enrichment of cell cycle pathways, E2F signaling, TP53 transcriptional regulation, Rb signaling, mitosis, and epithelial differentiation pathways.

Supplementary Figure 5 Gene set enrichment analysis (GSEA) of the top 20 signaling pathways activated in AUB-PrC cells relative to their tissue counterparts among the tumor samples. Comparison of data sets indicated tumor AUB-PrC cells had enrichment of cell cycle pathways, PLK1 signaling, DNA irradiation damage and cellular response via ATR, and epithelial differentiation pathways.

Supplementary Table 1 Patients’ clinical characteristics.

Supplementary Table 2 Overview of specific components and their respective concentrations added to prepare human prostate organoids culture medium. Adopted and modified from Drost et al. (2016).

Supplementary Table 3 Primer sequences and annealing temperatures for select human genes.

Supplementary Table 4 Gene set database used for pathway enrichment analysis (Human_GOBP_AllPathways_no_GO_iea_April_01_2020_symbol.gmt; downloaded from http://baderlab.org/GeneSets).

Supplementary Table 5 List of all differentially expressed genes (DEGs) between AUB-PrC cells and their tissue counterparts in unaffected samples.

Supplementary Table 6 List of all differentially expressed genes (DEGs) between AUB-PrC cells and their tissue counterparts in tumor samples.

Supplementary Table 7 List of all differentially expressed genes (DEGs) between AUB-PrC cells and their tissue counterparts that are unique to unaffected samples (543), unique to tumor samples (1410), and common between the two (2840).

Supplementary Table 8 List of biological processes identified that are unique to the unaffected AUB-PrC cells vs. tissue. There were 41 terms showing top five significant enrichments of GO:0043065~positive regulation of apoptotic process (16 genes), GO:0001755~neural crest cell migration (6 genes), GO:0045746~negative regulation of Notch signaling pathway (5 genes), GO:2000379~positive regulation of reactive oxygen species metabolic process (5 genes), and GO:0090074~negative regulation of protein homodimerization activity (3 genes).

Supplementary Table 9 List of biological processes identified that are unique to the tumor AUB-PrC cells vs. tissue. There were 58 terms showing top five significant enrichments of GO:0006887~exocytosis (14 genes), GO:0045909~positive regulation of vasodilation (8 genes), GO:0051090~regulation of sequence-specific DNA binding transcription factor activity (7 genes), GO:0001525~angiogenesis (25 genes), and GO:0019233~sensory perception of pain (10 genes).

Supplementary Table 10 List of biological processes identified that are common between unaffected and tumor AUB-PrC cells vs. tissue. There were 414 terms showing top five significant enrichments of GO:0007155~cell adhesion (158 genes), GO:0030198~extracellular matrix organization (83 genes), GO:0007165~signal transduction (249 genes), GO:0006954~inflammatory response (109 genes), and GO:0006955~immune response (109 genes).

Supplementary Table 11 Gene set annotations of the enrichment map presented in Figure 6A corresponding to the unaffected AUB-PrC cells vs. tissues identified using Cytoscape 3.7.2 software (EnrichmentMap tool).

Supplementary Table 12 Gene set annotations of the enrichment map presented in Figure 6A corresponding to the tumor AUB-PrC cells vs. tissues identified using Cytoscape 3.7.2 software (EnrichmentMap tool).

Supplementary Table 13 Signaling pathways that are activated in unaffected AUB-PrC cells vs. tissues identified using gene set enrichment analysis (GSEA).

Supplementary Table 14 Signaling pathways that are activated in tumor AUB-PrC cells vs. tissues identified using gene set enrichment analysis (GSEA).
