# Supplementary figures and images for: Epidermal Growth Factor Is Essential for the Maintenance of Novel Prostate Epithelial Cells Isolated From Patient-Derived Organoids
Source: Front Cell Dev Biol. 2020 Oct 29;8:571677. doi: 10.3389/fcell.2020.571677 (PMC7658326; doi:10.3389/fcell.2020.571677)

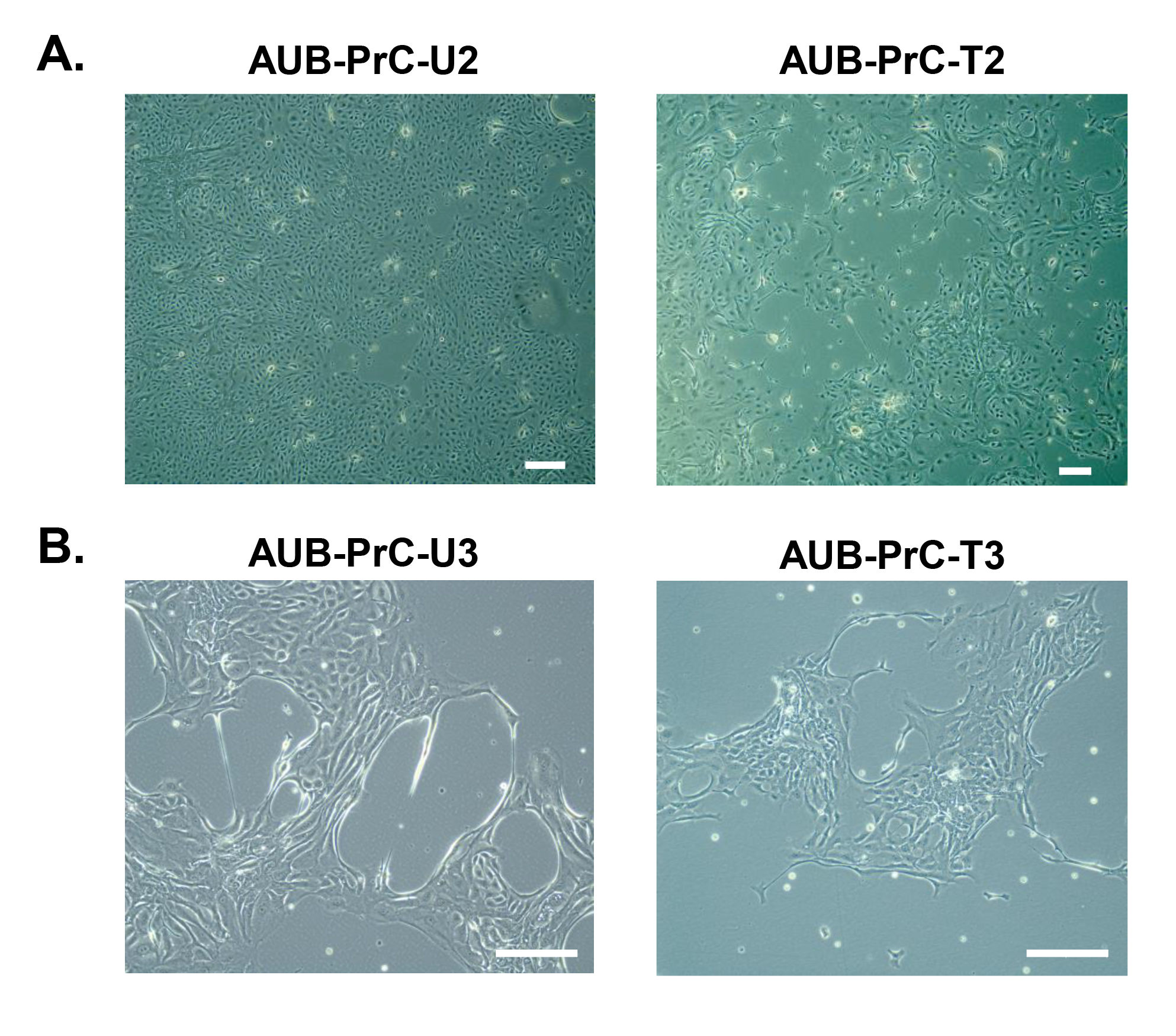

Supplement: Supplementary Figure 1 — Morphologic analysis of AUB-PrC cells from patients 2 and 3. Representative bright-field images of AUB-PrC-U2 and AUB-PrC-T2 cells (A) and AUB-PrC-U3 and AUB-PrC-T3 cells (B), displaying key characteristics of epithelial cells {patient 2 with Grade Group 3 [Gleason Score 7(4 +3)]; patient 3 with Grade Group 1 [Gleason Score 7(3 +4)]; patients characteristics in Supplementary Table S1}. Scale bar 200 μm. [file Image_1.TIF]

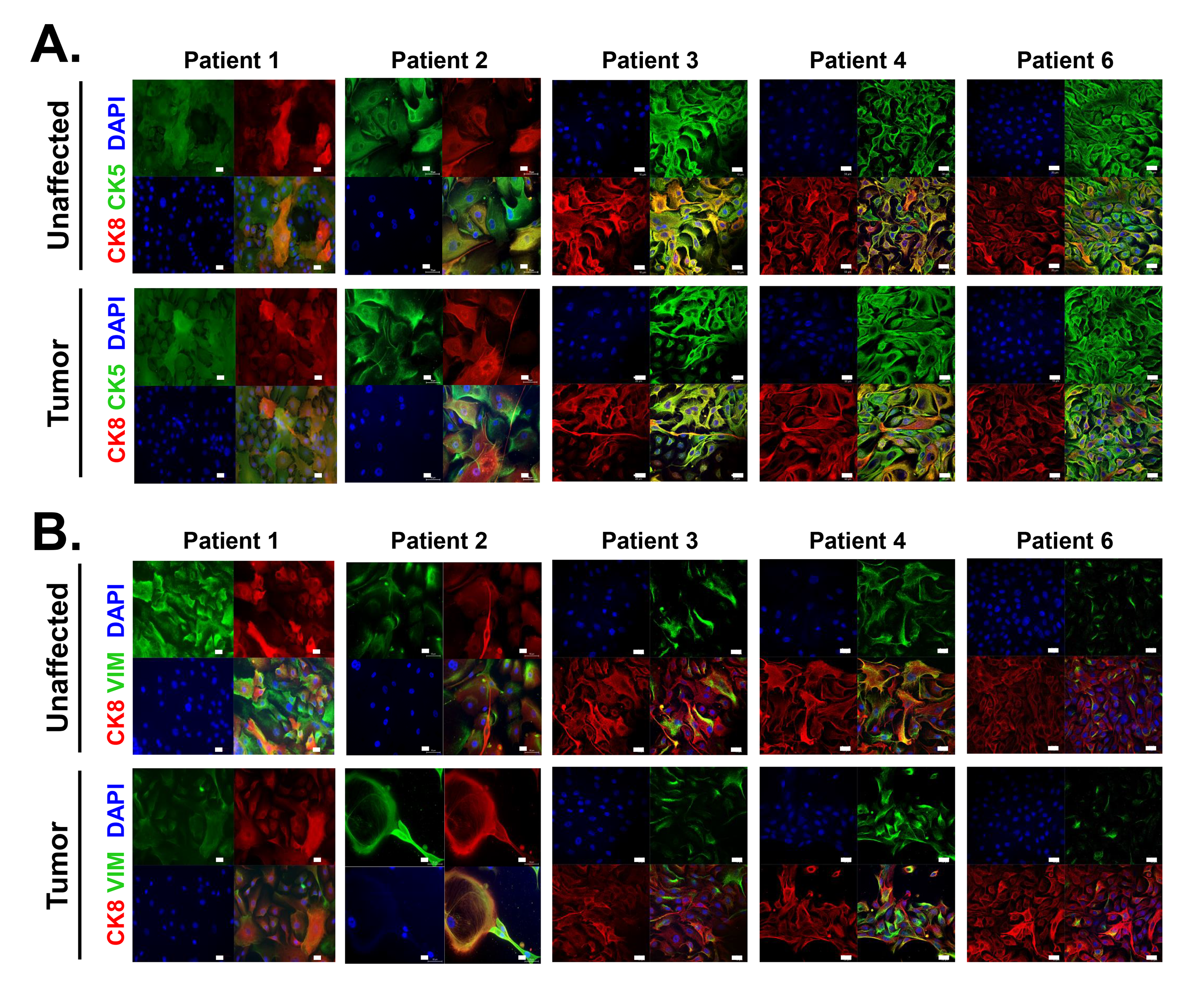

Supplement: Supplementary Figure 2 — Immunofluorescent epithelial lineage characterization of AUB-PrC cells for CK8/CK5 and CK8/VIM (B). Representative immunofluorescence images of AUB-PrC cells from unaffected and tumor prostate patient samples {patient 1 with Grade Group 5 [Gleason Score 9(5 +4)]; patient 2 with Grade Group 3 [Gleason Score 7(4 +3)]; patient 3 with Grade Group 2 [Gleason Score 7(3 +4)]; patient 4 with Grade Group 1 [Gleason Score 6(3 +3)]; patient 6 with Grade Group 2 [Gleason Score 7(3 +4)]; patient characteristics in Supplementary Table S1} stained for the lineage epithelial cell markers, CK8 (luminal epithelial cell marker), CK5 (basal epithelial cell marker), and VIM (mesenchymal cell marker), and the nuclear counterstain DAPI illustrating CK8 +/CK5 (A) and CK8+/VIM (B) characters. Scale bars 20 μm. [file Image_2.TIF]

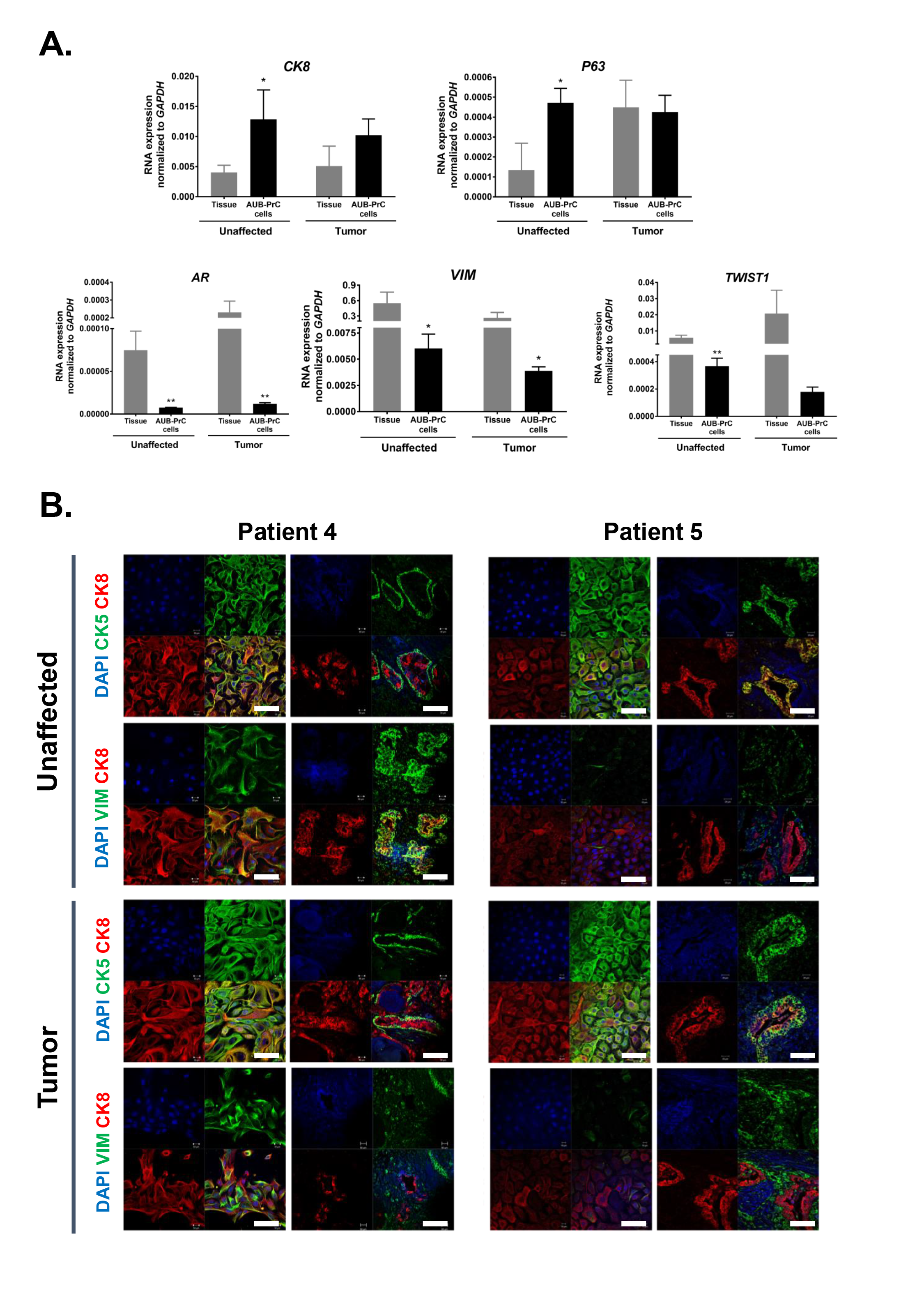

Supplement: Supplementary Figure 3 — Validation of dysregulated gene expression in AUB-PrC cells relative to their tissue counterparts. (A) Upregulation of CK8 and P63 and downregulation of AR, VIM, and TWIST1 in AUB-PrC cells compared to tissues [patient 5 with Grade Group 3 [Gleason Score 7(4 +3)]; patient characteristics in Supplementary Table S1} was validated by qRT-PCR and analyzed using the 2–Δ Ct method by normalization to GAPDH. Reactions were performed in technical triplicates and expression of individual genes was normalized to the house keeping gene GAPDH. Data were plotted relative to the tissue counterparts. Relative expression values are presented as means + SD (three technical replicates) (∗P <0.05; ∗∗P <0.01; by Student’s t-test). (B) Representative Immunofluorescent images of AUB-PrC cells {patient 4 with Grade Group 1 [Gleason Score 6(3 +3)] and patient 5 with Grade Group 3 [Gleason Score 7(4 +3)]; patients characteristics in Supplementary Table S1} stained with the prostate lineage epithelial markers CK8, CK5, and VIM. The nuclei were stained with anti-fade reagent Fluorogel II with DAPI. The images were acquired using the Zeiss LSM 710 laser scanning confocal microscope (Zeiss), and images were processed using the Carl Zeiss ZEN 2013 image software. Scale bar 200μm. [file Image_3.TIF]

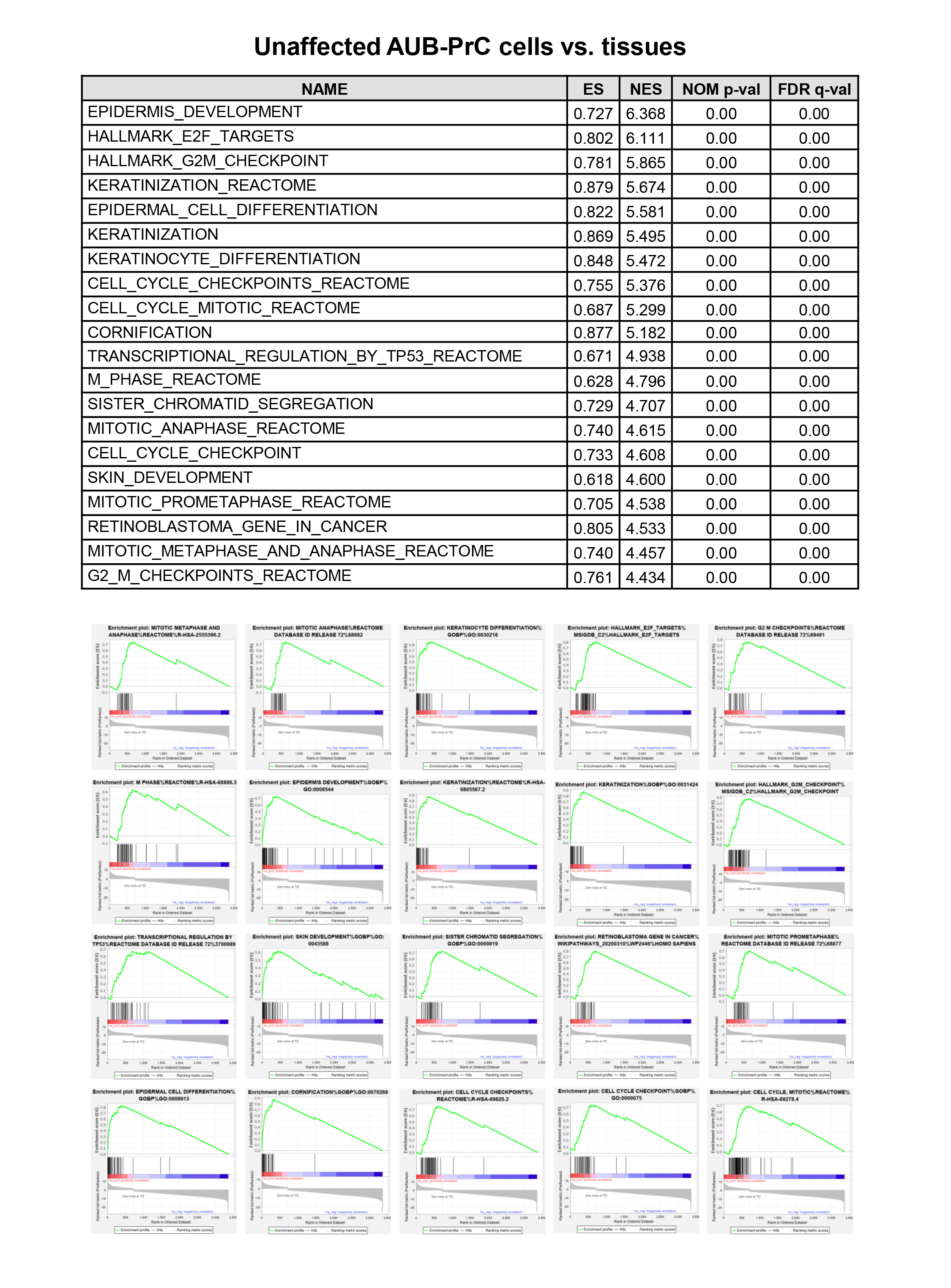

Supplement: Supplementary Figure 4 — Gene set enrichment analysis (GSEA) of the top 20 signaling pathways activated in AUB-PrC cells relative to their tissue counterparts among the unaffected samples. Comparison of data sets indicated unaffected AUB-PrC cells had enrichment of cell cycle pathways, E2F signaling, TP53 transcriptional regulation, Rb signaling, mitosis, and epithelial differentiation pathways. [file Image_4.TIF]

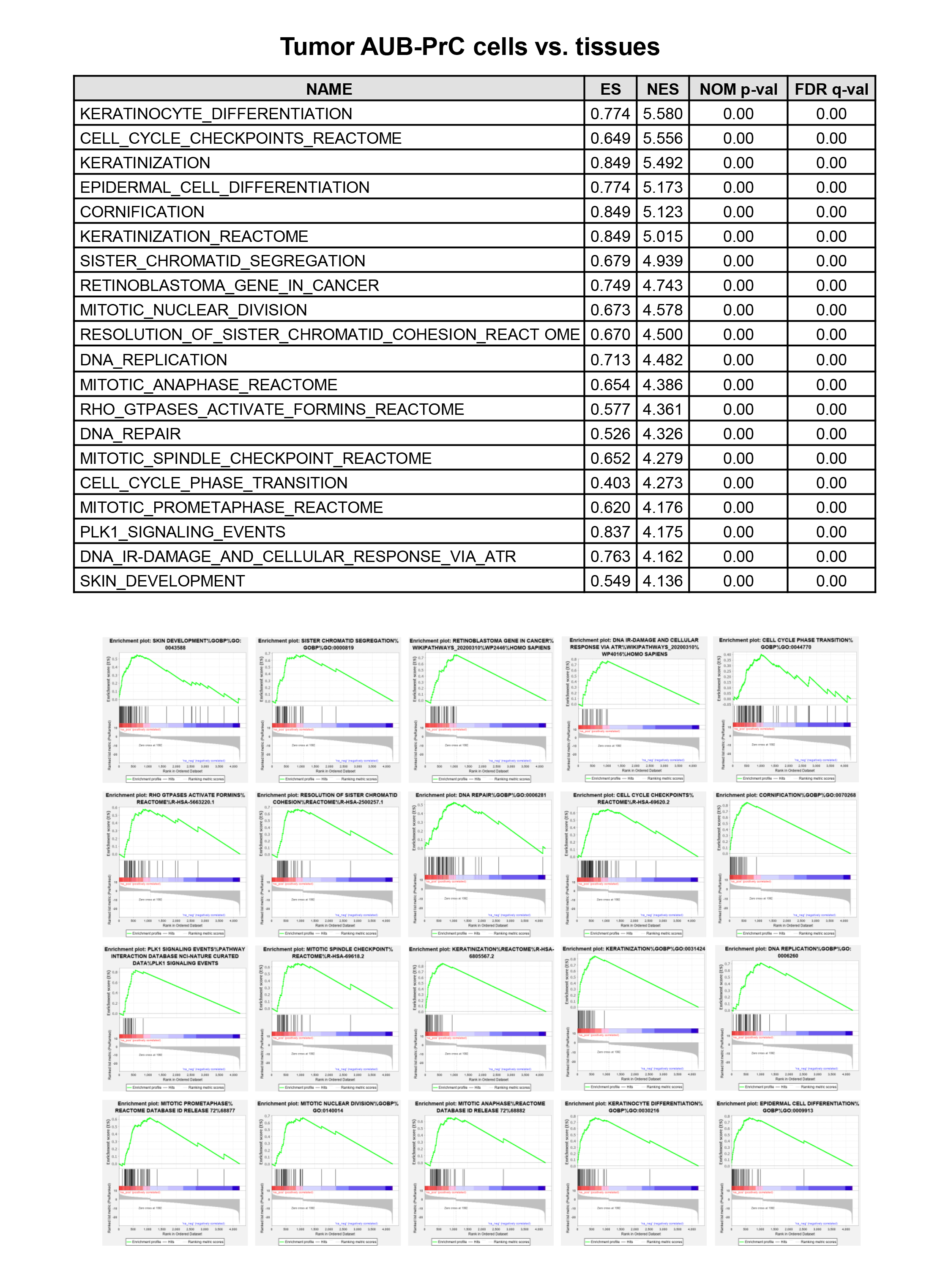

Supplement: Supplementary Figure 5 — Gene set enrichment analysis (GSEA) of the top 20 signaling pathways activated in AUB-PrC cells relative to their tissue counterparts among the tumor samples. Comparison of data sets indicated tumor AUB-PrC cells had enrichment of cell cycle pathways, PLK1 signaling, DNA irradiation damage and cellular response via ATR, and epithelial differentiation pathways. [file Image_5.TIF]
