# Supplementary material for: Epidermal Growth Factor Is Essential for the Maintenance of Novel Prostate Epithelial Cells Isolated From Patient-Derived Organoids
Source: Front Cell Dev Biol. 2020 Oct 29;8:571677. doi: 10.3389/fcell.2020.571677 (PMC7658326; doi:10.3389/fcell.2020.571677)
Supplement: Supplementary Table 1 — Patients’ clinical characteristics. [file Table_1.DOCX]

**Table S1. Patients’ clinical characteristics.**

| Patient # | Age (years) | PSA (ng/mL) | Gleason score | ISUP grade group | TNM Staging | Prostate size (in g) |
| --- | --- | --- | --- | --- | --- | --- |
| Patient 1 | 61 | 2.9 | 9(5+4) | Grade group 5 | T2a, N0, M0 | 80 |
| Patient 2 | 64 | 4 | 7(4+3) | Grade group 3 | T2b, N0, M0 | 60 |
| Patient 3 | 73 | 5.2 | 7(3+4) | Grade group 2 | T2b, N0, M0 | 40 |
| Patient 4 | 66 | 4.62 | 6(3+3) | Grade group 1 | T2b, N0, M0 | 35 |
| Patient 5 | 63 | 5.44 | 7(4+3) | Grade group 3 | T2c, N0, M0 | 65 |
| Patient 6 | 58 | - | 7(3+4) | Grade group 2 | T2c, N0, M1 | 55 |
| Patient 7 | 62 | - | 7(4+3) | Grade group 3 | - | 60 |

**Abbreviations**: ISUP: International Society of Urological Pathology
