# Supplementary material for: Epidermal Growth Factor Is Essential for the Maintenance of Novel Prostate Epithelial Cells Isolated From Patient-Derived Organoids
Source: Front Cell Dev Biol. 2020 Oct 29;8:571677. doi: 10.3389/fcell.2020.571677 (PMC7658326; doi:10.3389/fcell.2020.571677)
Supplement: Supplementary Table 2 — Overview of specific components and their respective concentrations added to prepare human prostate organoids culture medium. Adopted and modified from Drost et al. (2016). [file Table_2.DOCX]

**Table S2. Overview of specific components and their respective concentrations added to prepare human prostate organoids culture medium.** Adopted and modified from Drost *et al.* (Drost et al., 2016).

| Component | Stock concentration | Solvent | Final concentration |
| --- | --- | --- | --- |
| B27 | 50X | - | 1X |
| Nicotinamide | 1M | PBS | 10mM |
| NAC | 500 mM | PBS | 1.25 mM |
| EGF | 500 µg/mL* | PBS | 10 ng/mL* |
| A83 | 5 mM | DMSO | 500 nM |
| NOG | 100 µg/mL* | PBS + 0.1% BSA* | 50 ng/mL* |
| RSPO | 500 µg/mL* | PBS + 0.1% BSA* | 250 ng/mL* |
| DHT | 10 µM | Ethanol | 1 nM |
| FGF2 | 100 µg/mL* | PBS + 0.1% BSA* | 6 ng/mL* |
| FGF10 | 0.1 mg/mL | PBS + 0.1% BSA | 10 ng/mL |
| PGE2 | 10 mM | DMSO | 1 µM |
| SB | 10 mM | DMSO | 10 µM |
| Y-27632 | 10 mM | PBS + 0.1% BSA | 10 µM |

**Abbreviations: PBS:** phosphate buffered saline; **BSA:** bovine serum albumin; **DMSO:** dimethyl sulfoxide; **NAC:** N-acetylcysteine; **EGF:** epidermal growth factor; **A83:** A83-01, TGFβ kinase/activin receptor-like kinase (ALK 5) inhibitor; **NOG:** noggin; **RSPO:** R-spondin; **DHT:** dihydrotestosterone; **FGF2:** basic fibroblast growth factor (bFGF or FGF-β); **FGF10:** fibroblast growth factor 10; **PGE2:** prostaglandin E2; **SB:** SB202190, p38 MAP kinase inhibitor

* modifications applied to the protocol described by Drost et al. (Drost et al., 2016)

**References**

Drost, J., Karthaus, W.R., Gao, D., Driehuis, E., Sawyers, C.L., Chen, Y., et al. (2016). Organoid culture systems for prostate epithelial tissue and prostate cancer tissue. *Nat Protoc* 11(2)**,** 347-358. doi: 10.1038/nprot.2016.006.
