# Supplementary material for: Epidermal Growth Factor Is Essential for the Maintenance of Novel Prostate Epithelial Cells Isolated From Patient-Derived Organoids
Source: Front Cell Dev Biol. 2020 Oct 29;8:571677. doi: 10.3389/fcell.2020.571677 (PMC7658326; doi:10.3389/fcell.2020.571677)
Supplement: Supplementary Table 3 — Primer sequences and annealing temperatures for select human genes. [file Table_3.DOCX]

**Table S3. Primer sequences and annealing temperatures for select human genes.**

| **#** | **Human Gene** | **GenBank Accession #** | **Primer sequence** **(5’ – 3’)** | **Reference** |
| --- | --- | --- | --- | --- |
| **1** | ***AR*** | NM_001348061.1 | F- GGTGAGCAGAGTGCCCTATC | (Ahmad et al., 2017) |
|  |  |  | R- TTCCCTTCAGCGGCTCTTTT |  |
| **2** | ***CD44*** | NM_001001291.1 | F- TTTGCATTGCAGTCAACAGTC | (Jijiwa et al., 2011) |
|  |  |  | R- GTTACACCCCAATCTTCATGTCCAC |  |
| **3** | ***CDH1*** | NM_001317184.1 | F- TTCTGCTGCTCTTGCTGTTT | (Chen, 1993) |
|  |  |  | R- TGGCTCAAGTCAAAGTCCTG |  |
| **4** | ***CDH2*** | NM_001308176.1 | F- CCTGCTTATCCTTGTGCTGA | (Huang et al., 2016) |
|  |  |  | R- CCTGGTCTTCTTCTCCTCCA |  |
| **5** | ***CK8*** | NM_002273.3 | F- GCTGACCGACGAGATCAACT | (Li et al., 2012) |
|  |  |  | R- CCATGGACAGCACCACAGAT |  |
| **6** | ***CK18*** | NM_199187.1 | F- TCGCAAATACTGTGGACAATGC | (Sun et al., 2017) |
|  |  |  | R- GCAGTCGTGTGATATTGGTGT |  |
| **7** | ***GAPDH*** | NM_001256799.1 | F- GTCAGTGGTGGACCTGACCT | (Pasini et al., 2008) |
|  |  |  | R- TCGCTGTTGAAGTCAGAGGA |  |
| **8** | ***NKX3.1*** | NM_001256339.1 | F- GAGACGCTGGCAGAGACC | (Eide et al., 2013) |
|  |  |  | R- GCGCCTGAAGTGTTTTCAGA |  |
| **9** | ***P63*** | NM_001114980.1 | F- ACCTCCTCAGGGAGCTGTTA | (Rouleau et al., 2011) |
|  |  |  | R- ATACTGGGCATGGCTGTTCC |  |
| **10** | ***TWIST1*** | NM_000474.3 | F- CTCGGACAAGCTGAGCAAGA | (Chen et al., 2018) |
|  |  |  | R- GCTCTGGAGGACCTGGTAGA |  |
| **10** | ***VIM*** | NM_003380.4 | F- AGGTGGACCAGCTAACCAAC | (Jalaleddine et al., 2019) |
|  |  |  | R- TCTCCTCCTGCAATTTCTCC |  |

F, forward; R, reverse.

**References:**

Ahmad, Z., Xing, C., Panach, K., Kittler, R., McPhaul, M.J., and Wilson, J.D. (2017). Identification of the Underlying Androgen Receptor Defect in the Dallas Reifenstein Family. *Journal of the Endocrine Society* 1(7)**,** 836-842. doi: 10.1210/js.2017-00124.

Chen, S., Wang, W., Lin, G., and Zhong, S. 2018. MicroRNA-195 inhibits epithelial-mesenchymal transition via downregulating CDK4 in bladder cancer. *International journal of clinical and experimental pathology* [Online], 11(8). [Accessed 2018].

Chen, T.R. (1993). Chromosome identity of human prostate cancer cell lines, PC-3 and PPC-1. *Cytogenet Cell Genet* 62(2-3)**,** 183-184. doi: 10.1159/000133468.

Eide, T., Ramberg, H., Glackin, C., Tindall, D., and Taskén, K.A. 2013. TWIST1, A novel androgen-regulated gene, is a target for NKX3-1 in prostate cancer cells. *Cancer cell international* [Online], 13(1). [Accessed 2013].

Huang, M., Liu, T., Ma, P., Mitteer, R.A., Jr., Zhang, Z., Kim, H.J., et al. (2016). c-Met-mediated endothelial plasticity drives aberrant vascularization and chemoresistance in glioblastoma. *The Journal of clinical investigation* 126(5)**,** 1801-1814. doi: 10.1172/JCI84876.

Jalaleddine, N., El-Hajjar, L., Dakik, H., Shaito, A., Saliba, J., Safi, R., et al. (2019). Pannexin1 Is Associated with Enhanced Epithelial-To-Mesenchymal Transition in Human Patient Breast Cancer Tissues and in Breast Cancer Cell Lines. *Cancers* 11(12)**,** 1967. doi: 10.3390/cancers11121967.

Jijiwa, M., Demir, H., Gupta, S., Leung, C., Joshi, K., Orozco, N., et al. (2011). CD44v6 regulates growth of brain tumor stem cells partially through the AKT-mediated pathway. *PloS one* 6(9)**,** e24217-e24217. doi: 10.1371/journal.pone.0024217.

Li, M., Fu, X., Ma, G., Sun, X., Dong, X., Nagy, T., et al. (2012). Atbf1 regulates pubertal mammary gland development likely by inhibiting the pro-proliferative function of estrogen-ER signaling. *PloS one* 7(12)**,** e51283-e51283. doi: 10.1371/journal.pone.0051283.

Pasini, B., McWhinney, S.R., Bei, T., Matyakhina, L., Stergiopoulos, S., Muchow, M., et al. (2008). Clinical and molecular genetics of patients with the Carney-Stratakis syndrome and germline mutations of the genes coding for the succinate dehydrogenase subunits SDHB, SDHC, and SDHD. *Eur J Hum Genet* 16(1)**,** 79-88. doi: 10.1038/sj.ejhg.5201904.

Rouleau, M., Medawar, A., Hamon, L., Shivtiel, S., Wolchinsky, Z., Zhou, H., et al. (2011). TAp63 is important for cardiac differentiation of embryonic stem cells and heart development. *Stem Cells* 29(11)**,** 1672-1683. doi: 10.1002/stem.723.

Sun, Q., Deng, X.-M., Wang, Y.-L., Zhen, Y.-F., Li, F., Chen, R.-H., et al. (2017). Serum is an indispensable factor in the maintenance of the biological characteristics of sweat gland cells. *Molecular medicine reports* 16(3)**,** 2691-2699. doi: 10.3892/mmr.2017.6909.
