# Supplementary material for: Epidermal Growth Factor Is Essential for the Maintenance of Novel Prostate Epithelial Cells Isolated From Patient-Derived Organoids
Source: Front Cell Dev Biol. 2020 Oct 29;8:571677. doi: 10.3389/fcell.2020.571677 (PMC7658326; doi:10.3389/fcell.2020.571677)
Supplement: Supplementary Table 5 — List of all differentially expressed genes (DEGs) between AUB-PrC cells and their tissue counterparts in unaffected samples. [file Table_5.DOCX]

**Table S5. List of all differentially expressed genes (DEGs) between AUB-PrC cells and their tissue counterparts in unaffected samples.**

| **Ensemble ID** | **Gene symbol** | **Fold-change (log base 2)** | ***p*-adjusted** |
| --- | --- | --- | --- |
| ENSG00000214049 | *UCA1* | 11.19551465 | 0.001711584 |
| ENSG00000169174 | *PCSK9* | 9.912397312 | 0.000468432 |
| ENSG00000231131 | *LINC01468* | 9.6531045 | 0.002673741 |
| ENSG00000115008 | *IL1A* | 9.231417766 | 2.08E-05 |
| ENSG00000143556 | *S100A7* | 9.051672462 | 0.022847162 |
| ENSG00000196344 | *ADH7* | 8.882974492 | 8.16E-05 |
| ENSG00000170465 | *KRT6C* | 8.774740406 | 3.54E-07 |
| ENSG00000171564 | *FGB* | 8.757878724 | 0.005859494 |
| ENSG00000129455 | *KLK8* | 8.446253884 | 1.76E-05 |
| ENSG00000010438 | *PRSS3* | 8.407040968 | 3.24E-10 |
| ENSG00000205076 | *LGALS7* | 8.385043506 | 1.12E-09 |
| ENSG00000149948 | *HMGA2* | 8.358556656 | 3.29E-06 |
| ENSG00000109511 | *ANXA10* | 8.108834602 | 0.015350771 |
| ENSG00000197632 | *SERPINB2* | 8.036523028 | 0.000164949 |
| ENSG00000170454 | *KRT75* | 8.022313407 | 0.005093888 |
| ENSG00000185069 | *KRT76* | 8.00948977 | 2.24E-05 |
| ENSG00000198074 | *AKR1B10* | 7.934617409 | 2.04E-23 |
| ENSG00000137440 | *FGFBP1* | 7.911747708 | 2.58E-11 |
| ENSG00000123364 | *HOXC13* | 7.793068219 | 0.001983501 |
| ENSG00000121742 | *GJB6* | 7.727780007 | 5.74E-10 |
| ENSG00000238271 | *IFNWP19* | 7.725094347 | 3.83E-05 |
| ENSG00000185479 | *KRT6B* | 7.422718327 | 2.39E-06 |
| ENSG00000205420 | *KRT6A* | 7.404364083 | 1.50E-08 |
| ENSG00000153923 | *CLCA3P* | 7.323777511 | 0.007781577 |
| ENSG00000147509 | *RGS20* | 7.317714652 | 9.28E-05 |
| ENSG00000133710 | *SPINK5* | 7.206562242 | 3.25E-09 |
| ENSG00000229526 | *KRT16P4* | 7.121629816 | 1.63E-06 |
| ENSG00000134827 | *TCN1* | 7.112211788 | 2.42E-09 |
| ENSG00000057149 | *SERPINB3* | 7.07061798 | 8.87E-08 |
| ENSG00000249641 | *HOXC13-AS* | 7.041598121 | 0.002186919 |
| ENSG00000172782 | *FADS6* | 7.040545155 | 0.003105018 |
| ENSG00000213606 | *AKR1B10P1* | 7.034674271 | 0.006922142 |
| ENSG00000256969 | *RP11-320N7.2* | 6.971729423 | 0.00071252 |
| ENSG00000124882 | *EREG* | 6.958974599 | 4.41E-15 |
| ENSG00000203837 | *PNLIPRP3* | 6.935091311 | 0.045950748 |
| ENSG00000178934 | *LGALS7B* | 6.825130484 | 2.19E-09 |
| ENSG00000144834 | *TAGLN3* | 6.682733703 | 0.001436404 |
| ENSG00000226145 | *KRT16P6* | 6.682361473 | 2.11E-07 |
| ENSG00000138675 | *FGF5* | 6.653488637 | 0.00694366 |
| ENSG00000186007 | *LEMD1* | 6.639081231 | 1.46E-10 |
| ENSG00000167754 | *KLK5* | 6.550909178 | 2.41E-06 |
| ENSG00000134757 | *DSG3* | 6.54764449 | 1.52E-07 |
| ENSG00000206073 | *SERPINB4* | 6.52939674 | 0.010273101 |
| ENSG00000109321 | *AREG* | 6.506923876 | 4.25E-11 |
| ENSG00000148798 | *INA* | 6.505151336 | 1.62E-07 |
| ENSG00000115844 | *DLX2* | 6.495762233 | 0.033469171 |
| ENSG00000114638 | *UPK1B* | 6.493379772 | 1.16E-06 |
| ENSG00000253746 | *RP11-527N22.2* | 6.488239209 | 0.011137567 |
| ENSG00000188624 | *IGFL3* | 6.436869298 | 0.023398279 |
| ENSG00000275216 | *RP11-54H7.4* | 6.415836059 | 6.03E-08 |
| ENSG00000196754 | *S100A2* | 6.408836834 | 6.70E-08 |
| ENSG00000205325 | *AC005863.1* | 6.39897508 | 0.019380133 |
| ENSG00000179869 | *ABCA13* | 6.376464204 | 0.006361682 |
| ENSG00000169474 | *SPRR1A* | 6.258048644 | 0.00661096 |
| ENSG00000136155 | *SCEL* | 6.23196644 | 1.52E-06 |
| ENSG00000165474 | *GJB2* | 6.230543493 | 2.19E-07 |
| ENSG00000228951 | *RP11-336A10.4* | 6.226058666 | 0.016150763 |
| ENSG00000169035 | *KLK7* | 6.213434199 | 1.77E-09 |
| ENSG00000175874 | *CREG2* | 6.193541593 | 4.48E-05 |
| ENSG00000237594 | *AP000251.3* | 6.090880862 | 0.025390249 |
| ENSG00000125657 | *TNFSF9* | 6.024045375 | 1.58E-05 |
| ENSG00000101188 | *NTSR1* | 6.014135348 | 0.008003672 |
| ENSG00000169469 | *SPRR1B* | 5.985250245 | 0.000847598 |
| ENSG00000224984 | *RP11-524H19.2* | 5.980112492 | 0.025702989 |
| ENSG00000229647 | *AC007879.7* | 5.972087084 | 0.036280268 |
| ENSG00000230088 | *KRT16P5* | 5.956013587 | 9.31E-05 |
| ENSG00000268621 | *AC006262.5* | 5.903618798 | 0.00012723 |
| ENSG00000189334 | *S100A14* | 5.879671248 | 3.26E-08 |
| ENSG00000111981 | *ULBP1* | 5.775044516 | 0.01525199 |
| ENSG00000137975 | *CLCA2* | 5.76296322 | 3.19E-06 |
| ENSG00000169594 | *BNC1* | 5.749338924 | 3.38E-07 |
| ENSG00000128510 | *CPA4* | 5.702639616 | 0.000215691 |
| ENSG00000186847 | *KRT14* | 5.700102511 | 1.51E-09 |
| ENSG00000130829 | *DUSP9* | 5.665226873 | 0.006290342 |
| ENSG00000183780 | *SLC35F3* | 5.655842136 | 1.47E-05 |
| ENSG00000242147 | *RP13-463N16.6* | 5.651859321 | 0.015290641 |
| ENSG00000099194 | *SCD* | 5.526190433 | 4.39E-10 |
| ENSG00000265415 | *CTD-2510F5.4* | 5.427824393 | 0.02346345 |
| ENSG00000153294 | *ADGRF4* | 5.410307072 | 5.94E-05 |
| ENSG00000182585 | *EPGN* | 5.388150971 | 9.70E-05 |
| ENSG00000185668 | *POU3F1* | 5.366356315 | 1.89E-06 |
| ENSG00000121743 | *GJA3* | 5.302955359 | 0.000125507 |
| ENSG00000164929 | *BAALC* | 5.292838548 | 0.000155318 |
| ENSG00000139988 | *RDH12* | 5.274457214 | 1.83E-05 |
| ENSG00000186832 | *KRT16* | 5.220887033 | 1.78E-06 |
| ENSG00000223784 | *RP11-554I8.2* | 5.182126363 | 0.004614352 |
| ENSG00000198729 | *PPP1R14C* | 5.163465889 | 2.42E-09 |
| ENSG00000276335 | *MIR205* | 5.059381027 | 0.023389343 |
| ENSG00000159784 | *FAM131B* | 5.055950236 | 2.34E-14 |
| ENSG00000171889 | *MIR31HG* | 5.006158152 | 0.00015459 |
| ENSG00000140600 | *SH3GL3* | 4.996042945 | 1.32E-05 |
| ENSG00000206075 | *SERPINB5* | 4.988715544 | 1.27E-05 |
| ENSG00000137819 | *PAQR5* | 4.984955501 | 2.42E-09 |
| ENSG00000182040 | *USH1G* | 4.943630636 | 0.01948244 |
| ENSG00000100344 | *PNPLA3* | 4.942238835 | 0.022251825 |
| ENSG00000186212 | *SOWAHB* | 4.940380545 | 0.000452367 |
| ENSG00000178363 | *CALML3* | 4.920897447 | 0.00016612 |
| ENSG00000203722 | *RAET1G* | 4.907387119 | 6.89E-05 |
| ENSG00000186081 | *KRT5* | 4.812397494 | 0.000343431 |
| ENSG00000176153 | *GPX2* | 4.806725543 | 8.70E-05 |
| ENSG00000086696 | *HSD17B2* | 4.804883459 | 9.38E-05 |
| ENSG00000189001 | *SBSN* | 4.804254627 | 0.000246687 |
| ENSG00000073737 | *DHRS9* | 4.803395554 | 6.07E-05 |
| ENSG00000169607 | *CKAP2L* | 4.798144477 | 1.46E-07 |
| ENSG00000103044 | *HAS3* | 4.775969837 | 5.49E-07 |
| ENSG00000163331 | *DAPL1* | 4.759057227 | 2.00E-10 |
| ENSG00000265763 | *ZNF488* | 4.734973607 | 0.001804161 |
| ENSG00000087494 | *PTHLH* | 4.734139622 | 0.00286738 |
| ENSG00000125285 | *SOX21* | 4.720427098 | 8.74E-05 |
| ENSG00000167165 | *UGT1A6* | 4.679552217 | 0.014974234 |
| ENSG00000131015 | *ULBP2* | 4.665972782 | 0.006837532 |
| ENSG00000183729 | *NPBWR1* | 4.662761047 | 0.000140977 |
| ENSG00000121207 | *LRAT* | 4.660811839 | 9.81E-05 |
| ENSG00000164825 | *DEFB1* | 4.643731312 | 2.82E-08 |
| ENSG00000184995 | *IFNE* | 4.6203837 | 0.000232851 |
| ENSG00000183347 | *GBP6* | 4.608577216 | 5.34E-07 |
| ENSG00000178919 | *FOXE1* | 4.587446242 | 0.000326425 |
| ENSG00000151012 | *SLC7A11* | 4.565283223 | 5.79E-08 |
| ENSG00000143320 | *CRABP2* | 4.536078204 | 1.42E-07 |
| ENSG00000163207 | *IVL* | 4.52991964 | 0.000236693 |
| ENSG00000175592 | *FOSL1* | 4.515033316 | 7.55E-06 |
| ENSG00000172818 | *OVOL1* | 4.50460813 | 0.00375413 |
| ENSG00000069812 | *HES2* | 4.492840286 | 0.001927443 |
| ENSG00000051341 | *POLQ* | 4.474087287 | 8.00E-07 |
| ENSG00000165891 | *E2F7* | 4.424726553 | 6.31E-05 |
| ENSG00000175793 | *SFN* | 4.386681497 | 0.000328087 |
| ENSG00000123892 | *RAB38* | 4.384637337 | 1.07E-14 |
| ENSG00000135925 | *WNT10A* | 4.338184257 | 0.000904371 |
| ENSG00000065618 | *COL17A1* | 4.312616089 | 0.000785661 |
| ENSG00000149021 | *SCGB1A1* | 4.304689907 | 0.000779946 |
| ENSG00000279561 | *RP11-4L24.4* | 4.299702592 | 0.03500659 |
| ENSG00000132746 | *ALDH3B2* | 4.299097009 | 0.007687413 |
| ENSG00000110427 | *KIAA1549L* | 4.277930973 | 0.007226739 |
| ENSG00000156535 | *CD109* | 4.260598166 | 3.82E-08 |
| ENSG00000175065 | *DSG4* | 4.249127621 | 0.012343325 |
| ENSG00000174564 | *IL20RB* | 4.242058302 | 4.17E-05 |
| ENSG00000154764 | *WNT7A* | 4.228962851 | 0.003366876 |
| ENSG00000104892 | *KLC3* | 4.22478926 | 1.13E-06 |
| ENSG00000196542 | *SPTSSB* | 4.199605513 | 1.15E-06 |
| ENSG00000227300 | *KRT16P2* | 4.192997247 | 0.008451469 |
| ENSG00000188373 | *C10orf99* | 4.191798365 | 0.035470251 |
| ENSG00000158825 | *CDA* | 4.191211681 | 1.11E-08 |
| ENSG00000101255 | *TRIB3* | 4.177332688 | 0.000150966 |
| ENSG00000131019 | *ULBP3* | 4.155115272 | 0.025036946 |
| ENSG00000214856 | *KRT16P1* | 4.126987028 | 0.000790471 |
| ENSG00000158402 | *CDC25C* | 4.09566132 | 0.000402449 |
| ENSG00000164520 | *RAET1E* | 4.065915745 | 0.001354103 |
| ENSG00000245648 | *RP11-277P12.20* | 4.065309132 | 0.000296446 |
| ENSG00000118193 | *KIF14* | 4.057493236 | 1.50E-05 |
| ENSG00000103257 | *SLC7A5* | 4.049956285 | 0.000104413 |
| ENSG00000070731 | *ST6GALNAC2* | 4.037621651 | 2.95E-05 |
| ENSG00000169258 | *GPRIN1* | 4.034319826 | 6.27E-05 |
| ENSG00000151632 | *AKR1C2* | 4.011907328 | 6.30E-06 |
| ENSG00000253161 | *LINC01605* | 3.984667843 | 0.002167467 |
| ENSG00000135069 | *PSAT1* | 3.975977515 | 2.03E-06 |
| ENSG00000090889 | *KIF4A* | 3.973231225 | 1.14E-05 |
| ENSG00000100739 | *BDKRB1* | 3.968348861 | 0.009605836 |
| ENSG00000176678 | *FOXL1* | 3.9655615 | 0.00026578 |
| ENSG00000095587 | *TLL2* | 3.946950885 | 2.82E-05 |
| ENSG00000132518 | *GUCY2D* | 3.94648902 | 0.046375641 |
| ENSG00000133101 | *CCNA1* | 3.935066045 | 0.032736668 |
| ENSG00000023909 | *GCLM* | 3.933066016 | 3.05E-08 |
| ENSG00000100526 | *CDKN3* | 3.916514116 | 2.12E-05 |
| ENSG00000133135 | *RNF128* | 3.914057611 | 1.63E-05 |
| ENSG00000165304 | *MELK* | 3.890671297 | 1.05E-06 |
| ENSG00000272405 | *RP11-284F21.10* | 3.887878453 | 0.002023502 |
| ENSG00000197261 | *C6orf141* | 3.88257919 | 0.001518113 |
| ENSG00000148680 | *HTR7* | 3.878759016 | 4.15E-06 |
| ENSG00000140519 | *RHCG* | 3.860003942 | 0.030686932 |
| ENSG00000065328 | *MCM10* | 3.848682829 | 0.000527996 |
| ENSG00000168143 | *FAM83B* | 3.834166318 | 0.001043028 |
| ENSG00000073792 | *IGF2BP2* | 3.804931995 | 5.51E-07 |
| ENSG00000035499 | *DEPDC1B* | 3.803612528 | 1.73E-05 |
| ENSG00000130751 | *NPAS1* | 3.777799274 | 7.61E-05 |
| ENSG00000168032 | *ENTPD3* | 3.7736463 | 6.25E-08 |
| ENSG00000159166 | *LAD1* | 3.77089428 | 0.00938571 |
| ENSG00000184731 | *FAM110C* | 3.766482385 | 0.003945712 |
| ENSG00000172548 | *NIPAL4* | 3.749891852 | 2.78E-05 |
| ENSG00000094804 | *CDC6* | 3.733810914 | 3.59E-07 |
| ENSG00000110400 | *PVRL1* | 3.729640928 | 0.01878517 |
| ENSG00000227640 | *SOX21-AS1* | 3.720874631 | 0.003621349 |
| ENSG00000134762 | *DSC3* | 3.710353202 | 0.008503827 |
| ENSG00000140450 | *ARRDC4* | 3.709452226 | 7.28E-12 |
| ENSG00000107984 | *DKK1* | 3.703136071 | 0.006721506 |
| ENSG00000174371 | *EXO1* | 3.682183448 | 0.000135493 |
| ENSG00000169429 | *CXCL8* | 3.679799796 | 0.015353464 |
| ENSG00000024526 | *DEPDC1* | 3.651195292 | 0.000476581 |
| ENSG00000242265 | *PEG10* | 3.63240409 | 0.000163871 |
| ENSG00000152056 | *AP1S3* | 3.628927678 | 3.15E-05 |
| ENSG00000167656 | *LY6D* | 3.622419531 | 0.025884023 |
| ENSG00000121552 | *CSTA* | 3.613645658 | 3.69E-09 |
| ENSG00000141682 | *PMAIP1* | 3.60634318 | 0.000295293 |
| ENSG00000139318 | *DUSP6* | 3.606085715 | 0.000291007 |
| ENSG00000136689 | *IL1RN* | 3.602502939 | 0.00230954 |
| ENSG00000198088 | *NUP62CL* | 3.597217971 | 0.00294758 |
| ENSG00000115884 | *SDC1* | 3.596723795 | 0.003645962 |
| ENSG00000123689 | *G0S2* | 3.58527963 | 0.000219511 |
| ENSG00000168078 | *PBK* | 3.582365756 | 0.000135537 |
| ENSG00000145934 | *TENM2* | 3.574123801 | 0.009349613 |
| ENSG00000147689 | *FAM83A* | 3.569698472 | 0.019944224 |
| ENSG00000171345 | *KRT19* | 3.566836642 | 0.01803954 |
| ENSG00000127423 | *AUNIP* | 3.550311616 | 0.001682246 |
| ENSG00000129451 | *KLK10* | 3.535193156 | 0.001052536 |
| ENSG00000163689 | *C3orf67* | 3.521835155 | 0.004344737 |
| ENSG00000143126 | *CELSR2* | 3.520893149 | 0.014660636 |
| ENSG00000131747 | *TOP2A* | 3.512533143 | 1.21E-07 |
| ENSG00000108602 | *ALDH3A1* | 3.511188857 | 0.022670719 |
| ENSG00000088726 | *TMEM40* | 3.508648333 | 0.039675912 |
| ENSG00000189431 | *RASSF10* | 3.507560046 | 0.025494011 |
| ENSG00000011426 | *ANLN* | 3.50109344 | 6.05E-05 |
| ENSG00000089356 | *FXYD3* | 3.500291893 | 0.000205885 |
| ENSG00000136943 | *CTSV* | 3.489362014 | 0.001404925 |
| ENSG00000144452 | *ABCA12* | 3.488074052 | 0.007871683 |
| ENSG00000124343 | *XG* | 3.475914046 | 0.002810998 |
| ENSG00000173338 | *KCNK7* | 3.475786188 | 0.016411545 |
| ENSG00000138829 | *FBN2* | 3.474669543 | 0.001349605 |
| ENSG00000112742 | *TTK* | 3.466801642 | 1.50E-05 |
| ENSG00000137309 | *HMGA1* | 3.464599974 | 0.000389046 |
| ENSG00000171208 | *NETO2* | 3.454227116 | 0.00307368 |
| ENSG00000151725 | *CENPU* | 3.448468487 | 7.07E-06 |
| ENSG00000198691 | *ABCA4* | 3.445576843 | 0.007863596 |
| ENSG00000124466 | *LYPD3* | 3.436530473 | 0.00913635 |
| ENSG00000164930 | *FZD6* | 3.435573012 | 1.20E-06 |
| ENSG00000153714 | *LURAP1L* | 3.418894085 | 0.000130868 |
| ENSG00000066279 | *ASPM* | 3.406103824 | 1.98E-05 |
| ENSG00000101144 | *BMP7* | 3.402123241 | 0.007687413 |
| ENSG00000130164 | *LDLR* | 3.39647027 | 8.96E-10 |
| ENSG00000188910 | *GJB3* | 3.396037518 | 0.003639904 |
| ENSG00000109674 | *NEIL3* | 3.395534216 | 0.013187891 |
| ENSG00000129151 | *BBOX1* | 3.389621002 | 0.002763091 |
| ENSG00000189280 | *GJB5* | 3.388445194 | 0.008003672 |
| ENSG00000075618 | *FSCN1* | 3.384959442 | 0.000136485 |
| ENSG00000006118 | *TMEM132A* | 3.383024909 | 0.000924118 |
| ENSG00000118402 | *ELOVL4* | 3.37878785 | 0.000449155 |
| ENSG00000105376 | *ICAM5* | 3.378231868 | 0.000942602 |
| ENSG00000112984 | *KIF20A* | 3.371439101 | 1.68E-05 |
| ENSG00000104549 | *SQLE* | 3.365423394 | 2.42E-09 |
| ENSG00000205413 | *SAMD9* | 3.348849068 | 2.50E-07 |
| ENSG00000265190 | *ANXA8* | 3.347051711 | 0.029050315 |
| ENSG00000122641 | *INHBA* | 3.337434211 | 0.002004017 |
| ENSG00000134755 | *DSC2* | 3.328674217 | 0.000544142 |
| ENSG00000182010 | *RTKN2* | 3.328025732 | 0.000328087 |
| ENSG00000163220 | *S100A9* | 3.327242357 | 0.02121024 |
| ENSG00000109805 | *NCAPG* | 3.324463043 | 0.000512564 |
| ENSG00000186767 | *SPIN4* | 3.320528441 | 2.12E-05 |
| ENSG00000172893 | *DHCR7* | 3.318877212 | 6.06E-05 |
| ENSG00000227036 | *LINC00511* | 3.314640674 | 0.013180923 |
| ENSG00000112761 | *WISP3* | 3.308395424 | 0.014700541 |
| ENSG00000137807 | *KIF23* | 3.298784887 | 5.25E-05 |
| ENSG00000088325 | *TPX2* | 3.294519615 | 7.07E-07 |
| ENSG00000117724 | *CENPF* | 3.291672863 | 8.19E-05 |
| ENSG00000276850 | *CH17-360D5.2* | 3.274241845 | 0.032736668 |
| ENSG00000272398 | *CD24* | 3.270179583 | 4.00E-07 |
| ENSG00000058085 | *LAMC2* | 3.25751322 | 0.032750048 |
| ENSG00000101670 | *LIPG* | 3.254199369 | 0.014974234 |
| ENSG00000120756 | *PLS1* | 3.252066068 | 0.000619319 |
| ENSG00000266680 | *RP5-1148A21.3* | 3.24942255 | 0.035604955 |
| ENSG00000163235 | *TGFA* | 3.244057014 | 0.000885607 |
| ENSG00000136492 | *BRIP1* | 3.239340738 | 0.000648242 |
| ENSG00000196083 | *IL1RAP* | 3.233302901 | 0.000232343 |
| ENSG00000100003 | *SEC14L2* | 3.229084331 | 0.000407931 |
| ENSG00000029153 | *ARNTL2* | 3.22301809 | 0.001152744 |
| ENSG00000112559 | *MDFI* | 3.218019885 | 0.002287071 |
| ENSG00000197956 | *S100A6* | 3.208889009 | 0.000177076 |
| ENSG00000261116 | *RP3-523K23.2* | 3.196378755 | 0.000271048 |
| ENSG00000144063 | *MALL* | 3.173042789 | 0.001716917 |
| ENSG00000066248 | *NGEF* | 3.172296947 | 0.014208523 |
| ENSG00000281406 | *BLACAT1* | 3.170419343 | 0.035306599 |
| ENSG00000105991 | *HOXA1* | 3.170035835 | 0.002749898 |
| ENSG00000189057 | *FAM111B* | 3.16979819 | 0.000433051 |
| ENSG00000278843 | *MMP28* | 3.167236975 | 0.001912612 |
| ENSG00000100504 | *PYGL* | 3.162229173 | 5.71E-05 |
| ENSG00000167513 | *CDT1* | 3.156335303 | 0.000190618 |
| ENSG00000137812 | *CASC5* | 3.155302751 | 0.001453025 |
| ENSG00000140945 | *CDH13* | 3.153222633 | 0.002897018 |
| ENSG00000166803 | *KIAA0101* | 3.151225942 | 4.35E-05 |
| ENSG00000139629 | *GALNT6* | 3.144550437 | 0.001613592 |
| ENSG00000062282 | *DGAT2* | 3.138700759 | 0.001922523 |
| ENSG00000178947 | *SMIM10L2A* | 3.138389469 | 0.006382104 |
| ENSG00000046604 | *DSG2* | 3.13579553 | 0.001856534 |
| ENSG00000166845 | *C18orf54* | 3.122492625 | 0.001176701 |
| ENSG00000052802 | *MSMO1* | 3.121601299 | 6.84E-07 |
| ENSG00000198435 | *NRARP* | 3.106659013 | 0.027850019 |
| ENSG00000184363 | *PKP3* | 3.101609587 | 0.039561256 |
| ENSG00000164379 | *FOXQ1* | 3.100430858 | 0.045675863 |
| ENSG00000081277 | *PKP1* | 3.098778019 | 0.019708636 |
| ENSG00000139289 | *PHLDA1* | 3.096599784 | 6.05E-05 |
| ENSG00000164251 | *F2RL1* | 3.096250496 | 0.002034632 |
| ENSG00000138311 | *ZNF365* | 3.091976929 | 0.006562932 |
| ENSG00000185761 | *ADAMTSL5* | 3.081428537 | 0.000812146 |
| ENSG00000188643 | *S100A16* | 3.077812552 | 7.89E-08 |
| ENSG00000142627 | *EPHA2* | 3.076115193 | 0.002030395 |
| ENSG00000164687 | *FABP5* | 3.07190489 | 0.012094495 |
| ENSG00000127564 | *PKMYT1* | 3.061650013 | 0.017636722 |
| ENSG00000071539 | *TRIP13* | 3.058040113 | 0.001422976 |
| ENSG00000156475 | *PPP2R2B* | 3.052150941 | 0.025939552 |
| ENSG00000247081 | *BAALC-AS1* | 3.051895053 | 0.009436871 |
| ENSG00000163931 | *TKT* | 3.051001332 | 6.33E-05 |
| ENSG00000158292 | *GPR153* | 3.050110118 | 6.73E-05 |
| ENSG00000112378 | *PERP* | 3.048613374 | 8.97E-05 |
| ENSG00000096696 | *DSP* | 3.044860059 | 0.031584425 |
| ENSG00000092853 | *CLSPN* | 3.038287563 | 0.006426003 |
| ENSG00000117394 | *SLC2A1* | 3.032340501 | 1.99E-05 |
| ENSG00000158315 | *RHBDL2* | 3.029825283 | 0.017751489 |
| ENSG00000247095 | *MIR210HG* | 3.021840691 | 0.038599342 |
| ENSG00000171877 | *FRMD5* | 3.020644821 | 0.027162343 |
| ENSG00000134057 | *CCNB1* | 3.014201651 | 6.51E-05 |
| ENSG00000163032 | *VSNL1* | 2.998893192 | 0.006050561 |
| ENSG00000138160 | *KIF11* | 2.998604785 | 6.33E-06 |
| ENSG00000061337 | *LZTS1* | 2.990256047 | 0.01095501 |
| ENSG00000129194 | *SOX15* | 2.984969788 | 9.09E-05 |
| ENSG00000139734 | *DIAPH3* | 2.981912714 | 0.008288596 |
| ENSG00000001630 | *CYP51A1* | 2.974406168 | 0.004990508 |
| ENSG00000184661 | *CDCA2* | 2.974053049 | 0.001323951 |
| ENSG00000119514 | *GALNT12* | 2.969901347 | 6.72E-05 |
| ENSG00000113161 | *HMGCR* | 2.964214705 | 1.66E-07 |
| ENSG00000099219 | *ERMP1* | 2.951887573 | 0.002625057 |
| ENSG00000121621 | *KIF18A* | 2.951378143 | 0.013130674 |
| ENSG00000135378 | *PRRG4* | 2.948735467 | 0.005955937 |
| ENSG00000122861 | *PLAU* | 2.94312358 | 0.004470228 |
| ENSG00000231991 | *ANXA2P2* | 2.939774959 | 4.15E-06 |
| ENSG00000143061 | *IGSF3* | 2.928660268 | 0.007263555 |
| ENSG00000099960 | *SLC7A4* | 2.916025633 | 0.001982246 |
| ENSG00000112414 | *ADGRG6* | 2.901758784 | 0.003664334 |
| ENSG00000151136 | *BTBD11* | 2.899219591 | 0.003830239 |
| ENSG00000181458 | *TMEM45A* | 2.884495568 | 0.021236081 |
| ENSG00000102554 | *KLF5* | 2.866736743 | 0.015519216 |
| ENSG00000093009 | *CDC45* | 2.865264748 | 0.00624945 |
| ENSG00000164087 | *POC1A* | 2.86372799 | 0.005741251 |
| ENSG00000170006 | *TMEM154* | 2.853295396 | 0.001413445 |
| ENSG00000136108 | *CKAP2* | 2.850570173 | 6.69E-06 |
| ENSG00000186185 | *KIF18B* | 2.849731478 | 0.001973083 |
| ENSG00000136802 | *LRRC8A* | 2.840756121 | 0.000514999 |
| ENSG00000225526 | *MKRN2OS* | 2.83995066 | 0.040201712 |
| ENSG00000173801 | *JUP* | 2.839127815 | 0.010450687 |
| ENSG00000232040 | *ZBED9* | 2.838968487 | 0.019708636 |
| ENSG00000005884 | *ITGA3* | 2.834594277 | 0.010355571 |
| ENSG00000072571 | *HMMR* | 2.829226036 | 0.002231983 |
| ENSG00000125968 | *ID1* | 2.827189725 | 0.022652763 |
| ENSG00000125864 | *BFSP1* | 2.825936006 | 0.0014313 |
| ENSG00000164070 | *HSPA4L* | 2.822464073 | 0.00037077 |
| ENSG00000140950 | *TLDC1* | 2.820182493 | 6.44E-05 |
| ENSG00000068489 | *PRR11* | 2.809031149 | 0.004051608 |
| ENSG00000151715 | *TMEM45B* | 2.808248538 | 0.035548919 |
| ENSG00000186871 | *ERCC6L* | 2.804021674 | 0.005572022 |
| ENSG00000164611 | *PTTG1* | 2.798165086 | 0.004175067 |
| ENSG00000170312 | *CDK1* | 2.797529966 | 0.000106653 |
| ENSG00000117650 | *NEK2* | 2.792816348 | 0.001994344 |
| ENSG00000138271 | *GPR87* | 2.790290642 | 0.008046811 |
| ENSG00000182718 | *ANXA2* | 2.787614222 | 6.69E-06 |
| ENSG00000162981 | *FAM84A* | 2.7868182 | 0.049121131 |
| ENSG00000148926 | *ADM* | 2.783966354 | 0.000168416 |
| ENSG00000069849 | *ATP1B3* | 2.781450026 | 1.29E-05 |
| ENSG00000147883 | *CDKN2B* | 2.77831404 | 0.002161991 |
| ENSG00000115163 | *CENPA* | 2.777425592 | 0.033357988 |
| ENSG00000074410 | *CA12* | 2.764735158 | 0.00053645 |
| ENSG00000156970 | *BUB1B* | 2.759441667 | 0.003005728 |
| ENSG00000153044 | *CENPH* | 2.751591606 | 3.80E-05 |
| ENSG00000164045 | *CDC25A* | 2.749752384 | 0.000117737 |
| ENSG00000180739 | *S1PR5* | 2.743639258 | 0.027217461 |
| ENSG00000041353 | *RAB27B* | 2.736566854 | 0.011909954 |
| ENSG00000167900 | *TK1* | 2.728348272 | 0.001034694 |
| ENSG00000062038 | *CDH3* | 2.719611952 | 0.024953666 |
| ENSG00000138182 | *KIF20B* | 2.708902811 | 0.001348416 |
| ENSG00000163293 | *NIPAL1* | 2.695505357 | 0.041913288 |
| ENSG00000198554 | *WDHD1* | 2.689036355 | 0.000737986 |
| ENSG00000223478 | *RP11-545E17.3* | 2.677953727 | 0.001354103 |
| ENSG00000165480 | *SKA3* | 2.6764962 | 0.049534162 |
| ENSG00000163535 | *SGOL2* | 2.668636769 | 0.000981259 |
| ENSG00000230937 | *MIR205HG* | 2.66746401 | 0.030323904 |
| ENSG00000166106 | *ADAMTS15* | 2.664243907 | 0.005847025 |
| ENSG00000162783 | *IER5* | 2.663678764 | 8.79E-07 |
| ENSG00000158125 | *XDH* | 2.657953031 | 0.010443686 |
| ENSG00000115255 | *REEP6* | 2.65621352 | 0.026560505 |
| ENSG00000104147 | *OIP5* | 2.654924034 | 0.045501388 |
| ENSG00000171320 | *ESCO2* | 2.64380645 | 0.018085273 |
| ENSG00000198142 | *SOWAHC* | 2.642835992 | 9.72E-05 |
| ENSG00000140284 | *SLC27A2* | 2.63169557 | 0.010569391 |
| ENSG00000075275 | *CELSR1* | 2.629564677 | 0.047278223 |
| ENSG00000124762 | *CDKN1A* | 2.622985822 | 6.13E-07 |
| ENSG00000138180 | *CEP55* | 2.621040584 | 0.004730385 |
| ENSG00000196139 | *AKR1C3* | 2.619117578 | 0.036864712 |
| ENSG00000164465 | *DCBLD1* | 2.617155972 | 0.000838383 |
| ENSG00000171848 | *RRM2* | 2.617048789 | 0.000516887 |
| ENSG00000002587 | *HS3ST1* | 2.613922248 | 0.00108543 |
| ENSG00000154102 | *C16orf74* | 2.610100793 | 0.000310384 |
| ENSG00000102471 | *NDFIP2* | 2.610065783 | 0.000448231 |
| ENSG00000104413 | *ESRP1* | 2.606862832 | 0.045141991 |
| ENSG00000131188 | *PRR7* | 2.604034202 | 0.032397087 |
| ENSG00000128965 | *CHAC1* | 2.603534555 | 0.038559348 |
| ENSG00000120149 | *MSX2* | 2.598391725 | 0.025912566 |
| ENSG00000121152 | *NCAPH* | 2.596625012 | 0.011429867 |
| ENSG00000173156 | *RHOD* | 2.592072372 | 3.39E-05 |
| ENSG00000276043 | *UHRF1* | 2.59081904 | 0.002025044 |
| ENSG00000163624 | *CDS1* | 2.589494157 | 0.012031193 |
| ENSG00000237512 | *UNC5B-AS1* | 2.588478311 | 0.035085876 |
| ENSG00000114346 | *ECT2* | 2.587122211 | 0.001354103 |
| ENSG00000214796 | *RP11-480I12.5* | 2.582705135 | 0.033642072 |
| ENSG00000259153 | *RP6-65G23.3* | 2.581435707 | 0.04243493 |
| ENSG00000175305 | *CCNE2* | 2.578781065 | 0.036149444 |
| ENSG00000132698 | *RAB25* | 2.577421263 | 0.017492848 |
| ENSG00000105011 | *ASF1B* | 2.574631728 | 0.004102099 |
| ENSG00000121690 | *DEPDC7* | 2.572970445 | 0.012203738 |
| ENSG00000006327 | *TNFRSF12A* | 2.556824302 | 0.019053109 |
| ENSG00000198901 | *PRC1* | 2.556316478 | 0.000229555 |
| ENSG00000101003 | *GINS1* | 2.552317411 | 0.001305715 |
| ENSG00000148773 | *MKI67* | 2.537557708 | 0.016017702 |
| ENSG00000169679 | *BUB1* | 2.533902996 | 0.013081469 |
| ENSG00000198018 | *ENTPD7* | 2.533352901 | 0.043297628 |
| ENSG00000116717 | *GADD45A* | 2.531756729 | 0.002430005 |
| ENSG00000091651 | *ORC6* | 2.528792807 | 0.001688272 |
| ENSG00000142731 | *PLK4* | 2.528781789 | 0.005904493 |
| ENSG00000126787 | *DLGAP5* | 2.52485206 | 0.020593468 |
| ENSG00000197747 | *S100A10* | 2.522582796 | 0.002576058 |
| ENSG00000163191 | *S100A11* | 2.521205758 | 3.73E-05 |
| ENSG00000164171 | *ITGA2* | 2.510692192 | 0.020555382 |
| ENSG00000176597 | *B3GNT5* | 2.501428851 | 0.007801027 |
| ENSG00000146592 | *CREB5* | 2.489682239 | 0.025341017 |
| ENSG00000206159 | *GYG2P1* | 2.485533307 | 0.020968536 |
| ENSG00000060558 | *GNA15* | 2.484878894 | 0.01049503 |
| ENSG00000169247 | *SH3TC2* | 2.477310865 | 0.029802343 |
| ENSG00000276600 | *RAB7B* | 2.474598027 | 0.021232113 |
| ENSG00000138778 | *CENPE* | 2.472819218 | 0.011129593 |
| ENSG00000137563 | *GGH* | 2.470900651 | 0.000240377 |
| ENSG00000129195 | *FAM64A* | 2.466994129 | 0.021595376 |
| ENSG00000087586 | *AURKA* | 2.46224955 | 0.00142097 |
| ENSG00000152413 | *HOMER1* | 2.452762625 | 0.014355976 |
| ENSG00000156463 | *SH3RF2* | 2.447456155 | 0.032513257 |
| ENSG00000137804 | *NUSAP1* | 2.445864523 | 0.000535793 |
| ENSG00000116133 | *DHCR24* | 2.440410154 | 0.046271968 |
| ENSG00000091409 | *ITGA6* | 2.439339795 | 0.004714245 |
| ENSG00000112297 | *AIM1* | 2.438897029 | 0.035022646 |
| ENSG00000135046 | *ANXA1* | 2.434203553 | 9.99E-05 |
| ENSG00000123473 | *STIL* | 2.430819047 | 0.012432518 |
| ENSG00000063660 | *GPC1* | 2.430615264 | 0.028623497 |
| ENSG00000253368 | *TRNP1* | 2.427152663 | 0.000148786 |
| ENSG00000104738 | *MCM4* | 2.423250224 | 0.000653892 |
| ENSG00000040608 | *RTN4R* | 2.423180353 | 0.020381849 |
| ENSG00000228288 | *PCAT6* | 2.421465344 | 0.009300804 |
| ENSG00000143590 | *EFNA3* | 2.420826315 | 0.030603055 |
| ENSG00000089685 | *BIRC5* | 2.417956716 | 0.009872583 |
| ENSG00000122966 | *CIT* | 2.415123401 | 0.00838449 |
| ENSG00000123485 | *HJURP* | 2.414106253 | 0.012737753 |
| ENSG00000181019 | *NQO1* | 2.41174273 | 0.026780395 |
| ENSG00000075218 | *GTSE1* | 2.410011426 | 0.020248839 |
| ENSG00000182472 | *CAPN12* | 2.404533016 | 0.017051299 |
| ENSG00000186340 | *THBS2* | 2.396874055 | 0.034124482 |
| ENSG00000069011 | *PITX1* | 2.39638963 | 0.00053283 |
| ENSG00000161800 | *RACGAP1* | 2.394299923 | 0.004282389 |
| ENSG00000131187 | *F12* | 2.3892135 | 0.043366655 |
| ENSG00000139438 | *FAM222A* | 2.385307842 | 0.044715695 |
| ENSG00000280527 | *AL031587.1* | 2.383771682 | 0.022670719 |
| ENSG00000105427 | *CNFN* | 2.380279108 | 0.010559664 |
| ENSG00000139618 | *BRCA2* | 2.379864765 | 0.025884023 |
| ENSG00000175063 | *UBE2C* | 2.377236887 | 0.027548212 |
| ENSG00000100994 | *PYGB* | 2.369447405 | 0.000975845 |
| ENSG00000151090 | *THRB* | 2.36898137 | 0.003560614 |
| ENSG00000129474 | *AJUBA* | 2.367746006 | 0.022579638 |
| ENSG00000092621 | *PHGDH* | 2.365747963 | 0.000402449 |
| ENSG00000103485 | *QPRT* | 2.359289854 | 0.002690685 |
| ENSG00000099139 | *PCSK5* | 2.352624625 | 0.011565509 |
| ENSG00000145632 | *PLK2* | 2.351215141 | 0.013928302 |
| ENSG00000187210 | *GCNT1* | 2.341745222 | 0.014328411 |
| ENSG00000134215 | *VAV3* | 2.33528003 | 0.032083794 |
| ENSG00000128641 | *MYO1B* | 2.334067797 | 0.00303129 |
| ENSG00000185033 | *SEMA4B* | 2.33266689 | 0.038477022 |
| ENSG00000234546 | *RP3-510D11.2* | 2.330474467 | 0.031536196 |
| ENSG00000051596 | *THOC3* | 2.319815821 | 0.002249269 |
| ENSG00000189159 | *HN1* | 2.319701685 | 0.001804299 |
| ENSG00000079691 | *LRRC16A* | 2.319114883 | 5.37E-05 |
| ENSG00000145247 | *OCIAD2* | 2.313809412 | 0.003005728 |
| ENSG00000051180 | *RAD51* | 2.312730695 | 0.044626815 |
| ENSG00000123219 | *CENPK* | 2.312114419 | 0.010941796 |
| ENSG00000110921 | *MVK* | 2.299937496 | 0.000483044 |
| ENSG00000117399 | *CDC20* | 2.299092686 | 0.019708636 |
| ENSG00000161714 | *PLCD3* | 2.298396324 | 0.015977362 |
| ENSG00000226287 | *TMEM191A* | 2.290672619 | 0.031530152 |
| ENSG00000074181 | *NOTCH3* | 2.284606209 | 0.030293524 |
| ENSG00000164109 | *MAD2L1* | 2.277778228 | 0.003370168 |
| ENSG00000168398 | *BDKRB2* | 2.277556965 | 0.012410967 |
| ENSG00000026508 | *CD44* | 2.273586399 | 0.002534849 |
| ENSG00000278318 | *ZNF229* | 2.269903807 | 0.037196568 |
| ENSG00000097021 | *ACOT7* | 2.258014531 | 0.0018253 |
| ENSG00000170537 | *TMC7* | 2.258013263 | 0.012809569 |
| ENSG00000090776 | *EFNB1* | 2.248169742 | 0.000641949 |
| ENSG00000140525 | *FANCI* | 2.243748202 | 0.002795457 |
| ENSG00000164086 | *DUSP7* | 2.240074563 | 0.000595261 |
| ENSG00000120256 | *LRP11* | 2.227601279 | 0.003370168 |
| ENSG00000166401 | *SERPINB8* | 2.217997768 | 0.007560818 |
| ENSG00000184564 | *SLITRK6* | 2.217859315 | 0.00209421 |
| ENSG00000001084 | *GCLC* | 2.21732657 | 0.009213269 |
| ENSG00000107438 | *PDLIM1* | 2.213853903 | 0.00139253 |
| ENSG00000149554 | *CHEK1* | 2.211424722 | 0.003842131 |
| ENSG00000172292 | *CERS6* | 2.202325298 | 0.012349422 |
| ENSG00000146670 | *CDCA5* | 2.201053563 | 0.021552447 |
| ENSG00000135540 | *NHSL1* | 2.192067786 | 0.027512303 |
| ENSG00000105699 | *LSR* | 2.190902425 | 0.040036241 |
| ENSG00000119979 | *FAM45A* | 2.188902783 | 0.00027208 |
| ENSG00000147044 | *CASK* | 2.188489821 | 0.000233524 |
| ENSG00000149212 | *SESN3* | 2.187986526 | 0.003313796 |
| ENSG00000170425 | *ADORA2B* | 2.180262958 | 0.009409398 |
| ENSG00000128346 | *C22orf23* | 2.17892862 | 0.025596048 |
| ENSG00000057019 | *DCBLD2* | 2.178902337 | 0.00286738 |
| ENSG00000233901 | *LINC01503* | 2.17849231 | 0.006615031 |
| ENSG00000156453 | *PCDH1* | 2.176717777 | 0.049550417 |
| ENSG00000010278 | *CD9* | 2.176120437 | 5.32E-05 |
| ENSG00000155090 | *KLF10* | 2.175764391 | 6.46E-05 |
| ENSG00000165650 | *PDZD8* | 2.168522748 | 0.003151557 |
| ENSG00000100558 | *PLEK2* | 2.162903622 | 0.034626449 |
| ENSG00000166471 | *TMEM41B* | 2.161824892 | 0.002202706 |
| ENSG00000012048 | *BRCA1* | 2.160369486 | 0.019381892 |
| ENSG00000143476 | *DTL* | 2.158817785 | 0.038240572 |
| ENSG00000173848 | *NET1* | 2.142237678 | 0.004751679 |
| ENSG00000196743 | *GM2A* | 2.138297332 | 7.92E-05 |
| ENSG00000142657 | *PGD* | 2.135965905 | 0.000932906 |
| ENSG00000157456 | *CCNB2* | 2.132482375 | 0.037178607 |
| ENSG00000181467 | *RAP2B* | 2.12445004 | 0.001850851 |
| ENSG00000150054 | *MPP7* | 2.124391632 | 0.013395734 |
| ENSG00000174307 | *PHLDA3* | 2.120086417 | 0.001319988 |
| ENSG00000111206 | *FOXM1* | 2.116734481 | 0.014783392 |
| ENSG00000069956 | *MAPK6* | 2.115493363 | 0.007603466 |
| ENSG00000122952 | *ZWINT* | 2.109505496 | 0.004462064 |
| ENSG00000050405 | *LIMA1* | 2.107291937 | 0.002091592 |
| ENSG00000159228 | *CBR1* | 2.104509944 | 0.00093451 |
| ENSG00000091317 | *CMTM6* | 2.100498592 | 0.000204214 |
| ENSG00000134294 | *SLC38A2* | 2.096391936 | 0.006369535 |
| ENSG00000173218 | *VANGL1* | 2.094372756 | 0.013887562 |
| ENSG00000159182 | *PRAC1* | 2.092906971 | 0.004778721 |
| ENSG00000116661 | *FBXO2* | 2.082408493 | 0.017905349 |
| ENSG00000196517 | *SLC6A9* | 2.078114891 | 0.037572948 |
| ENSG00000198911 | *SREBF2* | 2.070549066 | 0.014355976 |
| ENSG00000170779 | *CDCA4* | 2.068426493 | 0.006821565 |
| ENSG00000173207 | *CKS1B* | 2.062203008 | 0.017201041 |
| ENSG00000131153 | *GINS2* | 2.059642888 | 0.004284478 |
| ENSG00000164543 | *STK17A* | 2.057488647 | 0.010941796 |
| ENSG00000174939 | *ASPHD1* | 2.055178539 | 0.034308403 |
| ENSG00000168785 | *TSPAN5* | 2.054779341 | 0.023901657 |
| ENSG00000142867 | *BCL10* | 2.044048367 | 0.004403168 |
| ENSG00000140022 | *STON2* | 2.039906106 | 0.006293448 |
| ENSG00000167553 | *TUBA1C* | 2.032520027 | 0.044945755 |
| ENSG00000198589 | *LRBA* | 2.030575217 | 0.028344969 |
| ENSG00000178999 | *AURKB* | 2.025631986 | 0.042334023 |
| ENSG00000126778 | *SIX1* | 2.024254036 | 0.00386123 |
| ENSG00000101773 | *RBBP8* | 2.018153161 | 0.001100147 |
| ENSG00000175984 | *DENND2C* | 2.015083806 | 0.02827262 |
| ENSG00000133818 | *RRAS2* | 2.013555018 | 0.012501304 |
| ENSG00000112655 | *PTK7* | 2.00792777 | 0.03136008 |
| ENSG00000181588 | *MEX3D* | 1.999967743 | 0.027136686 |
| ENSG00000149485 | *FADS1* | 1.999108624 | 0.024224113 |
| ENSG00000215458 | *AATBC* | 1.997921876 | 0.048230701 |
| ENSG00000141441 | *GAREM* | 1.987964463 | 0.031835514 |
| ENSG00000167642 | *SPINT2* | 1.987697456 | 0.034331997 |
| ENSG00000105655 | *ISYNA1* | 1.983367142 | 0.032435408 |
| ENSG00000108984 | *MAP2K6* | 1.972179643 | 0.012769159 |
| ENSG00000213190 | *MLLT11* | 1.969563321 | 0.016499391 |
| ENSG00000198087 | *CD2AP* | 1.965898207 | 0.006369535 |
| ENSG00000164951 | *PDP1* | 1.965590685 | 0.003605158 |
| ENSG00000117632 | *STMN1* | 1.961774666 | 0.029286064 |
| ENSG00000118242 | *MREG* | 1.95346286 | 0.0220053 |
| ENSG00000150782 | *IL18* | 1.949504878 | 0.044701712 |
| ENSG00000178184 | *PARD6G* | 1.946881347 | 0.005937695 |
| ENSG00000157193 | *LRP8* | 1.946010723 | 0.024845097 |
| ENSG00000157978 | *LDLRAP1* | 1.943198235 | 0.034740509 |
| ENSG00000091136 | *LAMB1* | 1.943065609 | 0.024255822 |
| ENSG00000124145 | *SDC4* | 1.941658875 | 0.001897938 |
| ENSG00000146733 | *PSPH* | 1.938058996 | 0.0067241 |
| ENSG00000128311 | *TST* | 1.937206048 | 0.004502415 |
| ENSG00000182134 | *TDRKH* | 1.934166473 | 0.03687812 |
| ENSG00000167103 | *PIP5KL1* | 1.93167572 | 0.044804786 |
| ENSG00000198363 | *ASPH* | 1.925378041 | 0.00286738 |
| ENSG00000198743 | *SLC5A3* | 1.924891649 | 0.034438486 |
| ENSG00000175906 | *ARL4D* | 1.922521281 | 0.009705034 |
| ENSG00000128578 | *STRIP2* | 1.917223739 | 0.023637675 |
| ENSG00000124766 | *SOX4* | 1.908346205 | 0.048709541 |
| ENSG00000135823 | *STX6* | 1.901723249 | 0.00134909 |
| ENSG00000213853 | *EMP2* | 1.900068652 | 0.004926875 |
| ENSG00000105755 | *ETHE1* | 1.897638837 | 0.005977734 |
| ENSG00000136824 | *SMC2* | 1.891587549 | 0.012607324 |
| ENSG00000135750 | *KCNK1* | 1.885969979 | 0.02003561 |
| ENSG00000119782 | *FKBP1B* | 1.885891343 | 0.03442529 |
| ENSG00000156110 | *ADK* | 1.885880907 | 0.002957326 |
| ENSG00000186493 | *C5orf38* | 1.884628116 | 0.040087055 |
| ENSG00000104723 | *TUSC3* | 1.881128971 | 0.003917394 |
| ENSG00000089682 | *RBM41* | 1.879833881 | 0.031906373 |
| ENSG00000277443 | *MARCKS* | 1.878738018 | 0.028310966 |
| ENSG00000106484 | *MEST* | 1.875155708 | 0.04035722 |
| ENSG00000167325 | *RRM1* | 1.871275174 | 0.009634336 |
| ENSG00000145545 | *SRD5A1* | 1.870341664 | 0.010089948 |
| ENSG00000132002 | *DNAJB1* | 1.858336719 | 0.004027392 |
| ENSG00000123983 | *ACSL3* | 1.850508883 | 0.01087719 |
| ENSG00000162627 | *SNX7* | 1.850076432 | 0.038497737 |
| ENSG00000168916 | *ZNF608* | 1.849243457 | 0.043946624 |
| ENSG00000144354 | *CDCA7* | 1.848656101 | 0.006721506 |
| ENSG00000059728 | *MXD1* | 1.847768116 | 0.03200641 |
| ENSG00000170522 | *ELOVL6* | 1.841559383 | 0.027640777 |
| ENSG00000040275 | *SPDL1* | 1.837332123 | 0.046102916 |
| ENSG00000120539 | *MASTL* | 1.835600141 | 0.025499281 |
| ENSG00000134824 | *FADS2* | 1.827521797 | 0.017335179 |
| ENSG00000133119 | *RFC3* | 1.82696103 | 0.04718686 |
| ENSG00000170899 | *GSTA4* | 1.825573009 | 0.03125815 |
| ENSG00000257084 | *U47924.27* | 1.805976743 | 0.047159724 |
| ENSG00000079459 | *FDFT1* | 1.800510326 | 0.002567399 |
| ENSG00000161243 | *FBXO27* | 1.790692747 | 0.014587476 |
| ENSG00000176105 | *YES1* | 1.790177291 | 0.012146483 |
| ENSG00000163249 | *CCNYL1* | 1.789008742 | 0.008558248 |
| ENSG00000106853 | *PTGR1* | 1.786347727 | 0.024455461 |
| ENSG00000143367 | *TUFT1* | 1.784764328 | 0.012917385 |
| ENSG00000011638 | *TMEM159* | 1.78078914 | 0.003431554 |
| ENSG00000184117 | *NIPSNAP1* | 1.776938452 | 0.009046375 |
| ENSG00000161249 | *DMKN* | 1.773720178 | 0.007086614 |
| ENSG00000106537 | *TSPAN13* | 1.771634955 | 0.049354517 |
| ENSG00000135698 | *MPHOSPH6* | 1.769839584 | 0.005063485 |
| ENSG00000144136 | *SLC20A1* | 1.768633326 | 0.01253949 |
| ENSG00000165097 | *KDM1B* | 1.767766175 | 0.01730421 |
| ENSG00000101166 | *SLMO2* | 1.765039023 | 0.015349709 |
| ENSG00000166949 | *SMAD3* | 1.759405509 | 0.023689191 |
| ENSG00000066084 | *DIP2B* | 1.757232298 | 0.018207996 |
| ENSG00000126777 | *KTN1* | 1.756227335 | 0.018701062 |
| ENSG00000171159 | *C9orf16* | 1.751551579 | 0.003276041 |
| ENSG00000148572 | *NRBF2* | 1.751417996 | 0.049875865 |
| ENSG00000151694 | *ADAM17* | 1.747725724 | 0.029989047 |
| ENSG00000122378 | *FAM213A* | 1.747654764 | 0.02126365 |
| ENSG00000035115 | *SH3YL1* | 1.746007177 | 0.025967127 |
| ENSG00000103021 | *CCDC113* | 1.743348245 | 0.03613231 |
| ENSG00000128487 | *SPECC1* | 1.742116534 | 0.024073775 |
| ENSG00000108582 | *CPD* | 1.734087944 | 0.028796541 |
| ENSG00000111247 | *RAD51AP1* | 1.732949438 | 0.043534138 |
| ENSG00000146918 | *NCAPG2* | 1.727976101 | 0.007245596 |
| ENSG00000182197 | *EXT1* | 1.723483946 | 0.01107052 |
| ENSG00000129235 | *TXNDC17* | 1.717173942 | 0.015237697 |
| ENSG00000106546 | *AHR* | 1.711538782 | 0.012468647 |
| ENSG00000180198 | *RCC1* | 1.70624961 | 0.024507965 |
| ENSG00000153879 | *CEBPG* | 1.703936424 | 0.024730053 |
| ENSG00000113810 | *SMC4* | 1.700557039 | 0.018790768 |
| ENSG00000072682 | *P4HA2* | 1.693208312 | 0.021005257 |
| ENSG00000162909 | *CAPN2* | 1.69201721 | 0.00310255 |
| ENSG00000109881 | *CCDC34* | 1.689745524 | 0.021005257 |
| ENSG00000101935 | *AMMECR1* | 1.689490011 | 0.02473933 |
| ENSG00000159259 | *CHAF1B* | 1.689139721 | 0.032338674 |
| ENSG00000127870 | *RNF6* | 1.684595822 | 0.014913397 |
| ENSG00000146242 | *TPBG* | 1.683978752 | 0.009264048 |
| ENSG00000012232 | *EXTL3* | 1.680116297 | 0.021005257 |
| ENSG00000149809 | *TM7SF2* | 1.665628498 | 0.044428817 |
| ENSG00000106829 | *TLE4* | 1.663793061 | 0.01353433 |
| ENSG00000178585 | *CTNNBIP1* | 1.649759842 | 0.016509748 |
| ENSG00000185963 | *BICD2* | 1.643980636 | 0.030161532 |
| ENSG00000196937 | *FAM3C* | 1.635455039 | 0.020604211 |
| ENSG00000132646 | *PCNA* | 1.631319563 | 0.012286924 |
| ENSG00000092820 | *EZR* | 1.629409599 | 0.006325746 |
| ENSG00000137269 | *LRRC1* | 1.626025222 | 0.020308165 |
| ENSG00000138413 | *IDH1* | 1.624688011 | 0.016282893 |
| ENSG00000100300 | *TSPO* | 1.609002184 | 0.007641925 |
| ENSG00000189060 | *H1F0* | 1.605720749 | 0.031643312 |
| ENSG00000170873 | *MTSS1* | 1.605622199 | 0.010975795 |
| ENSG00000071054 | *MAP4K4* | 1.603588739 | 0.016418154 |
| ENSG00000102699 | *PARP4* | 1.594575121 | 0.023395154 |
| ENSG00000004961 | *HCCS* | 1.591628653 | 0.042472578 |
| ENSG00000170017 | *ALCAM* | 1.588214916 | 0.025750171 |
| ENSG00000114738 | *MAPKAPK3* | 1.588087981 | 0.009300804 |
| ENSG00000080819 | *CPOX* | 1.585838606 | 0.041473191 |
| ENSG00000102572 | *STK24* | 1.57005298 | 0.015686141 |
| ENSG00000241685 | *ARPC1A* | 1.565536694 | 0.020619192 |
| ENSG00000169241 | *SLC50A1* | 1.565099172 | 0.033573512 |
| ENSG00000144036 | *EXOC6B* | 1.562997143 | 0.022431232 |
| ENSG00000185909 | *KLHDC8B* | 1.561884605 | 0.046007549 |
| ENSG00000123159 | *GIPC1* | 1.557807572 | 0.011624148 |
| ENSG00000089902 | *RCOR1* | 1.555761497 | 0.034828089 |
| ENSG00000163597 | *SNHG16* | 1.55437412 | 0.032809788 |
| ENSG00000196141 | *SPATS2L* | 1.551471109 | 0.032762112 |
| ENSG00000107789 | *MINPP1* | 1.55103792 | 0.01470768 |
| ENSG00000009844 | *VTA1* | 1.550773198 | 0.028864925 |
| ENSG00000139433 | *GLTP* | 1.549091664 | 0.026641471 |
| ENSG00000138758 | *11-Sep* | 1.546169227 | 0.018701062 |
| ENSG00000198176 | *TFDP1* | 1.542201416 | 0.011474841 |
| ENSG00000169851 | *PCDH7* | 1.533753393 | 0.043073431 |
| ENSG00000015475 | *BID* | 1.533527482 | 0.022497437 |
| ENSG00000151292 | *CSNK1G3* | 1.532036825 | 0.041310651 |
| ENSG00000234741 | *GAS5* | 1.531510566 | 0.041589626 |
| ENSG00000139921 | *TMX1* | 1.525938933 | 0.047684477 |
| ENSG00000164924 | *YWHAZ* | 1.520160988 | 0.036166216 |
| ENSG00000141401 | *IMPA2* | 1.517111318 | 0.032095186 |
| ENSG00000197535 | *MYO5A* | 1.515337755 | 0.049433619 |
| ENSG00000125266 | *EFNB2* | 1.510723325 | 0.02711559 |
| ENSG00000107566 | *ERLIN1* | 1.501491183 | 0.032082095 |
| ENSG00000070214 | *SLC44A1* | 1.498220963 | 0.043679007 |
| ENSG00000132256 | *TRIM5* | 1.496304059 | 0.043116764 |
| ENSG00000116260 | *QSOX1* | 1.493491071 | 0.026565932 |
| ENSG00000106780 | *MEGF9* | 1.48659139 | 0.042404805 |
| ENSG00000082701 | *GSK3B* | 1.476026056 | 0.021788951 |
| ENSG00000142192 | *APP* | 1.473620051 | 0.031415467 |
| ENSG00000137364 | *TPMT* | 1.469179291 | 0.023912027 |
| ENSG00000075151 | *EIF4G3* | 1.464745289 | 0.012508567 |
| ENSG00000095002 | *MSH2* | 1.45251954 | 0.042253578 |
| ENSG00000111371 | *SLC38A1* | 1.450100163 | 0.038274564 |
| ENSG00000170385 | *SLC30A1* | 1.436781641 | 0.04096512 |
| ENSG00000106367 | *AP1S1* | 1.433713353 | 0.032083794 |
| ENSG00000099810 | *MTAP* | 1.433391821 | 0.02839563 |
| ENSG00000163812 | *ZDHHC3* | 1.429589606 | 0.023852642 |
| ENSG00000177189 | *RPS6KA3* | 1.426652547 | 0.017222297 |
| ENSG00000163162 | *RNF149* | 1.426565584 | 0.035112556 |
| ENSG00000119314 | *PTBP3* | 1.419733373 | 0.023618312 |
| ENSG00000163950 | *SLBP* | 1.418387912 | 0.046010045 |
| ENSG00000117151 | *CTBS* | 1.401276373 | 0.039395664 |
| ENSG00000090054 | *SPTLC1* | 1.396926819 | 0.040793764 |
| ENSG00000119541 | *VPS4B* | 1.396123059 | 0.049445389 |
| ENSG00000116237 | *ICMT* | 1.392276989 | 0.020468428 |
| ENSG00000036257 | *CUL3* | 1.39086637 | 0.03310533 |
| ENSG00000114354 | *TFG* | 1.388720132 | 0.035540687 |
| ENSG00000179833 | *SERTAD2* | 1.382595288 | 0.033156243 |
| ENSG00000067225 | *PKM* | 1.374399829 | 0.028857675 |
| ENSG00000134684 | *YARS* | 1.369963891 | 0.049433619 |
| ENSG00000096063 | *SRPK1* | 1.36379273 | 0.038034188 |
| ENSG00000153113 | *CAST* | 1.359958726 | 0.041511498 |
| ENSG00000148154 | *UGCG* | 1.351811278 | 0.041758851 |
| ENSG00000138594 | *TMOD3* | 1.351223362 | 0.038585276 |
| ENSG00000116044 | *NFE2L2* | 1.346922725 | 0.026140292 |
| ENSG00000111726 | *CMAS* | 1.346701681 | 0.042334023 |
| ENSG00000128309 | *MPST* | 1.345306284 | 0.025468343 |
| ENSG00000164024 | *METAP1* | 1.335932147 | 0.04788399 |
| ENSG00000070756 | *PABPC1* | 1.296227741 | 0.033751905 |
| ENSG00000099204 | *ABLIM1* | 1.295065991 | 0.041292729 |
| ENSG00000187446 | *CHP1* | 1.275706939 | 0.036417644 |
| ENSG00000117360 | *PRPF3* | -1.324722067 | 0.034926883 |
| ENSG00000003756 | *RBM5* | -1.367561093 | 0.028857057 |
| ENSG00000198585 | *NUDT16* | -1.384909149 | 0.032825564 |
| ENSG00000108389 | *MTMR4* | -1.40205382 | 0.036063443 |
| ENSG00000088448 | *ANKRD10* | -1.41818526 | 0.038639713 |
| ENSG00000213339 | *QTRT1* | -1.423555375 | 0.046273624 |
| ENSG00000112763 | *BTN2A1* | -1.427293368 | 0.034745788 |
| ENSG00000167766 | *ZNF83* | -1.46201927 | 0.037120426 |
| ENSG00000072832 | *CRMP1* | -1.476660691 | 0.041609644 |
| ENSG00000150977 | *RILPL2* | -1.494433308 | 0.038693096 |
| ENSG00000104219 | *ZDHHC2* | -1.495662576 | 0.038836043 |
| ENSG00000160961 | *ZNF333* | -1.510225716 | 0.028443766 |
| ENSG00000167775 | *CD320* | -1.516293078 | 0.039870643 |
| ENSG00000224078 | *SNHG14* | -1.54198109 | 0.037021687 |
| ENSG00000170271 | *FAXDC2* | -1.543122728 | 0.036420956 |
| ENSG00000101849 | *TBL1X* | -1.550192924 | 0.036699874 |
| ENSG00000100523 | *DDHD1* | -1.55105785 | 0.034849469 |
| ENSG00000075234 | *TTC38* | -1.570245202 | 0.020984132 |
| ENSG00000004534 | *RBM6* | -1.577305253 | 0.023029049 |
| ENSG00000101577 | *LPIN2* | -1.581621338 | 0.022079056 |
| ENSG00000132274 | *TRIM22* | -1.582837477 | 0.036267026 |
| ENSG00000124104 | *SNX21* | -1.604272669 | 0.025390249 |
| ENSG00000051108 | *HERPUD1* | -1.606326443 | 0.021945805 |
| ENSG00000160613 | *PCSK7* | -1.609561597 | 0.02181069 |
| ENSG00000082014 | *SMARCD3* | -1.610972089 | 0.011129593 |
| ENSG00000126351 | *THRA* | -1.613658837 | 0.014208523 |
| ENSG00000117616 | *RSRP1* | -1.617373462 | 0.020824636 |
| ENSG00000108773 | *KAT2A* | -1.628180008 | 0.027061959 |
| ENSG00000068024 | *HDAC4* | -1.630732224 | 0.014520922 |
| ENSG00000143801 | *PSEN2* | -1.633458067 | 0.019708636 |
| ENSG00000132773 | *TOE1* | -1.64081343 | 0.04033015 |
| ENSG00000107186 | *MPDZ* | -1.65014095 | 0.023377881 |
| ENSG00000166912 | *MTMR10* | -1.659640496 | 0.026692232 |
| ENSG00000103064 | *SLC7A6* | -1.670214507 | 0.048324244 |
| ENSG00000125503 | *PPP1R12C* | -1.67504915 | 0.044824792 |
| ENSG00000112096 | *SOD2* | -1.676320329 | 0.039688336 |
| ENSG00000069974 | *RAB27A* | -1.685757911 | 0.037620931 |
| ENSG00000148175 | *STOM* | -1.700382672 | 0.039957714 |
| ENSG00000118965 | *WDR35* | -1.709406457 | 0.04414021 |
| ENSG00000135916 | *ITM2C* | -1.726493118 | 0.024068558 |
| ENSG00000261801 | *LOXL1-AS1* | -1.742897751 | 0.047251409 |
| ENSG00000170581 | *STAT2* | -1.751543771 | 0.035022646 |
| ENSG00000143344 | *RGL1* | -1.753077566 | 0.036173996 |
| ENSG00000166313 | *APBB1* | -1.762132439 | 0.015503446 |
| ENSG00000198105 | *ZNF248* | -1.767003087 | 0.019458066 |
| ENSG00000163596 | *ICA1L* | -1.769703637 | 0.01566868 |
| ENSG00000196693 | *ZNF33B* | -1.771110209 | 0.028383655 |
| ENSG00000137070 | *IL11RA* | -1.771902954 | 0.015453409 |
| ENSG00000157214 | *STEAP2* | -1.782228916 | 0.029929086 |
| ENSG00000179403 | *VWA1* | -1.784304333 | 0.024662168 |
| ENSG00000145022 | *TCTA* | -1.785168577 | 0.008318395 |
| ENSG00000212907 | *MT-ND4L* | -1.785831309 | 0.016940227 |
| ENSG00000183621 | *ZNF438* | -1.790709654 | 0.047775252 |
| ENSG00000235217 | *TSPY26P* | -1.795756997 | 0.023912027 |
| ENSG00000130844 | *ZNF331* | -1.799202802 | 0.038639713 |
| ENSG00000234585 | *CCT6P3* | -1.805699316 | 0.032082095 |
| ENSG00000163430 | *FSTL1* | -1.807663618 | 0.018100864 |
| ENSG00000228109 | *MFI2-AS1* | -1.817553818 | 0.046889677 |
| ENSG00000198886 | *MT-ND4* | -1.819412296 | 0.013610758 |
| ENSG00000153933 | *DGKE* | -1.820567779 | 0.026602528 |
| ENSG00000158062 | *UBXN11* | -1.826553223 | 0.014359283 |
| ENSG00000198873 | *GRK5* | -1.828664891 | 0.02203372 |
| ENSG00000167081 | *PBX3* | -1.829409433 | 0.010533672 |
| ENSG00000214548 | *MEG3* | -1.838941864 | 0.002321698 |
| ENSG00000163297 | *ANTXR2* | -1.847630253 | 0.028664141 |
| ENSG00000204149 | *AGAP6* | -1.855488288 | 0.025390249 |
| ENSG00000233608 | *TWIST2* | -1.859139215 | 0.035470251 |
| ENSG00000196507 | *TCEAL3* | -1.859781476 | 0.022964603 |
| ENSG00000110171 | *TRIM3* | -1.861871304 | 0.028719708 |
| ENSG00000257337 | *RP11-983P16.4* | -1.872680272 | 0.009527981 |
| ENSG00000006042 | *TMEM98* | -1.87890766 | 0.022858506 |
| ENSG00000182986 | *ZNF320* | -1.882924371 | 0.023259244 |
| ENSG00000173757 | *STAT5B* | -1.884990096 | 0.002711903 |
| ENSG00000158717 | *RNF166* | -1.885933846 | 0.025722547 |
| ENSG00000109501 | *WFS1* | -1.895532412 | 0.002877237 |
| ENSG00000179818 | *PCBP1-AS1* | -1.898139101 | 0.00715732 |
| ENSG00000177706 | *FAM20C* | -1.900858678 | 0.004535387 |
| ENSG00000143515 | *ATP8B2* | -1.908061073 | 0.03254072 |
| ENSG00000179094 | *PER1* | -1.908299824 | 0.048932685 |
| ENSG00000164088 | *PPM1M* | -1.911948861 | 0.019125344 |
| ENSG00000147576 | *ADHFE1* | -1.920654259 | 0.038492051 |
| ENSG00000175183 | *CSRP2* | -1.922163922 | 0.033653137 |
| ENSG00000164308 | *ERAP2* | -1.926639209 | 0.006740716 |
| ENSG00000145012 | *LPP* | -1.927426706 | 0.002543998 |
| ENSG00000113448 | *PDE4D* | -1.929306986 | 0.010998172 |
| ENSG00000128512 | *DOCK4* | -1.93834305 | 0.046608154 |
| ENSG00000156804 | *FBXO32* | -1.940314146 | 0.003980947 |
| ENSG00000068079 | *IFI35* | -1.941710338 | 0.003118368 |
| ENSG00000123096 | *SSPN* | -1.951846252 | 0.035866545 |
| ENSG00000213366 | *GSTM2* | -1.953215322 | 0.006379171 |
| ENSG00000186088 | *GSAP* | -1.953924176 | 0.034182917 |
| ENSG00000182983 | *ZNF662* | -1.95417121 | 0.01051844 |
| ENSG00000115556 | *PLCD4* | -1.958749299 | 0.021236081 |
| ENSG00000228253 | *MT-ATP8* | -1.961442734 | 0.003876473 |
| ENSG00000228223 | *HCG11* | -1.963030448 | 0.029455219 |
| ENSG00000105072 | *C19orf44* | -1.96340776 | 0.022224289 |
| ENSG00000232931 | *LINC00342* | -1.965137333 | 0.034810396 |
| ENSG00000113231 | *PDE8B* | -1.965681511 | 0.038445024 |
| ENSG00000111252 | *SH2B3* | -1.971505446 | 0.047367097 |
| ENSG00000150867 | *PIP4K2A* | -1.973799462 | 0.006673033 |
| ENSG00000139192 | *TAPBPL* | -1.975265796 | 0.042843064 |
| ENSG00000162341 | *TPCN2* | -1.98189843 | 0.018216971 |
| ENSG00000171385 | *KCND3* | -1.987427716 | 0.025255037 |
| ENSG00000187239 | *FNBP1* | -1.992184987 | 0.001938384 |
| ENSG00000182700 | *IGIP* | -1.993144111 | 0.00939076 |
| ENSG00000167528 | *ZNF641* | -1.994104274 | 0.001844002 |
| ENSG00000120458 | *MSANTD2* | -1.997382529 | 0.016407826 |
| ENSG00000071073 | *MGAT4A* | -1.99996125 | 0.014168659 |
| ENSG00000243943 | *ZNF512* | -2.004020167 | 0.002535802 |
| ENSG00000153179 | *RASSF3* | -2.008511719 | 0.023618312 |
| ENSG00000157110 | *RBPMS* | -2.011254203 | 0.002023502 |
| ENSG00000172878 | *METAP1D* | -2.013498474 | 0.018020971 |
| ENSG00000164056 | *SPRY1* | -2.014732567 | 0.027780686 |
| ENSG00000267519 | *CTD-3252C9.4* | -2.020512096 | 0.011071317 |
| ENSG00000198556 | *ZNF789* | -2.022141403 | 0.002131892 |
| ENSG00000071282 | *LMCD1* | -2.028729524 | 0.043267378 |
| ENSG00000198899 | *MT-ATP6* | -2.033485821 | 0.004082476 |
| ENSG00000156398 | *SFXN2* | -2.038537424 | 0.029398911 |
| ENSG00000157601 | *MX1* | -2.038918193 | 0.015732464 |
| ENSG00000133121 | *STARD13* | -2.039574511 | 0.041153425 |
| ENSG00000054983 | *GALC* | -2.041089142 | 0.00849337 |
| ENSG00000229320 | *KRT8P12* | -2.043964434 | 0.049787851 |
| ENSG00000128335 | *APOL2* | -2.053689318 | 0.004602364 |
| ENSG00000111752 | *PHC1* | -2.055150604 | 0.013324633 |
| ENSG00000155093 | *PTPRN2* | -2.061323869 | 0.008120584 |
| ENSG00000240038 | *AMY2B* | -2.06437454 | 0.042193885 |
| ENSG00000166562 | *SEC11C* | -2.069584981 | 0.007979089 |
| ENSG00000198727 | *MT-CYB* | -2.077377361 | 0.005322965 |
| ENSG00000117643 | *MAN1C1* | -2.079476871 | 0.012741124 |
| ENSG00000154930 | *ACSS1* | -2.083363874 | 0.029802343 |
| ENSG00000221944 | *TIGD1* | -2.08511315 | 0.023047715 |
| ENSG00000105472 | *CLEC11A* | -2.089345082 | 0.027886123 |
| ENSG00000225630 | *MTND2P28* | -2.09076319 | 0.003430769 |
| ENSG00000248527 | *MTATP6P1* | -2.100157283 | 0.002850341 |
| ENSG00000196263 | *ZNF471* | -2.107994903 | 0.046278204 |
| ENSG00000114626 | *ABTB1* | -2.112887238 | 0.007165623 |
| ENSG00000185404 | *SP140L* | -2.116972433 | 0.003971411 |
| ENSG00000105339 | *DENND3* | -2.119442341 | 0.018717133 |
| ENSG00000147526 | *TACC1* | -2.125330182 | 0.00195103 |
| ENSG00000150995 | *ITPR1* | -2.12584702 | 0.032998954 |
| ENSG00000172197 | *MBOAT1* | -2.129688288 | 0.026819185 |
| ENSG00000104936 | *DMPK* | -2.13435312 | 0.009102644 |
| ENSG00000113621 | *TXNDC15* | -2.139431899 | 0.003936054 |
| ENSG00000174428 | *GTF2IRD2B* | -2.142096414 | 0.002002858 |
| ENSG00000172638 | *EFEMP2* | -2.145845901 | 0.000622847 |
| ENSG00000100342 | *APOL1* | -2.146809725 | 0.004235686 |
| ENSG00000125434 | *SLC25A35* | -2.15786719 | 0.039972512 |
| ENSG00000272419 | *RP11-403I13.8* | -2.163519039 | 0.048285337 |
| ENSG00000248487 | *ABHD14A* | -2.16846573 | 0.012476164 |
| ENSG00000169604 | *ANTXR1* | -2.171491889 | 0.006340554 |
| ENSG00000168067 | *MAP4K2* | -2.171972163 | 0.008746408 |
| ENSG00000165868 | *HSPA12A* | -2.178603696 | 0.009349613 |
| ENSG00000238083 | *LRRC37A2* | -2.180525719 | 0.049023676 |
| ENSG00000169087 | *HSPBAP1* | -2.188568789 | 0.020308165 |
| ENSG00000257151 | *PWAR6* | -2.189036832 | 0.027241425 |
| ENSG00000179598 | *PLD6* | -2.192095592 | 0.020123116 |
| ENSG00000112561 | *TFEB* | -2.19591341 | 0.00718601 |
| ENSG00000139597 | *N4BP2L1* | -2.202056429 | 0.008017371 |
| ENSG00000079819 | *EPB41L2* | -2.203688082 | 0.000969902 |
| ENSG00000196267 | *ZNF836* | -2.204296389 | 0.036420956 |
| ENSG00000133624 | *ZNF767P* | -2.208119488 | 0.004860926 |
| ENSG00000174080 | *CTSF* | -2.20860292 | 0.042389093 |
| ENSG00000135736 | *CCDC102A* | -2.210059862 | 0.027640777 |
| ENSG00000080947 | *CROCCP3* | -2.213977231 | 0.037926666 |
| ENSG00000260563 | *RP13-516M14.1* | -2.222776531 | 0.01697871 |
| ENSG00000267100 | *ILF3-AS1* | -2.226388345 | 0.002191496 |
| ENSG00000184381 | *PLA2G6* | -2.227556563 | 0.034267936 |
| ENSG00000111674 | *ENO2* | -2.22825953 | 0.000601844 |
| ENSG00000183722 | *LHFP* | -2.232345227 | 0.026749135 |
| ENSG00000144445 | *KANSL1L* | -2.233594043 | 0.010761527 |
| ENSG00000167604 | *NFKBID* | -2.239637689 | 0.020824636 |
| ENSG00000154114 | *TBCEL* | -2.241677749 | 0.023618312 |
| ENSG00000151376 | *ME3* | -2.241784177 | 0.002085165 |
| ENSG00000185483 | *ROR1* | -2.246518673 | 0.02621214 |
| ENSG00000053702 | *NRIP2* | -2.252115429 | 0.03053652 |
| ENSG00000198763 | *MT-ND2* | -2.253248 | 0.002322157 |
| ENSG00000165716 | *FAM69B* | -2.254647716 | 0.02409031 |
| ENSG00000165424 | *ZCCHC24* | -2.264279225 | 0.000527386 |
| ENSG00000108830 | *RND2* | -2.266583348 | 0.035010403 |
| ENSG00000231711 | *LINC00899* | -2.267832932 | 0.023912027 |
| ENSG00000003989 | *SLC7A2* | -2.271646665 | 0.039870643 |
| ENSG00000214357 | *NEURL1B* | -2.271700275 | 0.002811324 |
| ENSG00000132522 | *GPS2* | -2.27296958 | 0.031778955 |
| ENSG00000145569 | *FAM105A* | -2.281290205 | 0.027914988 |
| ENSG00000111885 | *MAN1A1* | -2.281360483 | 0.025909788 |
| ENSG00000076555 | *ACACB* | -2.284031576 | 0.011826438 |
| ENSG00000196440 | *ARMCX4* | -2.285658942 | 0.003664708 |
| ENSG00000229180 | *GS1-124K5.11* | -2.292180422 | 0.010701662 |
| ENSG00000162409 | *PRKAA2* | -2.293766401 | 0.028383655 |
| ENSG00000198933 | *TBKBP1* | -2.294886672 | 0.023618312 |
| ENSG00000154175 | *ABI3BP* | -2.295769338 | 0.047816356 |
| ENSG00000087086 | *FTL* | -2.297071836 | 0.003595895 |
| ENSG00000121310 | *ECHDC2* | -2.301791245 | 0.001536853 |
| ENSG00000119946 | *CNNM1* | -2.306511295 | 0.04251649 |
| ENSG00000196275 | *GTF2IRD2* | -2.306985438 | 0.006686999 |
| ENSG00000161647 | *MPP3* | -2.31016026 | 0.011618834 |
| ENSG00000177694 | *NAALADL2* | -2.311514303 | 0.018662442 |
| ENSG00000138074 | *SLC5A6* | -2.312399886 | 0.000701528 |
| ENSG00000198939 | *ZFP2* | -2.31295265 | 0.042800858 |
| ENSG00000165113 | *GKAP1* | -2.312983704 | 0.033876058 |
| ENSG00000214274 | *ANG* | -2.314381429 | 0.027312571 |
| ENSG00000113273 | *ARSB* | -2.315858015 | 0.01803954 |
| ENSG00000112425 | *EPM2A* | -2.317696072 | 0.008351078 |
| ENSG00000058056 | *USP13* | -2.322403082 | 0.001791418 |
| ENSG00000236609 | *ZNF853* | -2.333427495 | 0.009102644 |
| ENSG00000269858 | *EGLN2* | -2.335877078 | 0.001924275 |
| ENSG00000164430 | *MB21D1* | -2.33692793 | 0.047159724 |
| ENSG00000059378 | *PARP12* | -2.336992108 | 0.012709216 |
| ENSG00000069493 | *CLEC2D* | -2.340898118 | 0.016955893 |
| ENSG00000185885 | *IFITM1* | -2.348579009 | 0.004926875 |
| ENSG00000128849 | *CGNL1* | -2.350222348 | 0.006290342 |
| ENSG00000161298 | *ZNF382* | -2.351237087 | 0.046376024 |
| ENSG00000115594 | *IL1R1* | -2.351853798 | 0.000172408 |
| ENSG00000113971 | *NPHP3* | -2.353025285 | 0.001516445 |
| ENSG00000140937 | *CDH11* | -2.358163385 | 0.034807093 |
| ENSG00000138185 | *ENTPD1* | -2.359799636 | 0.001988148 |
| ENSG00000177337 | *DLGAP1-AS1* | -2.360255579 | 0.008351078 |
| ENSG00000109738 | *GLRB* | -2.362910128 | 0.047775252 |
| ENSG00000119711 | *ALDH6A1* | -2.366453939 | 0.001373102 |
| ENSG00000156218 | *ADAMTSL3* | -2.369097752 | 0.004297643 |
| ENSG00000144712 | *CAND2* | -2.37116609 | 0.024225231 |
| ENSG00000143878 | *RHOB* | -2.373653886 | 0.006425613 |
| ENSG00000162426 | *SLC45A1* | -2.375550509 | 0.00715732 |
| ENSG00000178764 | *ZHX2* | -2.376375511 | 0.000247746 |
| ENSG00000213865 | *C8orf44* | -2.377527731 | 0.015427083 |
| ENSG00000270189 | *RP11-258C19.7* | -2.382424888 | 0.003564878 |
| ENSG00000184205 | *TSPYL2* | -2.384780174 | 0.000240551 |
| ENSG00000141519 | *CCDC40* | -2.386100475 | 0.041119621 |
| ENSG00000111450 | *STX2* | -2.38849353 | 0.049534162 |
| ENSG00000091592 | *NLRP1* | -2.389894774 | 0.025532797 |
| ENSG00000259891 | *CTA-204B4.2* | -2.390067333 | 0.040482508 |
| ENSG00000166405 | *RIC3* | -2.3946984 | 0.030624098 |
| ENSG00000111801 | *BTN3A3* | -2.396600116 | 0.011565509 |
| ENSG00000275367 | *RP11-266K4.14* | -2.403705851 | 0.041994663 |
| ENSG00000074657 | *ZNF532* | -2.404577828 | 0.001197871 |
| ENSG00000166343 | *MSS51* | -2.405340426 | 0.017460696 |
| ENSG00000272645 | *RP11-504P24.8* | -2.408285006 | 0.009872583 |
| ENSG00000163803 | *PLB1* | -2.411215057 | 0.025295096 |
| ENSG00000100968 | *NFATC4* | -2.414001968 | 6.05E-05 |
| ENSG00000196923 | *PDLIM7* | -2.41621098 | 0.00066624 |
| ENSG00000111666 | *CHPT1* | -2.416572579 | 0.00778713 |
| ENSG00000219607 | *PPP1R3G* | -2.416698677 | 0.0231492 |
| ENSG00000142089 | *IFITM3* | -2.423106436 | 0.001436404 |
| ENSG00000092964 | *DPYSL2* | -2.424009208 | 0.008632319 |
| ENSG00000241015 | *TPM3P9* | -2.426369615 | 0.013928302 |
| ENSG00000071205 | *ARHGAP10* | -2.428067286 | 0.001982246 |
| ENSG00000184271 | *POU6F1* | -2.433540969 | 0.00437034 |
| ENSG00000139508 | *SLC46A3* | -2.4372946 | 0.003738043 |
| ENSG00000141480 | *ARRB2* | -2.439765124 | 0.006667196 |
| ENSG00000078487 | *ZCWPW1* | -2.441925971 | 0.00149001 |
| ENSG00000100116 | *GCAT* | -2.442500293 | 0.000412199 |
| ENSG00000134986 | *NREP* | -2.454255017 | 0.027104945 |
| ENSG00000134262 | *AP4B1* | -2.455230615 | 0.004403168 |
| ENSG00000176293 | *ZNF135* | -2.455976078 | 0.00315307 |
| ENSG00000249992 | *TMEM158* | -2.456381157 | 0.00670167 |
| ENSG00000128709 | *HOXD9* | -2.45858359 | 0.041950343 |
| ENSG00000139194 | *RBP5* | -2.458809955 | 0.018590092 |
| ENSG00000165874 | *FAM35BP* | -2.459399679 | 0.047504118 |
| ENSG00000025434 | *NR1H3* | -2.461951588 | 0.016567578 |
| ENSG00000134470 | *IL15RA* | -2.462229329 | 0.025363036 |
| ENSG00000271646 | *RP11-326I11.3* | -2.462579033 | 0.029802343 |
| ENSG00000173917 | *HOXB2* | -2.465468775 | 0.036288882 |
| ENSG00000233841 | *HLA-C* | -2.470951342 | 0.032762112 |
| ENSG00000183718 | *TRIM52* | -2.47890284 | 0.000500276 |
| ENSG00000110237 | *ARHGEF17* | -2.482894506 | 0.002707425 |
| ENSG00000222009 | *BTBD19* | -2.484394791 | 0.024224113 |
| ENSG00000134245 | *WNT2B* | -2.488017475 | 0.011316825 |
| ENSG00000090530 | *P3H2* | -2.490212642 | 0.049416147 |
| ENSG00000165410 | *CFL2* | -2.492839119 | 0.041349781 |
| ENSG00000161265 | *U2AF1L4* | -2.493399637 | 0.006812046 |
| ENSG00000152137 | *HSPB8* | -2.497037242 | 0.007119239 |
| ENSG00000178498 | *DTX3* | -2.49853931 | 0.000413128 |
| ENSG00000104998 | *IL27RA* | -2.500747651 | 0.011229802 |
| ENSG00000248124 | *RRN3P1* | -2.503144026 | 0.001613592 |
| ENSG00000198888 | *MT-ND1* | -2.503615467 | 0.002500392 |
| ENSG00000157514 | *TSC22D3* | -2.504188437 | 0.009497279 |
| ENSG00000164125 | *FAM198B* | -2.504469491 | 0.001298083 |
| ENSG00000273847 | *RP11-846E15.2* | -2.504971731 | 0.02827262 |
| ENSG00000157350 | *ST3GAL2* | -2.505464735 | 0.022032353 |
| ENSG00000186994 | *KANK3* | -2.506769841 | 0.020581415 |
| ENSG00000166710 | *B2M* | -2.522628841 | 0.00050701 |
| ENSG00000176700 | *SCAND2P* | -2.529531293 | 0.008673415 |
| ENSG00000134285 | *FKBP11* | -2.535621359 | 0.013835943 |
| ENSG00000103196 | *CRISPLD2* | -2.537310406 | 0.000670117 |
| ENSG00000136895 | *GARNL3* | -2.539261061 | 0.001077206 |
| ENSG00000100307 | *CBX7* | -2.547377248 | 4.21E-05 |
| ENSG00000166762 | *CATSPER2* | -2.548029045 | 0.01266812 |
| ENSG00000126878 | *AIF1L* | -2.550069091 | 0.019907712 |
| ENSG00000162639 | *HENMT1* | -2.554722171 | 0.016684756 |
| ENSG00000187608 | *ISG15* | -2.554773551 | 0.018836358 |
| ENSG00000218227 | *RP11-889L3.1* | -2.555920548 | 0.02121024 |
| ENSG00000168734 | *PKIG* | -2.556872175 | 0.015145538 |
| ENSG00000231889 | *TRAF3IP2-AS1* | -2.5597836 | 0.022093969 |
| ENSG00000196405 | *EVL* | -2.560973119 | 0.000718657 |
| ENSG00000113108 | *APBB3* | -2.574003097 | 0.001711729 |
| ENSG00000165028 | *NIPSNAP3B* | -2.576634759 | 0.036338896 |
| ENSG00000179820 | *MYADM* | -2.576699192 | 0.000468432 |
| ENSG00000146674 | *IGFBP3* | -2.584840308 | 0.010370464 |
| ENSG00000088543 | *C3orf18* | -2.584977158 | 0.004243375 |
| ENSG00000167995 | *BEST1* | -2.587722734 | 0.018929031 |
| ENSG00000276168 | *RN7SL1* | -2.589807654 | 0.039649727 |
| ENSG00000115526 | *CHST10* | -2.590814038 | 0.003086023 |
| ENSG00000150764 | *DIXDC1* | -2.593895999 | 0.006369535 |
| ENSG00000143178 | *TBX19* | -2.594970314 | 0.034807093 |
| ENSG00000076706 | *MCAM* | -2.595344628 | 0.003261747 |
| ENSG00000137628 | *DDX60* | -2.598961495 | 0.000735032 |
| ENSG00000130775 | *THEMIS2* | -2.602680074 | 0.035552538 |
| ENSG00000176401 | *EID2B* | -2.604347423 | 0.03148022 |
| ENSG00000271601 | *LIX1L* | -2.606325674 | 0.002811324 |
| ENSG00000196924 | *FLNA* | -2.608879631 | 0.003505837 |
| ENSG00000077782 | *FGFR1* | -2.614002564 | 0.000812146 |
| ENSG00000273802 | *HIST1H2BG* | -2.615770794 | 0.03291644 |
| ENSG00000112769 | *LAMA4* | -2.626369276 | 0.008433446 |
| ENSG00000130653 | *PNPLA7* | -2.627213195 | 0.047240912 |
| ENSG00000131016 | *AKAP12* | -2.627778366 | 0.041208694 |
| ENSG00000197256 | *KANK2* | -2.628721274 | 0.000425624 |
| ENSG00000234912 | *SNHG20* | -2.629769422 | 0.004546278 |
| ENSG00000132182 | *NUP210* | -2.630244008 | 0.028774804 |
| ENSG00000233695 | *GAS6-AS1* | -2.637715912 | 0.012939228 |
| ENSG00000169891 | *REPS2* | -2.640148994 | 0.009300804 |
| ENSG00000140263 | *SORD* | -2.644057722 | 0.006179182 |
| ENSG00000198429 | *ZNF69* | -2.644916073 | 0.014587476 |
| ENSG00000246985 | *SOCS2-AS1* | -2.647685819 | 0.009885748 |
| ENSG00000185338 | *SOCS1* | -2.647762687 | 0.006754773 |
| ENSG00000077942 | *FBLN1* | -2.658896343 | 0.006717753 |
| ENSG00000102878 | *HSF4* | -2.659401103 | 0.005382511 |
| ENSG00000135363 | *LMO2* | -2.666924861 | 0.012734566 |
| ENSG00000116117 | *PARD3B* | -2.668969892 | 0.020381849 |
| ENSG00000161544 | *CYGB* | -2.669065754 | 0.001360714 |
| ENSG00000177721 | *ANXA2R* | -2.67581564 | 0.028383655 |
| ENSG00000082512 | *TRAF5* | -2.676251451 | 0.000466633 |
| ENSG00000136147 | *PHF11* | -2.682697108 | 6.14E-05 |
| ENSG00000105711 | *SCN1B* | -2.687415758 | 0.019117212 |
| ENSG00000161791 | *FMNL3* | -2.688137718 | 0.020724085 |
| ENSG00000166974 | *MAPRE2* | -2.689483381 | 0.005989915 |
| ENSG00000213398 | *LCAT* | -2.689899538 | 0.00055613 |
| ENSG00000078687 | *TNRC6C* | -2.691943914 | 1.66E-05 |
| ENSG00000181804 | *SLC9A9* | -2.697636302 | 0.003252217 |
| ENSG00000138642 | *HERC6* | -2.699639429 | 9.00E-05 |
| ENSG00000261584 | *RP11-457M11.5* | -2.711288508 | 0.041349781 |
| ENSG00000110852 | *CLEC2B* | -2.716962153 | 0.000641949 |
| ENSG00000117228 | *GBP1* | -2.719874008 | 0.000677604 |
| ENSG00000162946 | *DISC1* | -2.724328969 | 0.042885489 |
| ENSG00000186056 | *MATN1-AS1* | -2.724604073 | 0.044864211 |
| ENSG00000180340 | *FZD2* | -2.727400336 | 0.038793895 |
| ENSG00000128045 | *RASL11B* | -2.72977649 | 0.015290641 |
| ENSG00000101347 | *SAMHD1* | -2.734340418 | 0.000133695 |
| ENSG00000203497 | *PDCD4-AS1* | -2.736293998 | 0.025845181 |
| ENSG00000135929 | *CYP27A1* | -2.737023899 | 0.002602471 |
| ENSG00000116741 | *RGS2* | -2.75004048 | 0.013670767 |
| ENSG00000091986 | *CCDC80* | -2.753625086 | 0.022147285 |
| ENSG00000197905 | *TEAD4* | -2.754263626 | 0.006694233 |
| ENSG00000134775 | *FHOD3* | -2.754543849 | 0.01248602 |
| ENSG00000134802 | *SLC43A3* | -2.755056587 | 0.008879591 |
| ENSG00000121316 | *PLBD1* | -2.755486471 | 0.024591319 |
| ENSG00000149328 | *GLB1L2* | -2.755738508 | 0.000497389 |
| ENSG00000065675 | *PRKCQ* | -2.761001978 | 0.046335586 |
| ENSG00000134072 | *CAMK1* | -2.774144658 | 0.008673415 |
| ENSG00000059377 | *TBXAS1* | -2.778401828 | 0.00165152 |
| ENSG00000031081 | *ARHGAP31* | -2.781072167 | 0.000625019 |
| ENSG00000119714 | *GPR68* | -2.781119205 | 0.016762442 |
| ENSG00000185010 | *F8* | -2.785271321 | 0.008679221 |
| ENSG00000067191 | *CACNB1* | -2.791948337 | 0.001068226 |
| ENSG00000114698 | *PLSCR4* | -2.791964327 | 0.000598398 |
| ENSG00000171227 | *TMEM37* | -2.793935511 | 0.006054199 |
| ENSG00000145287 | *PLAC8* | -2.800210353 | 0.047498072 |
| ENSG00000015479 | *MATR3* | -2.807435496 | 0.049993785 |
| ENSG00000137098 | *SPAG8* | -2.808019348 | 0.013585908 |
| ENSG00000050767 | *COL23A1* | -2.808234495 | 0.026179307 |
| ENSG00000113209 | *PCDHB5* | -2.811271982 | 0.027640777 |
| ENSG00000106948 | *AKNA* | -2.817138378 | 0.003918162 |
| ENSG00000103489 | *XYLT1* | -2.820632943 | 0.005459428 |
| ENSG00000274712 | *RP11-147L13.15* | -2.82532549 | 0.016499391 |
| ENSG00000259820 | *AC083843.1* | -2.835350186 | 0.033232544 |
| ENSG00000159176 | *CSRP1* | -2.844377029 | 0.001957807 |
| ENSG00000105483 | *CARD8* | -2.845768898 | 7.72E-05 |
| ENSG00000162882 | *HAAO* | -2.848933632 | 8.31E-05 |
| ENSG00000137198 | *GMPR* | -2.855854032 | 0.000125995 |
| ENSG00000116574 | *RHOU* | -2.857054242 | 0.015503446 |
| ENSG00000198963 | *RORB* | -2.858978536 | 0.016472199 |
| ENSG00000198840 | *MT-ND3* | -2.861073553 | 0.000339759 |
| ENSG00000112759 | *SLC29A1* | -2.869617666 | 0.000234851 |
| ENSG00000183578 | *TNFAIP8L3* | -2.879373799 | 0.022753278 |
| ENSG00000130303 | *BST2* | -2.883459357 | 0.022652763 |
| ENSG00000188039 | *NWD1* | -2.885095949 | 0.02131182 |
| ENSG00000084636 | *COL16A1* | -2.89984578 | 0.000578874 |
| ENSG00000110031 | *LPXN* | -2.904493428 | 0.013180923 |
| ENSG00000102575 | *ACP5* | -2.906688624 | 0.007910624 |
| ENSG00000273686 | *B2M* | -2.907006529 | 0.007530104 |
| ENSG00000035862 | *TIMP2* | -2.91331452 | 8.78E-05 |
| ENSG00000137094 | *DNAJB5* | -2.923821114 | 0.009312355 |
| ENSG00000198208 | *RPS6KL1* | -2.926952167 | 0.011921296 |
| ENSG00000261884 | *CTC-479C5.12* | -2.936183229 | 0.021953214 |
| ENSG00000108797 | *CNTNAP1* | -2.938526165 | 0.000484674 |
| ENSG00000168404 | *MLKL* | -2.939206007 | 2.59E-06 |
| ENSG00000090339 | *ICAM1* | -2.945053792 | 0.019684121 |
| ENSG00000091513 | *TF* | -2.948713539 | 0.042613139 |
| ENSG00000124191 | *TOX2* | -2.949501823 | 0.00423642 |
| ENSG00000214063 | *TSPAN4* | -2.953217852 | 0.001759885 |
| ENSG00000135678 | *CPM* | -2.954527457 | 0.013432593 |
| ENSG00000228299 | *HLA-C* | -2.958626048 | 0.000163802 |
| ENSG00000159713 | *TPPP3* | -2.959806585 | 0.001807118 |
| ENSG00000079150 | *FKBP7* | -2.960645118 | 0.007132884 |
| ENSG00000153898 | *MCOLN2* | -2.961490356 | 0.0418218 |
| ENSG00000168060 | *NAALADL1* | -2.963736217 | 0.009266964 |
| ENSG00000260686 | *CTB-36H16.2* | -2.963980183 | 0.03412588 |
| ENSG00000100292 | *HMOX1* | -2.963994866 | 0.006922142 |
| ENSG00000141574 | *SECTM1* | -2.968182454 | 0.046010045 |
| ENSG00000184060 | *ADAP2* | -2.970605806 | 0.010257804 |
| ENSG00000173068 | *BNC2* | -2.97122839 | 0.02498096 |
| ENSG00000065989 | *PDE4A* | -2.97270642 | 0.003292585 |
| ENSG00000140416 | *TPM1* | -2.973157229 | 0.005956618 |
| ENSG00000166833 | *NAV2* | -2.975192295 | 0.015268743 |
| ENSG00000184194 | *GPR173* | -2.977311783 | 0.041349781 |
| ENSG00000117020 | *AKT3* | -2.977890414 | 0.000858626 |
| ENSG00000185070 | *FLRT2* | -2.980296025 | 0.029284162 |
| ENSG00000260428 | *SCX* | -2.990958036 | 0.035409635 |
| ENSG00000026950 | *BTN3A1* | -2.992174535 | 0.002662891 |
| ENSG00000074416 | *MGLL* | -2.993079528 | 2.46E-05 |
| ENSG00000177409 | *SAMD9L* | -2.994017165 | 0.000492073 |
| ENSG00000134198 | *TSPAN2* | -2.999122185 | 0.002991183 |
| ENSG00000146477 | *SLC22A3* | -3.002234854 | 0.000168372 |
| ENSG00000130830 | *MPP1* | -3.004270944 | 3.81E-05 |
| ENSG00000185532 | *PRKG1* | -3.007115656 | 0.009247176 |
| ENSG00000257354 | *RP11-631N16.2* | -3.012908253 | 0.009183564 |
| ENSG00000220785 | *MTMR9LP* | -3.017697907 | 0.000379673 |
| ENSG00000180113 | *TDRD6* | -3.018520404 | 0.025434065 |
| ENSG00000104332 | *SFRP1* | -3.021857117 | 0.000886686 |
| ENSG00000132330 | *SCLY* | -3.026179109 | 0.044912425 |
| ENSG00000112208 | *BAG2* | -3.026985714 | 0.002543998 |
| ENSG00000259605 | *AC074212.5* | -3.031320586 | 0.04477557 |
| ENSG00000196154 | *S100A4* | -3.031504157 | 0.011408089 |
| ENSG00000056998 | *GYG2* | -3.035587422 | 0.001201443 |
| ENSG00000109610 | *SOD3* | -3.03616759 | 0.00209724 |
| ENSG00000237753 | *AC079922.3* | -3.040483734 | 0.022093969 |
| ENSG00000198133 | *TMEM229B* | -3.044241165 | 0.000731721 |
| ENSG00000149177 | *PTPRJ* | -3.046860546 | 0.000221849 |
| ENSG00000176387 | *HSD11B2* | -3.047752124 | 0.041757244 |
| ENSG00000143434 | *SEMA6C* | -3.050074528 | 0.003917394 |
| ENSG00000105270 | *CLIP3* | -3.05206858 | 0.005346554 |
| ENSG00000102886 | *GDPD3* | -3.05215754 | 0.002633106 |
| ENSG00000081818 | *PCDHB4* | -3.062182175 | 0.031030568 |
| ENSG00000083814 | *ZNF671* | -3.062862594 | 0.000150305 |
| ENSG00000188130 | *MAPK12* | -3.064867965 | 0.023458397 |
| ENSG00000112293 | *GPLD1* | -3.068173966 | 0.006721506 |
| ENSG00000186470 | *BTN3A2* | -3.070660944 | 0.00075826 |
| ENSG00000101460 | *MAP1LC3A* | -3.071026973 | 2.68E-05 |
| ENSG00000160318 | *CLDND2* | -3.077007327 | 0.045501388 |
| ENSG00000064989 | *CALCRL* | -3.077858932 | 0.00582545 |
| ENSG00000102904 | *TSNAXIP1* | -3.089290847 | 0.027988923 |
| ENSG00000243335 | *KCTD7* | -3.094507872 | 5.05E-05 |
| ENSG00000167315 | *ACAA2* | -3.094543833 | 0.001057308 |
| ENSG00000168026 | *TTC21A* | -3.096974444 | 2.43E-05 |
| ENSG00000259972 | *AC009120.6* | -3.099322061 | 0.002030059 |
| ENSG00000125257 | *ABCC4* | -3.104078942 | 0.006467651 |
| ENSG00000164776 | *PHKG1* | -3.104114259 | 0.028549344 |
| ENSG00000159674 | *SPON2* | -3.109388009 | 0.010664261 |
| ENSG00000004777 | *ARHGAP33* | -3.109478804 | 0.014691932 |
| ENSG00000188206 | *HNRNPU-AS1* | -3.110912908 | 0.025390249 |
| ENSG00000143995 | *MEIS1* | -3.120484555 | 3.18E-05 |
| ENSG00000109794 | *FAM149A* | -3.121889575 | 0.001564318 |
| ENSG00000063127 | *SLC6A16* | -3.122326695 | 0.001925281 |
| ENSG00000169083 | *AR* | -3.124728527 | 0.014359283 |
| ENSG00000148288 | *GBGT1* | -3.127640622 | 0.000306987 |
| ENSG00000077684 | *JADE1* | -3.130735832 | 0.00093451 |
| ENSG00000183098 | *GPC6* | -3.132241437 | 0.031357373 |
| ENSG00000168306 | *ACOX2* | -3.135463888 | 0.007089792 |
| ENSG00000196730 | *DAPK1* | -3.135897757 | 0.034840897 |
| ENSG00000074706 | *IPCEF1* | -3.148556767 | 0.043899771 |
| ENSG00000198948 | *MFAP3L* | -3.149922549 | 0.015290641 |
| ENSG00000122824 | *NUDT10* | -3.15007986 | 0.048995295 |
| ENSG00000143333 | *RGS16* | -3.15444066 | 0.021414913 |
| ENSG00000135218 | *CD36* | -3.155148621 | 0.018135667 |
| ENSG00000109771 | *LRP2BP* | -3.155546274 | 0.006336846 |
| ENSG00000136383 | *ALPK3* | -3.156541147 | 0.025722547 |
| ENSG00000167874 | *TMEM88* | -3.157532507 | 0.015349709 |
| ENSG00000272870 | *RP11-798M19.6* | -3.158217167 | 0.00535971 |
| ENSG00000183963 | *SMTN* | -3.158502159 | 1.29E-05 |
| ENSG00000165338 | *HECTD2* | -3.163974201 | 7.34E-05 |
| ENSG00000263345 | *RP1-59D14.5* | -3.165131197 | 0.046376674 |
| ENSG00000162004 | *CCDC78* | -3.165935863 | 0.025284864 |
| ENSG00000147408 | *CSGALNACT1* | -3.166942585 | 0.003420001 |
| ENSG00000008516 | *MMP25* | -3.168540239 | 0.043267378 |
| ENSG00000152784 | *PRDM8* | -3.173728282 | 0.000823518 |
| ENSG00000106560 | *GIMAP2* | -3.17605695 | 0.003917394 |
| ENSG00000134533 | *RERG* | -3.179603787 | 0.000670539 |
| ENSG00000198467 | *TPM2* | -3.179881767 | 0.002448557 |
| ENSG00000187193 | *MT1X* | -3.180782962 | 0.004376637 |
| ENSG00000187391 | *MAGI2* | -3.185505794 | 0.002038276 |
| ENSG00000148848 | *ADAM12* | -3.19128498 | 0.038585276 |
| ENSG00000196814 | *MVB12B* | -3.193481319 | 0.000363736 |
| ENSG00000172889 | *EGFL7* | -3.202902253 | 1.12E-05 |
| ENSG00000242282 | *AC108488.4* | -3.20902149 | 0.014660395 |
| ENSG00000147174 | *ACRC* | -3.2099189 | 0.013670767 |
| ENSG00000183281 | *PLGLB1* | -3.212282918 | 0.001599915 |
| ENSG00000185201 | *IFITM2* | -3.214105208 | 0.000439525 |
| ENSG00000136040 | *PLXNC1* | -3.214657964 | 0.001342858 |
| ENSG00000162944 | *RFTN2* | -3.218952697 | 0.030013485 |
| ENSG00000203772 | *SPRN* | -3.220444939 | 0.011103366 |
| ENSG00000165124 | *SVEP1* | -3.220462792 | 0.009666805 |
| ENSG00000215217 | *C5orf49* | -3.221037284 | 0.021291204 |
| ENSG00000172164 | *SNTB1* | -3.221989223 | 0.003433988 |
| ENSG00000215068 | *AC025171.1* | -3.225383815 | 0.030105495 |
| ENSG00000162804 | *SNED1* | -3.22592714 | 0.000999691 |
| ENSG00000122786 | *CALD1* | -3.228336065 | 0.000620762 |
| ENSG00000125430 | *HS3ST3B1* | -3.229212752 | 0.005978393 |
| ENSG00000145244 | *CORIN* | -3.23137438 | 0.049943599 |
| ENSG00000112320 | *SOBP* | -3.241153228 | 1.28E-05 |
| ENSG00000182218 | *HHIPL1* | -3.241369969 | 0.03254072 |
| ENSG00000101955 | *SRPX* | -3.244198566 | 0.024646219 |
| ENSG00000017483 | *SLC38A5* | -3.247644713 | 0.002227462 |
| ENSG00000220201 | *ZGLP1* | -3.248172715 | 0.028383655 |
| ENSG00000214140 | *PRCD* | -3.25114314 | 0.014192844 |
| ENSG00000274925 | *CTD-2547G23.4* | -3.251693868 | 0.022029695 |
| ENSG00000174125 | *TLR1* | -3.258239686 | 0.004110138 |
| ENSG00000160111 | *CPAMD8* | -3.265911752 | 0.021282782 |
| ENSG00000164136 | *IL15* | -3.2659609 | 0.015122739 |
| ENSG00000187800 | *PEAR1* | -3.26980311 | 0.002014584 |
| ENSG00000251661 | *RP11-326C3.11* | -3.273067336 | 0.013391701 |
| ENSG00000151617 | *EDNRA* | -3.273403075 | 0.001581531 |
| ENSG00000233622 | *CYP2T1P* | -3.275896958 | 0.049179369 |
| ENSG00000184304 | *PRKD1* | -3.278179421 | 0.00045097 |
| ENSG00000128606 | *LRRC17* | -3.282832143 | 0.012744538 |
| ENSG00000226833 | *AC097724.3* | -3.284546827 | 0.016066819 |
| ENSG00000169116 | *PARM1* | -3.28828947 | 0.016073011 |
| ENSG00000120693 | *SMAD9* | -3.292174366 | 0.00250893 |
| ENSG00000113389 | *NPR3* | -3.292540228 | 0.03442132 |
| ENSG00000229619 | *MBNL1-AS1* | -3.294760791 | 0.011056039 |
| ENSG00000079337 | *RAPGEF3* | -3.295168396 | 3.07E-07 |
| ENSG00000129465 | *RIPK3* | -3.296437973 | 0.010348244 |
| ENSG00000119686 | *FLVCR2* | -3.306808978 | 0.001472665 |
| ENSG00000196876 | *SCN8A* | -3.308399161 | 0.048101885 |
| ENSG00000167034 | *NKX3-1* | -3.312773757 | 0.001182805 |
| ENSG00000176907 | *C8orf4* | -3.313276758 | 0.002213395 |
| ENSG00000187824 | *TMEM220* | -3.315217181 | 0.011170968 |
| ENSG00000168016 | *TRANK1* | -3.316024119 | 0.001253859 |
| ENSG00000259658 | *RP11-89K11.1* | -3.31755899 | 0.018462631 |
| ENSG00000188677 | *PARVB* | -3.319078931 | 0.000206982 |
| ENSG00000180448 | *HMHA1* | -3.319393632 | 0.008975469 |
| ENSG00000186462 | *NAP1L2* | -3.32319708 | 0.019435843 |
| ENSG00000129596 | *CDO1* | -3.323632208 | 0.003738043 |
| ENSG00000231721 | *LINC-PINT* | -3.327249829 | 3.71E-06 |
| ENSG00000223756 | *TSSC2* | -3.329448862 | 0.016910617 |
| ENSG00000130222 | *GADD45G* | -3.335985862 | 0.026413703 |
| ENSG00000158715 | *SLC45A3* | -3.339149284 | 0.018522536 |
| ENSG00000173546 | *CSPG4* | -3.343788965 | 0.00151139 |
| ENSG00000107611 | *CUBN* | -3.356367476 | 0.003950876 |
| ENSG00000172771 | *EFCAB12* | -3.357439994 | 0.037072899 |
| ENSG00000164742 | *ADCY1* | -3.364792237 | 0.023512749 |
| ENSG00000160460 | *SPTBN4* | -3.367362033 | 0.003471664 |
| ENSG00000196739 | *COL27A1* | -3.368137914 | 0.000963359 |
| ENSG00000204397 | *CARD16* | -3.3701155 | 0.013711941 |
| ENSG00000169439 | *SDC2* | -3.375567482 | 3.86E-05 |
| ENSG00000236333 | *TRHDE-AS1* | -3.376647041 | 0.023791393 |
| ENSG00000235568 | *NFAM1* | -3.379571163 | 0.008834392 |
| ENSG00000213071 | *LPAL2* | -3.384996649 | 0.023362112 |
| ENSG00000134539 | *KLRD1* | -3.386098619 | 0.007401276 |
| ENSG00000126562 | *WNK4* | -3.386978418 | 0.025171578 |
| ENSG00000127324 | *TSPAN8* | -3.38733673 | 0.006287628 |
| ENSG00000182963 | *GJC1* | -3.390421953 | 0.00010554 |
| ENSG00000231731 | *AC010976.2* | -3.395599866 | 0.047655968 |
| ENSG00000273373 | *RP5-1074L1.4* | -3.396667701 | 0.001805064 |
| ENSG00000183018 | *SPNS2* | -3.399545214 | 0.006431441 |
| ENSG00000157554 | *ERG* | -3.404746986 | 2.49E-05 |
| ENSG00000160469 | *BRSK1* | -3.408182364 | 0.004921851 |
| ENSG00000129521 | *EGLN3* | -3.415389596 | 0.001173296 |
| ENSG00000197635 | *DPP4* | -3.420752995 | 0.014914051 |
| ENSG00000269821 | *KCNQ1OT1* | -3.428829673 | 0.001551312 |
| ENSG00000251595 | *ABCA11P* | -3.430894492 | 0.04106289 |
| ENSG00000197355 | *UAP1L1* | -3.436077556 | 7.43E-05 |
| ENSG00000138685 | *FGF2* | -3.443892724 | 0.000755256 |
| ENSG00000278376 | *RP11-158I9.8* | -3.448446769 | 0.007927559 |
| ENSG00000137573 | *SULF1* | -3.452268479 | 0.000716512 |
| ENSG00000159588 | *CCDC17* | -3.453345048 | 0.009122622 |
| ENSG00000261126 | *RP11-795F19.1* | -3.461027109 | 0.043136598 |
| ENSG00000172183 | *ISG20* | -3.46508978 | 0.007927559 |
| ENSG00000163462 | *TRIM46* | -3.467441567 | 0.006429123 |
| ENSG00000123358 | *NR4A1* | -3.470245449 | 0.012503797 |
| ENSG00000066629 | *EML1* | -3.471754713 | 6.33E-05 |
| ENSG00000147255 | *IGSF1* | -3.474577229 | 0.045614113 |
| ENSG00000075073 | *TACR2* | -3.476985345 | 0.027931282 |
| ENSG00000088538 | *DOCK3* | -3.477141248 | 0.000467376 |
| ENSG00000159899 | *NPR2* | -3.477341222 | 3.95E-06 |
| ENSG00000111644 | *ACRBP* | -3.479931956 | 0.026602528 |
| ENSG00000146094 | *DOK3* | -3.48047498 | 0.009503527 |
| ENSG00000180096 | *1-Sep* | -3.482477727 | 0.006748184 |
| ENSG00000270012 | *LL0XNC01-7P3.1* | -3.48450094 | 0.012349634 |
| ENSG00000047648 | *ARHGAP6* | -3.495131446 | 0.002964677 |
| ENSG00000133321 | *RARRES3* | -3.495922934 | 0.000817989 |
| ENSG00000176490 | *DIRAS1* | -3.498434694 | 0.000313071 |
| ENSG00000109846 | *CRYAB* | -3.501744632 | 0.019746194 |
| ENSG00000261326 | *LINC01355* | -3.503262153 | 0.007732923 |
| ENSG00000171951 | *SCG2* | -3.505055588 | 0.039317735 |
| ENSG00000142227 | *EMP3* | -3.510039654 | 0.000512564 |
| ENSG00000144488 | *ESPNL* | -3.511516222 | 0.027471971 |
| ENSG00000135437 | *RDH5* | -3.516963593 | 0.014429921 |
| ENSG00000196209 | *SIRPB2* | -3.520268435 | 0.021190403 |
| ENSG00000103056 | *SMPD3* | -3.521680864 | 0.000845267 |
| ENSG00000056558 | *TRAF1* | -3.526617951 | 0.000130352 |
| ENSG00000033122 | *LRRC7* | -3.529350526 | 0.049433619 |
| ENSG00000088882 | *CPXM1* | -3.539935882 | 5.22E-07 |
| ENSG00000134627 | *PIWIL4* | -3.540651665 | 0.002221735 |
| ENSG00000132718 | *SYT11* | -3.546326022 | 0.000204214 |
| ENSG00000082397 | *EPB41L3* | -3.547745252 | 0.002878413 |
| ENSG00000005249 | *PRKAR2B* | -3.547966982 | 0.011677886 |
| ENSG00000122035 | *RASL11A* | -3.549410139 | 0.000381644 |
| ENSG00000075461 | *CACNG4* | -3.549456932 | 0.032762112 |
| ENSG00000221890 | *NPTXR* | -3.549904785 | 0.00134569 |
| ENSG00000165121 | *RP11-213G2.3* | -3.558057992 | 0.000101271 |
| ENSG00000154556 | *SORBS2* | -3.560268973 | 0.000210485 |
| ENSG00000127311 | *HELB* | -3.560374205 | 0.010476876 |
| ENSG00000189420 | *ZFP92* | -3.569578703 | 0.031221457 |
| ENSG00000245105 | *A2M-AS1* | -3.574999119 | 0.018174733 |
| ENSG00000085514 | *PILRA* | -3.577291701 | 0.000614359 |
| ENSG00000239697 | *TNFSF12* | -3.578339175 | 3.06E-05 |
| ENSG00000113555 | *PCDH12* | -3.58441842 | 0.000155039 |
| ENSG00000119699 | *TGFB3* | -3.585210526 | 6.67E-10 |
| ENSG00000137124 | *ALDH1B1* | -3.585909846 | 6.37E-05 |
| ENSG00000187942 | *LDLRAD2* | -3.587652105 | 0.02160946 |
| ENSG00000202198 | *RN7SK* | -3.589370656 | 0.002244593 |
| ENSG00000197599 | *CCDC154* | -3.591707387 | 0.04656717 |
| ENSG00000019485 | *PRDM11* | -3.596921767 | 9.57E-05 |
| ENSG00000137496 | *IL18BP* | -3.597867533 | 0.000309328 |
| ENSG00000178821 | *TMEM52* | -3.604872781 | 0.023863721 |
| ENSG00000205085 | *FAM71F2* | -3.611721861 | 0.023687513 |
| ENSG00000235501 | *RP4-639F20.1* | -3.613432775 | 0.000444667 |
| ENSG00000225614 | *ZNF469* | -3.615363474 | 0.040369054 |
| ENSG00000108176 | *DNAJC12* | -3.616291365 | 0.045070083 |
| ENSG00000198189 | *HSD17B11* | -3.625928302 | 3.64E-06 |
| ENSG00000136883 | *KIF12* | -3.627283609 | 0.018396381 |
| ENSG00000091656 | *ZFHX4* | -3.627383334 | 0.001569131 |
| ENSG00000170500 | *LONRF2* | -3.628578393 | 9.85E-05 |
| ENSG00000038427 | *VCAN* | -3.628987875 | 0.029825615 |
| ENSG00000111799 | *COL12A1* | -3.631140256 | 0.015849308 |
| ENSG00000116157 | *GPX7* | -3.636400388 | 0.001467177 |
| ENSG00000144857 | *BOC* | -3.639164376 | 3.49E-05 |
| ENSG00000106078 | *COBL* | -3.640436529 | 3.93E-06 |
| ENSG00000117425 | *PTCH2* | -3.642873296 | 0.014054514 |
| ENSG00000136999 | *NOV* | -3.644195993 | 0.014259145 |
| ENSG00000062524 | *LTK* | -3.649482047 | 0.027866274 |
| ENSG00000163531 | *NFASC* | -3.652413067 | 1.71E-05 |
| ENSG00000143341 | *HMCN1* | -3.66170473 | 0.019804602 |
| ENSG00000132329 | *RAMP1* | -3.664452759 | 9.20E-05 |
| ENSG00000172159 | *FRMD3* | -3.666851196 | 0.02404956 |
| ENSG00000196338 | *NLGN3* | -3.667837776 | 0.000419115 |
| ENSG00000174672 | *BRSK2* | -3.678166994 | 0.04560262 |
| ENSG00000079308 | *TNS1* | -3.678447263 | 0.000269009 |
| ENSG00000104953 | *TLE6* | -3.685321273 | 0.002427385 |
| ENSG00000104324 | *CPQ* | -3.688725768 | 4.37E-05 |
| ENSG00000129749 | *CHRNA10* | -3.68954077 | 0.016488129 |
| ENSG00000254535 | *PABPC4L* | -3.696784432 | 0.014587476 |
| ENSG00000165072 | *MAMDC2* | -3.706573413 | 0.003957096 |
| ENSG00000132386 | *SERPINF1* | -3.71413345 | 7.68E-06 |
| ENSG00000114450 | *GNB4* | -3.717504449 | 0.001800637 |
| ENSG00000261496 | *RP13-514E23.1* | -3.737424669 | 0.03883485 |
| ENSG00000134769 | *DTNA* | -3.738678544 | 0.036539555 |
| ENSG00000137672 | *TRPC6* | -3.739956376 | 0.000227685 |
| ENSG00000078596 | *ITM2A* | -3.748315813 | 0.001071594 |
| ENSG00000215452 | *ZNF663P* | -3.758991731 | 0.003266585 |
| ENSG00000137872 | *SEMA6D* | -3.760462744 | 0.001983501 |
| ENSG00000153814 | *JAZF1* | -3.761723806 | 0.000114408 |
| ENSG00000267082 | *CTC-510F12.2* | -3.763282828 | 0.019461535 |
| ENSG00000111348 | *ARHGDIB* | -3.765867673 | 0.001154402 |
| ENSG00000272078 | *RP4-734G22.3* | -3.766723112 | 0.045318804 |
| ENSG00000087076 | *HSD17B14* | -3.766731763 | 1.99E-06 |
| ENSG00000197594 | *ENPP1* | -3.767592175 | 0.000121396 |
| ENSG00000176485 | *PLA2G16* | -3.769088416 | 0.002731065 |
| ENSG00000180447 | *GAS1* | -3.769262382 | 0.000909496 |
| ENSG00000171476 | *HOPX* | -3.772616782 | 0.000366697 |
| ENSG00000261490 | *RP11-448G15.3* | -3.773829722 | 0.00019669 |
| ENSG00000134326 | *CMPK2* | -3.776279596 | 0.008661269 |
| ENSG00000133067 | *LGR6* | -3.776320389 | 0.044652084 |
| ENSG00000163995 | *ABLIM2* | -3.782232558 | 0.000338346 |
| ENSG00000113594 | *LIFR* | -3.784868411 | 0.003017404 |
| ENSG00000111077 | *TNS2* | -3.785908362 | 2.94E-07 |
| ENSG00000196167 | *COLCA1* | -3.786348885 | 0.008433446 |
| ENSG00000182621 | *PLCB1* | -3.788790265 | 0.00303129 |
| ENSG00000119138 | *KLF9* | -3.789184549 | 0.000240551 |
| ENSG00000108960 | *MMD* | -3.789697918 | 0.006768476 |
| ENSG00000122574 | *WIPF3* | -3.795211321 | 1.45E-06 |
| ENSG00000281650 | *CH17-64J14.5* | -3.797925873 | 0.017457741 |
| ENSG00000069702 | *TGFBR3* | -3.80097728 | 0.002060219 |
| ENSG00000142959 | *BEST4* | -3.805900396 | 0.038581636 |
| ENSG00000073910 | *FRY* | -3.811304514 | 0.002861076 |
| ENSG00000008226 | *DLEC1* | -3.827813565 | 0.013049072 |
| ENSG00000086289 | *EPDR1* | -3.833787624 | 7.59E-05 |
| ENSG00000185339 | *TCN2* | -3.834239665 | 1.73E-05 |
| ENSG00000129675 | *ARHGEF6* | -3.838669676 | 1.43E-06 |
| ENSG00000269911 | *RP1-172N19.5* | -3.83891962 | 0.008879591 |
| ENSG00000087085 | *ACHE* | -3.838959099 | 0.005971438 |
| ENSG00000125730 | *C3* | -3.840197873 | 0.004779272 |
| ENSG00000183833 | *MAATS1* | -3.841221545 | 5.12E-05 |
| ENSG00000224934 | *RP11-441O15.3* | -3.841286623 | 0.008017371 |
| ENSG00000162241 | *SLC25A45* | -3.841442742 | 0.001226395 |
| ENSG00000140993 | *TIGD7* | -3.843848458 | 0.004245152 |
| ENSG00000099994 | *SUSD2* | -3.84674526 | 0.008370645 |
| ENSG00000171119 | *NRTN* | -3.847016524 | 0.013171288 |
| ENSG00000170074 | *FAM153A* | -3.849635816 | 4.79E-05 |
| ENSG00000232611 | *RP11-1114A5.4* | -3.853229154 | 0.001538713 |
| ENSG00000175745 | *NR2F1* | -3.856074421 | 0.002013386 |
| ENSG00000277449 | *CEBPB-AS1* | -3.856835364 | 0.011598271 |
| ENSG00000147027 | *TMEM47* | -3.857909519 | 0.00067051 |
| ENSG00000225032 | *RP11-228B15.4* | -3.862241482 | 0.009934236 |
| ENSG00000126561 | *STAT5A* | -3.862837254 | 4.80E-07 |
| ENSG00000184497 | *TMEM255B* | -3.867691604 | 0.002540868 |
| ENSG00000176438 | *SYNE3* | -3.868098623 | 0.000183523 |
| ENSG00000198932 | *GPRASP1* | -3.870723677 | 5.24E-08 |
| ENSG00000064201 | *TSPAN32* | -3.872376361 | 0.010302703 |
| ENSG00000126709 | *IFI6* | -3.875083425 | 3.43E-05 |
| ENSG00000113140 | *SPARC* | -3.878431123 | 0.000143856 |
| ENSG00000171812 | *COL8A2* | -3.882124715 | 0.002407482 |
| ENSG00000263812 | *LINC00908* | -3.882709582 | 0.00563978 |
| ENSG00000124713 | *GNMT* | -3.887346886 | 0.011756563 |
| ENSG00000274137 | *MYOM2* | -3.900129705 | 0.032552125 |
| ENSG00000171502 | *COL24A1* | -3.907200918 | 0.045070083 |
| ENSG00000197816 | *CCDC180* | -3.908359807 | 0.040248175 |
| ENSG00000144152 | *FBLN7* | -3.913745737 | 0.002453092 |
| ENSG00000048740 | *CELF2* | -3.921366415 | 1.24E-07 |
| ENSG00000133246 | *PRAM1* | -3.925596336 | 0.001754901 |
| ENSG00000132530 | *XAF1* | -3.926558084 | 0.01429074 |
| ENSG00000136859 | *ANGPTL2* | -3.927169378 | 6.55E-10 |
| ENSG00000168952 | *STXBP6* | -3.928550296 | 0.003595279 |
| ENSG00000181790 | *ADGRB1* | -3.928880155 | 0.020133725 |
| ENSG00000118292 | *C1orf54* | -3.933699703 | 8.73E-05 |
| ENSG00000162461 | *SLC25A34* | -3.939306328 | 0.009893307 |
| ENSG00000186998 | *EMID1* | -3.950602426 | 6.87E-05 |
| ENSG00000020633 | *RUNX3* | -3.95581809 | 0.013773781 |
| ENSG00000163453 | *IGFBP7* | -3.96137249 | 2.91E-07 |
| ENSG00000007174 | *DNAH9* | -3.962039129 | 0.024073775 |
| ENSG00000134363 | *FST* | -3.9639606 | 0.013390633 |
| ENSG00000183486 | *MX2* | -3.970910833 | 0.003796725 |
| ENSG00000242732 | *RGAG4* | -3.972297747 | 0.001536748 |
| ENSG00000174804 | *FZD4* | -3.974094856 | 1.12E-08 |
| ENSG00000120049 | *KCNIP2* | -3.977557577 | 0.043177196 |
| ENSG00000184226 | *PCDH9* | -3.980850553 | 0.006369535 |
| ENSG00000260549 | *MT1L* | -3.983309956 | 1.95E-05 |
| ENSG00000197181 | *PIWIL2* | -3.986518359 | 0.032736668 |
| ENSG00000122188 | *LAX1* | -3.986962931 | 0.046220654 |
| ENSG00000135596 | *MICAL1* | -3.988012527 | 2.70E-05 |
| ENSG00000183615 | *FAM167B* | -3.989742528 | 6.60E-06 |
| ENSG00000152931 | *PART1* | -3.992364026 | 0.000445996 |
| ENSG00000108622 | *ICAM2* | -3.992962956 | 0.000360576 |
| ENSG00000241839 | *PLEKHO2* | -3.997737867 | 2.37E-07 |
| ENSG00000260805 | *RP11-61J19.5* | -4.003049851 | 0.014194126 |
| ENSG00000144724 | *PTPRG* | -4.004584039 | 0.001197506 |
| ENSG00000276148 | *RP11-278C7.4* | -4.005864812 | 0.034944144 |
| ENSG00000137558 | *PI15* | -4.007622042 | 0.000238512 |
| ENSG00000166669 | *ATF7IP2* | -4.0103387 | 5.09E-06 |
| ENSG00000049089 | *COL9A2* | -4.010553125 | 0.003690261 |
| ENSG00000255248 | *RP11-166D19.1* | -4.021892944 | 0.002379804 |
| ENSG00000164691 | *TAGAP* | -4.024764398 | 0.006459293 |
| ENSG00000129244 | *ATP1B2* | -4.029005438 | 0.000440576 |
| ENSG00000223532 | *HLA-B* | -4.036477642 | 0.000180345 |
| ENSG00000170955 | *PRKCDBP* | -4.049423085 | 4.70E-06 |
| ENSG00000136717 | *BIN1* | -4.056633498 | 3.66E-09 |
| ENSG00000102802 | *MEDAG* | -4.059660373 | 0.024097315 |
| ENSG00000168062 | *BATF2* | -4.060020432 | 0.003679626 |
| ENSG00000139278 | *GLIPR1* | -4.062832336 | 0.002677746 |
| ENSG00000267280 | *TBX2-AS1* | -4.06441986 | 0.005825518 |
| ENSG00000205426 | *KRT81* | -4.066025594 | 0.01608116 |
| ENSG00000235790 | *RP11-73M7.6* | -4.066476296 | 0.026031245 |
| ENSG00000138639 | *ARHGAP24* | -4.070371685 | 0.000375092 |
| ENSG00000168497 | *SDPR* | -4.07118584 | 0.00031313 |
| ENSG00000105639 | *JAK3* | -4.075680182 | 0.000925318 |
| ENSG00000110900 | *TSPAN11* | -4.076852515 | 0.005935371 |
| ENSG00000096060 | *FKBP5* | -4.094368375 | 0.00134909 |
| ENSG00000171291 | *ZNF439* | -4.096902914 | 4.28E-07 |
| ENSG00000073712 | *FERMT2* | -4.097178852 | 1.18E-05 |
| ENSG00000115325 | *DOK1* | -4.098159666 | 6.01E-05 |
| ENSG00000167992 | *VWCE* | -4.100385756 | 0.00174008 |
| ENSG00000049192 | *ADAMTS6* | -4.107197738 | 0.027471971 |
| ENSG00000121797 | *CCRL2* | -4.113130298 | 0.042894528 |
| ENSG00000184313 | *MROH7* | -4.114376784 | 0.009800009 |
| ENSG00000262655 | *SPON1* | -4.122857319 | 3.77E-06 |
| ENSG00000267934 | *CTB-176F20.3* | -4.124871564 | 0.047153132 |
| ENSG00000189409 | *MMP23B* | -4.135021576 | 0.011590775 |
| ENSG00000130208 | *APOC1* | -4.137299904 | 0.00011398 |
| ENSG00000274220 | *RP11-77K12.9* | -4.146745603 | 0.047555632 |
| ENSG00000166920 | *C15orf48* | -4.148279248 | 0.006048083 |
| ENSG00000224383 | *PRR29* | -4.150180864 | 0.014089914 |
| ENSG00000140682 | *TGFB1I1* | -4.151134204 | 3.01E-09 |
| ENSG00000185527 | *PDE6G* | -4.151821388 | 0.029857235 |
| ENSG00000064692 | *SNCAIP* | -4.16337588 | 0.003097176 |
| ENSG00000089041 | *P2RX7* | -4.164294063 | 1.98E-05 |
| ENSG00000175315 | *CST6* | -4.167542395 | 0.029777753 |
| ENSG00000168961 | *LGALS9* | -4.170819624 | 0.013747273 |
| ENSG00000004399 | *PLXND1* | -4.171383327 | 1.00E-08 |
| ENSG00000182853 | *VMO1* | -4.181292232 | 0.003786712 |
| ENSG00000172247 | *C1QTNF4* | -4.188806903 | 0.00425442 |
| ENSG00000165300 | *SLITRK5* | -4.200449219 | 0.000303172 |
| ENSG00000107551 | *RASSF4* | -4.20208389 | 3.98E-08 |
| ENSG00000188385 | *JAKMIP3* | -4.207945252 | 0.020024791 |
| ENSG00000178882 | *FAM101A* | -4.213031779 | 0.030080833 |
| ENSG00000260077 | *RP11-254F7.2* | -4.21331657 | 0.000282184 |
| ENSG00000089692 | *LAG3* | -4.216796669 | 0.001974581 |
| ENSG00000132821 | *VSTM2L* | -4.22173363 | 0.004054624 |
| ENSG00000260807 | *RP11-161M6.2* | -4.224931573 | 0.000524327 |
| ENSG00000079101 | *CLUL1* | -4.225911261 | 0.014531653 |
| ENSG00000023902 | *PLEKHO1* | -4.231722047 | 6.23E-06 |
| ENSG00000151789 | *ZNF385D* | -4.239735188 | 4.42E-05 |
| ENSG00000112394 | *SLC16A10* | -4.262694053 | 0.008708762 |
| ENSG00000183873 | *SCN5A* | -4.267629013 | 0.028857057 |
| ENSG00000260230 | *FRRS1L* | -4.267797278 | 0.000498774 |
| ENSG00000106069 | *CHN2* | -4.270222358 | 0.006417121 |
| ENSG00000198417 | *MT1F* | -4.271645925 | 1.31E-05 |
| ENSG00000248019 | *FAM13A-AS1* | -4.271702249 | 0.003041341 |
| ENSG00000188906 | *LRRK2* | -4.272057025 | 2.99E-06 |
| ENSG00000163131 | *CTSS* | -4.277434384 | 1.14E-05 |
| ENSG00000121904 | *CSMD2* | -4.282908494 | 0.011966541 |
| ENSG00000131097 | *HIGD1B* | -4.288899476 | 0.017769695 |
| ENSG00000263990 | *CTC-542B22.2* | -4.291007239 | 0.040009441 |
| ENSG00000272668 | *RP11-190A12.8* | -4.295880059 | 0.013391701 |
| ENSG00000182667 | *NTM* | -4.296151382 | 0.047159724 |
| ENSG00000104213 | *PDGFRL* | -4.304400203 | 0.00821207 |
| ENSG00000158887 | *MPZ* | -4.306425848 | 0.000555501 |
| ENSG00000153956 | *CACNA2D1* | -4.31156267 | 0.001077206 |
| ENSG00000163132 | *MSX1* | -4.319604992 | 0.005484061 |
| ENSG00000144229 | *THSD7B* | -4.322200083 | 0.038639713 |
| ENSG00000167549 | *CORO6* | -4.323472754 | 3.00E-06 |
| ENSG00000143387 | *CTSK* | -4.324591503 | 1.53E-07 |
| ENSG00000224608 | *HLA-B* | -4.329735707 | 0.000402976 |
| ENSG00000244486 | *SCARF2* | -4.34354855 | 1.54E-07 |
| ENSG00000272037 | *KB-431C1.5* | -4.346420233 | 0.041988052 |
| ENSG00000243696 | *RP5-966M1.6* | -4.348725051 | 0.000801969 |
| ENSG00000164946 | *FREM1* | -4.349761836 | 0.000755607 |
| ENSG00000156265 | *MAP3K7CL* | -4.34993305 | 0.001077206 |
| ENSG00000241170 | *RP11-147I3.1* | -4.35502798 | 0.003756935 |
| ENSG00000112299 | *VNN1* | -4.355713982 | 0.049254445 |
| ENSG00000253829 | *RP11-723D22.3* | -4.361971261 | 0.039752928 |
| ENSG00000248596 | *RP11-844P9.2* | -4.36490405 | 0.020325054 |
| ENSG00000154928 | *EPHB1* | -4.373466422 | 0.003001484 |
| ENSG00000100599 | *RIN3* | -4.374066064 | 2.99E-07 |
| ENSG00000174807 | *CD248* | -4.37667148 | 0.002897018 |
| ENSG00000130413 | *STK33* | -4.378738321 | 0.000221633 |
| ENSG00000244945 | *RP11-1379J22.2* | -4.382452766 | 0.011104536 |
| ENSG00000169085 | *C8orf46* | -4.383907772 | 4.12E-05 |
| ENSG00000008311 | *AASS* | -4.385553538 | 2.91E-06 |
| ENSG00000179314 | *WSCD1* | -4.386006461 | 0.040248175 |
| ENSG00000196793 | *ZNF239* | -4.392357146 | 0.002244593 |
| ENSG00000159761 | *C16orf86* | -4.393275137 | 0.000473219 |
| ENSG00000023445 | *BIRC3* | -4.396648118 | 1.44E-05 |
| ENSG00000147166 | *ITGB1BP2* | -4.398486706 | 0.004952715 |
| ENSG00000092929 | *UNC13D* | -4.413759677 | 8.67E-06 |
| ENSG00000152270 | *PDE3B* | -4.417027237 | 6.17E-05 |
| ENSG00000105643 | *ARRDC2* | -4.417901164 | 1.81E-10 |
| ENSG00000158163 | *DZIP1L* | -4.444606426 | 4.15E-06 |
| ENSG00000169436 | *COL22A1* | -4.448313325 | 0.016762442 |
| ENSG00000263400 | *TMEM220-AS1* | -4.448438232 | 0.046443496 |
| ENSG00000138028 | *CGREF1* | -4.45243095 | 0.005696103 |
| ENSG00000110777 | *POU2AF1* | -4.454365049 | 0.008974052 |
| ENSG00000145147 | *SLIT2* | -4.469163229 | 1.51E-05 |
| ENSG00000254838 | *GVINP1* | -4.476295164 | 5.77E-05 |
| ENSG00000160145 | *KALRN* | -4.477724701 | 0.000279111 |
| ENSG00000137727 | *ARHGAP20* | -4.48402865 | 8.38E-06 |
| ENSG00000259969 | *RP11-999E24.3* | -4.486160252 | 0.03188702 |
| ENSG00000036530 | *CYP46A1* | -4.486169866 | 0.008582812 |
| ENSG00000004799 | *PDK4* | -4.49073139 | 0.012551122 |
| ENSG00000122986 | *HVCN1* | -4.491536324 | 4.50E-05 |
| ENSG00000278309 | *RP11-102K13.5* | -4.494831945 | 0.011466988 |
| ENSG00000126882 | *FAM78A* | -4.496023845 | 6.22E-05 |
| ENSG00000232807 | *RP11-536K7.3* | -4.503054757 | 0.00863005 |
| ENSG00000107295 | *SH3GL2* | -4.50659277 | 0.035540687 |
| ENSG00000113248 | *PCDHB15* | -4.510603655 | 0.000126217 |
| ENSG00000153071 | *DAB2* | -4.512089079 | 0.012501304 |
| ENSG00000108387 | *4-Sep* | -4.51234953 | 0.001827089 |
| ENSG00000069535 | *MAOB* | -4.520867889 | 4.69E-06 |
| ENSG00000166924 | *NYAP1* | -4.522338814 | 0.005838773 |
| ENSG00000280332 | *CTD-2013N17.6* | -4.529063142 | 0.009466479 |
| ENSG00000225691 | *HLA-C* | -4.533772017 | 0.001348416 |
| ENSG00000112182 | *BACH2* | -4.544405828 | 0.000367039 |
| ENSG00000204677 | *FAM153C* | -4.548324375 | 0.00095686 |
| ENSG00000184922 | *FMNL1* | -4.551296542 | 4.78E-05 |
| ENSG00000198300 | *PEG3* | -4.552277331 | 0.002908918 |
| ENSG00000171791 | *BCL2* | -4.55572003 | 9.82E-09 |
| ENSG00000133048 | *CHI3L1* | -4.556098689 | 0.000215691 |
| ENSG00000158528 | *PPP1R9A* | -4.559379719 | 3.17E-05 |
| ENSG00000151692 | *RNF144A* | -4.566649242 | 3.65E-06 |
| ENSG00000182511 | *FES* | -4.568038617 | 1.47E-08 |
| ENSG00000166676 | *TVP23A* | -4.569605071 | 3.28E-05 |
| ENSG00000058866 | *DGKG* | -4.573468231 | 1.86E-05 |
| ENSG00000272501 | *XXbac-BPG299F13.17* | -4.576160911 | 0.00838449 |
| ENSG00000188763 | *FZD9* | -4.579918751 | 0.00675925 |
| ENSG00000101210 | *EEF1A2* | -4.580428456 | 0.002955631 |
| ENSG00000162373 | *BEND5* | -4.583339674 | 0.000966239 |
| ENSG00000205181 | *LINC00654* | -4.588525115 | 0.007538179 |
| ENSG00000135439 | *AGAP2* | -4.598384386 | 0.001628393 |
| ENSG00000251143 | *RP11-849H4.4* | -4.599621427 | 0.016084337 |
| ENSG00000172794 | *RAB37* | -4.600757125 | 0.000126217 |
| ENSG00000280339 | *RP11-736K20.4* | -4.603711841 | 0.02955786 |
| ENSG00000260005 | *AC027601.1* | -4.612508272 | 0.032082095 |
| ENSG00000197142 | *ACSL5* | -4.613072125 | 6.04E-05 |
| ENSG00000215386 | *MIR99AHG* | -4.617718766 | 5.83E-06 |
| ENSG00000077157 | *PPP1R12B* | -4.628931845 | 1.13E-14 |
| ENSG00000153404 | *PLEKHG4B* | -4.629337184 | 0.009675742 |
| ENSG00000157734 | *SNX22* | -4.634997908 | 0.00116 |
| ENSG00000246859 | *STARD4-AS1* | -4.635202804 | 0.010976991 |
| ENSG00000259976 | *RP11-553L6.5* | -4.635993286 | 6.83E-08 |
| ENSG00000122254 | *HS3ST2* | -4.636422768 | 0.012679437 |
| ENSG00000186517 | *ARHGAP30* | -4.638322407 | 0.000221849 |
| ENSG00000119508 | *NR4A3* | -4.644807859 | 8.28E-06 |
| ENSG00000141431 | *ASXL3* | -4.650260793 | 0.000911604 |
| ENSG00000111186 | *WNT5B* | -4.653343197 | 2.62E-11 |
| ENSG00000177875 | *CCDC184* | -4.656572414 | 0.017552533 |
| ENSG00000134121 | *CHL1* | -4.663311975 | 0.009832403 |
| ENSG00000137726 | *FXYD6* | -4.665944792 | 0.000122564 |
| ENSG00000204262 | *COL5A2* | -4.668103631 | 1.23E-06 |
| ENSG00000106991 | *ENG* | -4.668331508 | 1.53E-12 |
| ENSG00000154822 | *PLCL2* | -4.670116273 | 0.002460322 |
| ENSG00000129993 | *CBFA2T3* | -4.671071833 | 0.002052529 |
| ENSG00000160191 | *PDE9A* | -4.673838616 | 9.35E-05 |
| ENSG00000220563 | *PKMP3* | -4.67934335 | 0.014680206 |
| ENSG00000145428 | *RNF175* | -4.682454502 | 0.001128664 |
| ENSG00000272505 | *RP11-981G7.6* | -4.684872391 | 0.023247434 |
| ENSG00000169245 | *CXCL10* | -4.686344929 | 0.00678607 |
| ENSG00000100311 | *PDGFB* | -4.691359202 | 7.36E-06 |
| ENSG00000115318 | *LOXL3* | -4.693464882 | 2.22E-05 |
| ENSG00000261616 | *RP11-6O2.3* | -4.702625718 | 0.002034632 |
| ENSG00000249626 | *RP11-496H1.1* | -4.705055582 | 0.025284864 |
| ENSG00000267102 | *RP11-686D22.7* | -4.707377503 | 0.026140292 |
| ENSG00000168329 | *CX3CR1* | -4.709578185 | 0.042371467 |
| ENSG00000161055 | *SCGB3A1* | -4.723213854 | 0.003548018 |
| ENSG00000197467 | *COL13A1* | -4.729228513 | 1.37E-06 |
| ENSG00000139187 | *KLRG1* | -4.733223734 | 0.004740105 |
| ENSG00000165617 | *DACT1* | -4.734487633 | 6.44E-07 |
| ENSG00000173110 | *HSPA6* | -4.740625008 | 0.000762725 |
| ENSG00000184343 | *SRPK3* | -4.745235857 | 9.31E-05 |
| ENSG00000249307 | *LINC01088* | -4.746520651 | 0.011618834 |
| ENSG00000159640 | *ACE* | -4.747242781 | 0.000360576 |
| ENSG00000146197 | *SCUBE3* | -4.748751551 | 6.17E-07 |
| ENSG00000048052 | *HDAC9* | -4.757748136 | 1.75E-06 |
| ENSG00000162415 | *ZSWIM5* | -4.758076736 | 0.000143536 |
| ENSG00000169894 | *MUC3A* | -4.758237255 | 0.002592392 |
| ENSG00000128284 | *APOL3* | -4.758337784 | 1.49E-08 |
| ENSG00000170458 | *CD14* | -4.758532142 | 1.60E-06 |
| ENSG00000023171 | *GRAMD1B* | -4.764815531 | 0.031959226 |
| ENSG00000170837 | *GPR27* | -4.765218782 | 0.000614359 |
| ENSG00000224713 | *AC025165.8* | -4.767551658 | 0.003679626 |
| ENSG00000130635 | *COL5A1* | -4.770679713 | 1.44E-06 |
| ENSG00000277496 | *RP11-93B14.9* | -4.777405518 | 0.01044746 |
| ENSG00000133083 | *DCLK1* | -4.777627344 | 2.93E-05 |
| ENSG00000166963 | *MAP1A* | -4.780601388 | 2.56E-05 |
| ENSG00000166257 | *SCN3B* | -4.782720698 | 0.008996591 |
| ENSG00000134321 | *RSAD2* | -4.784160519 | 0.00125928 |
| ENSG00000223396 | *RP11-134G8.5* | -4.794132396 | 0.025884023 |
| ENSG00000145861 | *C1QTNF2* | -4.801503451 | 0.004232362 |
| ENSG00000235475 | *LINC01372* | -4.810181051 | 0.028941896 |
| ENSG00000112149 | *CD83* | -4.812931485 | 1.31E-06 |
| ENSG00000142494 | *SLC47A1* | -4.813572644 | 2.77E-05 |
| ENSG00000100079 | *LGALS2* | -4.82743108 | 0.016406207 |
| ENSG00000113296 | *THBS4* | -4.828741494 | 1.03E-05 |
| ENSG00000240771 | *ARHGEF25* | -4.829274537 | 5.41E-10 |
| ENSG00000106333 | *PCOLCE* | -4.833666199 | 1.27E-12 |
| ENSG00000276698 | *RP11-468E2.10* | -4.83428947 | 0.019317389 |
| ENSG00000230438 | *SERPINB9P1* | -4.841172618 | 0.013009127 |
| ENSG00000102935 | *ZNF423* | -4.844221898 | 3.09E-06 |
| ENSG00000274536 | *RP6-159A1.4* | -4.847759602 | 0.038484667 |
| ENSG00000187479 | *C11orf96* | -4.853802791 | 0.000124575 |
| ENSG00000184905 | *TCEAL2* | -4.853982856 | 0.000367039 |
| ENSG00000121577 | *POPDC2* | -4.85622759 | 2.94E-06 |
| ENSG00000137673 | *MMP7* | -4.861895973 | 1.78E-05 |
| ENSG00000106038 | *EVX1* | -4.869689544 | 0.012809569 |
| ENSG00000013725 | *CD6* | -4.882717036 | 0.000346462 |
| ENSG00000278126 | *RP11-454E5.4* | -4.882871148 | 0.002042416 |
| ENSG00000113319 | *RASGRF2* | -4.883491667 | 1.64E-08 |
| ENSG00000169885 | *CALML6* | -4.884329978 | 0.000200863 |
| ENSG00000178031 | *ADAMTSL1* | -4.903591539 | 4.69E-05 |
| ENSG00000183844 | *FAM3B* | -4.9041914 | 4.54E-05 |
| ENSG00000108370 | *RGS9* | -4.91082431 | 5.15E-06 |
| ENSG00000172985 | *SH3RF3* | -4.918092107 | 3.33E-09 |
| ENSG00000136235 | *GPNMB* | -4.918145106 | 9.44E-11 |
| ENSG00000206417 | *H1FX-AS1* | -4.919486268 | 9.33E-06 |
| ENSG00000255587 | *RAB44* | -4.922247749 | 0.040363984 |
| ENSG00000184451 | *CCR10* | -4.922790981 | 0.009728206 |
| ENSG00000186479 | *RGS7BP* | -4.929612161 | 0.009702283 |
| ENSG00000114200 | *BCHE* | -4.936032937 | 0.004425947 |
| ENSG00000100346 | *CACNA1I* | -4.936077178 | 0.044315427 |
| ENSG00000159433 | *STARD9* | -4.938090388 | 1.11E-07 |
| ENSG00000132702 | *HAPLN2* | -4.938520059 | 7.32E-09 |
| ENSG00000136404 | *TM6SF1* | -4.945111752 | 0.003980947 |
| ENSG00000142347 | *MYO1F* | -4.947347517 | 4.04E-07 |
| ENSG00000146205 | *ANO7* | -4.949578591 | 0.000128199 |
| ENSG00000091972 | *CD200* | -4.950080793 | 6.17E-07 |
| ENSG00000206450 | *HLA-B* | -4.952095531 | 1.87E-05 |
| ENSG00000065413 | *ANKRD44* | -4.952749073 | 1.61E-07 |
| ENSG00000105538 | *RASIP1* | -4.956449695 | 1.01E-07 |
| ENSG00000053918 | *KCNQ1* | -4.960281208 | 0.001115907 |
| ENSG00000140092 | *FBLN5* | -4.962857658 | 3.38E-05 |
| ENSG00000110448 | *CD5* | -4.963171379 | 0.001349605 |
| ENSG00000196196 | *HRCT1* | -4.964676655 | 0.001348416 |
| ENSG00000112137 | *PHACTR1* | -4.967483315 | 5.30E-05 |
| ENSG00000007062 | *PROM1* | -4.967785031 | 0.000761916 |
| ENSG00000128567 | *PODXL* | -4.973315938 | 2.64E-06 |
| ENSG00000218336 | *TENM3* | -4.983778987 | 6.31E-05 |
| ENSG00000279312 | *RP3-331H24.7* | -4.987229773 | 0.000315956 |
| ENSG00000267414 | *RP11-456K23.1* | -4.993977151 | 0.001229526 |
| ENSG00000123570 | *RAB9B* | -4.99573927 | 3.42E-05 |
| ENSG00000260572 | *RP11-16N11.2* | -4.99819551 | 0.007735754 |
| ENSG00000102385 | *DRP2* | -4.998301972 | 0.000143541 |
| ENSG00000261353 | *CTA-14H9.5* | -4.999747091 | 0.004587869 |
| ENSG00000266714 | *MYO15B* | -5.003771953 | 1.21E-07 |
| ENSG00000186417 | *GLDN* | -5.00658957 | 0.004922572 |
| ENSG00000147251 | *DOCK11* | -5.007353753 | 5.36E-07 |
| ENSG00000143110 | *C1orf162* | -5.008294855 | 6.40E-06 |
| ENSG00000120820 | *GLT8D2* | -5.009638266 | 0.000151549 |
| ENSG00000151322 | *NPAS3* | -5.009870763 | 0.036928623 |
| ENSG00000100302 | *RASD2* | -5.012835805 | 8.11E-05 |
| ENSG00000177791 | *MYOZ1* | -5.018897508 | 0.006141215 |
| ENSG00000115414 | *FN1* | -5.021685087 | 0.000527386 |
| ENSG00000180660 | *MAB21L1* | -5.026164692 | 0.038639713 |
| ENSG00000232126 | *HLA-B* | -5.032733627 | 1.91E-05 |
| ENSG00000138735 | *PDE5A* | -5.038921299 | 1.78E-07 |
| ENSG00000102962 | *CCL22* | -5.040095713 | 0.002245231 |
| ENSG00000225492 | *GBP1P1* | -5.040412918 | 1.97E-05 |
| ENSG00000095370 | *SH2D3C* | -5.046028483 | 2.39E-10 |
| ENSG00000111087 | *GLI1* | -5.055319746 | 0.006187741 |
| ENSG00000056487 | *PHF21B* | -5.055794982 | 0.002031249 |
| ENSG00000154027 | *AK5* | -5.062432425 | 0.01046712 |
| ENSG00000157404 | *KIT* | -5.064391526 | 0.000112803 |
| ENSG00000167994 | *RAB3IL1* | -5.065507085 | 1.05E-06 |
| ENSG00000170558 | *CDH2* | -5.077717598 | 0.047498072 |
| ENSG00000158113 | *LRRC43* | -5.080426293 | 0.016066819 |
| ENSG00000157502 | *MUM1L1* | -5.08407253 | 0.009291656 |
| ENSG00000101605 | *MYOM1* | -5.084750599 | 0.000902872 |
| ENSG00000064886 | *CHI3L2* | -5.09098083 | 0.001247992 |
| ENSG00000182575 | *NXPH3* | -5.092229815 | 3.89E-06 |
| ENSG00000223403 | *MEG9* | -5.092447437 | 0.009817907 |
| ENSG00000049768 | *FOXP3* | -5.100583292 | 0.011647489 |
| ENSG00000152495 | *CAMK4* | -5.101675461 | 9.41E-08 |
| ENSG00000142156 | *COL6A1* | -5.10411779 | 2.76E-16 |
| ENSG00000122694 | *GLIPR2* | -5.106161459 | 2.40E-06 |
| ENSG00000185482 | *STAC3* | -5.108971502 | 0.000293585 |
| ENSG00000205362 | *MT1A* | -5.10957763 | 0.001330369 |
| ENSG00000101194 | *SLC17A9* | -5.112639968 | 0.000977094 |
| ENSG00000221866 | *PLXNA4* | -5.116310254 | 0.046640274 |
| ENSG00000156113 | *KCNMA1* | -5.11682243 | 3.39E-16 |
| ENSG00000111879 | *FAM184A* | -5.122306413 | 3.73E-05 |
| ENSG00000213145 | *CRIP1* | -5.123246026 | 0.017728456 |
| ENSG00000158555 | *GDPD5* | -5.134495938 | 1.76E-06 |
| ENSG00000116852 | *KIF21B* | -5.14015234 | 0.008007689 |
| ENSG00000165269 | *AQP7* | -5.143349111 | 0.00959164 |
| ENSG00000107099 | *DOCK8* | -5.146610188 | 2.03E-08 |
| ENSG00000163219 | *ARHGAP25* | -5.148597174 | 0.000103825 |
| ENSG00000196358 | *NTNG2* | -5.154658143 | 0.000267923 |
| ENSG00000198832 | *SELM* | -5.156992954 | 5.67E-08 |
| ENSG00000145416 | *1-Mar* | -5.168041313 | 4.73E-06 |
| ENSG00000197361 | *FBXL22* | -5.175189675 | 1.51E-09 |
| ENSG00000267060 | *PTGES3L* | -5.181944542 | 0.037926666 |
| ENSG00000130988 | *RGN* | -5.203136873 | 5.69E-08 |
| ENSG00000177374 | *HIC1* | -5.205200519 | 4.61E-08 |
| ENSG00000269516 | *CYP4F23P* | -5.206209233 | 0.049226321 |
| ENSG00000177990 | *DPY19L2* | -5.210566065 | 5.47E-10 |
| ENSG00000111664 | *GNB3* | -5.211359779 | 0.001585667 |
| ENSG00000168243 | *GNG4* | -5.211514075 | 1.49E-05 |
| ENSG00000206452 | *HLA-C* | -5.214013505 | 0.004651806 |
| ENSG00000163644 | *PPM1K* | -5.2147472 | 2.61E-10 |
| ENSG00000260296 | *RP11-395I6.3* | -5.215505051 | 0.000843273 |
| ENSG00000188859 | *FAM78B* | -5.226257013 | 0.003558196 |
| ENSG00000155657 | *TTN* | -5.227050017 | 9.57E-05 |
| ENSG00000060709 | *RIMBP2* | -5.228093452 | 0.020879752 |
| ENSG00000042062 | *FAM65C* | -5.229900502 | 1.72E-08 |
| ENSG00000169760 | *NLGN1* | -5.2370614 | 0.027162343 |
| ENSG00000134242 | *PTPN22* | -5.23900736 | 0.005474542 |
| ENSG00000164764 | *SBSPON* | -5.244821144 | 0.000202558 |
| ENSG00000143127 | *ITGA10* | -5.245911426 | 2.12E-05 |
| ENSG00000168280 | *KIF5C* | -5.248888074 | 7.83E-05 |
| ENSG00000152804 | *HHEX* | -5.253117794 | 8.06E-05 |
| ENSG00000162407 | *PPAP2B* | -5.271670879 | 1.56E-13 |
| ENSG00000171016 | *PYGO1* | -5.275122858 | 3.80E-05 |
| ENSG00000163661 | *PTX3* | -5.278615752 | 0.04545462 |
| ENSG00000279117 | *CTD-2562J17.6* | -5.285053115 | 0.000193313 |
| ENSG00000103742 | *IGDCC4* | -5.292971914 | 0.040858026 |
| ENSG00000101342 | *TLDC2* | -5.295351617 | 0.004393806 |
| ENSG00000087258 | *GNAO1* | -5.295631955 | 6.21E-05 |
| ENSG00000184785 | *SMIM10* | -5.297954259 | 7.35E-06 |
| ENSG00000142102 | *ATHL1* | -5.308163953 | 7.18E-10 |
| ENSG00000197405 | *C5AR1* | -5.311541204 | 1.25E-06 |
| ENSG00000179909 | *ZNF154* | -5.315353996 | 7.36E-07 |
| ENSG00000165810 | *BTNL9* | -5.324626132 | 0.000335221 |
| ENSG00000076344 | *RGS11* | -5.326235258 | 3.48E-09 |
| ENSG00000172508 | *CARNS1* | -5.326718939 | 0.002462848 |
| ENSG00000177363 | *LRRN4CL* | -5.331881023 | 2.02E-05 |
| ENSG00000152213 | *ARL11* | -5.332187893 | 0.000700442 |
| ENSG00000179583 | *CIITA* | -5.334140398 | 6.47E-05 |
| ENSG00000184988 | *TMEM106A* | -5.336824581 | 2.82E-06 |
| ENSG00000111729 | *CLEC4A* | -5.340256412 | 0.000113138 |
| ENSG00000102879 | *CORO1A* | -5.340713134 | 1.63E-05 |
| ENSG00000154589 | *LY96* | -5.343730253 | 0.000370482 |
| ENSG00000152377 | *SPOCK1* | -5.351543478 | 6.06E-05 |
| ENSG00000105419 | *MEIS3* | -5.352434602 | 2.17E-07 |
| ENSG00000167680 | *SEMA6B* | -5.354606615 | 9.20E-06 |
| ENSG00000127743 | *IL17B* | -5.355213849 | 0.02122158 |
| ENSG00000109265 | *KIAA1211* | -5.360697026 | 0.004232362 |
| ENSG00000100593 | *ISM2* | -5.36860599 | 0.002736517 |
| ENSG00000184730 | *APOBR* | -5.368997371 | 0.000432746 |
| ENSG00000170382 | *LRRN2* | -5.369747121 | 0.002687564 |
| ENSG00000278709 | *NKILA* | -5.370448857 | 0.002156468 |
| ENSG00000197536 | *C5orf56* | -5.376392828 | 6.97E-09 |
| ENSG00000165731 | *RET* | -5.376804586 | 9.31E-05 |
| ENSG00000118777 | *ABCG2* | -5.378491744 | 1.54E-05 |
| ENSG00000138356 | *AOX1* | -5.382110827 | 1.06E-07 |
| ENSG00000132622 | *HSPA12B* | -5.386967921 | 6.00E-06 |
| ENSG00000128218 | *VPREB3* | -5.389298015 | 0.022224289 |
| ENSG00000162576 | *MXRA8* | -5.391749948 | 5.56E-12 |
| ENSG00000161638 | *ITGA5* | -5.392044358 | 4.52E-10 |
| ENSG00000125910 | *S1PR4* | -5.393568193 | 0.000229555 |
| ENSG00000142552 | *RCN3* | -5.393627947 | 5.38E-12 |
| ENSG00000173705 | *SUSD5* | -5.395231914 | 0.00621353 |
| ENSG00000130294 | *KIF1A* | -5.396429598 | 0.004928554 |
| ENSG00000133106 | *EPSTI1* | -5.401611217 | 5.60E-06 |
| ENSG00000183307 | *CECR6* | -5.406242228 | 0.003874983 |
| ENSG00000167984 | *NLRC3* | -5.406927287 | 3.94E-07 |
| ENSG00000273771 | *RP11-236L14.2* | -5.408845359 | 0.005935371 |
| ENSG00000139914 | *FITM1* | -5.409436999 | 0.009795574 |
| ENSG00000164303 | *ENPP6* | -5.410757117 | 0.000622847 |
| ENSG00000163491 | *NEK10* | -5.424376697 | 0.02578172 |
| ENSG00000103472 | *RRN3P2* | -5.428793178 | 2.26E-05 |
| ENSG00000115935 | *WIPF1* | -5.428825692 | 1.94E-11 |
| ENSG00000164236 | *ANKRD33B* | -5.430369376 | 0.000296397 |
| ENSG00000198003 | *CCDC151* | -5.433720718 | 0.04788399 |
| ENSG00000163072 | *NOSTRIN* | -5.435099657 | 0.006435194 |
| ENSG00000128262 | *POM121L9P* | -5.436215096 | 0.003620045 |
| ENSG00000090376 | *IRAK3* | -5.436993812 | 6.17E-07 |
| ENSG00000150337 | *FCGR1A* | -5.448155809 | 0.002025044 |
| ENSG00000206052 | *DOK6* | -5.448548237 | 0.026630715 |
| ENSG00000001626 | *CFTR* | -5.451446325 | 0.001960155 |
| ENSG00000167371 | *PRRT2* | -5.461499914 | 0.001323951 |
| ENSG00000142611 | *PRDM16* | -5.462089692 | 0.028383655 |
| ENSG00000137486 | *ARRB1* | -5.465729962 | 7.38E-08 |
| ENSG00000111913 | *FAM65B* | -5.468158847 | 4.25E-11 |
| ENSG00000005379 | *BZRAP1* | -5.471676898 | 1.43E-07 |
| ENSG00000131730 | *CKMT2* | -5.474838972 | 9.09E-05 |
| ENSG00000115112 | *TFCP2L1* | -5.486767555 | 0.00129073 |
| ENSG00000148219 | *ASTN2* | -5.491029502 | 5.80E-10 |
| ENSG00000214578 | *HMGN2P15* | -5.491744814 | 0.039263018 |
| ENSG00000179761 | *PIPOX* | -5.494510285 | 5.83E-06 |
| ENSG00000168071 | *CCDC88B* | -5.495185231 | 7.75E-06 |
| ENSG00000141639 | *MAPK4* | -5.497116455 | 8.47E-06 |
| ENSG00000198959 | *TGM2* | -5.497208279 | 2.07E-16 |
| ENSG00000171189 | *GRIK1* | -5.500745487 | 0.015944891 |
| ENSG00000158517 | *NCF1* | -5.505114611 | 0.000247133 |
| ENSG00000125144 | *MT1G* | -5.509922413 | 2.93E-09 |
| ENSG00000112303 | *VNN2* | -5.51168056 | 0.001989006 |
| ENSG00000104043 | *ATP8B4* | -5.520690236 | 0.000110735 |
| ENSG00000144596 | *GRIP2* | -5.522467048 | 8.31E-05 |
| ENSG00000008118 | *CAMK1G* | -5.524964804 | 0.000780485 |
| ENSG00000105737 | *GRIK5* | -5.526837623 | 2.37E-07 |
| ENSG00000068615 | *REEP1* | -5.527990026 | 7.08E-05 |
| ENSG00000122176 | *FMOD* | -5.531731495 | 2.08E-14 |
| ENSG00000169213 | *RAB3B* | -5.53202628 | 0.000483901 |
| ENSG00000042980 | *ADAM28* | -5.53249847 | 0.000289734 |
| ENSG00000130558 | *OLFM1* | -5.53306501 | 6.14E-07 |
| ENSG00000110446 | *SLC15A3* | -5.538499414 | 7.65E-07 |
| ENSG00000204584 | *RP11-304F15.3* | -5.544871697 | 0.003246779 |
| ENSG00000123685 | *BATF3* | -5.548347444 | 0.001645997 |
| ENSG00000166086 | *JAM3* | -5.548766069 | 2.77E-10 |
| ENSG00000166432 | *ZMAT1* | -5.552460404 | 9.22E-07 |
| ENSG00000180616 | *SSTR2* | -5.554524932 | 6.85E-05 |
| ENSG00000214189 | *ZNF788* | -5.554932684 | 0.000846742 |
| ENSG00000108821 | *COL1A1* | -5.556246515 | 1.56E-10 |
| ENSG00000225968 | *ELFN1* | -5.560657383 | 0.042793753 |
| ENSG00000196353 | *CPNE4* | -5.565005923 | 0.001613592 |
| ENSG00000184838 | *PRR16* | -5.570374217 | 1.56E-05 |
| ENSG00000158560 | *DYNC1I1* | -5.571716044 | 4.80E-07 |
| ENSG00000119535 | *CSF3R* | -5.571782999 | 0.000150219 |
| ENSG00000176402 | *GJC3* | -5.572998323 | 0.014089914 |
| ENSG00000146038 | *DCDC2* | -5.573431552 | 0.012203738 |
| ENSG00000157766 | *ACAN* | -5.574377035 | 0.036332902 |
| ENSG00000072818 | *ACAP1* | -5.57618457 | 3.29E-07 |
| ENSG00000169248 | *CXCL11* | -5.57978554 | 0.043507665 |
| ENSG00000099954 | *CECR2* | -5.587781977 | 0.000571986 |
| ENSG00000187486 | *KCNJ11* | -5.597558346 | 0.002997053 |
| ENSG00000184613 | *NELL2* | -5.599288286 | 0.000466897 |
| ENSG00000055118 | *KCNH2* | -5.613512196 | 7.61E-05 |
| ENSG00000050030 | *KIAA2022* | -5.614793043 | 0.003890178 |
| ENSG00000187699 | *C2orf88* | -5.616478125 | 8.13E-07 |
| ENSG00000127528 | *KLF2* | -5.621749675 | 5.45E-05 |
| ENSG00000278989 | *RP11-762L8.6* | -5.622396394 | 0.041349781 |
| ENSG00000165995 | *CACNB2* | -5.627537403 | 7.88E-08 |
| ENSG00000274444 | *RP11-128A17.2* | -5.629169982 | 0.048765771 |
| ENSG00000170681 | *MURC* | -5.630158354 | 0.009200791 |
| ENSG00000135502 | *SLC26A10* | -5.630928865 | 0.000319523 |
| ENSG00000135454 | *B4GALNT1* | -5.640025625 | 2.41E-06 |
| ENSG00000163909 | *HEYL* | -5.643920667 | 6.45E-06 |
| ENSG00000133800 | *LYVE1* | -5.644465986 | 0.003864447 |
| ENSG00000215018 | *COL28A1* | -5.649934934 | 0.009203958 |
| ENSG00000280153 | *RP11-876N24.3* | -5.655407478 | 4.97E-06 |
| ENSG00000167100 | *SAMD14* | -5.661819694 | 0.000271097 |
| ENSG00000162614 | *NEXN* | -5.666468408 | 2.19E-05 |
| ENSG00000155465 | *SLC7A7* | -5.668012521 | 1.66E-06 |
| ENSG00000134871 | *COL4A2* | -5.668831921 | 1.92E-06 |
| ENSG00000162654 | *GBP4* | -5.678813323 | 6.64E-06 |
| ENSG00000250056 | *LINC01018* | -5.683862661 | 0.003429115 |
| ENSG00000188305 | *C19orf35* | -5.688076305 | 0.040924889 |
| ENSG00000078814 | *MYH7B* | -5.69038332 | 2.94E-06 |
| ENSG00000171533 | *MAP6* | -5.693712364 | 5.14E-10 |
| ENSG00000171798 | *KNDC1* | -5.700386219 | 0.004536349 |
| ENSG00000256164 | *CCND2-AS1* | -5.703046388 | 0.031658279 |
| ENSG00000159212 | *CLIC6* | -5.708543721 | 1.12E-06 |
| ENSG00000204136 | *GGTA1P* | -5.709009264 | 2.33E-06 |
| ENSG00000244119 | *PDCL3P4* | -5.711031761 | 0.000887986 |
| ENSG00000162989 | *KCNJ3* | -5.712929672 | 0.037021687 |
| ENSG00000125869 | *LAMP5* | -5.718203945 | 0.018299917 |
| ENSG00000124479 | *NDP* | -5.726859108 | 0.001122908 |
| ENSG00000166501 | *PRKCB* | -5.727052638 | 7.44E-06 |
| ENSG00000117245 | *KIF17* | -5.728965918 | 0.00386123 |
| ENSG00000205277 | *MUC12* | -5.738341813 | 0.001353318 |
| ENSG00000135077 | *HAVCR2* | -5.751938534 | 3.72E-07 |
| ENSG00000153064 | *BANK1* | -5.752948733 | 4.97E-06 |
| ENSG00000114013 | *CD86* | -5.755189913 | 4.16E-05 |
| ENSG00000262222 | *RP11-876N24.4* | -5.756303102 | 0.000826478 |
| ENSG00000104951 | *IL4I1* | -5.761673549 | 0.000460822 |
| ENSG00000189129 | *PLAC9* | -5.76459925 | 5.95E-05 |
| ENSG00000150625 | *GPM6A* | -5.76609809 | 0.008903807 |
| ENSG00000196684 | *HSH2D* | -5.78130591 | 0.001052536 |
| ENSG00000125675 | *GRIA3* | -5.784235145 | 0.037178607 |
| ENSG00000206549 | *PRSS50* | -5.784564205 | 0.000620762 |
| ENSG00000136490 | *LIMD2* | -5.786187459 | 7.71E-06 |
| ENSG00000011590 | *ZBTB32* | -5.786649024 | 0.002687647 |
| ENSG00000198795 | *ZNF521* | -5.789605038 | 0.000332056 |
| ENSG00000118004 | *COLEC11* | -5.791282721 | 0.015534451 |
| ENSG00000198019 | *FCGR1B* | -5.792521003 | 0.035049142 |
| ENSG00000140749 | *IGSF6* | -5.79876646 | 1.02E-06 |
| ENSG00000242600 | *MBL1P* | -5.807242987 | 0.009497553 |
| ENSG00000272971 | *RP11-284F21.11* | -5.80781802 | 0.022147285 |
| ENSG00000113657 | *DPYSL3* | -5.808748755 | 7.81E-07 |
| ENSG00000112309 | *B3GAT2* | -5.81617392 | 0.035160938 |
| ENSG00000087495 | *PHACTR3* | -5.818223242 | 0.035160938 |
| ENSG00000115590 | *IL1R2* | -5.821741679 | 0.020675157 |
| ENSG00000231672 | *DIRC3* | -5.823820525 | 0.027367613 |
| ENSG00000083454 | *P2RX5* | -5.827500957 | 0.003433988 |
| ENSG00000143248 | *RGS5* | -5.830154948 | 4.28E-12 |
| ENSG00000115896 | *PLCL1* | -5.830949788 | 8.05E-06 |
| ENSG00000141449 | *GREB1L* | -5.834742819 | 0.000245507 |
| ENSG00000138135 | *CH25H* | -5.839269045 | 0.002633495 |
| ENSG00000115461 | *IGFBP5* | -5.852365736 | 3.87E-06 |
| ENSG00000175463 | *TBC1D10C* | -5.860926559 | 1.64E-05 |
| ENSG00000227136 | *LINC00595* | -5.866267577 | 0.023079322 |
| ENSG00000113212 | *PCDHB7* | -5.868411698 | 0.001014957 |
| ENSG00000273100 | *RP11-302L19.3* | -5.868972514 | 0.040011296 |
| ENSG00000263934 | *SNORD3A* | -5.868996998 | 5.87E-07 |
| ENSG00000107518 | *ATRNL1* | -5.869574756 | 0.000384238 |
| ENSG00000126950 | *TMEM35* | -5.870222307 | 1.44E-12 |
| ENSG00000127903 | *ZNF835* | -5.870361093 | 0.002997053 |
| ENSG00000184524 | *CEND1* | -5.871569888 | 2.02E-05 |
| ENSG00000160255 | *ITGB2* | -5.876531307 | 2.62E-08 |
| ENSG00000162711 | *NLRP3* | -5.876752856 | 0.000887325 |
| ENSG00000104490 | *NCALD* | -5.878332223 | 5.69E-07 |
| ENSG00000150048 | *CLEC1A* | -5.887654904 | 0.000461383 |
| ENSG00000129682 | *FGF13* | -5.889876613 | 5.34E-07 |
| ENSG00000074966 | *TXK* | -5.893981461 | 0.002448557 |
| ENSG00000139193 | *CD27* | -5.896147419 | 0.000203053 |
| ENSG00000182183 | *FAM159A* | -5.906335139 | 0.006462785 |
| ENSG00000087245 | *MMP2* | -5.908724237 | 1.64E-16 |
| ENSG00000162174 | *ASRGL1* | -5.909693316 | 3.77E-06 |
| ENSG00000197614 | *MFAP5* | -5.910168089 | 0.000407931 |
| ENSG00000102524 | *TNFSF13B* | -5.925786488 | 1.41E-05 |
| ENSG00000138411 | *HECW2* | -5.926435949 | 0.000690117 |
| ENSG00000162512 | *SDC3* | -5.932329839 | 1.24E-08 |
| ENSG00000078295 | *ADCY2* | -5.936891969 | 1.49E-06 |
| ENSG00000015285 | *WAS* | -5.943583467 | 3.26E-06 |
| ENSG00000128573 | *FOXP2* | -5.9514196 | 0.001660052 |
| ENSG00000144668 | *ITGA9* | -5.956948051 | 2.40E-13 |
| ENSG00000128596 | *CCDC136* | -5.961646356 | 8.11E-09 |
| ENSG00000159958 | *TNFRSF13C* | -5.961684918 | 0.007732923 |
| ENSG00000172215 | *CXCR6* | -5.964032332 | 9.09E-05 |
| ENSG00000008277 | *ADAM22* | -5.967597283 | 1.79E-13 |
| ENSG00000123572 | *NRK* | -5.971468933 | 0.000806745 |
| ENSG00000117013 | *KCNQ4* | -5.974318366 | 1.12E-05 |
| ENSG00000198075 | *SULT1C4* | -5.974411734 | 0.000621015 |
| ENSG00000149970 | *CNKSR2* | -5.974881745 | 0.017443272 |
| ENSG00000189184 | *PCDH18* | -5.980815261 | 4.81E-11 |
| ENSG00000183091 | *NEB* | -5.982552285 | 0.000190757 |
| ENSG00000196569 | *LAMA2* | -5.984842741 | 2.69E-12 |
| ENSG00000198734 | *F5* | -5.986323078 | 0.002543998 |
| ENSG00000247982 | *LINC00926* | -5.987630625 | 6.08E-06 |
| ENSG00000168993 | *CPLX1* | -5.990232955 | 7.16E-06 |
| ENSG00000152953 | *STK32B* | -5.991799188 | 0.020879752 |
| ENSG00000109819 | *PPARGC1A* | -5.998123088 | 0.000166184 |
| ENSG00000170011 | *MYRIP* | -6.001882724 | 0.027266991 |
| ENSG00000167157 | *PRRX2* | -6.00484511 | 2.99E-07 |
| ENSG00000196376 | *SLC35F1* | -6.01595018 | 0.045541115 |
| ENSG00000049540 | *ELN* | -6.018374071 | 0.031584425 |
| ENSG00000166592 | *RRAD* | -6.032046193 | 1.22E-06 |
| ENSG00000171115 | *GIMAP8* | -6.042889259 | 1.21E-06 |
| ENSG00000113396 | *SLC27A6* | -6.048169996 | 5.29E-06 |
| ENSG00000148541 | *FAM13C* | -6.054687727 | 2.66E-06 |
| ENSG00000224397 | *LINC01272* | -6.05721642 | 0.003244426 |
| ENSG00000173114 | *LRRN3* | -6.062019486 | 0.003690261 |
| ENSG00000164434 | *FABP7* | -6.062419044 | 0.023514001 |
| ENSG00000189056 | *RELN* | -6.071325196 | 0.027383231 |
| ENSG00000197646 | *PDCD1LG2* | -6.074090131 | 0.001813096 |
| ENSG00000115252 | *PDE1A* | -6.076096575 | 0.000188225 |
| ENSG00000186310 | *NAP1L3* | -6.08010634 | 0.000466633 |
| ENSG00000100055 | *CYTH4* | -6.083085972 | 2.77E-08 |
| ENSG00000279369 | *RP11-51F16.1* | -6.083173162 | 0.0012771 |
| ENSG00000007908 | *SELE* | -6.083207446 | 9.32E-08 |
| ENSG00000152402 | *GUCY1A2* | -6.084573382 | 0.000211988 |
| ENSG00000154262 | *ABCA6* | -6.085836065 | 2.61E-05 |
| ENSG00000149599 | *DUSP15* | -6.086611822 | 6.13E-05 |
| ENSG00000250138 | *RP11-848G14.5* | -6.086831141 | 0.02203372 |
| ENSG00000233820 | *RP11-535M15.2* | -6.087613523 | 0.04221967 |
| ENSG00000206190 | *ATP10A* | -6.091052158 | 1.27E-08 |
| ENSG00000171714 | *ANO5* | -6.091578817 | 1.54E-07 |
| ENSG00000182870 | *GALNT9* | -6.096470506 | 0.041988052 |
| ENSG00000067798 | *NAV3* | -6.099646712 | 1.70E-05 |
| ENSG00000178538 | *CA8* | -6.102861024 | 0.000656231 |
| ENSG00000132688 | *NES* | -6.110272523 | 1.43E-09 |
| ENSG00000170509 | *HSD17B13* | -6.110880291 | 0.005559529 |
| ENSG00000108515 | *ENO3* | -6.11249435 | 1.79E-06 |
| ENSG00000149633 | *KIAA1755* | -6.116123148 | 2.10E-08 |
| ENSG00000095713 | *CRTAC1* | -6.120116158 | 0.025363036 |
| ENSG00000009790 | *TRAF3IP3* | -6.128797325 | 7.56E-06 |
| ENSG00000061455 | *PRDM6* | -6.133838666 | 6.41E-08 |
| ENSG00000140853 | *NLRC5* | -6.136113663 | 5.14E-10 |
| ENSG00000153902 | *LGI4* | -6.136584528 | 2.61E-10 |
| ENSG00000201185 | *RNA5SP202* | -6.141163204 | 0.000409144 |
| ENSG00000188404 | *SELL* | -6.144727315 | 9.37E-05 |
| ENSG00000080854 | *IGSF9B* | -6.14568116 | 0.00013736 |
| ENSG00000167912 | *RP11-25K19.1* | -6.147222355 | 0.025363036 |
| ENSG00000239884 | *RN7SL608P* | -6.147793519 | 0.033594682 |
| ENSG00000144278 | *GALNT13* | -6.148829753 | 0.009832403 |
| ENSG00000148655 | *C10orf11* | -6.154162283 | 1.98E-06 |
| ENSG00000117069 | *ST6GALNAC5* | -6.15655471 | 0.036338896 |
| ENSG00000136630 | *HLX* | -6.159642353 | 7.49E-07 |
| ENSG00000108381 | *ASPA* | -6.162887485 | 1.20E-07 |
| ENSG00000187260 | *WDR86* | -6.170337039 | 0.000177673 |
| ENSG00000214491 | *SEC14L6* | -6.173329517 | 0.000524327 |
| ENSG00000153446 | *C16orf89* | -6.17574921 | 8.28E-06 |
| ENSG00000067840 | *PDZD4* | -6.17990007 | 6.06E-07 |
| ENSG00000180525 | *PRR26* | -6.182564666 | 0.011337347 |
| ENSG00000250510 | *GPR162* | -6.186420301 | 5.50E-06 |
| ENSG00000142185 | *TRPM2* | -6.200204583 | 8.59E-06 |
| ENSG00000162543 | *UBXN10* | -6.20376821 | 3.33E-05 |
| ENSG00000255471 | *RP11-736K20.5* | -6.208112548 | 0.044117054 |
| ENSG00000170989 | *S1PR1* | -6.213832127 | 3.30E-06 |
| ENSG00000183423 | *LRIT3* | -6.214071074 | 0.045447398 |
| ENSG00000156076 | *WIF1* | -6.216874143 | 7.15E-05 |
| ENSG00000136541 | *ERMN* | -6.223650804 | 0.045306549 |
| ENSG00000115616 | *SLC9A2* | -6.22739161 | 0.020468428 |
| ENSG00000121769 | *FABP3* | -6.240398239 | 4.35E-10 |
| ENSG00000167037 | *SGSM1* | -6.241336513 | 0.00045097 |
| ENSG00000228221 | *LINC00578* | -6.241928088 | 0.014089914 |
| ENSG00000138646 | *HERC5* | -6.251318738 | 2.82E-07 |
| ENSG00000139200 | *PIANP* | -6.253456657 | 9.49E-06 |
| ENSG00000089820 | *ARHGAP4* | -6.255876504 | 7.61E-09 |
| ENSG00000149781 | *FERMT3* | -6.262948621 | 1.45E-08 |
| ENSG00000136869 | *TLR4* | -6.264647441 | 6.34E-08 |
| ENSG00000105499 | *PLA2G4C* | -6.265530236 | 3.94E-06 |
| ENSG00000132854 | *KANK4* | -6.265720981 | 0.01468915 |
| ENSG00000005108 | *THSD7A* | -6.269744788 | 1.24E-05 |
| ENSG00000158859 | *ADAMTS4* | -6.272279038 | 1.55E-11 |
| ENSG00000027869 | *SH2D2A* | -6.277907724 | 3.98E-05 |
| ENSG00000121895 | *TMEM156* | -6.282662408 | 0.001395689 |
| ENSG00000113532 | *ST8SIA4* | -6.283372345 | 0.000319059 |
| ENSG00000128253 | *RFPL2* | -6.283583887 | 0.030702313 |
| ENSG00000135333 | *EPHA7* | -6.286682138 | 3.03E-06 |
| ENSG00000159403 | *C1R* | -6.292683311 | 6.71E-20 |
| ENSG00000151812 | *SLC35F4* | -6.2977699 | 0.007679375 |
| ENSG00000122025 | *FLT3* | -6.301110555 | 0.016692252 |
| ENSG00000167895 | *TMC8* | -6.306066481 | 1.56E-07 |
| ENSG00000164344 | *KLKB1* | -6.307298463 | 0.010076767 |
| ENSG00000255389 | *C6orf3* | -6.307949707 | 0.003866102 |
| ENSG00000253304 | *TMEM200B* | -6.30845395 | 1.83E-11 |
| ENSG00000126353 | *CCR7* | -6.319132156 | 0.00043321 |
| ENSG00000120251 | *GRIA2* | -6.324316858 | 0.042736226 |
| ENSG00000188820 | *FAM26F* | -6.332724842 | 1.79E-05 |
| ENSG00000270640 | *RP11-373D23.2* | -6.338697713 | 0.021005257 |
| ENSG00000255974 | *CYP2A6* | -6.341711781 | 0.026067727 |
| ENSG00000019582 | *CD74* | -6.346430863 | 1.34E-11 |
| ENSG00000197043 | *ANXA6* | -6.350706884 | 5.81E-12 |
| ENSG00000109956 | *B3GAT1* | -6.352193452 | 0.011474841 |
| ENSG00000115232 | *ITGA4* | -6.352502929 | 0.001911381 |
| ENSG00000104814 | *MAP4K1* | -6.358620441 | 1.19E-06 |
| ENSG00000239911 | *PRKAG2-AS1* | -6.361127029 | 9.81E-05 |
| ENSG00000172348 | *RCAN2* | -6.370343919 | 1.47E-11 |
| ENSG00000265542 | *RP11-60A24.3* | -6.374262893 | 0.031584425 |
| ENSG00000169946 | *ZFPM2* | -6.379665019 | 0.027500911 |
| ENSG00000161940 | *BCL6B* | -6.380703343 | 9.20E-08 |
| ENSG00000282608 | *ADORA3* | -6.388462997 | 0.014914051 |
| ENSG00000127920 | *GNG11* | -6.388955632 | 5.27E-06 |
| ENSG00000228784 | *LINC00954* | -6.403255595 | 0.005959766 |
| ENSG00000188060 | *RAB42* | -6.404830164 | 0.000452253 |
| ENSG00000189292 | *FAM150B* | -6.408751001 | 0.012585157 |
| ENSG00000198354 | *DCAF12L2* | -6.410919602 | 0.036457399 |
| ENSG00000163762 | *TM4SF18* | -6.411683261 | 0.000467942 |
| ENSG00000205403 | *CFI* | -6.416938848 | 4.03E-11 |
| ENSG00000044524 | *EPHA3* | -6.42150664 | 1.23E-15 |
| ENSG00000148053 | *NTRK2* | -6.42566505 | 8.11E-09 |
| ENSG00000126759 | *CFP* | -6.427647457 | 6.84E-05 |
| ENSG00000234336 | *JAZF1-AS1* | -6.430544413 | 0.020809576 |
| ENSG00000101082 | *SLA2* | -6.431867514 | 0.00800837 |
| ENSG00000153976 | *HS3ST3A1* | -6.449079348 | 6.24E-06 |
| ENSG00000273179 | *RP11-20I20.4* | -6.455364268 | 0.006717753 |
| ENSG00000086730 | *LAT2* | -6.462165779 | 6.76E-08 |
| ENSG00000147588 | *PMP2* | -6.468948844 | 0.037178607 |
| ENSG00000100365 | *NCF4* | -6.469362277 | 1.21E-05 |
| ENSG00000231346 | *LINC01160* | -6.470805555 | 0.002997053 |
| ENSG00000153823 | *PID1* | -6.476005852 | 6.03E-07 |
| ENSG00000111052 | *LIN7A* | -6.47621715 | 0.007329998 |
| ENSG00000167641 | *PPP1R14A* | -6.476868289 | 2.37E-09 |
| ENSG00000182557 | *SPNS3* | -6.478232482 | 0.017220431 |
| ENSG00000173868 | *PHOSPHO1* | -6.487348141 | 0.038954212 |
| ENSG00000187808 | *SOWAHD* | -6.488914471 | 0.020533687 |
| ENSG00000166341 | *DCHS1* | -6.494382488 | 7.26E-11 |
| ENSG00000065534 | *MYLK* | -6.495486705 | 2.95E-09 |
| ENSG00000268089 | *GABRQ* | -6.499383891 | 7.83E-05 |
| ENSG00000165323 | *FAT3* | -6.500439269 | 7.61E-08 |
| ENSG00000276975 | *HYDIN2* | -6.501500279 | 0.011618834 |
| ENSG00000175229 | *GAL3ST3* | -6.502165503 | 0.022794415 |
| ENSG00000169224 | *GCSAML* | -6.503243444 | 0.035904206 |
| ENSG00000186281 | *GPAT2* | -6.51024487 | 0.020307632 |
| ENSG00000083067 | *TRPM3* | -6.517822088 | 0.011727751 |
| ENSG00000162892 | *IL24* | -6.519076715 | 0.000431413 |
| ENSG00000116833 | *NR5A2* | -6.522575881 | 0.000994512 |
| ENSG00000196159 | *FAT4* | -6.525452963 | 7.99E-09 |
| ENSG00000164530 | *PI16* | -6.527330844 | 0.000374966 |
| ENSG00000176533 | *GNG7* | -6.533741199 | 1.20E-14 |
| ENSG00000174611 | *KY* | -6.538287022 | 0.006263508 |
| ENSG00000131401 | *NAPSB* | -6.539731153 | 6.40E-06 |
| ENSG00000149150 | *SLC43A1* | -6.549172854 | 3.28E-09 |
| ENSG00000055955 | *ITIH4* | -6.552012144 | 0.030475855 |
| ENSG00000149403 | *GRIK4* | -6.55442925 | 0.036851834 |
| ENSG00000135094 | *SDS* | -6.555472526 | 0.000495038 |
| ENSG00000162687 | *KCNT2* | -6.556077547 | 0.005741251 |
| ENSG00000143028 | *SYPL2* | -6.558663308 | 7.75E-06 |
| ENSG00000148516 | *ZEB1* | -6.558796283 | 2.95E-09 |
| ENSG00000113924 | *HGD* | -6.561388449 | 1.11E-06 |
| ENSG00000186510 | *CLCNKA* | -6.565916282 | 0.022296206 |
| ENSG00000154133 | *ROBO4* | -6.568036865 | 1.54E-07 |
| ENSG00000213514 | *RP11-428P16.2* | -6.574178367 | 0.016461195 |
| ENSG00000158186 | *MRAS* | -6.57498395 | 1.55E-09 |
| ENSG00000123119 | *NECAB1* | -6.575886429 | 0.000675549 |
| ENSG00000157103 | *SLC6A1* | -6.575951123 | 0.006661841 |
| ENSG00000198756 | *COLGALT2* | -6.580726217 | 2.78E-07 |
| ENSG00000166960 | *CCDC178* | -6.593512425 | 0.001835933 |
| ENSG00000233117 | *LINC00702* | -6.607776701 | 0.022924293 |
| ENSG00000276305 | *AC106788.1* | -6.614120438 | 0.036614203 |
| ENSG00000272491 | *RP5-1024N4.4* | -6.617032543 | 0.03302589 |
| ENSG00000146151 | *HMGCLL1* | -6.618817264 | 0.011154619 |
| ENSG00000155962 | *CLIC2* | -6.621720618 | 5.22E-07 |
| ENSG00000074370 | *ATP2A3* | -6.627660836 | 2.88E-06 |
| ENSG00000178199 | *ZC3H12D* | -6.628026212 | 0.000288834 |
| ENSG00000183571 | *PGPEP1L* | -6.630813196 | 0.025845181 |
| ENSG00000068976 | *PYGM* | -6.633271956 | 4.59E-12 |
| ENSG00000188282 | *RUFY4* | -6.636010624 | 0.001356824 |
| ENSG00000245954 | *RP11-18H21.1* | -6.641750508 | 0.02442185 |
| ENSG00000018280 | *SLC11A1* | -6.642941308 | 1.22E-07 |
| ENSG00000168702 | *LRP1B* | -6.644619466 | 0.002111693 |
| ENSG00000150637 | *CD226* | -6.645391981 | 0.004724956 |
| ENSG00000161929 | *SCIMP* | -6.646896598 | 0.000532638 |
| ENSG00000172260 | *NEGR1* | -6.652310378 | 3.14E-05 |
| ENSG00000135298 | *ADGRB3* | -6.656290066 | 0.027162343 |
| ENSG00000131018 | *SYNE1* | -6.661658285 | 2.40E-24 |
| ENSG00000214787 | *MS4A4E* | -6.664082294 | 0.024081364 |
| ENSG00000173714 | *WFIKKN2* | -6.666332985 | 0.038034188 |
| ENSG00000114654 | *EFCC1* | -6.667145369 | 5.37E-05 |
| ENSG00000185652 | *NTF3* | -6.676236679 | 0.001984015 |
| ENSG00000164128 | *NPY1R* | -6.67926326 | 0.015350771 |
| ENSG00000236383 | *LINC00854* | -6.683593376 | 0.011948398 |
| ENSG00000135097 | *MSI1* | -6.692450141 | 0.009575786 |
| ENSG00000154258 | *ABCA9* | -6.698555658 | 2.37E-05 |
| ENSG00000087303 | *NID2* | -6.70024553 | 2.24E-07 |
| ENSG00000273733 | *CTC-510F12.7* | -6.702610568 | 0.005299535 |
| ENSG00000196104 | *SPOCK3* | -6.712151738 | 4.52E-10 |
| ENSG00000101336 | *HCK* | -6.713201221 | 2.93E-08 |
| ENSG00000185736 | *ADARB2* | -6.714317985 | 0.034169197 |
| ENSG00000167077 | *MEI1* | -6.717291048 | 5.22E-05 |
| ENSG00000115468 | *EFHD1* | -6.719939395 | 9.44E-11 |
| ENSG00000010932 | *FMO1* | -6.724692586 | 0.026266073 |
| ENSG00000198771 | *RCSD1* | -6.72520757 | 4.77E-09 |
| ENSG00000224982 | *TMEM233* | -6.726786465 | 0.042463572 |
| ENSG00000225194 | *LINC00092* | -6.728625733 | 0.01529211 |
| ENSG00000183837 | *PNMA3* | -6.734123788 | 0.000105247 |
| ENSG00000225684 | *FAM225B* | -6.741787061 | 0.049993785 |
| ENSG00000140459 | *CYP11A1* | -6.749131016 | 0.005544598 |
| ENSG00000149131 | *SERPING1* | -6.751574001 | 3.91E-11 |
| ENSG00000231246 | *RP5-965F6.2* | -6.755451248 | 0.026034112 |
| ENSG00000111886 | *GABRR2* | -6.756577862 | 0.039128222 |
| ENSG00000042832 | *TG* | -6.756989686 | 6.84E-05 |
| ENSG00000228798 | *AP000473.5* | -6.757064664 | 0.018279944 |
| ENSG00000272168 | *CASC15* | -6.757346059 | 0.000422022 |
| ENSG00000170369 | *CST2* | -6.762548683 | 0.030375288 |
| ENSG00000170801 | *HTRA3* | -6.763126006 | 9.32E-09 |
| ENSG00000091831 | *ESR1* | -6.768515022 | 3.81E-10 |
| ENSG00000167483 | *FAM129C* | -6.775918985 | 0.000262182 |
| ENSG00000152092 | *ASTN1* | -6.780407869 | 0.001348631 |
| ENSG00000166091 | *CMTM5* | -6.781940112 | 0.01091715 |
| ENSG00000110799 | *VWF* | -6.784753897 | 2.27E-12 |
| ENSG00000169495 | *HTRA4* | -6.78529895 | 0.044170216 |
| ENSG00000170390 | *DCLK2* | -6.789609768 | 5.67E-09 |
| ENSG00000158714 | *SLAMF8* | -6.793418076 | 4.14E-10 |
| ENSG00000160097 | *FNDC5* | -6.795734968 | 4.87E-05 |
| ENSG00000180061 | *TMEM150B* | -6.797415996 | 0.023731255 |
| ENSG00000170703 | *TTLL6* | -6.79961317 | 0.04935804 |
| ENSG00000248890 | *HHIP-AS1* | -6.800301792 | 0.002288645 |
| ENSG00000150051 | *MKX* | -6.801005027 | 6.40E-07 |
| ENSG00000022267 | *FHL1* | -6.80366654 | 8.34E-10 |
| ENSG00000248587 | *GDNF-AS1* | -6.806180362 | 0.002667108 |
| ENSG00000144891 | *AGTR1* | -6.813367482 | 0.02747065 |
| ENSG00000250942 | *ENPP7P11* | -6.813886462 | 0.034849469 |
| ENSG00000227954 | *TARID* | -6.817510764 | 0.00509063 |
| ENSG00000198838 | *RYR3* | -6.822024346 | 6.92E-08 |
| ENSG00000150510 | *FAM124A* | -6.822753132 | 2.70E-08 |
| ENSG00000183662 | *FAM19A1* | -6.827558819 | 0.035868126 |
| ENSG00000255197 | *RP11-750H9.5* | -6.840431617 | 0.033156243 |
| ENSG00000179915 | *NRXN1* | -6.841029592 | 0.009267924 |
| ENSG00000169026 | *MFSD7* | -6.845517128 | 8.10E-06 |
| ENSG00000169507 | *SLC38A11* | -6.859365792 | 3.94E-07 |
| ENSG00000156103 | *MMP16* | -6.860174661 | 0.015132333 |
| ENSG00000128482 | *RNF112* | -6.862909447 | 3.49E-11 |
| ENSG00000145708 | *CRHBP* | -6.868487972 | 0.037163679 |
| ENSG00000266088 | *RP5-1028K7.2* | -6.869264002 | 0.002537994 |
| ENSG00000127074 | *RGS13* | -6.87023416 | 0.017870864 |
| ENSG00000130822 | *PNCK* | -6.872486484 | 1.34E-18 |
| ENSG00000125735 | *TNFSF14* | -6.873997254 | 0.002144621 |
| ENSG00000095970 | *TREM2* | -6.875259809 | 0.003705498 |
| ENSG00000101134 | *DOK5* | -6.877437002 | 0.013821342 |
| ENSG00000240219 | *RP11-430C7.5* | -6.878343036 | 0.00386123 |
| ENSG00000074047 | *GLI2* | -6.879718672 | 3.58E-05 |
| ENSG00000171101 | *SIGLEC17P* | -6.880041785 | 0.017479268 |
| ENSG00000170577 | *SIX2* | -6.888050628 | 8.95E-05 |
| ENSG00000241163 | *LINC00877* | -6.893076957 | 0.023415279 |
| ENSG00000161649 | *CD300LG* | -6.897733821 | 0.017279158 |
| ENSG00000140795 | *MYLK3* | -6.900610751 | 0.008834362 |
| ENSG00000164488 | *DACT2* | -6.901261044 | 0.003544316 |
| ENSG00000225400 | *RAB28P5* | -6.90746514 | 0.015350771 |
| ENSG00000266378 | *RP11-214O1.3* | -6.908644 | 0.035613791 |
| ENSG00000088320 | *REM1* | -6.915451805 | 0.001877847 |
| ENSG00000164616 | *FBXL21* | -6.915825441 | 0.012954032 |
| ENSG00000226306 | *NPY6R* | -6.917315891 | 0.013395734 |
| ENSG00000258837 | *CTD-2566J3.1* | -6.923404722 | 0.016940227 |
| ENSG00000010319 | *SEMA3G* | -6.925326751 | 1.78E-06 |
| ENSG00000131378 | *RFTN1* | -6.928458087 | 5.02E-08 |
| ENSG00000133116 | *KL* | -6.928513326 | 7.62E-05 |
| ENSG00000129467 | *ADCY4* | -6.929903725 | 4.09E-18 |
| ENSG00000155816 | *FMN2* | -6.929952639 | 0.001264115 |
| ENSG00000272511 | *RP11-180N14.1* | -6.930782891 | 0.011366304 |
| ENSG00000101265 | *RASSF2* | -6.931726625 | 6.32E-07 |
| ENSG00000230500 | *MKX-AS1* | -6.934549676 | 0.007988105 |
| ENSG00000109927 | *TECTA* | -6.934801873 | 0.000812146 |
| ENSG00000139220 | *PPFIA2* | -6.937902361 | 0.002460322 |
| ENSG00000122367 | *LDB3* | -6.944572418 | 1.12E-18 |
| ENSG00000204065 | *TCEAL5* | -6.953435266 | 0.004502415 |
| ENSG00000184682 | *PRR33* | -6.959940927 | 0.026413703 |
| ENSG00000268758 | *ADGRE4P* | -6.961593035 | 0.04984591 |
| ENSG00000188452 | *CERKL* | -6.970255309 | 0.000261874 |
| ENSG00000176049 | *JAKMIP2* | -6.972795421 | 0.011598271 |
| ENSG00000107742 | *SPOCK2* | -6.973552112 | 2.85E-06 |
| ENSG00000147138 | *GPR174* | -6.974109668 | 0.041932241 |
| ENSG00000010295 | *IFFO1* | -6.979267202 | 1.19E-14 |
| ENSG00000182230 | *FAM153B* | -6.981460385 | 4.96E-10 |
| ENSG00000123610 | *TNFAIP6* | -6.984087269 | 0.0231492 |
| ENSG00000182168 | *UNC5C* | -6.98494605 | 1.39E-05 |
| ENSG00000162894 | *FCMR* | -6.986505648 | 3.55E-07 |
| ENSG00000269113 | *TRABD2B* | -6.988563341 | 1.25E-05 |
| ENSG00000106819 | *ASPN* | -6.989295764 | 0.001240559 |
| ENSG00000272463 | *RP11-532F6.3* | -6.994365001 | 0.01109472 |
| ENSG00000078549 | *ADCYAP1R1* | -6.994826242 | 0.002613139 |
| ENSG00000069122 | *ADGRF5* | -6.995328818 | 8.90E-09 |
| ENSG00000152760 | *TCTEX1D1* | -6.996406405 | 0.040573487 |
| ENSG00000259881 | *RP11-830F9.5* | -6.998823211 | 0.003166701 |
| ENSG00000140287 | *HDC* | -7.007791795 | 2.90E-06 |
| ENSG00000123500 | *COL10A1* | -7.008835613 | 0.001514435 |
| ENSG00000143226 | *FCGR2A* | -7.009481768 | 3.65E-09 |
| ENSG00000245812 | *RP11-175K6.1* | -7.010636678 | 0.03800468 |
| ENSG00000162669 | *HFM1* | -7.014085384 | 0.003866102 |
| ENSG00000248309 | *MEF2C-AS1* | -7.018992794 | 0.014283869 |
| ENSG00000232414 | *CYP21A2* | -7.023866618 | 0.03870564 |
| ENSG00000198223 | *CSF2RA* | -7.030810344 | 9.10E-07 |
| ENSG00000160838 | *LRRC71* | -7.03477358 | 0.020172448 |
| ENSG00000092051 | *JPH4* | -7.040612884 | 9.10E-12 |
| ENSG00000152128 | *TMEM163* | -7.041200452 | 0.008137393 |
| ENSG00000085563 | *ABCB1* | -7.045031966 | 3.16E-10 |
| ENSG00000271579 | *RP11-116D17.3* | -7.04567711 | 0.021861715 |
| ENSG00000131386 | *GALNT15* | -7.053216875 | 0.00303129 |
| ENSG00000102837 | *OLFM4* | -7.053288739 | 0.018566874 |
| ENSG00000173702 | *MUC13* | -7.058331443 | 0.013784159 |
| ENSG00000082684 | *SEMA5B* | -7.058364808 | 0.02711559 |
| ENSG00000080224 | *EPHA6* | -7.058769305 | 0.015268743 |
| ENSG00000229056 | *AC020571.3* | -7.061466888 | 0.002379804 |
| ENSG00000028277 | *POU2F2* | -7.064972694 | 6.41E-07 |
| ENSG00000169744 | *LDB2* | -7.065268334 | 2.91E-08 |
| ENSG00000077264 | *PAK3* | -7.066525582 | 5.58E-06 |
| ENSG00000270547 | *LINC01235* | -7.06766817 | 0.005698555 |
| ENSG00000077616 | *NAALAD2* | -7.069086236 | 0.001162461 |
| ENSG00000271833 | *RP11-356B19.11* | -7.069689765 | 0.010260048 |
| ENSG00000228214 | *LINC00693* | -7.06980887 | 0.003938105 |
| ENSG00000169442 | *CD52* | -7.078621361 | 5.82E-08 |
| ENSG00000267121 | *CTD-2020K17.1* | -7.088552156 | 2.80E-06 |
| ENSG00000110848 | *CD69* | -7.099035392 | 2.56E-07 |
| ENSG00000197520 | *FAM177B* | -7.101497055 | 0.004418336 |
| ENSG00000129167 | *TPH1* | -7.10289286 | 0.006036797 |
| ENSG00000112214 | *FHL5* | -7.103012092 | 0.011497068 |
| ENSG00000164591 | *MYOZ3* | -7.110268304 | 9.17E-05 |
| ENSG00000106538 | *RARRES2* | -7.113150236 | 7.26E-11 |
| ENSG00000197415 | *VEPH1* | -7.113324235 | 0.009343219 |
| ENSG00000100427 | *MLC1* | -7.113928462 | 0.002030395 |
| ENSG00000171777 | *RASGRP4* | -7.116060241 | 0.002452122 |
| ENSG00000213949 | *ITGA1* | -7.121507503 | 2.11E-10 |
| ENSG00000128656 | *CHN1* | -7.121949319 | 2.96E-08 |
| ENSG00000168079 | *SCARA5* | -7.124238771 | 0.000432788 |
| ENSG00000128917 | *DLL4* | -7.12779067 | 1.71E-09 |
| ENSG00000263639 | *MSMB* | -7.130884785 | 0.023376675 |
| ENSG00000105137 | *SYDE1* | -7.135372909 | 9.20E-09 |
| ENSG00000267532 | *MIR497HG* | -7.135494286 | 0.001925281 |
| ENSG00000143195 | *ILDR2* | -7.135774093 | 1.05E-06 |
| ENSG00000167850 | *CD300C* | -7.137857435 | 0.015616859 |
| ENSG00000095637 | *SORBS1* | -7.138580291 | 3.90E-19 |
| ENSG00000277351 | *RP11-325L12.6* | -7.138953559 | 0.02080528 |
| ENSG00000260249 | *RP11-401P9.5* | -7.139051035 | 0.02589591 |
| ENSG00000253520 | *RP11-798K23.5* | -7.139118457 | 0.002760881 |
| ENSG00000138080 | *EMILIN1* | -7.140847638 | 7.56E-24 |
| ENSG00000170396 | *ZNF804A* | -7.141905727 | 0.002931546 |
| ENSG00000261371 | *PECAM1* | -7.142551933 | 0.008289417 |
| ENSG00000280726 | *PCAT19* | -7.149403063 | 0.015095591 |
| ENSG00000085741 | *WNT11* | -7.149922302 | 3.46E-06 |
| ENSG00000128918 | *ALDH1A2* | -7.150756192 | 1.12E-13 |
| ENSG00000170370 | *EMX2* | -7.151971897 | 0.028796541 |
| ENSG00000206195 | *DUXAP8* | -7.152409178 | 0.006839971 |
| ENSG00000183801 | *OLFML1* | -7.154012305 | 1.36E-09 |
| ENSG00000253379 | *RP11-1102P16.1* | -7.160140504 | 0.014355976 |
| ENSG00000258227 | *CLEC5A* | -7.161890336 | 0.031717159 |
| ENSG00000134917 | *ADAMTS8* | -7.16220368 | 2.81E-08 |
| ENSG00000065609 | *SNAP91* | -7.162539404 | 0.004064329 |
| ENSG00000139910 | *NOVA1* | -7.162656542 | 3.08E-07 |
| ENSG00000223561 | *AC003090.1* | -7.173504385 | 0.00129073 |
| ENSG00000280237 | *MIR4697HG* | -7.175534826 | 1.27E-09 |
| ENSG00000132554 | *RGS22* | -7.177786725 | 0.000677298 |
| ENSG00000225756 | *DBH-AS1* | -7.181315559 | 0.010709228 |
| ENSG00000255986 | *MT1JP* | -7.190917062 | 0.002725973 |
| ENSG00000156049 | *GNA14* | -7.191964101 | 1.28E-07 |
| ENSG00000233093 | *LINC00892* | -7.192752129 | 0.013391701 |
| ENSG00000170276 | *HSPB2* | -7.214600271 | 0.014359283 |
| ENSG00000181074 | *OR52N4* | -7.214716579 | 0.016111869 |
| ENSG00000204403 | *CASP12* | -7.215188897 | 0.024195779 |
| ENSG00000123342 | *MMP19* | -7.21527106 | 4.28E-07 |
| ENSG00000180044 | *C3orf80* | -7.2194548 | 0.049010095 |
| ENSG00000010610 | *CD4* | -7.22018446 | 5.48E-06 |
| ENSG00000175489 | *LRRC25* | -7.22289316 | 2.38E-07 |
| ENSG00000102755 | *FLT1* | -7.227257449 | 1.15E-08 |
| ENSG00000131831 | *RAI2* | -7.231386634 | 2.69E-12 |
| ENSG00000165084 | *C8orf34* | -7.234479227 | 0.008879591 |
| ENSG00000196664 | *TLR7* | -7.235501213 | 0.003816959 |
| ENSG00000171408 | *PDE7B* | -7.236077487 | 1.41E-05 |
| ENSG00000166428 | *PLD4* | -7.238248872 | 6.01E-05 |
| ENSG00000167664 | *TMIGD2* | -7.240653588 | 0.045070083 |
| ENSG00000169282 | *KCNAB1* | -7.241393576 | 7.79E-17 |
| ENSG00000267127 | *RP11-795F19.5* | -7.245043513 | 0.008451469 |
| ENSG00000039537 | *C6* | -7.245620503 | 0.020281561 |
| ENSG00000202538 | *RNU4-2* | -7.251461416 | 0.024662168 |
| ENSG00000187498 | *COL4A1* | -7.255056188 | 3.99E-08 |
| ENSG00000120645 | *IQSEC3* | -7.255207279 | 0.003338851 |
| ENSG00000168229 | *PTGDR* | -7.256043845 | 0.01114036 |
| ENSG00000260105 | *AOC4P* | -7.268509746 | 0.02160946 |
| ENSG00000147647 | *DPYS* | -7.273084858 | 0.032902007 |
| ENSG00000255366 | *RP11-1134I14.8* | -7.277827184 | 0.008657011 |
| ENSG00000185477 | *GPRIN3* | -7.280520282 | 3.84E-05 |
| ENSG00000117594 | *HSD11B1* | -7.281272127 | 4.23E-07 |
| ENSG00000254415 | *SIGLEC14* | -7.286179444 | 0.001820753 |
| ENSG00000130035 | *GALNT8* | -7.29068285 | 0.027312571 |
| ENSG00000218052 | *ADAMTS7P4* | -7.290917262 | 0.029035674 |
| ENSG00000181856 | *SLC2A4* | -7.291824587 | 4.90E-17 |
| ENSG00000100060 | *MFNG* | -7.293572639 | 6.28E-10 |
| ENSG00000100285 | *NEFH* | -7.29564482 | 1.11E-06 |
| ENSG00000125245 | *GPR18* | -7.296276007 | 0.004926875 |
| ENSG00000278301 | *GRAMD4P3* | -7.296950205 | 0.011466988 |
| ENSG00000182836 | *PLCXD3* | -7.298206821 | 0.005947876 |
| ENSG00000130052 | *STARD8* | -7.300145417 | 2.67E-13 |
| ENSG00000171840 | *NINJ2* | -7.303532537 | 0.000539478 |
| ENSG00000188848 | *BEND4* | -7.307046513 | 0.008003672 |
| ENSG00000203685 | *C1orf95* | -7.307393474 | 8.10E-10 |
| ENSG00000189350 | *FAM179A* | -7.312789322 | 0.000633112 |
| ENSG00000187889 | *C1orf168* | -7.313321982 | 0.025884023 |
| ENSG00000127533 | *F2RL3* | -7.315747843 | 0.014758861 |
| ENSG00000086288 | *NME8* | -7.317369583 | 0.011729677 |
| ENSG00000215861 | *WI2-1896O14.1* | -7.318358687 | 0.000182636 |
| ENSG00000108932 | *SLC16A6* | -7.319450103 | 0.023265917 |
| ENSG00000273837 | *LLNLR-470E3.1* | -7.322204318 | 0.042253578 |
| ENSG00000260135 | *RP11-212I21.2* | -7.329656501 | 0.02203372 |
| ENSG00000227357 | *HLA-DRB4* | -7.330717777 | 0.030686932 |
| ENSG00000227619 | *RP11-492E3.2* | -7.336077082 | 0.01468915 |
| ENSG00000275326 | *NOSTRIN* | -7.336440306 | 0.010215552 |
| ENSG00000161835 | *GRASP* | -7.336545844 | 1.29E-15 |
| ENSG00000142583 | *SLC2A5* | -7.338917249 | 3.77E-14 |
| ENSG00000243836 | *WDR86-AS1* | -7.339450317 | 0.034575731 |
| ENSG00000153012 | *LGI2* | -7.340960909 | 0.001278599 |
| ENSG00000189058 | *APOD* | -7.34382709 | 1.17E-25 |
| ENSG00000127329 | *PTPRB* | -7.344598697 | 9.32E-09 |
| ENSG00000113494 | *PRLR* | -7.347981745 | 0.004887964 |
| ENSG00000135424 | *ITGA7* | -7.350421781 | 1.41E-18 |
| ENSG00000267272 | *LINC01140* | -7.354753151 | 0.005139013 |
| ENSG00000198286 | *CARD11* | -7.354908013 | 4.24E-05 |
| ENSG00000145362 | *ANK2* | -7.364038914 | 7.44E-09 |
| ENSG00000112319 | *EYA4* | -7.365750495 | 0.02200647 |
| ENSG00000261625 | *RP11-554A11.4* | -7.366979658 | 0.003086023 |
| ENSG00000172349 | *IL16* | -7.371629846 | 1.24E-09 |
| ENSG00000121068 | *TBX2* | -7.374344172 | 2.76E-09 |
| ENSG00000164483 | *SAMD3* | -7.376052578 | 0.01109472 |
| ENSG00000176083 | *ZNF683* | -7.385564215 | 0.038492211 |
| ENSG00000126895 | *AVPR2* | -7.393855523 | 0.008643696 |
| ENSG00000132639 | *SNAP25* | -7.395197837 | 1.19E-06 |
| ENSG00000053328 | *METTL24* | -7.402399539 | 0.005176302 |
| ENSG00000131203 | *IDO1* | -7.40270058 | 0.000846742 |
| ENSG00000205436 | *EXOC3L4* | -7.403342294 | 0.000177754 |
| ENSG00000140368 | *PSTPIP1* | -7.407986775 | 6.02E-08 |
| ENSG00000185818 | *NAT8L* | -7.410130606 | 0.000137826 |
| ENSG00000169291 | *SHE* | -7.41049824 | 2.40E-06 |
| ENSG00000131459 | *GFPT2* | -7.414095708 | 3.99E-12 |
| ENSG00000204131 | *NHSL2* | -7.424721592 | 5.28E-08 |
| ENSG00000135744 | *AGT* | -7.428716038 | 5.44E-08 |
| ENSG00000112280 | *COL9A1* | -7.428905505 | 0.011624271 |
| ENSG00000183023 | *SLC8A1* | -7.431293158 | 3.20E-11 |
| ENSG00000167434 | *CA4* | -7.431505954 | 0.003361775 |
| ENSG00000143768 | *LEFTY2* | -7.432762966 | 0.011012867 |
| ENSG00000221972 | *C3orf36* | -7.435766486 | 0.044370674 |
| ENSG00000150681 | *RGS18* | -7.437126 | 0.001490784 |
| ENSG00000120729 | *MYOT* | -7.439504646 | 0.04017881 |
| ENSG00000224367 | *OACYLP* | -7.440912887 | 0.009203958 |
| ENSG00000179542 | *SLITRK4* | -7.449680696 | 0.012094495 |
| ENSG00000163106 | *HPGDS* | -7.449918044 | 0.001806059 |
| ENSG00000163519 | *TRAT1* | -7.452549634 | 0.019600121 |
| ENSG00000242258 | *LINC00996* | -7.461683455 | 0.038801565 |
| ENSG00000198732 | *SMOC1* | -7.462427949 | 4.71E-15 |
| ENSG00000136099 | *PCDH8* | -7.463661818 | 0.000407931 |
| ENSG00000115607 | *IL18RAP* | -7.465762615 | 0.002827061 |
| ENSG00000170962 | *PDGFD* | -7.466174957 | 1.56E-07 |
| ENSG00000109099 | *PMP22* | -7.469448835 | 4.28E-12 |
| ENSG00000137809 | *ITGA11* | -7.470627533 | 3.06E-07 |
| ENSG00000250208 | *FZD10-AS1* | -7.47469987 | 2.02E-09 |
| ENSG00000166825 | *ANPEP* | -7.475232468 | 3.04E-14 |
| ENSG00000158488 | *CD1E* | -7.482951392 | 0.002239556 |
| ENSG00000115353 | *TACR1* | -7.484033327 | 0.004478168 |
| ENSG00000172156 | *CCL11* | -7.48819078 | 0.015721429 |
| ENSG00000279805 | *CTA-212A2.1* | -7.493346543 | 0.006950715 |
| ENSG00000174004 | *NRROS* | -7.495659128 | 1.66E-05 |
| ENSG00000112183 | *RBM24* | -7.499414483 | 1.83E-05 |
| ENSG00000186265 | *BTLA* | -7.5067181 | 0.004990508 |
| ENSG00000183346 | *C10orf107* | -7.509385317 | 0.000179364 |
| ENSG00000185559 | *DLK1* | -7.511745864 | 0.034652896 |
| ENSG00000161381 | *PLXDC1* | -7.512531294 | 6.69E-15 |
| ENSG00000227039 | *ITGB2-AS1* | -7.515961244 | 1.11E-05 |
| ENSG00000104894 | *CD37* | -7.51920001 | 9.86E-09 |
| ENSG00000159307 | *SCUBE1* | -7.519756212 | 0.002555703 |
| ENSG00000189233 | *NUGGC* | -7.521172717 | 0.008802039 |
| ENSG00000080709 | *KCNN2* | -7.523347702 | 0.001075656 |
| ENSG00000220008 | *LINGO3* | -7.523618891 | 0.036591435 |
| ENSG00000163710 | *PCOLCE2* | -7.527679212 | 2.66E-08 |
| ENSG00000169508 | *GPR183* | -7.529979974 | 6.27E-08 |
| ENSG00000174407 | *MIR1-1HG* | -7.530653181 | 0.010452656 |
| ENSG00000170927 | *PKHD1* | -7.536114897 | 0.006054199 |
| ENSG00000169418 | *NPR1* | -7.544893843 | 4.14E-07 |
| ENSG00000002746 | *HECW1* | -7.545377863 | 0.004003667 |
| ENSG00000101331 | *CCM2L* | -7.545448268 | 0.00045097 |
| ENSG00000177519 | *RPRM* | -7.551945413 | 8.58E-08 |
| ENSG00000118946 | *PCDH17* | -7.553073515 | 0.003736002 |
| ENSG00000265142 | *MIR133A1HG* | -7.556545553 | 0.011647489 |
| ENSG00000179954 | *SSC5D* | -7.556554096 | 8.78E-07 |
| ENSG00000136842 | *TMOD1* | -7.557829394 | 1.58E-09 |
| ENSG00000162551 | *ALPL* | -7.562127775 | 3.21E-07 |
| ENSG00000144481 | *TRPM8* | -7.562701982 | 1.61E-05 |
| ENSG00000219438 | *FAM19A5* | -7.569238804 | 0.000192558 |
| ENSG00000151650 | *VENTX* | -7.570398177 | 0.004928554 |
| ENSG00000149380 | *P4HA3* | -7.572293242 | 0.000436665 |
| ENSG00000133063 | *CHIT1* | -7.572830043 | 4.46E-07 |
| ENSG00000121898 | *CPXM2* | -7.575550716 | 1.62E-10 |
| ENSG00000166573 | *GALR1* | -7.576700405 | 0.013671029 |
| ENSG00000163638 | *ADAMTS9* | -7.577574353 | 1.97E-08 |
| ENSG00000159753 | *RLTPR* | -7.577866594 | 6.49E-06 |
| ENSG00000236625 | *C4B* | -7.581465876 | 0.026630715 |
| ENSG00000128052 | *KDR* | -7.581675559 | 1.27E-09 |
| ENSG00000087250 | *MT3* | -7.585928467 | 0.0029172 |
| ENSG00000165349 | *SLC7A3* | -7.588090395 | 0.004464008 |
| ENSG00000146192 | *FGD2* | -7.594082611 | 1.01E-11 |
| ENSG00000258498 | *DIO3OS* | -7.595589761 | 1.15E-12 |
| ENSG00000109063 | *MYH3* | -7.603422501 | 2.61E-08 |
| ENSG00000196092 | *PAX5* | -7.603712035 | 0.001076929 |
| ENSG00000148677 | *ANKRD1* | -7.609289294 | 0.032736668 |
| ENSG00000154678 | *PDE1C* | -7.611438605 | 0.000348515 |
| ENSG00000279526 | *AC011239.2* | -7.611721321 | 0.000800284 |
| ENSG00000138615 | *CILP* | -7.613075626 | 0.000202357 |
| ENSG00000172543 | *CTSW* | -7.614794323 | 4.01E-05 |
| ENSG00000110675 | *ELMOD1* | -7.621073977 | 0.022302445 |
| ENSG00000165495 | *PKNOX2* | -7.622031868 | 1.52E-07 |
| ENSG00000241386 | *HLA-DOB* | -7.627239952 | 0.048932685 |
| ENSG00000120903 | *CHRNA2* | -7.629274337 | 0.00415491 |
| ENSG00000241641 | *RPS23P6* | -7.629696544 | 0.007362702 |
| ENSG00000108924 | *HLF* | -7.631861319 | 2.69E-13 |
| ENSG00000172572 | *PDE3A* | -7.645791793 | 3.52E-12 |
| ENSG00000198821 | *CD247* | -7.652233281 | 6.25E-07 |
| ENSG00000101096 | *NFATC2* | -7.655394265 | 4.90E-09 |
| ENSG00000131055 | *COX4I2* | -7.658738206 | 0.00079841 |
| ENSG00000271009 | *RP11-346C20.3* | -7.665520996 | 0.004290356 |
| ENSG00000166317 | *SYNPO2L* | -7.667337864 | 0.00112028 |
| ENSG00000149564 | *ESAM* | -7.667430234 | 2.32E-11 |
| ENSG00000164188 | *RANBP3L* | -7.667679758 | 0.000981631 |
| ENSG00000145087 | *STXBP5L* | -7.673965567 | 0.00041077 |
| ENSG00000092009 | *CMA1* | -7.678304812 | 0.007641925 |
| ENSG00000163618 | *CADPS* | -7.679321487 | 0.000409256 |
| ENSG00000146352 | *CLVS2* | -7.681629081 | 0.000312096 |
| ENSG00000142449 | *FBN3* | -7.681638379 | 0.001410742 |
| ENSG00000077274 | *CAPN6* | -7.681825455 | 1.79E-06 |
| ENSG00000149534 | *MS4A2* | -7.686894717 | 0.000436516 |
| ENSG00000244575 | *IGKV1-27* | -7.690039702 | 0.049001659 |
| ENSG00000182492 | *BGN* | -7.695238961 | 9.13E-15 |
| ENSG00000043462 | *LCP2* | -7.699243124 | 7.26E-11 |
| ENSG00000173918 | *C1QTNF1* | -7.699981832 | 1.48E-11 |
| ENSG00000254507 | *RP11-481A20.10* | -7.701270674 | 0.011884171 |
| ENSG00000156427 | *FGF18* | -7.702471723 | 0.000705198 |
| ENSG00000182132 | *KCNIP1* | -7.706604587 | 0.006300768 |
| ENSG00000211659 | *IGLV3-25* | -7.708975825 | 0.003569644 |
| ENSG00000223350 | *IGLV9-49* | -7.712467088 | 0.042894528 |
| ENSG00000269220 | *LINC00528* | -7.712611323 | 0.046310719 |
| ENSG00000185046 | *ANKS1B* | -7.714687793 | 0.000870394 |
| ENSG00000037280 | *FLT4* | -7.717051315 | 5.75E-10 |
| ENSG00000204442 | *FAM155A* | -7.720698726 | 0.000975462 |
| ENSG00000165178 | *NCF1C* | -7.722368415 | 1.52E-05 |
| ENSG00000147570 | *DNAJC5B* | -7.737817688 | 0.006937323 |
| ENSG00000181036 | *FCRL6* | -7.744102351 | 0.006028759 |
| ENSG00000237181 | *AC147651.4* | -7.746334317 | 0.008903807 |
| ENSG00000112195 | *TREML2* | -7.746457799 | 0.013240211 |
| ENSG00000153563 | *CD8A* | -7.748879715 | 3.24E-09 |
| ENSG00000134853 | *PDGFRA* | -7.753647887 | 6.55E-10 |
| ENSG00000247774 | *PCED1B-AS1* | -7.755209053 | 3.06E-07 |
| ENSG00000168546 | *GFRA2* | -7.756619691 | 0.002247622 |
| ENSG00000175356 | *SCUBE2* | -7.766415788 | 3.43E-11 |
| ENSG00000236304 | *AP001189.4* | -7.771309223 | 0.030522517 |
| ENSG00000182326 | *C1S* | -7.782407032 | 5.90E-22 |
| ENSG00000136244 | *IL6* | -7.783250076 | 0.001107511 |
| ENSG00000157570 | *TSPAN18* | -7.786977128 | 1.91E-14 |
| ENSG00000163600 | *ICOS* | -7.787641431 | 0.004422068 |
| ENSG00000137507 | *LRRC32* | -7.789533687 | 9.70E-16 |
| ENSG00000078053 | *AMPH* | -7.791549705 | 0.000297749 |
| ENSG00000169896 | *ITGAM* | -7.794641273 | 6.07E-07 |
| ENSG00000184005 | *ST6GALNAC3* | -7.812559259 | 0.003738043 |
| ENSG00000154553 | *PDLIM3* | -7.820987905 | 4.06E-12 |
| ENSG00000106823 | *ECM2* | -7.828043581 | 9.55E-08 |
| ENSG00000122756 | *CNTFR* | -7.831024888 | 0.000466633 |
| ENSG00000251039 | *IGKV2D-40* | -7.836200971 | 0.039317735 |
| ENSG00000110876 | *SELPLG* | -7.837034858 | 4.52E-10 |
| ENSG00000254851 | *RP11-109L13.1* | -7.837245494 | 0.001781797 |
| ENSG00000211772 | *TRBC2* | -7.838033106 | 0.006055352 |
| ENSG00000258168 | *RP11-588H23.3* | -7.840390362 | 0.015004412 |
| ENSG00000227418 | *PCGEM1* | -7.841367149 | 0.002458051 |
| ENSG00000255026 | *RP11-326C3.2* | -7.848686469 | 1.83E-05 |
| ENSG00000107796 | *ACTA2* | -7.855835725 | 3.95E-24 |
| ENSG00000272872 | *LL22NC03-N14H11.1* | -7.857081765 | 0.00800837 |
| ENSG00000133687 | *TMTC1* | -7.858041662 | 8.86E-10 |
| ENSG00000162383 | *SLC1A7* | -7.861833822 | 0.000778261 |
| ENSG00000151892 | *GFRA1* | -7.866251184 | 1.56E-07 |
| ENSG00000121594 | *CD80* | -7.869123276 | 0.017794697 |
| ENSG00000047617 | *ANO2* | -7.875210411 | 0.001633107 |
| ENSG00000253308 | *RP1-170O19.17* | -7.87638202 | 0.000492761 |
| ENSG00000059804 | *SLC2A3* | -7.877090406 | 3.35E-12 |
| ENSG00000196188 | *CTSE* | -7.877362381 | 0.007090279 |
| ENSG00000143185 | *XCL2* | -7.881073593 | 0.011842474 |
| ENSG00000071991 | *CDH19* | -7.881219735 | 0.002042416 |
| ENSG00000171596 | *NMUR1* | -7.883133192 | 0.000528367 |
| ENSG00000258314 | *CTD-2314B22.1* | -7.885333579 | 0.00460367 |
| ENSG00000117154 | *IGSF21* | -7.886828341 | 0.000698409 |
| ENSG00000262370 | *RP11-473M20.9* | -7.887671447 | 0.00063048 |
| ENSG00000142512 | *SIGLEC10* | -7.887770686 | 3.17E-09 |
| ENSG00000130201 | *EXOC3L2* | -7.892495767 | 0.000127263 |
| ENSG00000168421 | *RHOH* | -7.897777193 | 2.46E-07 |
| ENSG00000134020 | *PEBP4* | -7.901771677 | 3.77E-05 |
| ENSG00000151490 | *PTPRO* | -7.902222735 | 0.001459101 |
| ENSG00000186198 | *SLC51B* | -7.902751604 | 0.001546214 |
| ENSG00000277734 | *TRAC* | -7.912496081 | 1.60E-08 |
| ENSG00000197540 | *GZMM* | -7.913126901 | 0.001170437 |
| ENSG00000163873 | *GRIK3* | -7.920277158 | 0.002360264 |
| ENSG00000185811 | *IKZF1* | -7.923304821 | 4.46E-10 |
| ENSG00000234883 | *MIR155HG* | -7.923452505 | 0.002583373 |
| ENSG00000009694 | *TENM1* | -7.931637678 | 4.70E-05 |
| ENSG00000280977 | *Z93930.1* | -7.932687031 | 0.000595699 |
| ENSG00000049249 | *TNFRSF9* | -7.936438507 | 0.025978016 |
| ENSG00000241684 | *ADAMTS9-AS2* | -7.937636386 | 0.000128073 |
| ENSG00000253405 | *EVX1-AS* | -7.94029186 | 0.002436374 |
| ENSG00000174576 | *NPAS4* | -7.941117888 | 0.000411523 |
| ENSG00000108950 | *FAM20A* | -7.94282897 | 1.19E-06 |
| ENSG00000174946 | *GPR171* | -7.953416724 | 0.000793211 |
| ENSG00000167083 | *GNGT2* | -7.953667565 | 0.000938688 |
| ENSG00000025423 | *HSD17B6* | -7.954600075 | 5.27E-13 |
| ENSG00000135472 | *FAIM2* | -7.954684666 | 3.87E-09 |
| ENSG00000113749 | *HRH2* | -7.959735553 | 0.002673741 |
| ENSG00000172346 | *CSDC2* | -7.970755261 | 1.11E-09 |
| ENSG00000229847 | *EMX2OS* | -7.984202777 | 0.005221024 |
| ENSG00000205056 | *RP11-693J15.5* | -7.984774672 | 0.006754773 |
| ENSG00000211653 | *IGLV1-40* | -7.986134033 | 0.013261702 |
| ENSG00000135144 | *DTX1* | -7.987317034 | 0.000114908 |
| ENSG00000166147 | *FBN1* | -7.989443098 | 1.03E-11 |
| ENSG00000184113 | *CLDN5* | -7.9968567 | 4.06E-12 |
| ENSG00000267369 | *RP11-1094M14.8* | -8.008107808 | 0.003866102 |
| ENSG00000080573 | *COL5A3* | -8.013016327 | 1.76E-11 |
| ENSG00000140285 | *FGF7* | -8.013267543 | 4.74E-10 |
| ENSG00000204165 | *CXorf65* | -8.016177247 | 0.007716949 |
| ENSG00000130300 | *PLVAP* | -8.016359457 | 3.02E-12 |
| ENSG00000271178 | *IGHV3OR16-13* | -8.018511894 | 0.028525003 |
| ENSG00000122223 | *CD244* | -8.020525085 | 0.003786712 |
| ENSG00000163884 | *KLF15* | -8.02402082 | 6.96E-09 |
| ENSG00000122862 | *SRGN* | -8.02570839 | 8.09E-15 |
| ENSG00000168621 | *GDNF* | -8.026787808 | 0.000230423 |
| ENSG00000179362 | *HMGN2P46* | -8.032830745 | 5.46E-05 |
| ENSG00000158022 | *TRIM63* | -8.034184542 | 0.000724136 |
| ENSG00000120156 | *TEK* | -8.035984704 | 0.000150077 |
| ENSG00000108700 | *CCL8* | -8.043462679 | 0.031031212 |
| ENSG00000100351 | *GRAP2* | -8.044264188 | 0.000175196 |
| ENSG00000177301 | *KCNA2* | -8.057872703 | 0.000649432 |
| ENSG00000162618 | *ADGRL4* | -8.062708833 | 2.09E-07 |
| ENSG00000235997 | *AC109642.1* | -8.063238804 | 0.004167685 |
| ENSG00000266524 | *GDF10* | -8.065969549 | 0.00167209 |
| ENSG00000249751 | *ECSCR* | -8.070347166 | 0.001029026 |
| ENSG00000224137 | *AC079767.4* | -8.073412016 | 0.032643186 |
| ENSG00000160013 | *PTGIR* | -8.080917851 | 0.004270254 |
| ENSG00000146215 | *CRIP3* | -8.083379372 | 2.69E-05 |
| ENSG00000168004 | *HRASLS5* | -8.087061843 | 0.00045097 |
| ENSG00000151702 | *FLI1* | -8.090838597 | 8.51E-08 |
| ENSG00000198400 | *NTRK1* | -8.092904556 | 0.000530912 |
| ENSG00000204161 | *C10orf128* | -8.096363911 | 6.30E-07 |
| ENSG00000140807 | *NKD1* | -8.096684216 | 3.59E-11 |
| ENSG00000126264 | *HCST* | -8.096857901 | 0.000221028 |
| ENSG00000270093 | *AP000473.8* | -8.098585901 | 0.000582834 |
| ENSG00000086205 | *FOLH1* | -8.102214135 | 3.80E-06 |
| ENSG00000265107 | *GJA5* | -8.102711407 | 0.000220915 |
| ENSG00000238121 | *LINC00426* | -8.105324433 | 0.000712075 |
| ENSG00000163827 | *LRRC2* | -8.112206834 | 0.001324062 |
| ENSG00000096088 | *PGC* | -8.121184401 | 0.000323528 |
| ENSG00000168995 | *SIGLEC7* | -8.122975078 | 0.001382611 |
| ENSG00000180549 | *FUT7* | -8.131567979 | 0.03762439 |
| ENSG00000244306 | *LINC01296* | -8.13239755 | 0.005037886 |
| ENSG00000166928 | *MS4A14* | -8.13254842 | 0.000368869 |
| ENSG00000116194 | *ANGPTL1* | -8.134033392 | 0.000248024 |
| ENSG00000233038 | *AC011899.9* | -8.134105743 | 0.001088852 |
| ENSG00000139970 | *RTN1* | -8.134666912 | 2.95E-05 |
| ENSG00000163508 | *EOMES* | -8.135952331 | 0.002019838 |
| ENSG00000172995 | *ARPP21* | -8.140864354 | 0.000127448 |
| ENSG00000256508 | *MRGPRF-AS1* | -8.14853171 | 0.000133708 |
| ENSG00000166831 | *RBPMS2* | -8.154221928 | 4.06E-12 |
| ENSG00000178175 | *ZNF366* | -8.155943795 | 0.002810998 |
| ENSG00000132840 | *BHMT2* | -8.159988969 | 1.88E-09 |
| ENSG00000178033 | *FAM26E* | -8.161364473 | 0.000524551 |
| ENSG00000152672 | *CLEC4F* | -8.166047318 | 0.000372386 |
| ENSG00000250360 | *CTD-2089N3.1* | -8.166101553 | 0.000481509 |
| ENSG00000267107 | *PCAT19* | -8.166132585 | 0.000202357 |
| ENSG00000162733 | *DDR2* | -8.168265313 | 1.25E-22 |
| ENSG00000251301 | *RP11-81H14.2* | -8.17389486 | 0.008660418 |
| ENSG00000101916 | *TLR8* | -8.175365306 | 0.001682246 |
| ENSG00000204851 | *PNMAL2* | -8.176230139 | 0.000154176 |
| ENSG00000163145 | *C1QTNF7* | -8.176793239 | 1.86E-05 |
| ENSG00000169031 | *COL4A3* | -8.17758768 | 1.29E-10 |
| ENSG00000113088 | *GZMK* | -8.17991462 | 0.00149001 |
| ENSG00000185565 | *LSAMP* | -8.184421055 | 5.43E-15 |
| ENSG00000211689 | *TRGC1* | -8.184903727 | 0.001452594 |
| ENSG00000143194 | *MAEL* | -8.18851853 | 0.000444238 |
| ENSG00000185985 | *SLITRK2* | -8.190756878 | 0.008671911 |
| ENSG00000225670 | *CADM3-AS1* | -8.19545738 | 0.000968768 |
| ENSG00000124701 | *APOBEC2* | -8.197406277 | 0.000500276 |
| ENSG00000007933 | *FMO3* | -8.200578226 | 0.000887986 |
| ENSG00000096996 | *IL12RB1* | -8.210303948 | 0.000143536 |
| ENSG00000121933 | *ADORA3* | -8.213092609 | 0.002676492 |
| ENSG00000127241 | *MASP1* | -8.217468197 | 5.51E-15 |
| ENSG00000102683 | *SGCG* | -8.219573096 | 0.000259054 |
| ENSG00000123496 | *IL13RA2* | -8.220480076 | 8.42E-05 |
| ENSG00000100626 | *GALNT16* | -8.226889788 | 1.60E-05 |
| ENSG00000281759 | *CTD-2007N20.1* | -8.228547807 | 0.011966541 |
| ENSG00000241666 | *RP3-455J7.4* | -8.235558369 | 0.002083334 |
| ENSG00000168427 | *KLHL30* | -8.237615663 | 0.000528546 |
| ENSG00000108001 | *EBF3* | -8.246642597 | 0.000425428 |
| ENSG00000069431 | *ABCC9* | -8.250241789 | 2.56E-12 |
| ENSG00000160307 | *S100B* | -8.254521566 | 6.23E-05 |
| ENSG00000178162 | *FAR2P2* | -8.263442826 | 3.46E-05 |
| ENSG00000123243 | *ITIH5* | -8.270282282 | 1.73E-26 |
| ENSG00000183798 | *EMILIN3* | -8.272626836 | 1.97E-08 |
| ENSG00000177238 | *TRIM72* | -8.273517829 | 0.003264982 |
| ENSG00000108691 | *CCL2* | -8.281941716 | 1.57E-10 |
| ENSG00000198851 | *CD3E* | -8.289297959 | 1.03E-09 |
| ENSG00000163815 | *CLEC3B* | -8.297577836 | 4.43E-05 |
| ENSG00000162706 | *CADM3* | -8.299142544 | 0.000661845 |
| ENSG00000175538 | *KCNE3* | -8.302023737 | 4.46E-05 |
| ENSG00000006638 | *TBXA2R* | -8.305381049 | 5.32E-05 |
| ENSG00000280429 | *AF001548.3* | -8.306191078 | 0.000131495 |
| ENSG00000126218 | *F10* | -8.310085582 | 6.02E-09 |
| ENSG00000188488 | *SERPINA5* | -8.311265228 | 0.000805012 |
| ENSG00000073861 | *TBX21* | -8.311271139 | 0.001251026 |
| ENSG00000170891 | *CYTL1* | -8.312225764 | 7.63E-05 |
| ENSG00000153283 | *CD96* | -8.32058085 | 1.49E-07 |
| ENSG00000276886 | *GREM1* | -8.322545126 | 0.001705456 |
| ENSG00000103569 | *AQP9* | -8.32317417 | 0.003874983 |
| ENSG00000240382 | *IGKV1-17* | -8.331272186 | 0.014296942 |
| ENSG00000173175 | *ADCY5* | -8.332255489 | 3.74E-16 |
| ENSG00000046889 | *PREX2* | -8.336231783 | 1.83E-05 |
| ENSG00000187922 | *LCN10* | -8.344787769 | 0.00050634 |
| ENSG00000144230 | *GPR17* | -8.346987577 | 0.001125819 |
| ENSG00000136160 | *EDNRB* | -8.352660001 | 3.27E-08 |
| ENSG00000106952 | *TNFSF8* | -8.353168738 | 0.000530542 |
| ENSG00000172322 | *CLEC12A* | -8.362059691 | 0.000363437 |
| ENSG00000111796 | *KLRB1* | -8.373529767 | 1.44E-06 |
| ENSG00000101098 | *RIMS4* | -8.375160846 | 0.000147522 |
| ENSG00000115523 | *GNLY* | -8.37638525 | 0.000521004 |
| ENSG00000142303 | *ADAMTS10* | -8.392790503 | 1.34E-07 |
| ENSG00000026751 | *SLAMF7* | -8.3964248 | 3.29E-07 |
| ENSG00000088992 | *TESC* | -8.400457057 | 2.61E-05 |
| ENSG00000007314 | *SCN4A* | -8.403875678 | 0.00202237 |
| ENSG00000182253 | *SYNM* | -8.415301824 | 8.86E-24 |
| ENSG00000197253 | *TPSB2* | -8.421445645 | 1.00E-09 |
| ENSG00000259834 | *RP11-284N8.3* | -8.426558165 | 0.001278599 |
| ENSG00000151572 | *ANO4* | -8.427348247 | 0.00045097 |
| ENSG00000235531 | *MSC-AS1* | -8.427675684 | 0.000413674 |
| ENSG00000271856 | *LINC01215* | -8.42995994 | 0.005267065 |
| ENSG00000237949 | *LINC00844* | -8.431662426 | 0.000357883 |
| ENSG00000179855 | *GIPC3* | -8.4399468 | 0.000136462 |
| ENSG00000197406 | *DIO3* | -8.44146894 | 0.000249576 |
| ENSG00000225783 | *MIAT* | -8.441701858 | 2.65E-06 |
| ENSG00000275575 | *PTP4A3* | -8.444791064 | 0.000119224 |
| ENSG00000179772 | *FOXS1* | -8.448359006 | 0.004225165 |
| ENSG00000182771 | *GRID1* | -8.44946629 | 0.000219482 |
| ENSG00000211448 | *DIO2* | -8.450751317 | 4.44E-12 |
| ENSG00000138722 | *MMRN1* | -8.452635108 | 0.000225609 |
| ENSG00000196329 | *GIMAP5* | -8.453231906 | 0.000179413 |
| ENSG00000102245 | *CD40LG* | -8.455467921 | 0.002694372 |
| ENSG00000170153 | *RNF150* | -8.456024379 | 2.95E-14 |
| ENSG00000198848 | *CES1* | -8.458424599 | 0.00011188 |
| ENSG00000251321 | *PCAT4* | -8.460366654 | 0.003135026 |
| ENSG00000128655 | *PDE11A* | -8.464730984 | 2.29E-05 |
| ENSG00000164619 | *BMPER* | -8.466512641 | 2.90E-07 |
| ENSG00000271811 | *RP1-79C4.4* | -8.473089475 | 0.000199665 |
| ENSG00000126860 | *EVI2A* | -8.476590054 | 3.28E-05 |
| ENSG00000184845 | *DRD1* | -8.490678006 | 0.00023105 |
| ENSG00000186827 | *TNFRSF4* | -8.494269189 | 6.10E-05 |
| ENSG00000158869 | *FCER1G* | -8.496240254 | 1.33E-10 |
| ENSG00000181631 | *P2RY13* | -8.499385254 | 0.000407699 |
| ENSG00000110324 | *IL10RA* | -8.501756752 | 5.56E-12 |
| ENSG00000144619 | *CNTN4* | -8.504220197 | 0.000207509 |
| ENSG00000019169 | *MARCO* | -8.513049989 | 4.61E-07 |
| ENSG00000170476 | *MZB1* | -8.518240329 | 0.000847731 |
| ENSG00000169783 | *LINGO1* | -8.523470357 | 0.000595261 |
| ENSG00000197291 | *RAMP2-AS1* | -8.524196329 | 5.36E-05 |
| ENSG00000010282 | *HHATL* | -8.526979391 | 0.003317175 |
| ENSG00000188176 | *SMTNL2* | -8.537036368 | 0.026031245 |
| ENSG00000234638 | *AC053503.6* | -8.537441313 | 1.44E-05 |
| ENSG00000110680 | *CALCA* | -8.53897443 | 0.001820753 |
| ENSG00000167261 | *DPEP2* | -8.541389379 | 1.97E-05 |
| ENSG00000141293 | *SKAP1* | -8.544271927 | 5.71E-05 |
| ENSG00000113361 | *CDH6* | -8.548630019 | 0.000338624 |
| ENSG00000171246 | *NPTX1* | -8.551098668 | 9.78E-05 |
| ENSG00000146013 | *GFRA3* | -8.55894445 | 0.00026887 |
| ENSG00000174837 | *ADGRE1* | -8.559721171 | 0.001241195 |
| ENSG00000259370 | *RP11-1069G10.1* | -8.559764772 | 0.000997221 |
| ENSG00000162692 | *VCAM1* | -8.568616911 | 1.40E-10 |
| ENSG00000000938 | *FGR* | -8.579401608 | 5.59E-13 |
| ENSG00000067715 | *SYT1* | -8.588510415 | 0.020152709 |
| ENSG00000164867 | *NOS3* | -8.590628234 | 1.16E-05 |
| ENSG00000006071 | *ABCC8* | -8.602759408 | 9.72E-05 |
| ENSG00000162630 | *B3GALT2* | -8.602934902 | 2.99E-05 |
| ENSG00000211669 | *IGLV3-10* | -8.613223608 | 0.001031002 |
| ENSG00000228157 | *AC007952.5* | -8.617817496 | 0.00116 |
| ENSG00000197977 | *ELOVL2* | -8.61842609 | 8.42E-05 |
| ENSG00000146966 | *DENND2A* | -8.624392752 | 6.90E-10 |
| ENSG00000134532 | *SOX5* | -8.626703549 | 0.000235174 |
| ENSG00000128266 | *GNAZ* | -8.633203102 | 4.99E-08 |
| ENSG00000255794 | *RMST* | -8.649422078 | 0.000625019 |
| ENSG00000076662 | *ICAM3* | -8.650270632 | 0.001721997 |
| ENSG00000148357 | *HMCN2* | -8.656450102 | 5.13E-13 |
| ENSG00000120280 | *CXorf21* | -8.660361942 | 0.000756025 |
| ENSG00000144837 | *PLA1A* | -8.665381297 | 8.96E-10 |
| ENSG00000180353 | *HCLS1* | -8.676059221 | 7.05E-13 |
| ENSG00000138792 | *ENPEP* | -8.678752048 | 8.48E-05 |
| ENSG00000160219 | *GAB3* | -8.686971244 | 7.72E-06 |
| ENSG00000129450 | *SIGLEC9* | -8.690807321 | 7.19E-05 |
| ENSG00000169218 | *RSPO1* | -8.69301053 | 1.77E-05 |
| ENSG00000137571 | *SLCO5A1* | -8.701873415 | 0.00036433 |
| ENSG00000187848 | *P2RX2* | -8.709254671 | 1.31E-05 |
| ENSG00000157782 | *CABP1* | -8.712069145 | 2.22E-05 |
| ENSG00000181195 | *PENK* | -8.715445766 | 5.54E-05 |
| ENSG00000244116 | *IGKV2-28* | -8.718176335 | 0.001327491 |
| ENSG00000102032 | *RENBP* | -8.718573057 | 5.33E-06 |
| ENSG00000171873 | *ADRA1D* | -8.719989954 | 6.36E-06 |
| ENSG00000206305 | *HLA-DQA1* | -8.72460919 | 0.000285697 |
| ENSG00000137959 | *IFI44L* | -8.727102994 | 1.79E-06 |
| ENSG00000141338 | *ABCA8* | -8.730313733 | 1.53E-10 |
| ENSG00000082482 | *KCNK2* | -8.736217379 | 0.000398225 |
| ENSG00000173406 | *DAB1* | -8.737910706 | 3.42E-05 |
| ENSG00000124772 | *CPNE5* | -8.740289138 | 1.23E-07 |
| ENSG00000180209 | *MYLPF* | -8.744573445 | 0.012174671 |
| ENSG00000142661 | *MYOM3* | -8.745855548 | 9.06E-05 |
| ENSG00000184160 | *ADRA2C* | -8.746136738 | 3.12E-06 |
| ENSG00000123560 | *PLP1* | -8.751646828 | 4.94E-05 |
| ENSG00000181778 | *TMEM252* | -8.755348367 | 0.000120001 |
| ENSG00000101463 | *SYNDIG1* | -8.756296619 | 1.48E-05 |
| ENSG00000108018 | *SORCS1* | -8.759302797 | 5.74E-05 |
| ENSG00000259207 | *ITGB3* | -8.765290435 | 6.14E-05 |
| ENSG00000230463 | *HLA-DRB3* | -8.765743867 | 5.90E-05 |
| ENSG00000111728 | *ST8SIA1* | -8.777276877 | 1.79E-06 |
| ENSG00000104415 | *WISP1* | -8.789124578 | 1.44E-05 |
| ENSG00000152969 | *JAKMIP1* | -8.791694255 | 0.000161891 |
| ENSG00000214313 | *AZGP1P1* | -8.792292581 | 0.000142677 |
| ENSG00000166292 | *TMEM100* | -8.792538318 | 2.21E-05 |
| ENSG00000152689 | *RASGRP3* | -8.796462813 | 5.27E-06 |
| ENSG00000151067 | *CACNA1C* | -8.798404528 | 3.90E-15 |
| ENSG00000279508 | *AL589743.1* | -8.8017784 | 0.000229257 |
| ENSG00000124126 | *PREX1* | -8.803300752 | 3.46E-12 |
| ENSG00000197380 | *DACT3* | -8.807024337 | 2.55E-16 |
| ENSG00000280623 | *PCAT14* | -8.810111212 | 1.56E-05 |
| ENSG00000186074 | *CD300LF* | -8.822389043 | 5.77E-05 |
| ENSG00000081052 | *COL4A4* | -8.825267951 | 6.63E-09 |
| ENSG00000197245 | *FAM110D* | -8.827693267 | 2.01E-05 |
| ENSG00000155011 | *DKK2* | -8.830346826 | 9.57E-05 |
| ENSG00000167851 | *CD300A* | -8.832484226 | 3.84E-06 |
| ENSG00000206240 | *HLA-DRB1* | -8.834875648 | 4.28E-06 |
| ENSG00000105967 | *TFEC* | -8.839672525 | 3.09E-05 |
| ENSG00000174640 | *SLCO2A1* | -8.840404176 | 2.67E-13 |
| ENSG00000123360 | *PDE1B* | -8.84318338 | 3.77E-05 |
| ENSG00000197705 | *KLHL14* | -8.843990087 | 8.43E-05 |
| ENSG00000040731 | *CDH10* | -8.846764883 | 0.000345318 |
| ENSG00000211890 | *IGHA2* | -8.858786652 | 2.38E-05 |
| ENSG00000136167 | *LCP1* | -8.862304919 | 0.000499256 |
| ENSG00000050628 | *PTGER3* | -8.863673572 | 1.70E-05 |
| ENSG00000140030 | *GPR65* | -8.867444845 | 4.21E-05 |
| ENSG00000144218 | *AFF3* | -8.868862232 | 1.40E-09 |
| ENSG00000081189 | *MEF2C* | -8.872119074 | 2.58E-11 |
| ENSG00000261269 | *RP11-389C8.2* | -8.87303359 | 4.25E-05 |
| ENSG00000125810 | *CD93* | -8.874498399 | 8.27E-14 |
| ENSG00000078098 | *FAP* | -8.877177143 | 0.000674453 |
| ENSG00000104967 | *NOVA2* | -8.878091947 | 5.12E-05 |
| ENSG00000095917 | *TPSD1* | -8.878679876 | 2.78E-05 |
| ENSG00000198502 | *HLA-DRB5* | -8.880683655 | 3.73E-05 |
| ENSG00000016082 | *ISL1* | -8.884487294 | 0.003433988 |
| ENSG00000173762 | *CD7* | -8.888036411 | 7.53E-05 |
| ENSG00000153707 | *PTPRD* | -8.889724532 | 2.03E-05 |
| ENSG00000204291 | *COL15A1* | -8.894374757 | 3.16E-19 |
| ENSG00000116031 | *CD207* | -8.89446406 | 8.14E-05 |
| ENSG00000035720 | *STAP1* | -8.896950717 | 0.00067461 |
| ENSG00000230630 | *DNM3OS* | -8.897320205 | 5.61E-05 |
| ENSG00000177464 | *GPR4* | -8.898378775 | 7.92E-05 |
| ENSG00000162267 | *ITIH3* | -8.90232245 | 0.000332056 |
| ENSG00000240505 | *TNFRSF13B* | -8.907803201 | 0.001804299 |
| ENSG00000183807 | *FAM162B* | -8.918203883 | 3.18E-05 |
| ENSG00000166148 | *AVPR1A* | -8.918333816 | 1.23E-05 |
| ENSG00000090382 | *LYZ* | -8.923145418 | 1.41E-10 |
| ENSG00000183813 | *CCR4* | -8.923451369 | 0.000200863 |
| ENSG00000166523 | *CLEC4E* | -8.928761544 | 1.41E-05 |
| ENSG00000075035 | *WSCD2* | -8.937361261 | 2.71E-05 |
| ENSG00000239975 | *IGKV1D-33* | -8.938990487 | 0.000635964 |
| ENSG00000010671 | *BTK* | -8.939208831 | 1.01E-07 |
| ENSG00000105383 | *CD33* | -8.942479633 | 2.02E-05 |
| ENSG00000151062 | *CACNA2D4* | -8.945025172 | 1.65E-05 |
| ENSG00000165973 | *NELL1* | -8.953758827 | 7.58E-06 |
| ENSG00000259663 | *CTD-2314G24.2* | -8.960611966 | 1.03E-05 |
| ENSG00000280323 | *AC053503.12* | -8.974934586 | 3.52E-06 |
| ENSG00000177675 | *CD163L1* | -8.985264274 | 3.14E-05 |
| ENSG00000100985 | *MMP9* | -8.9861956 | 0.001435559 |
| ENSG00000168539 | *CHRM1* | -8.989661217 | 9.82E-05 |
| ENSG00000100448 | *CTSG* | -8.999134261 | 5.87E-05 |
| ENSG00000274993 | *RP11-395B7.2* | -9.000553698 | 0.000634142 |
| ENSG00000196932 | *TMEM26* | -9.006848689 | 6.65E-06 |
| ENSG00000167749 | *KLK4* | -9.006877149 | 3.20E-13 |
| ENSG00000160185 | *UBASH3A* | -9.011978406 | 0.000135605 |
| ENSG00000072952 | *MRVI1* | -9.0184411 | 6.43E-18 |
| ENSG00000140538 | *NTRK3* | -9.022700259 | 1.87E-06 |
| ENSG00000265206 | *RP5-1171I10.5* | -9.023668839 | 0.00038405 |
| ENSG00000078328 | *RBFOX1* | -9.033466652 | 0.000425428 |
| ENSG00000245164 | *LINC00861* | -9.034393099 | 0.000363736 |
| ENSG00000188536 | *HBA2* | -9.036532792 | 5.99E-05 |
| ENSG00000281103 | *TRG-AS1* | -9.044702219 | 1.76E-05 |
| ENSG00000172403 | *SYNPO2* | -9.045303576 | 9.13E-25 |
| ENSG00000018625 | *ATP1A2* | -9.054247782 | 6.17E-18 |
| ENSG00000078589 | *P2RY10* | -9.063650333 | 0.000512238 |
| ENSG00000163239 | *TDRD10* | -9.071194464 | 1.03E-05 |
| ENSG00000162998 | *FRZB* | -9.077038599 | 3.01E-09 |
| ENSG00000254510 | *RP11-867G23.10* | -9.082340235 | 1.09E-05 |
| ENSG00000174059 | *CD34* | -9.085001165 | 2.37E-17 |
| ENSG00000105369 | *CD79A* | -9.096006983 | 0.002639167 |
| ENSG00000131471 | *AOC3* | -9.102666024 | 6.54E-27 |
| ENSG00000038945 | *MSR1* | -9.109813246 | 1.44E-06 |
| ENSG00000227191 | *TRGC2* | -9.116209206 | 2.43E-05 |
| ENSG00000105851 | *PIK3CG* | -9.120825741 | 0.000479428 |
| ENSG00000277494 | *GPIHBP1* | -9.122800523 | 7.36E-05 |
| ENSG00000267405 | *CTC-296K1.4* | -9.127408263 | 9.37E-06 |
| ENSG00000163751 | *CPA3* | -9.135548597 | 8.90E-09 |
| ENSG00000090659 | *CD209* | -9.142586327 | 1.35E-05 |
| ENSG00000166741 | *NNMT* | -9.155645031 | 0.000315884 |
| ENSG00000164879 | *CA3* | -9.156746025 | 0.003431554 |
| ENSG00000203883 | *SOX18* | -9.157116773 | 4.28E-12 |
| ENSG00000166448 | *TMEM130* | -9.158485544 | 9.31E-05 |
| ENSG00000160654 | *CD3G* | -9.160878775 | 0.000110116 |
| ENSG00000110665 | *C11orf21* | -9.167052363 | 0.000209726 |
| ENSG00000141750 | *STAC2* | -9.170306567 | 1.57E-05 |
| ENSG00000165379 | *LRFN5* | -9.171848197 | 4.70E-06 |
| ENSG00000137841 | *PLCB2* | -9.17579831 | 2.77E-10 |
| ENSG00000167332 | *OR51E2* | -9.176504193 | 7.43E-06 |
| ENSG00000160862 | *AZGP1* | -9.183098638 | 1.43E-13 |
| ENSG00000211685 | *IGLC7* | -9.189546759 | 0.000134991 |
| ENSG00000205221 | *VIT* | -9.194676149 | 5.97E-05 |
| ENSG00000244682 | *FCGR2C* | -9.195162742 | 8.25E-06 |
| ENSG00000048462 | *TNFRSF17* | -9.195471755 | 0.00121035 |
| ENSG00000125384 | *PTGER2* | -9.206744017 | 7.17E-07 |
| ENSG00000154188 | *ANGPT1* | -9.214647927 | 2.93E-06 |
| ENSG00000154783 | *FGD5* | -9.216282091 | 8.74E-07 |
| ENSG00000235621 | *LINC00494* | -9.216415452 | 0.000614359 |
| ENSG00000171659 | *GPR34* | -9.216612812 | 1.52E-05 |
| ENSG00000164440 | *TXLNB* | -9.226146491 | 4.10E-05 |
| ENSG00000002726 | *AOC1* | -9.230698614 | 1.46E-07 |
| ENSG00000134460 | *IL2RA* | -9.232992009 | 6.95E-06 |
| ENSG00000014257 | *ACPP* | -9.238534429 | 7.41E-13 |
| ENSG00000136286 | *MYO1G* | -9.238729141 | 1.53E-07 |
| ENSG00000180139 | *ACTA2-AS1* | -9.24227001 | 1.67E-12 |
| ENSG00000185052 | *SLC24A3* | -9.242998557 | 2.91E-12 |
| ENSG00000224041 | *IGKV3D-15* | -9.245034066 | 0.000411394 |
| ENSG00000166823 | *MESP1* | -9.245707323 | 3.27E-06 |
| ENSG00000163564 | *PYHIN1* | -9.250354439 | 7.78E-05 |
| ENSG00000154864 | *PIEZO2* | -9.251244643 | 5.01E-07 |
| ENSG00000187513 | *GJA4* | -9.254071161 | 3.67E-06 |
| ENSG00000135636 | *DYSF* | -9.259189322 | 2.69E-09 |
| ENSG00000154451 | *GBP5* | -9.269469383 | 1.92E-05 |
| ENSG00000167654 | *ATCAY* | -9.270396958 | 8.07E-07 |
| ENSG00000182162 | *P2RY8* | -9.275055882 | 4.17E-05 |
| ENSG00000276849 | *TRBC2* | -9.279235181 | 8.90E-05 |
| ENSG00000198846 | *TOX* | -9.280000573 | 1.80E-06 |
| ENSG00000020181 | *ADGRA2* | -9.2807489 | 1.25E-15 |
| ENSG00000087116 | *ADAMTS2* | -9.295610493 | 1.95E-07 |
| ENSG00000271605 | *MILR1* | -9.297739972 | 9.18E-06 |
| ENSG00000127951 | *FGL2* | -9.30437706 | 2.84E-07 |
| ENSG00000101335 | *MYL9* | -9.306071897 | 2.47E-23 |
| ENSG00000165186 | *PTCHD1* | -9.307025631 | 1.06E-06 |
| ENSG00000108405 | *P2RX1* | -9.311023742 | 1.03E-06 |
| ENSG00000174332 | *GLIS1* | -9.320316373 | 0.009872583 |
| ENSG00000162367 | *TAL1* | -9.320973019 | 3.62E-06 |
| ENSG00000182487 | *NCF1B* | -9.323185208 | 0.000276316 |
| ENSG00000132185 | *FCRLA* | -9.323332689 | 0.000366697 |
| ENSG00000235505 | *RP11-693N9.2* | -9.32485406 | 5.52E-06 |
| ENSG00000127083 | *OMD* | -9.326500863 | 1.91E-05 |
| ENSG00000149591 | *TAGLN* | -9.331784077 | 8.14E-21 |
| ENSG00000186469 | *GNG2* | -9.332154811 | 1.43E-06 |
| ENSG00000272789 | *RP11-286H15.1* | -9.334436053 | 7.72E-06 |
| ENSG00000124203 | *ZNF831* | -9.338151074 | 4.40E-05 |
| ENSG00000133392 | *MYH11* | -9.341481081 | 1.69E-06 |
| ENSG00000164035 | *EMCN* | -9.350179186 | 1.79E-06 |
| ENSG00000181234 | *TMEM132C* | -9.354666179 | 5.32E-06 |
| ENSG00000179639 | *FCER1A* | -9.355758405 | 3.64E-06 |
| ENSG00000171860 | *C3AR1* | -9.357428948 | 8.80E-07 |
| ENSG00000261685 | *RP11-401P9.4* | -9.368656187 | 2.95E-07 |
| ENSG00000146285 | *SCML4* | -9.374719772 | 1.49E-05 |
| ENSG00000089012 | *SIRPG* | -9.380264007 | 6.60E-05 |
| ENSG00000070193 | *FGF10* | -9.381578602 | 9.95E-05 |
| ENSG00000161281 | *COX7A1* | -9.388300962 | 5.32E-11 |
| ENSG00000174099 | *MSRB3* | -9.389265296 | 4.44E-12 |
| ENSG00000172399 | *MYOZ2* | -9.402179278 | 0.02131182 |
| ENSG00000263961 | *C1orf186* | -9.40683443 | 6.08E-06 |
| ENSG00000105989 | *WNT2* | -9.410137327 | 7.24E-06 |
| ENSG00000163563 | *MNDA* | -9.412460986 | 3.48E-06 |
| ENSG00000152049 | *KCNE4* | -9.416932279 | 7.48E-07 |
| ENSG00000121871 | *SLITRK3* | -9.418941989 | 0.000231629 |
| ENSG00000171051 | *FPR1* | -9.425174255 | 1.15E-05 |
| ENSG00000228278 | *ORM2* | -9.427260879 | 0.000103111 |
| ENSG00000089472 | *HEPH* | -9.4304568 | 6.06E-15 |
| ENSG00000169126 | *ARMC4* | -9.433009194 | 2.11E-06 |
| ENSG00000205364 | *MT1M* | -9.433076422 | 3.46E-07 |
| ENSG00000109906 | *ZBTB16* | -9.434133543 | 3.35E-07 |
| ENSG00000005844 | *ITGAL* | -9.434813601 | 1.13E-09 |
| ENSG00000211640 | *IGLV6-57* | -9.435118244 | 0.039039157 |
| ENSG00000257542 | *OR7E47P* | -9.438523196 | 1.88E-06 |
| ENSG00000116748 | *AMPD1* | -9.440587537 | 0.000206696 |
| ENSG00000164736 | *SOX17* | -9.443259435 | 5.02E-06 |
| ENSG00000135905 | *DOCK10* | -9.443351439 | 4.14E-05 |
| ENSG00000175445 | *LPL* | -9.464444262 | 1.22E-06 |
| ENSG00000136732 | *GYPC* | -9.465732292 | 2.52E-12 |
| ENSG00000196616 | *ADH1B* | -9.470209052 | 5.69E-05 |
| ENSG00000101470 | *TNNC2* | -9.48011947 | 0.001858702 |
| ENSG00000105664 | *COMP* | -9.488501346 | 0.000604407 |
| ENSG00000228080 | *HLA-DRB1* | -9.490150157 | 9.49E-06 |
| ENSG00000169994 | *MYO7B* | -9.500319418 | 1.32E-05 |
| ENSG00000079102 | *RUNX1T1* | -9.502329698 | 6.60E-07 |
| ENSG00000130307 | *USHBP1* | -9.502370744 | 5.24E-06 |
| ENSG00000115361 | *ACADL* | -9.504134481 | 4.49E-07 |
| ENSG00000147443 | *DOK2* | -9.507090923 | 3.22E-06 |
| ENSG00000131634 | *TMEM204* | -9.507570602 | 1.44E-10 |
| ENSG00000100884 | *CPNE6* | -9.508353185 | 2.22E-06 |
| ENSG00000211751 | *TRBV25-1* | -9.512949283 | 0.000119764 |
| ENSG00000115085 | *ZAP70* | -9.515851689 | 1.97E-08 |
| ENSG00000090104 | *RGS1* | -9.538458897 | 3.28E-19 |
| ENSG00000196208 | *GREB1* | -9.540235793 | 1.44E-12 |
| ENSG00000113721 | *PDGFRB* | -9.54276339 | 2.71E-20 |
| ENSG00000106018 | *VIPR2* | -9.55242932 | 6.22E-06 |
| ENSG00000161405 | *IKZF3* | -9.553741579 | 1.42E-08 |
| ENSG00000123338 | *NCKAP1L* | -9.565886335 | 1.27E-12 |
| ENSG00000167208 | *SNX20* | -9.573892172 | 4.90E-05 |
| ENSG00000166482 | *MFAP4* | -9.580393314 | 5.31E-27 |
| ENSG00000167281 | *RBFOX3* | -9.604012237 | 1.40E-13 |
| ENSG00000160801 | *PTH1R* | -9.605507367 | 3.71E-07 |
| ENSG00000255399 | *TBX5-AS1* | -9.61305832 | 3.61E-05 |
| ENSG00000100450 | *GZMH* | -9.618166254 | 1.20E-05 |
| ENSG00000178860 | *MSC* | -9.626228067 | 2.36E-05 |
| ENSG00000079263 | *SP140* | -9.635118023 | 1.38E-05 |
| ENSG00000007264 | *MATK* | -9.635339746 | 7.52E-06 |
| ENSG00000131196 | *NFATC1* | -9.640768772 | 4.97E-06 |
| ENSG00000120279 | *MYCT1* | -9.642298637 | 2.99E-06 |
| ENSG00000280143 | *AP000892.6* | -9.64232236 | 1.73E-14 |
| ENSG00000107719 | *PALD1* | -9.661082897 | 1.78E-07 |
| ENSG00000279496 | *CTC-251I16.1* | -9.662331004 | 2.62E-06 |
| ENSG00000115165 | *CYTIP* | -9.664934476 | 1.22E-06 |
| ENSG00000104903 | *LYL1* | -9.665525616 | 1.67E-06 |
| ENSG00000203710 | *CR1* | -9.669660657 | 6.99E-05 |
| ENSG00000143632 | *ACTA1* | -9.677168786 | 0.027312571 |
| ENSG00000005102 | *MEOX1* | -9.681306496 | 1.81E-05 |
| ENSG00000089225 | *TBX5* | -9.690897736 | 7.06E-06 |
| ENSG00000225986 | *UBXN10-AS1* | -9.697625621 | 4.85E-06 |
| ENSG00000164692 | *COL1A2* | -9.699596215 | 8.95E-24 |
| ENSG00000079931 | *MOXD1* | -9.70245006 | 5.14E-10 |
| ENSG00000183918 | *SH2D1A* | -9.707269947 | 5.31E-05 |
| ENSG00000172005 | *MAL* | -9.712342334 | 2.48E-06 |
| ENSG00000267505 | *CTC-296K1.3* | -9.71656724 | 3.59E-07 |
| ENSG00000154736 | *ADAMTS5* | -9.725167058 | 1.95E-06 |
| ENSG00000100453 | *GZMB* | -9.732178358 | 0.030654086 |
| ENSG00000187068 | *C3orf70* | -9.747226702 | 3.71E-07 |
| ENSG00000139329 | *LUM* | -9.749676805 | 4.36E-15 |
| ENSG00000214402 | *LCNL1* | -9.760260858 | 7.50E-06 |
| ENSG00000135447 | *PPP1R1A* | -9.76744971 | 5.25E-08 |
| ENSG00000120907 | *ADRA1A* | -9.769532466 | 3.09E-07 |
| ENSG00000103522 | *IL21R* | -9.769878708 | 5.37E-05 |
| ENSG00000198626 | *RYR2* | -9.776471216 | 1.21E-06 |
| ENSG00000163599 | *CTLA4* | -9.7808781 | 7.00E-06 |
| ENSG00000177455 | *CD19* | -9.781556627 | 0.006740716 |
| ENSG00000163823 | *CCR1* | -9.781827266 | 2.65E-06 |
| ENSG00000134061 | *CD180* | -9.788224586 | 6.39E-06 |
| ENSG00000178562 | *CD28* | -9.793585537 | 3.53E-05 |
| ENSG00000185862 | *EVI2B* | -9.802042233 | 6.43E-07 |
| ENSG00000135426 | *TESPA1* | -9.809516092 | 5.11E-06 |
| ENSG00000050555 | *LAMC3* | -9.813420999 | 5.27E-06 |
| ENSG00000276480 | *MYH11* | -9.814299361 | 9.21E-07 |
| ENSG00000162511 | *LAPTM5* | -9.815096816 | 1.83E-24 |
| ENSG00000147113 | *CXorf36* | -9.821992775 | 2.75E-08 |
| ENSG00000185905 | *C16orf54* | -9.831161604 | 2.46E-05 |
| ENSG00000160539 | *PPAPDC3* | -9.833021933 | 2.39E-07 |
| ENSG00000103710 | *RASL12* | -9.836174139 | 1.41E-19 |
| ENSG00000139567 | *ACVRL1* | -9.836989142 | 3.25E-08 |
| ENSG00000276231 | *PIK3R6* | -9.841127471 | 5.79E-06 |
| ENSG00000111452 | *ADGRD1* | -9.858983013 | 1.01E-07 |
| ENSG00000196126 | *HLA-DRB1* | -9.865221131 | 2.11E-07 |
| ENSG00000158473 | *CD1D* | -9.866062243 | 1.33E-06 |
| ENSG00000162878 | *PKDCC* | -9.873306115 | 6.09E-07 |
| ENSG00000172116 | *CD8B* | -9.887834408 | 4.96E-06 |
| ENSG00000064205 | *WISP2* | -9.890330992 | 2.12E-05 |
| ENSG00000211897 | *IGHG3* | -9.899171949 | 0.00157881 |
| ENSG00000105246 | *EBI3* | -9.903425245 | 9.61E-07 |
| ENSG00000180644 | *PRF1* | -9.910784347 | 1.02E-05 |
| ENSG00000230708 | *HLA-DPB1* | -9.929512291 | 0.000793302 |
| ENSG00000154721 | *JAM2* | -9.952650423 | 1.49E-07 |
| ENSG00000161896 | *IP6K3* | -9.953448501 | 2.43E-07 |
| ENSG00000137078 | *SIT1* | -9.956550512 | 2.10E-05 |
| ENSG00000130528 | *HRC* | -9.956771656 | 0.017419216 |
| ENSG00000142173 | *COL6A2* | -9.959580566 | 1.95E-27 |
| ENSG00000163568 | *AIM2* | -9.974907519 | 3.74E-05 |
| ENSG00000118729 | *CASQ2* | -9.981581425 | 5.09E-06 |
| ENSG00000122224 | *LY9* | -10.00142827 | 1.14E-05 |
| ENSG00000244734 | *HBB* | -10.00159407 | 0.01448578 |
| ENSG00000110076 | *NRXN2* | -10.00394344 | 4.21E-07 |
| ENSG00000099998 | *GGT5* | -10.00434213 | 2.13E-14 |
| ENSG00000124205 | *EDN3* | -10.01746657 | 0.03612816 |
| ENSG00000133574 | *GIMAP4* | -10.04528685 | 5.67E-08 |
| ENSG00000163154 | *TNFAIP8L2* | -10.04819234 | 1.33E-06 |
| ENSG00000269404 | *SPIB* | -10.05360258 | 0.015689683 |
| ENSG00000163359 | *COL6A3* | -10.05469762 | 2.48E-35 |
| ENSG00000160791 | *CCR5* | -10.05874134 | 4.77E-06 |
| ENSG00000112562 | *SMOC2* | -10.0607658 | 3.90E-15 |
| ENSG00000160883 | *HK3* | -10.06209222 | 4.88E-06 |
| ENSG00000155307 | *SAMSN1* | -10.0780117 | 1.44E-06 |
| ENSG00000121807 | *CCR2* | -10.07866253 | 4.40E-06 |
| ENSG00000211895 | *IGHA1* | -10.07869749 | 0.001676723 |
| ENSG00000028137 | *TNFRSF1B* | -10.0888886 | 8.24E-11 |
| ENSG00000173991 | *TCAP* | -10.09226031 | 6.35E-05 |
| ENSG00000158481 | *CD1C* | -10.10400309 | 3.50E-06 |
| ENSG00000164330 | *EBF1* | -10.10774252 | 4.17E-06 |
| ENSG00000260314 | *MRC1* | -10.11731084 | 1.96E-07 |
| ENSG00000124256 | *ZBP1* | -10.12457143 | 0.020133725 |
| ENSG00000143603 | *KCNN3* | -10.1262136 | 2.99E-07 |
| ENSG00000118473 | *SGIP1* | -10.13392317 | 1.81E-07 |
| ENSG00000130224 | *LRCH2* | -10.1376638 | 2.46E-07 |
| ENSG00000197859 | *ADAMTSL2* | -10.13931056 | 3.33E-06 |
| ENSG00000167476 | *JSRP1* | -10.14334423 | 1.67E-05 |
| ENSG00000197471 | *SPN* | -10.14552226 | 1.33E-05 |
| ENSG00000241351 | *IGKV3-11* | -10.16423587 | 0.022234465 |
| ENSG00000100368 | *CSF2RB* | -10.16867707 | 5.83E-06 |
| ENSG00000093072 | *CECR1* | -10.17352022 | 6.15E-12 |
| ENSG00000160808 | *MYL3* | -10.17595599 | 0.023237832 |
| ENSG00000170624 | *SGCD* | -10.17705396 | 1.10E-07 |
| ENSG00000164122 | *ASB5* | -10.17782409 | 0.003060636 |
| ENSG00000175899 | *A2M* | -10.18238913 | 1.54E-05 |
| ENSG00000133878 | *DUSP26* | -10.19061863 | 5.16E-06 |
| ENSG00000130600 | *H19* | -10.19117056 | 5.54E-05 |
| ENSG00000150594 | *ADRA2A* | -10.19815296 | 2.18E-07 |
| ENSG00000128438 | *TBC1D27* | -10.20205033 | 0.043017726 |
| ENSG00000111341 | *MGP* | -10.20979637 | 1.58E-14 |
| ENSG00000175857 | *GAPT* | -10.23251895 | 1.22E-05 |
| ENSG00000143318 | *CASQ1* | -10.23571818 | 8.74E-06 |
| ENSG00000229295 | *HLA-DPB1* | -10.24381049 | 5.16E-08 |
| ENSG00000160856 | *FCRL3* | -10.25049164 | 0.031667962 |
| ENSG00000174348 | *PODN* | -10.25074246 | 2.88E-15 |
| ENSG00000168675 | *LDLRAD4* | -10.25770934 | 5.97E-09 |
| ENSG00000138755 | *CXCL9* | -10.25898608 | 3.47E-06 |
| ENSG00000106066 | *CPVL* | -10.27496588 | 9.32E-09 |
| ENSG00000225217 | *HSPA7* | -10.27980846 | 2.06E-07 |
| ENSG00000110079 | *MS4A4A* | -10.28429647 | 5.71E-08 |
| ENSG00000149294 | *NCAM1* | -10.29671679 | 1.28E-08 |
| ENSG00000277016 | *IGHG4* | -10.30438405 | 0.027263704 |
| ENSG00000187474 | *FPR3* | -10.31188064 | 1.78E-07 |
| ENSG00000157445 | *CACNA2D3* | -10.33566113 | 4.95E-08 |
| ENSG00000002933 | *TMEM176A* | -10.34290282 | 2.51E-08 |
| ENSG00000116824 | *CD2* | -10.34557125 | 1.06E-07 |
| ENSG00000172935 | *MRGPRF* | -10.35827297 | 9.65E-14 |
| ENSG00000034971 | *MYOC* | -10.36618256 | 0.010813633 |
| ENSG00000156127 | *BATF* | -10.37638056 | 5.78E-07 |
| ENSG00000167244 | *IGF2* | -10.38900585 | 3.16E-07 |
| ENSG00000111249 | *CUX2* | -10.40830469 | 6.98E-06 |
| ENSG00000158445 | *KCNB1* | -10.41249422 | 2.51E-08 |
| ENSG00000227993 | *HLA-DRA* | -10.41615379 | 6.66E-09 |
| ENSG00000072694 | *FCGR2B* | -10.41823221 | 3.35E-07 |
| ENSG00000187955 | *COL14A1* | -10.4445128 | 9.83E-14 |
| ENSG00000117090 | *SLAMF1* | -10.4548967 | 2.21E-06 |
| ENSG00000156738 | *MS4A1* | -10.45956298 | 0.008655456 |
| ENSG00000213203 | *GIMAP1* | -10.46202836 | 7.10E-08 |
| ENSG00000145423 | *SFRP2* | -10.46373704 | 1.18E-14 |
| ENSG00000274497 | *IGHG2* | -10.46394166 | 0.019165594 |
| ENSG00000144810 | *COL8A1* | -10.46513934 | 4.92E-07 |
| ENSG00000077984 | *CST7* | -10.47747488 | 4.39E-07 |
| ENSG00000136546 | *SCN7A* | -10.48050332 | 5.43E-07 |
| ENSG00000166105 | *GLB1L3* | -10.4931504 | 2.84E-08 |
| ENSG00000113263 | *ITK* | -10.50544132 | 2.20E-07 |
| ENSG00000147655 | *RSPO2* | -10.53449529 | 0.00509303 |
| ENSG00000124440 | *HIF3A* | -10.53649978 | 4.04E-09 |
| ENSG00000112782 | *CLIC5* | -10.56723539 | 2.16E-07 |
| ENSG00000138650 | *PCDH10* | -10.56806133 | 5.92E-09 |
| ENSG00000133107 | *TRPC4* | -10.57661656 | 1.06E-07 |
| ENSG00000141506 | *PIK3R5* | -10.57996917 | 9.04E-08 |
| ENSG00000181847 | *TIGIT* | -10.588572 | 0.009509794 |
| ENSG00000134516 | *DOCK2* | -10.62508289 | 7.24E-08 |
| ENSG00000122585 | *NPY* | -10.63543628 | 8.78E-07 |
| ENSG00000241158 | *ADAMTS9-AS1* | -10.64239385 | 3.32E-08 |
| ENSG00000118526 | *TCF21* | -10.64460427 | 1.70E-08 |
| ENSG00000168542 | *COL3A1* | -10.64499326 | 7.40E-35 |
| ENSG00000172236 | *TPSAB1* | -10.64591348 | 1.09E-08 |
| ENSG00000090539 | *CHRD* | -10.6526632 | 4.14E-09 |
| ENSG00000162745 | *OLFML2B* | -10.65708316 | 1.13E-08 |
| ENSG00000250423 | *KIAA1210* | -10.66597002 | 0.006643286 |
| ENSG00000144681 | *STAC* | -10.66992451 | 1.03E-07 |
| ENSG00000137491 | *SLCO2B1* | -10.67275463 | 8.96E-10 |
| ENSG00000118785 | *SPP1* | -10.68104208 | 2.30E-11 |
| ENSG00000101445 | *PPP1R16B* | -10.68581326 | 0.002625057 |
| ENSG00000127472 | *PLA2G5* | -10.69262683 | 2.68E-08 |
| ENSG00000132514 | *CLEC10A* | -10.70290941 | 2.23E-08 |
| ENSG00000048540 | *LMO3* | -10.7043223 | 3.18E-07 |
| ENSG00000130595 | *TNNT3* | -10.70842865 | 0.000333649 |
| ENSG00000213088 | *ACKR1* | -10.71362198 | 6.02E-09 |
| ENSG00000116774 | *OLFML3* | -10.71741151 | 3.52E-12 |
| ENSG00000106624 | *AEBP1* | -10.72439055 | 4.75E-34 |
| ENSG00000017427 | *IGF1* | -10.73572658 | 7.91E-08 |
| ENSG00000188783 | *PRELP* | -10.73903143 | 3.10E-25 |
| ENSG00000215218 | *UBE2QL1* | -10.74307306 | 4.56E-09 |
| ENSG00000136960 | *ENPP2* | -10.74788856 | 3.72E-09 |
| ENSG00000143125 | *PROK1* | -10.75681172 | 2.46E-07 |
| ENSG00000180875 | *GREM2* | -10.76348339 | 1.97E-08 |
| ENSG00000145649 | *GZMA* | -10.76734471 | 4.46E-07 |
| ENSG00000108798 | *ABI3* | -10.79850943 | 4.75E-09 |
| ENSG00000112799 | *LY86* | -10.79917511 | 9.35E-08 |
| ENSG00000075884 | *ARHGAP15* | -10.81833315 | 1.89E-08 |
| ENSG00000240583 | *AQP1* | -10.82011029 | 5.57E-23 |
| ENSG00000158270 | *COLEC12* | -10.83183304 | 2.35E-09 |
| ENSG00000173376 | *NDNF* | -10.83767495 | 2.95E-09 |
| ENSG00000010327 | *STAB1* | -10.84189365 | 2.63E-22 |
| ENSG00000110077 | *MS4A6A* | -10.85108762 | 1.26E-09 |
| ENSG00000124491 | *F13A1* | -10.85360523 | 1.24E-08 |
| ENSG00000242534 | *IGKV2D-28* | -10.85986329 | 0.008151768 |
| ENSG00000159189 | *C1QC* | -10.86156556 | 5.94E-15 |
| ENSG00000066294 | *CD84* | -10.89543111 | 2.25E-08 |
| ENSG00000107562 | *CXCL12* | -10.89567666 | 4.18E-09 |
| ENSG00000085265 | *FCN1* | -10.90285132 | 0.001546214 |
| ENSG00000162739 | *SLAMF6* | -10.90481387 | 2.70E-08 |
| ENSG00000105374 | *NKG7* | -10.91664084 | 0.001154402 |
| ENSG00000176435 | *CLEC14A* | -10.91901815 | 4.32E-09 |
| ENSG00000171303 | *KCNK3* | -10.93029724 | 2.54E-08 |
| ENSG00000133561 | *GIMAP6* | -10.93182325 | 2.42E-09 |
| ENSG00000100473 | *COCH* | -10.95261603 | 6.64E-10 |
| ENSG00000268388 | *FENDRR* | -10.95549047 | 1.54E-10 |
| ENSG00000105894 | *PTN* | -10.97633044 | 8.73E-09 |
| ENSG00000274827 | *LINC01297* | -10.98333422 | 2.93E-07 |
| ENSG00000082175 | *PGR* | -10.99304393 | 6.64E-10 |
| ENSG00000088827 | *SIGLEC1* | -10.99831039 | 9.20E-09 |
| ENSG00000106772 | *PRUNE2* | -11.00414423 | 5.93E-22 |
| ENSG00000166927 | *MS4A7* | -11.01064646 | 1.62E-09 |
| ENSG00000131477 | *RAMP2* | -11.02282634 | 8.11E-09 |
| ENSG00000205358 | *MT1H* | -11.03192172 | 0.00767475 |
| ENSG00000054938 | *CHRDL2* | -11.03217351 | 7.92E-09 |
| ENSG00000165457 | *FOLR2* | -11.03711829 | 2.17E-08 |
| ENSG00000077420 | *APBB1IP* | -11.04750858 | 2.07E-07 |
| ENSG00000211648 | *IGLV1-47* | -11.0563869 | 0.032214342 |
| ENSG00000211893 | *IGHG2* | -11.07382722 | 0.027640777 |
| ENSG00000174600 | *CMKLR1* | -11.07540862 | 9.67E-09 |
| ENSG00000173641 | *HSPB7* | -11.08705422 | 2.02E-08 |
| ENSG00000144339 | *TMEFF2* | -11.08915824 | 6.96E-10 |
| ENSG00000179144 | *GIMAP7* | -11.09025904 | 3.07E-09 |
| ENSG00000185739 | *SRL* | -11.09279308 | 0.002706537 |
| ENSG00000187688 | *TRPV2* | -11.09394611 | 1.02E-08 |
| ENSG00000167286 | *CD3D* | -11.09515008 | 5.61E-08 |
| ENSG00000129009 | *ISLR* | -11.10268965 | 2.62E-14 |
| ENSG00000019991 | *HGF* | -11.10277666 | 5.87E-09 |
| ENSG00000136250 | *AOAH* | -11.10464733 | 1.73E-08 |
| ENSG00000007312 | *CD79B* | -11.1066573 | 1.20E-07 |
| ENSG00000204936 | *CD177* | -11.11187421 | 1.73E-06 |
| ENSG00000155659 | *VSIG4* | -11.12626145 | 8.81E-10 |
| ENSG00000211896 | *IGHG1* | -11.13061113 | 0.001006792 |
| ENSG00000186642 | *PDE2A* | -11.13107493 | 2.88E-10 |
| ENSG00000119147 | *C2orf40* | -11.13674991 | 1.81E-09 |
| ENSG00000121361 | *KCNJ8* | -11.13710009 | 2.23E-09 |
| ENSG00000114854 | *TNNC1* | -11.15941279 | 7.85E-05 |
| ENSG00000179776 | *CDH5* | -11.16131783 | 3.01E-10 |
| ENSG00000173200 | *PARP15* | -11.16644079 | 1.08E-07 |
| ENSG00000231679 | *HLA-DRB3* | -11.17347464 | 1.15E-07 |
| ENSG00000155849 | *ELMO1* | -11.17376999 | 1.13E-09 |
| ENSG00000122679 | *RAMP3* | -11.17441756 | 3.25E-10 |
| ENSG00000128815 | *WDFY4* | -11.18496874 | 0.00533528 |
| ENSG00000172987 | *HPSE2* | -11.18967958 | 7.02E-08 |
| ENSG00000174403 | *C20orf166-AS1* | -11.19324905 | 4.51E-10 |
| ENSG00000155629 | *PIK3AP1* | -11.19845919 | 1.81E-08 |
| ENSG00000165168 | *CYBB* | -11.2010437 | 6.05E-09 |
| ENSG00000066056 | *TIE1* | -11.20952982 | 4.24E-10 |
| ENSG00000137077 | *CCL21* | -11.22122293 | 0.000959806 |
| ENSG00000164106 | *SCRG1* | -11.22138141 | 9.03E-08 |
| ENSG00000140678 | *ITGAX* | -11.22850571 | 2.77E-08 |
| ENSG00000119865 | *CNRIP1* | -11.23258795 | 1.94E-09 |
| ENSG00000138944 | *KIAA1644* | -11.23352159 | 4.18E-09 |
| ENSG00000198844 | *ARHGEF15* | -11.24044139 | 2.29E-10 |
| ENSG00000145703 | *IQGAP2* | -11.25367666 | 4.48E-10 |
| ENSG00000169413 | *RNASE6* | -11.26260445 | 1.65E-09 |
| ENSG00000146374 | *RSPO3* | -11.2696017 | 0.001229526 |
| ENSG00000128591 | *FLNC* | -11.30093538 | 1.18E-36 |
| ENSG00000106483 | *SFRP4* | -11.3016456 | 6.51E-09 |
| ENSG00000203747 | *FCGR3A* | -11.31336354 | 4.51E-09 |
| ENSG00000164161 | *HHIP* | -11.32277767 | 1.46E-09 |
| ENSG00000243466 | *IGKV1-5* | -11.32875781 | 0.03620438 |
| ENSG00000145936 | *KCNMB1* | -11.33330602 | 1.44E-16 |
| ENSG00000102445 | *KIAA0226L* | -11.34059014 | 1.32E-08 |
| ENSG00000241644 | *INMT* | -11.35718835 | 1.39E-09 |
| ENSG00000081237 | *PTPRC* | -11.37186236 | 1.68E-08 |
| ENSG00000004468 | *CD38* | -11.38752698 | 2.36E-09 |
| ENSG00000185274 | *WBSCR17* | -11.38781758 | 6.65E-10 |
| ENSG00000147168 | *IL2RG* | -11.39325001 | 0.000356224 |
| ENSG00000182578 | *CSF1R* | -11.39588646 | 3.87E-10 |
| ENSG00000129538 | *RNASE1* | -11.40555615 | 2.95E-09 |
| ENSG00000118308 | *LRMP* | -11.41397658 | 7.29E-08 |
| ENSG00000165966 | *PDZRN4* | -11.4383131 | 2.05E-09 |
| ENSG00000130598 | *TNNI2* | -11.44308574 | 0.001182805 |
| ENSG00000012223 | *LTF* | -11.44724461 | 0.000839138 |
| ENSG00000174175 | *SELP* | -11.44842237 | 1.17E-09 |
| ENSG00000116132 | *PRRX1* | -11.4521958 | 1.86E-10 |
| ENSG00000110934 | *BIN2* | -11.51344639 | 3.01E-09 |
| ENSG00000169554 | *ZEB2* | -11.52084315 | 3.99E-11 |
| ENSG00000011465 | *DCN* | -11.54440611 | 1.75E-17 |
| ENSG00000253755 | *IGHGP* | -11.55939763 | 0.011288452 |
| ENSG00000154096 | *THY1* | -11.57711571 | 2.85E-09 |
| ENSG00000143196 | *DPT* | -11.59243261 | 2.67E-09 |
| ENSG00000103241 | *FOXF1* | -11.60713912 | 6.13E-11 |
| ENSG00000163431 | *LMOD1* | -11.61719244 | 3.93E-32 |
| ENSG00000092054 | *MYH7* | -11.69104951 | 0.007439146 |
| ENSG00000068831 | *RASGRP2* | -11.70074609 | 6.96E-10 |
| ENSG00000117091 | *CD48* | -11.70964659 | 8.70E-09 |
| ENSG00000132965 | *ALOX5AP* | -11.71574764 | 0.000308618 |
| ENSG00000072163 | *LIMS2* | -11.71669344 | 9.98E-20 |
| ENSG00000143119 | *CD53* | -11.71682098 | 4.82E-10 |
| ENSG00000154330 | *PGM5* | -11.73269076 | 2.04E-32 |
| ENSG00000149596 | *JPH2* | -11.75325061 | 1.94E-11 |
| ENSG00000155926 | *SLA* | -11.75522116 | 4.81E-11 |
| ENSG00000130176 | *CNN1* | -11.76180095 | 9.25E-32 |
| ENSG00000143851 | *PTPN7* | -11.76661928 | 8.11E-09 |
| ENSG00000183160 | *TMEM119* | -11.7683077 | 3.64E-09 |
| ENSG00000106034 | *CPED1* | -11.77231918 | 1.33E-10 |
| ENSG00000136573 | *BLK* | -11.77424445 | 0.015732464 |
| ENSG00000134201 | *GSTM5* | -11.79203965 | 1.37E-10 |
| ENSG00000239474 | *KLHL41* | -11.79844839 | 0.035995814 |
| ENSG00000133110 | *POSTN* | -11.82629226 | 3.19E-10 |
| ENSG00000066336 | *SPI1* | -11.82679131 | 2.20E-09 |
| ENSG00000165633 | *VSTM4* | -11.86282057 | 7.00E-12 |
| ENSG00000168685 | *IL7R* | -11.90124211 | 1.18E-11 |
| ENSG00000160593 | *AMICA1* | -11.91576956 | 1.43E-10 |
| ENSG00000104313 | *EYA1* | -11.92770464 | 4.46E-10 |
| ENSG00000108823 | *SGCA* | -11.92918872 | 6.76E-11 |
| ENSG00000146122 | *DAAM2* | -11.95129928 | 9.14E-12 |
| ENSG00000138964 | *PARVG* | -11.98966629 | 3.92E-10 |
| ENSG00000182866 | *LCK* | -12.00667843 | 5.79E-09 |
| ENSG00000231286 | *HLA-DQB1* | -12.01187301 | 2.02E-09 |
| ENSG00000103175 | *WFDC1* | -12.03241688 | 3.04E-11 |
| ENSG00000143297 | *FCRL5* | -12.04973205 | 0.015220346 |
| ENSG00000141052 | *MYOCD* | -12.06274398 | 4.01E-11 |
| ENSG00000122122 | *SASH3* | -12.07992802 | 0.000233718 |
| ENSG00000177575 | *CD163* | -12.08987476 | 3.21E-12 |
| ENSG00000121966 | *CXCR4* | -12.12902845 | 2.25E-11 |
| ENSG00000106809 | *OGN* | -12.17472447 | 4.45E-11 |
| ENSG00000152583 | *SPARCL1* | -12.18455911 | 7.76E-29 |
| ENSG00000198523 | *PLN* | -12.21575749 | 0.000219482 |
| ENSG00000140968 | *IRF8* | -12.23591999 | 7.59E-05 |
| ENSG00000123329 | *ARHGAP9* | -12.27541142 | 3.83E-05 |
| ENSG00000175084 | *DES* | -12.31758651 | 1.55E-56 |
| ENSG00000276173 | *IGHA2* | -12.33565528 | 0.00661096 |
| ENSG00000105122 | *RASAL3* | -12.38404837 | 6.13E-10 |
| ENSG00000249669 | *MIR143HG* | -12.44546031 | 2.82E-12 |
| ENSG00000106565 | *TMEM176B* | -12.51829791 | 9.98E-12 |
| ENSG00000277893 | *SRD5A2* | -12.53211672 | 1.40E-10 |
| ENSG00000211663 | *IGLV3-19* | -12.53950366 | 0.031643312 |
| ENSG00000115956 | *PLEK* | -12.55072577 | 4.41E-05 |
| ENSG00000012124 | *CD22* | -12.61614776 | 1.57E-09 |
| ENSG00000130755 | *GMFG* | -12.62248766 | 1.41E-10 |
| ENSG00000168530 | *MYL1* | -12.64133894 | 0.048932685 |
| ENSG00000197629 | *MPEG1* | -12.64581111 | 1.79E-10 |
| ENSG00000224958 | *PGM5-AS1* | -12.67620967 | 2.62E-05 |
| ENSG00000167751 | *KLK2* | -12.7398387 | 3.53E-20 |
| ENSG00000163017 | *ACTG2* | -12.74071752 | 4.46E-39 |
| ENSG00000260802 | *LINC00890* | -12.7612093 | 1.45E-10 |
| ENSG00000104879 | *CKM* | -12.78013128 | 6.69E-05 |
| ENSG00000011600 | *TYROBP* | -12.80936744 | 3.26E-12 |
| ENSG00000124212 | *PTGIS* | -12.81171896 | 4.95E-13 |
| ENSG00000004776 | *HSPB6* | -12.81530921 | 4.59E-13 |
| ENSG00000077522 | *ACTN2* | -12.86274061 | 0.006633853 |
| ENSG00000112936 | *C7* | -12.91396016 | 3.65E-10 |
| ENSG00000172724 | *CCL19* | -12.9486439 | 0.000126217 |
| ENSG00000282094 | *IGHGP* | -12.9626439 | 0.001467227 |
| ENSG00000211677 | *IGLC2* | -13.0280092 | 0.000103465 |
| ENSG00000183036 | *PCP4* | -13.07851002 | 1.79E-11 |
| ENSG00000130592 | *LSP1* | -13.09701733 | 3.67E-12 |
| ENSG00000277633 | *IGHG1* | -13.12683574 | 0.001448362 |
| ENSG00000228987 | *HLA-DRA* | -13.16951379 | 0.015944891 |
| ENSG00000077943 | *ITGA8* | -13.23652147 | 8.18E-13 |
| ENSG00000211662 | *IGLV3-21* | -13.30262857 | 0.034958087 |
| ENSG00000188257 | *PLA2G2A* | -13.31155105 | 5.39E-12 |
| ENSG00000196091 | *MYBPC1* | -13.35983194 | 3.99E-12 |
| ENSG00000196557 | *CACNA1H* | -13.39675975 | 4.11E-06 |
| ENSG00000101951 | *PAGE4* | -13.46250358 | 0.000507572 |
| ENSG00000159251 | *ACTC1* | -13.67652951 | 0.00127998 |
| ENSG00000142515 | *KLK3* | -13.7456992 | 1.06E-21 |
| ENSG00000173369 | *C1QB* | -13.7784162 | 7.50E-15 |
| ENSG00000168309 | *FAM107A* | -13.81727772 | 2.95E-14 |
| ENSG00000156234 | *CXCL13* | -13.95147348 | 0.000261874 |
| ENSG00000211598 | *IGKV4-1* | -14.01247 | 0.003578115 |
| ENSG00000211666 | *IGLV2-14* | -14.14499687 | 0.007475351 |
| ENSG00000149451 | *ADAM33* | -14.17253387 | 2.07E-16 |
| ENSG00000173372 | *C1QA* | -14.23605123 | 8.56E-15 |
| ENSG00000269936 | *RP11-394O4.5* | -14.25588057 | 1.29E-15 |
| ENSG00000101938 | *CHRDL1* | -14.4753676 | 2.26E-15 |
| ENSG00000107317 | *PTGDS* | -14.73368459 | 2.79E-15 |
| ENSG00000132465 | *JCHAIN* | -14.91966562 | 3.12E-07 |
| ENSG00000211679 | *IGLC3* | -14.94015921 | 5.22E-07 |
| ENSG00000211592 | *IGKC* | -15.14283335 | 8.64E-06 |
| ENSG00000211642 | *IGLV10-54* | -23.4097953 | 2.02E-05 |
| ENSG00000111046 | *MYF6* | -23.45840612 | 1.94E-05 |
| ENSG00000186526 | *CYP4F8* | -23.6672002 | 1.60E-05 |
| ENSG00000163092 | *XIRP2* | -23.69121126 | 1.57E-05 |
| ENSG00000132704 | *FCRL2* | -23.82692273 | 1.38E-05 |
| ENSG00000211660 | *IGLV2-23* | -23.84036878 | 1.37E-05 |
| ENSG00000122180 | *MYOG* | -23.86400172 | 1.34E-05 |
| ENSG00000100721 | *TCL1A* | -23.92264188 | 1.27E-05 |
| ENSG00000282399 | *IGHV1-69-2* | -23.93598761 | 1.25E-05 |
| ENSG00000233209 | *HLA-DQB1* | -24.00917708 | 1.16E-05 |
| ENSG00000138100 | *TRIM54* | -24.09908896 | 1.07E-05 |
| ENSG00000170290 | *SLN* | -25.37538246 | 3.01E-06 |
| ENSG00000186439 | *TRDN* | -25.90903646 | 1.73E-06 |
| ENSG00000117215 | *PLA2G2D* | -25.95770873 | 1.65E-06 |
