# Supplementary material for: Epidermal Growth Factor Is Essential for the Maintenance of Novel Prostate Epithelial Cells Isolated From Patient-Derived Organoids
Source: Front Cell Dev Biol. 2020 Oct 29;8:571677. doi: 10.3389/fcell.2020.571677 (PMC7658326; doi:10.3389/fcell.2020.571677)
Supplement: Supplementary Table 6 — List of all differentially expressed genes (DEGs) between AUB-PrC cells and their tissue counterparts in tumor samples. [file Table_6.DOCX]

**Table S6. List of all differentially expressed genes (DEGs) between AUB-PrC cells and their tissue counterparts in tumor samples.**

| **Ensemble ID** | **Gene symbol** | **Fold-change (log base 2)** | ***p*-adjusted** |
| --- | --- | --- | --- |
| ENSG00000241749 | *RPSAP52* | 8.465194004 | 0.000590608 |
| ENSG00000163207 | *IVL* | 8.064218348 | 2.75E-05 |
| ENSG00000256268 | *RP11-221N13.3* | 7.987351234 | 0.002026177 |
| ENSG00000123364 | *HOXC13* | 7.707820856 | 0.00189619 |
| ENSG00000214049 | *UCA1* | 7.548650716 | 0.025247702 |
| ENSG00000175874 | *CREG2* | 7.518921491 | 1.42E-05 |
| ENSG00000111981 | *ULBP1* | 7.476891933 | 0.004190109 |
| ENSG00000148798 | *INA* | 7.46954612 | 1.03E-07 |
| ENSG00000182585 | *EPGN* | 7.345109178 | 0.000182357 |
| ENSG00000241635 | *UGT1A1* | 7.289754557 | 0.01955811 |
| ENSG00000253746 | *RP11-527N22.2* | 7.145006188 | 0.003258361 |
| ENSG00000136155 | *SCEL* | 7.085822556 | 7.12E-08 |
| ENSG00000249641 | *HOXC13-AS* | 7.036819794 |  |
| ENSG00000149948 | *HMGA2* | 6.890599091 | 4.27E-09 |
| ENSG00000239893 | *ZNF736P9Y* | 6.887262097 | 0.004336254 |
| ENSG00000110203 | *FOLR3* | 6.830111892 | 0.032771003 |
| ENSG00000242147 | *RP13-463N16.6* | 6.815113536 | 0.002489611 |
| ENSG00000256894 | *RP11-283G6.3* | 6.782185469 | 0.00355336 |
| ENSG00000166897 | *ELFN2* | 6.551102188 | 0.006527319 |
| ENSG00000247134 | *RP11-11N9.4* | 6.546679794 | 0.002855754 |
| ENSG00000188624 | *IGFL3* | 6.540517184 | 0.01543973 |
| ENSG00000249279 | *CTC-436P18.3* | 6.535895962 | 0.022346309 |
| ENSG00000265203 | *RBP3* | 6.51827903 | 0.034807877 |
| ENSG00000169258 | *GPRIN1* | 6.475400468 | 8.69E-06 |
| ENSG00000251493 | *FOXD1* | 6.391300507 | 0.0159207 |
| ENSG00000183729 | *NPBWR1* | 6.322026924 | 0.001427093 |
| ENSG00000183347 | *GBP6* | 6.241700991 | 1.74E-09 |
| ENSG00000115008 | *IL1A* | 6.212826644 | 0.000940796 |
| ENSG00000169469 | *SPRR1B* | 6.176785634 | 0.002819743 |
| ENSG00000147509 | *RGS20* | 6.14974556 | 1.85E-06 |
| ENSG00000110427 | *KIAA1549L* | 6.08801281 | 0.000367417 |
| ENSG00000138675 | *FGF5* | 6.049124854 | 0.002813709 |
| ENSG00000137440 | *FGFBP1* | 6.048192709 | 4.13E-07 |
| ENSG00000169474 | *SPRR1A* | 5.954647458 | 0.02394271 |
| ENSG00000186007 | *LEMD1* | 5.836868654 | 1.52E-08 |
| ENSG00000205325 | *AC005863.1* | 5.782194205 | 0.030786119 |
| ENSG00000148680 | *HTR7* | 5.773032155 | 4.15E-10 |
| ENSG00000114638 | *UPK1B* | 5.768132648 | 1.23E-06 |
| ENSG00000258479 | *LINC00640* | 5.763526718 | 0.024323006 |
| ENSG00000130829 | *DUSP9* | 5.763063387 | 0.004601998 |
| ENSG00000169174 | *PCSK9* | 5.726530173 | 0.045714274 |
| ENSG00000225362 | *CT62* | 5.69523437 | 0.031130496 |
| ENSG00000099194 | *SCD* | 5.659235474 | 1.25E-10 |
| ENSG00000169035 | *KLK7* | 5.634456859 | 6.91E-08 |
| ENSG00000175065 | *DSG4* | 5.604605789 | 0.017492482 |
| ENSG00000131015 | *ULBP2* | 5.560693112 | 0.002049954 |
| ENSG00000109674 | *NEIL3* | 5.559198024 | 0.000575433 |
| ENSG00000165474 | *GJB2* | 5.511060057 | 5.46E-06 |
| ENSG00000065328 | *MCM10* | 5.498120209 | 3.89E-06 |
| ENSG00000261786 | *RP4-555D20.2* | 5.483009286 | 0.023793036 |
| ENSG00000167165 | *UGT1A6* | 5.46275012 | 0.001910484 |
| ENSG00000214856 | *KRT16P1* | 5.461780513 | 0.000988351 |
| ENSG00000267218 | *AC005336.5* | 5.415435233 | 0.020374811 |
| ENSG00000121742 | *GJB6* | 5.401390388 | 9.91E-08 |
| ENSG00000189045 | *ANKDD1B* | 5.392953433 | 0.026382576 |
| ENSG00000169594 | *BNC1* | 5.376384718 | 1.51E-06 |
| ENSG00000131969 | *ABHD12B* | 5.363829225 | 0.026346432 |
| ENSG00000228951 | *RP11-336A10.4* | 5.335663791 | 0.014473542 |
| ENSG00000171877 | *FRMD5* | 5.32730975 | 3.31E-05 |
| ENSG00000167754 | *KLK5* | 5.326734339 | 0.000155466 |
| ENSG00000198074 | *AKR1B10* | 5.32138578 | 4.53E-12 |
| ENSG00000183780 | *SLC35F3* | 5.310403132 | 2.24E-05 |
| ENSG00000196754 | *S100A2* | 5.305995103 | 1.10E-05 |
| ENSG00000259153 | *RP6-65G23.3* | 5.296603995 | 2.97E-05 |
| ENSG00000128510 | *CPA4* | 5.272587558 | 0.000479429 |
| ENSG00000178934 | *LGALS7B* | 5.230649994 | 1.67E-06 |
| ENSG00000268621 | *AC006262.5* | 5.210883095 | 0.000148075 |
| ENSG00000189334 | *S100A14* | 5.180805726 | 1.39E-06 |
| ENSG00000176244 | *ACBD7* | 5.179131164 | 0.034793008 |
| ENSG00000147689 | *FAM83A* | 5.14216763 | 0.000434649 |
| ENSG00000095932 | *SMIM24* | 5.139315307 | 0.031488915 |
| ENSG00000107984 | *DKK1* | 5.103738095 | 5.41E-05 |
| ENSG00000134827 | *TCN1* | 5.081682105 | 3.95E-05 |
| ENSG00000124882 | *EREG* | 5.07533718 | 3.28E-09 |
| ENSG00000265763 | *ZNF488* | 5.073275841 | 0.000671876 |
| ENSG00000226145 | *KRT16P6* | 5.071657262 | 0.00013379 |
| ENSG00000105991 | *HOXA1* | 5.067252697 | 7.60E-07 |
| ENSG00000159784 | *FAM131B* | 5.050676674 | 1.94E-12 |
| ENSG00000204941 | *PSG5* | 5.041853564 | 0.027014952 |
| ENSG00000105825 | *TFPI2* | 5.021318959 | 1.53E-06 |
| ENSG00000206159 | *GYG2P1* | 5.000334033 | 1.18E-05 |
| ENSG00000129455 | *KLK8* | 4.998745529 | 0.000274969 |
| ENSG00000137975 | *CLCA2* | 4.992974653 | 6.71E-05 |
| ENSG00000229526 | *KRT16P4* | 4.988729589 | 0.000634864 |
| ENSG00000152056 | *AP1S3* | 4.987355118 | 5.25E-09 |
| ENSG00000205076 | *LGALS7* | 4.977747401 | 3.60E-05 |
| ENSG00000179133 | *C10orf67* | 4.967042572 | 0.009038328 |
| ENSG00000205420 | *KRT6A* | 4.95964855 | 0.000296104 |
| ENSG00000134757 | *DSG3* | 4.955553708 | 0.000111331 |
| ENSG00000115507 | *OTX1* | 4.926344042 | 0.028127806 |
| ENSG00000185069 | *KRT76* | 4.920550106 | 0.000203943 |
| ENSG00000057149 | *SERPINB3* | 4.917964903 | 0.000366471 |
| ENSG00000121743 | *GJA3* | 4.916310172 | 0.000115618 |
| ENSG00000167656 | *LY6D* | 4.906891084 | 0.001193883 |
| ENSG00000253161 | *LINC01605* | 4.90404272 | 0.00010338 |
| ENSG00000197632 | *SERPINB2* | 4.89843081 | 0.013945276 |
| ENSG00000256969 | *RP11-320N7.2* | 4.894409373 | 0.001537678 |
| ENSG00000184995 | *IFNE* | 4.869013466 | 7.08E-05 |
| ENSG00000238271 | *IFNWP19* | 4.862556185 | 3.09E-05 |
| ENSG00000176153 | *GPX2* | 4.836433574 | 6.93E-05 |
| ENSG00000065618 | *COL17A1* | 4.820388967 | 0.000108055 |
| ENSG00000206075 | *SERPINB5* | 4.811278394 | 2.47E-05 |
| ENSG00000174564 | *IL20RB* | 4.804745547 | 2.41E-06 |
| ENSG00000061337 | *LZTS1* | 4.78832295 | 1.13E-05 |
| ENSG00000281614 | *INPP5D* | 4.780681245 | 0.001117256 |
| ENSG00000186081 | *KRT5* | 4.773792617 | 0.000336497 |
| ENSG00000186115 | *CYP4F2* | 4.750407439 | 0.042954539 |
| ENSG00000182040 | *USH1G* | 4.744579167 | 0.016781138 |
| ENSG00000144834 | *TAGLN3* | 4.726933994 | 0.001019062 |
| ENSG00000055732 | *MCOLN3* | 4.687245822 | 0.000116978 |
| ENSG00000178919 | *FOXE1* | 4.666297739 | 0.000252899 |
| ENSG00000266680 | *RP5-1148A21.3* | 4.66499749 | 0.00769501 |
| ENSG00000058085 | *LAMC2* | 4.649886126 | 0.000662048 |
| ENSG00000010438 | *PRSS3* | 4.637276744 | 1.47E-05 |
| ENSG00000154764 | *WNT7A* | 4.623728165 | 0.000394973 |
| ENSG00000223784 | *RP11-554I8.2* | 4.615666037 | 0.005674392 |
| ENSG00000135925 | *WNT10A* | 4.601530086 | 0.000319609 |
| ENSG00000240891 | *PLCXD2* | 4.590377814 | 0.006930029 |
| ENSG00000260628 | *RP11-1166P10.1* | 4.589569156 | 0.023624878 |
| ENSG00000069812 | *HES2* | 4.563628703 | 0.001336408 |
| ENSG00000153294 | *ADGRF4* | 4.549317565 | 0.000589564 |
| ENSG00000136943 | *CTSV* | 4.534992979 | 1.18E-05 |
| ENSG00000125657 | *TNFSF9* | 4.532726344 | 2.87E-05 |
| ENSG00000100344 | *PNPLA3* | 4.528915139 | 0.01307563 |
| ENSG00000109805 | *NCAPG* | 4.527836443 | 2.13E-06 |
| ENSG00000156535 | *CD109* | 4.524365371 | 3.89E-09 |
| ENSG00000151012 | *SLC7A11* | 4.512468086 | 3.32E-08 |
| ENSG00000108602 | *ALDH3A1* | 4.4990523 | 0.001670289 |
| ENSG00000109321 | *AREG* | 4.489387585 | 7.99E-06 |
| ENSG00000062282 | *DGAT2* | 4.452582835 | 4.02E-06 |
| ENSG00000040608 | *RTN4R* | 4.43494622 | 1.37E-05 |
| ENSG00000179674 | *ARL14* | 4.422540284 | 0.024425519 |
| ENSG00000178363 | *CALML3* | 4.40464228 | 0.000833035 |
| ENSG00000171889 | *MIR31HG* | 4.402110786 | 0.000634343 |
| ENSG00000196542 | *SPTSSB* | 4.400292109 | 2.76E-07 |
| ENSG00000189057 | *FAM111B* | 4.387112877 | 1.32E-06 |
| ENSG00000186847 | *KRT14* | 4.366470301 | 6.16E-06 |
| ENSG00000120457 | *KCNJ5* | 4.36516646 | 0.000170191 |
| ENSG00000198729 | *PPP1R14C* | 4.350610909 | 5.32E-07 |
| ENSG00000169607 | *CKAP2L* | 4.318264701 | 5.93E-07 |
| ENSG00000205413 | *SAMD9* | 4.31806202 | 1.39E-11 |
| ENSG00000143476 | *DTL* | 4.313155378 | 4.16E-06 |
| ENSG00000120756 | *PLS1* | 4.299617746 | 3.47E-06 |
| ENSG00000158825 | *CDA* | 4.286540796 | 3.86E-09 |
| ENSG00000265415 | *CTD-2510F5.4* | 4.27337795 | 0.028074527 |
| ENSG00000035499 | *DEPDC1B* | 4.261804224 | 3.90E-07 |
| ENSG00000137819 | *PAQR5* | 4.259350269 | 1.81E-07 |
| ENSG00000189001 | *SBSN* | 4.258042484 | 0.015727938 |
| ENSG00000230088 | *KRT16P5* | 4.257583521 | 0.005704869 |
| ENSG00000158402 | *CDC25C* | 4.228517434 | 0.000374529 |
| ENSG00000203722 | *RAET1G* | 4.21649395 | 0.000480478 |
| ENSG00000265190 | *ANXA8* | 4.206766683 | 0.002831981 |
| ENSG00000081277 | *PKP1* | 4.199716108 | 0.000535865 |
| ENSG00000136492 | *BRIP1* | 4.19212668 | 7.45E-06 |
| ENSG00000144452 | *ABCA12* | 4.189503388 | 0.001917582 |
| ENSG00000125285 | *SOX21* | 4.16233617 | 0.002341556 |
| ENSG00000023909 | *GCLM* | 4.157946548 | 3.10E-09 |
| ENSG00000164520 | *RAET1E* | 4.153537921 | 0.00051 |
| ENSG00000227640 | *SOX21-AS1* | 4.149042861 | 0.004696493 |
| ENSG00000276850 | *CH17-360D5.2* | 4.145342622 | 0.003065919 |
| ENSG00000275216 | *RP11-54H7.4* | 4.143587782 | 3.25E-05 |
| ENSG00000078725 | *BRINP1* | 4.139495362 | 0.000614063 |
| ENSG00000101680 | *LAMA1* | 4.12767778 | 0.004461844 |
| ENSG00000066279 | *ASPM* | 4.110606226 | 1.11E-07 |
| ENSG00000118193 | *KIF14* | 4.108039377 | 6.15E-06 |
| ENSG00000089250 | *NOS1* | 4.092161917 | 0.012282302 |
| ENSG00000109255 | *NMU* | 4.084579409 | 0.012693215 |
| ENSG00000163331 | *DAPL1* | 4.082415889 | 6.41E-08 |
| ENSG00000205488 | *CALML3-AS1* | 4.07532165 | 0.013749009 |
| ENSG00000086570 | *FAT2* | 4.071297949 | 0.017812427 |
| ENSG00000069188 | *SDK2* | 4.070681141 | 0.001395752 |
| ENSG00000174371 | *EXO1* | 4.068744167 | 3.45E-05 |
| ENSG00000087494 | *PTHLH* | 4.067847635 | 0.010458113 |
| ENSG00000046604 | *DSG2* | 4.066128007 | 1.91E-05 |
| ENSG00000171320 | *ESCO2* | 4.063088316 | 0.000257413 |
| ENSG00000126787 | *DLGAP5* | 4.061985706 | 4.15E-05 |
| ENSG00000101670 | *LIPG* | 4.036804804 | 0.001107908 |
| ENSG00000124343 | *XG* | 4.033513828 | 0.00026685 |
| ENSG00000054277 | *OPN3* | 4.032490407 | 0.003837223 |
| ENSG00000245648 | *RP11-277P12.20* | 4.03111654 | 0.000344271 |
| ENSG00000177494 | *ZBED2* | 4.021628518 | 0.007289633 |
| ENSG00000145934 | *TENM2* | 4.016190401 | 0.002070475 |
| ENSG00000134762 | *DSC3* | 3.992712676 | 0.003204643 |
| ENSG00000164929 | *BAALC* | 3.957662374 | 0.005805708 |
| ENSG00000111863 | *ADTRP* | 3.955057406 | 0.033536102 |
| ENSG00000143126 | *CELSR2* | 3.951337066 | 0.003870212 |
| ENSG00000103044 | *HAS3* | 3.950005203 | 4.86E-05 |
| ENSG00000139734 | *DIAPH3* | 3.945550419 | 0.000152902 |
| ENSG00000088726 | *TMEM40* | 3.926271175 | 0.013974901 |
| ENSG00000101842 | *VSIG1* | 3.925567986 | 0.032517454 |
| ENSG00000264230 | *ANXA8L1* | 3.918181766 | 0.010160928 |
| ENSG00000164251 | *F2RL1* | 3.917855183 | 5.25E-05 |
| ENSG00000156869 | *FRRS1* | 3.916781922 | 0.000493546 |
| ENSG00000133101 | *CCNA1* | 3.912902476 | 0.025971135 |
| ENSG00000090889 | *KIF4A* | 3.911888072 | 1.30E-05 |
| ENSG00000188610 | *FAM72B* | 3.871651041 | 0.03596591 |
| ENSG00000185761 | *ADAMTSL5* | 3.865669625 | 1.41E-05 |
| ENSG00000168032 | *ENTPD3* | 3.855472643 | 1.89E-08 |
| ENSG00000267374 | *RP11-244M2.1* | 3.850827559 | 0.026993797 |
| ENSG00000169679 | *BUB1* | 3.847047369 | 4.57E-05 |
| ENSG00000181333 | *HEPHL1* | 3.846690286 | 0.046525586 |
| ENSG00000135069 | *PSAT1* | 3.829839072 | 4.67E-06 |
| ENSG00000272398 | *CD24* | 3.827319186 | 1.45E-09 |
| ENSG00000070731 | *ST6GALNAC2* | 3.823180328 | 7.70E-05 |
| ENSG00000112414 | *ADGRG6* | 3.818217224 | 4.60E-05 |
| ENSG00000052802 | *MSMO1* | 3.815175 | 4.93E-10 |
| ENSG00000121690 | *DEPDC7* | 3.809937577 | 6.53E-05 |
| ENSG00000112742 | *TTK* | 3.808319235 | 1.88E-06 |
| ENSG00000156970 | *BUB1B* | 3.807636266 | 2.10E-05 |
| ENSG00000115884 | *SDC1* | 3.802031227 | 0.001544613 |
| ENSG00000178947 | *SMIM10L2A* | 3.801075102 | 0.000494189 |
| ENSG00000281406 | *BLACAT1* | 3.78496063 | 0.011017538 |
| ENSG00000112984 | *KIF20A* | 3.76733977 | 1.89E-06 |
| ENSG00000139988 | *RDH12* | 3.750371559 | 0.011541437 |
| ENSG00000140945 | *CDH13* | 3.748937798 | 0.000212437 |
| ENSG00000196083 | *IL1RAP* | 3.748264355 | 1.07E-05 |
| ENSG00000100504 | *PYGL* | 3.74471623 | 8.78E-07 |
| ENSG00000159166 | *LAD1* | 3.735018038 | 0.008555901 |
| ENSG00000117650 | *NEK2* | 3.720183547 | 3.31E-05 |
| ENSG00000185479 | *KRT6B* | 3.718205696 | 0.035252027 |
| ENSG00000099219 | *ERMP1* | 3.714439216 | 6.35E-05 |
| ENSG00000103034 | *NDRG4* | 3.712234236 | 0.002198785 |
| ENSG00000163293 | *NIPAL1* | 3.711837984 | 0.002155213 |
| ENSG00000075618 | *FSCN1* | 3.71165389 | 1.93E-05 |
| ENSG00000101144 | *BMP7* | 3.711305517 | 0.002513573 |
| ENSG00000165304 | *MELK* | 3.707981813 | 2.42E-06 |
| ENSG00000170006 | *TMEM154* | 3.704914609 | 1.37E-05 |
| ENSG00000110400 | *PVRL1* | 3.702499079 | 0.016480874 |
| ENSG00000101255 | *TRIB3* | 3.699492079 | 0.000938067 |
| ENSG00000123892 | *RAB38* | 3.694145646 | 6.71E-11 |
| ENSG00000029153 | *ARNTL2* | 3.693197165 | 0.000115973 |
| ENSG00000109182 | *CWH43* | 3.680088655 | 0.016392675 |
| ENSG00000121152 | *NCAPH* | 3.677121276 | 0.000174129 |
| ENSG00000053747 | *LAMA3* | 3.673532089 | 0.0001688 |
| ENSG00000136689 | *IL1RN* | 3.660845343 | 0.001760558 |
| ENSG00000268592 | *RAET1E-AS1* | 3.660728585 | 0.022031676 |
| ENSG00000150782 | *IL18* | 3.652742132 | 1.04E-05 |
| ENSG00000164171 | *ITGA2* | 3.644880661 | 0.000192733 |
| ENSG00000093009 | *CDC45* | 3.642012376 | 0.000286268 |
| ENSG00000134755 | *DSC2* | 3.639637326 | 0.000115973 |
| ENSG00000186871 | *ERCC6L* | 3.638447456 | 0.000207817 |
| ENSG00000138829 | *FBN2* | 3.635362973 | 0.000595943 |
| ENSG00000073792 | *IGF2BP2* | 3.619368028 | 1.65E-06 |
| ENSG00000133808 | *MICALCL* | 3.614008315 | 0.008920344 |
| ENSG00000172738 | *TMEM217* | 3.603789806 | 0.0209041 |
| ENSG00000096696 | *DSP* | 3.599643266 | 0.006212415 |
| ENSG00000197261 | *C6orf141* | 3.599482428 | 0.005184764 |
| ENSG00000137309 | *HMGA1* | 3.598834575 | 0.000178341 |
| ENSG00000184661 | *CDCA2* | 3.598017559 | 9.19E-05 |
| ENSG00000163032 | *VSNL1* | 3.586207298 | 0.000564394 |
| ENSG00000186831 | *KRT17P2* | 3.584069569 | 0.031077309 |
| ENSG00000086696 | *HSD17B2* | 3.574707382 | 0.005415873 |
| ENSG00000175793 | *SFN* | 3.574486309 | 0.004327948 |
| ENSG00000163689 | *C3orf67* | 3.574165323 | 0.004354438 |
| ENSG00000163535 | *SGOL2* | 3.567935878 | 4.92E-06 |
| ENSG00000177076 | *ACER2* | 3.565912511 | 0.008167938 |
| ENSG00000104549 | *SQLE* | 3.552980404 | 2.05E-10 |
| ENSG00000051341 | *POLQ* | 3.547900772 | 5.82E-05 |
| ENSG00000139438 | *FAM222A* | 3.543350024 | 0.009584755 |
| ENSG00000068489 | *PRR11* | 3.53262001 | 0.000134166 |
| ENSG00000180739 | *S1PR5* | 3.532370927 | 0.002052459 |
| ENSG00000128641 | *MYO1B* | 3.529440493 | 1.17E-06 |
| ENSG00000131747 | *TOP2A* | 3.518753488 | 9.74E-08 |
| ENSG00000120471 | *TP53AIP1* | 3.503740694 | 0.00127756 |
| ENSG00000105376 | *ICAM5* | 3.494747899 | 0.000351832 |
| ENSG00000198018 | *ENTPD7* | 3.488555047 | 0.001967744 |
| ENSG00000103257 | *SLC7A5* | 3.486918481 | 0.000988774 |
| ENSG00000165891 | *E2F7* | 3.486178503 | 0.001252046 |
| ENSG00000051180 | *RAD51* | 3.482791778 | 0.000962988 |
| ENSG00000172893 | *DHCR7* | 3.47624757 | 2.04E-05 |
| ENSG00000143320 | *CRABP2* | 3.475025857 | 8.92E-05 |
| ENSG00000012048 | *BRCA1* | 3.469454047 | 3.10E-05 |
| ENSG00000133135 | *RNF128* | 3.468206454 | 0.000163314 |
| ENSG00000231991 | *ANXA2P2* | 3.455706813 | 3.62E-08 |
| ENSG00000157168 | *NRG1* | 3.454725627 | 0.000186167 |
| ENSG00000275880 | *RP11-90L1.8* | 3.450853008 | 0.030176758 |
| ENSG00000129810 | *SGOL1* | 3.442140695 | 0.015220221 |
| ENSG00000163931 | *TKT* | 3.437267998 | 3.97E-06 |
| ENSG00000140450 | *ARRDC4* | 3.433962022 | 2.36E-10 |
| ENSG00000169752 | *NRG4* | 3.427058651 | 0.03194308 |
| ENSG00000178826 | *TMEM139* | 3.42402157 | 0.009649108 |
| ENSG00000113578 | *FGF1* | 3.420116155 | 0.003134493 |
| ENSG00000227300 | *KRT16P2* | 3.403423 | 0.03647551 |
| ENSG00000169710 | *FASN* | 3.402964813 | 0.011393759 |
| ENSG00000163235 | *TGFA* | 3.396783748 | 0.000388806 |
| ENSG00000186212 | *SOWAHB* | 3.389331779 | 0.023192506 |
| ENSG00000099960 | *SLC7A4* | 3.388636591 | 0.000305007 |
| ENSG00000128346 | *C22orf23* | 3.382678111 | 0.000191224 |
| ENSG00000101311 | *FERMT1* | 3.380609171 | 0.00574489 |
| ENSG00000188910 | *GJB3* | 3.376469345 | 0.003391255 |
| ENSG00000106714 | *CNTNAP3* | 3.371598004 | 0.014305146 |
| ENSG00000100003 | *SEC14L2* | 3.35201456 | 0.000188905 |
| ENSG00000171208 | *NETO2* | 3.350942607 | 0.002659842 |
| ENSG00000011426 | *ANLN* | 3.348094682 | 0.000100134 |
| ENSG00000196878 | *LAMB3* | 3.347101402 | 0.020766884 |
| ENSG00000006118 | *TMEM132A* | 3.346436953 | 0.00094237 |
| ENSG00000117724 | *CENPF* | 3.345798903 | 5.41E-05 |
| ENSG00000179593 | *ALOX15B* | 3.345154548 | 0.014102235 |
| ENSG00000094804 | *CDC6* | 3.342949634 | 4.69E-06 |
| ENSG00000170537 | *TMC7* | 3.333057165 | 6.78E-05 |
| ENSG00000165480 | *SKA3* | 3.329430568 | 0.009571506 |
| ENSG00000113161 | *HMGCR* | 3.324374027 | 2.51E-09 |
| ENSG00000156011 | *PSD3* | 3.320700542 | 6.02E-05 |
| ENSG00000021826 | *CPS1* | 3.305356178 | 0.008199008 |
| ENSG00000134824 | *FADS2* | 3.302622969 | 1.05E-06 |
| ENSG00000146410 | *MTFR2* | 3.294130156 | 0.008400163 |
| ENSG00000154227 | *CERS3* | 3.291586687 | 0.029549268 |
| ENSG00000136108 | *CKAP2* | 3.28769193 | 1.24E-07 |
| ENSG00000158292 | *GPR153* | 3.286422566 | 1.35E-05 |
| ENSG00000170312 | *CDK1* | 3.276293757 | 3.67E-06 |
| ENSG00000164825 | *DEFB1* | 3.276289971 | 0.000177884 |
| ENSG00000172548 | *NIPAL4* | 3.274011967 | 0.000371806 |
| ENSG00000107731 | *UNC5B* | 3.267511037 | 0.023036676 |
| ENSG00000144063 | *MALL* | 3.264161998 | 0.001025133 |
| ENSG00000091409 | *ITGA6* | 3.263625473 | 5.03E-05 |
| ENSG00000145824 | *CXCL14* | 3.26089307 | 0.03204699 |
| ENSG00000057019 | *DCBLD2* | 3.260794531 | 1.32E-06 |
| ENSG00000092853 | *CLSPN* | 3.25889704 | 0.002896121 |
| ENSG00000104892 | *KLC3* | 3.256698835 | 0.000256786 |
| ENSG00000151693 | *ASAP2* | 3.25443116 | 0.014351712 |
| ENSG00000168078 | *PBK* | 3.247093836 | 0.000504925 |
| ENSG00000163993 | *S100P* | 3.244260955 | 0.048845381 |
| ENSG00000071539 | *TRIP13* | 3.24197544 | 0.000516474 |
| ENSG00000183840 | *GPR39* | 3.236902523 | 0.031330918 |
| ENSG00000164086 | *DUSP7* | 3.222803961 | 1.90E-07 |
| ENSG00000026508 | *CD44* | 3.217615039 | 4.37E-06 |
| ENSG00000187210 | *GCNT1* | 3.212654554 | 0.000247957 |
| ENSG00000276600 | *RAB7B* | 3.209979247 | 0.001138806 |
| ENSG00000165490 | *DDIAS* | 3.206804999 | 0.003877877 |
| ENSG00000230943 | *RP11-367G18.1* | 3.199815934 | 0.02988979 |
| ENSG00000170425 | *ADORA2B* | 3.199160588 | 3.60E-05 |
| ENSG00000171848 | *RRM2* | 3.195017357 | 1.18E-05 |
| ENSG00000133710 | *SPINK5* | 3.195007265 | 0.018759344 |
| ENSG00000147676 | *MAL2* | 3.193120376 | 0.006870322 |
| ENSG00000138160 | *KIF11* | 3.189105803 | 1.32E-06 |
| ENSG00000171517 | *LPAR3* | 3.183985897 | 0.017621878 |
| ENSG00000104413 | *ESRP1* | 3.181045201 | 0.007791774 |
| ENSG00000173805 | *HAP1* | 3.179855158 | 0.029066075 |
| ENSG00000138182 | *KIF20B* | 3.170145086 | 9.04E-05 |
| ENSG00000088325 | *TPX2* | 3.166954191 | 1.79E-06 |
| ENSG00000198088 | *NUP62CL* | 3.166126499 | 0.008301388 |
| ENSG00000064300 | *NGFR* | 3.1581801 | 0.00434866 |
| ENSG00000213713 | *PIGCP1* | 3.145509251 | 0.035380181 |
| ENSG00000139318 | *DUSP6* | 3.145484635 | 0.001799113 |
| ENSG00000139514 | *SLC7A1* | 3.130395253 | 0.001970077 |
| ENSG00000005884 | *ITGA3* | 3.128512431 | 0.003000444 |
| ENSG00000151136 | *BTBD11* | 3.127291527 | 0.001319671 |
| ENSG00000164379 | *FOXQ1* | 3.124073976 | 0.035783166 |
| ENSG00000153044 | *CENPH* | 3.123652014 | 1.88E-06 |
| ENSG00000196581 | *AJAP1* | 3.122149445 | 0.033345349 |
| ENSG00000185585 | *OLFML2A* | 3.115415625 | 0.001575152 |
| ENSG00000149256 | *TENM4* | 3.110697571 | 0.0037201 |
| ENSG00000111424 | *VDR* | 3.104909364 | 0.028867044 |
| ENSG00000167513 | *CDT1* | 3.103991449 | 0.000250535 |
| ENSG00000186832 | *KRT16* | 3.100431275 | 0.009187754 |
| ENSG00000140284 | *SLC27A2* | 3.094539921 | 0.002732454 |
| ENSG00000139304 | *PTPRQ* | 3.09300179 | 0.035490629 |
| ENSG00000075275 | *CELSR1* | 3.088350686 | 0.011777581 |
| ENSG00000100558 | *PLEK2* | 3.086435338 | 0.000773236 |
| ENSG00000070614 | *NDST1* | 3.085858065 | 0.006551734 |
| ENSG00000102384 | *CENPI* | 3.085109102 | 0.024634473 |
| ENSG00000117595 | *IRF6* | 3.073956847 | 0.012509707 |
| ENSG00000189280 | *GJB5* | 3.069931959 | 0.016657064 |
| ENSG00000133216 | *EPHB2* | 3.062297524 | 0.002761424 |
| ENSG00000129451 | *KLK10* | 3.061370187 | 0.005151196 |
| ENSG00000182718 | *ANXA2* | 3.061182869 | 4.91E-07 |
| ENSG00000137812 | *CASC5* | 3.060985315 | 0.0015087 |
| ENSG00000175591 | *P2RY2* | 3.058942144 | 0.023243777 |
| ENSG00000267750 | *RUNDC3A-AS1* | 3.058807626 | 0.048060848 |
| ENSG00000089356 | *FXYD3* | 3.055776478 | 0.001381666 |
| ENSG00000072571 | *HMMR* | 3.054842932 | 0.000511356 |
| ENSG00000006432 | *MAP3K9* | 3.045098067 | 0.027602957 |
| ENSG00000187123 | *LYPD6* | 3.033568064 | 0.011300826 |
| ENSG00000124466 | *LYPD3* | 3.033319631 | 0.022210895 |
| ENSG00000138778 | *CENPE* | 3.027613551 | 0.000858498 |
| ENSG00000136802 | *LRRC8A* | 3.02389367 | 0.000160959 |
| ENSG00000001084 | *GCLC* | 3.017670511 | 0.000125187 |
| ENSG00000102471 | *NDFIP2* | 3.016976802 | 2.64E-05 |
| ENSG00000145555 | *MYO10* | 3.001275454 | 0.000768965 |
| ENSG00000164543 | *STK17A* | 2.998440702 | 4.70E-05 |
| ENSG00000107438 | *PDLIM1* | 2.997987311 | 4.17E-06 |
| ENSG00000171345 | *KRT19* | 2.997424781 | 0.049168839 |
| ENSG00000148773 | *MKI67* | 2.99386636 | 0.002552909 |
| ENSG00000112297 | *AIM1* | 2.992827144 | 0.004945997 |
| ENSG00000170421 | *KRT8* | 2.992323785 | 0.000434145 |
| ENSG00000179431 | *FJX1* | 2.990561596 | 0.0049072 |
| ENSG00000117394 | *SLC2A1* | 2.988766445 | 2.43E-05 |
| ENSG00000105810 | *CDK6* | 2.988439994 | 0.000284458 |
| ENSG00000164070 | *HSPA4L* | 2.98461092 | 0.000121433 |
| ENSG00000278948 | *RP5-1039K5.12* | 2.983874682 | 0.008106641 |
| ENSG00000197747 | *S100A10* | 2.971285143 | 0.000202061 |
| ENSG00000129195 | *FAM64A* | 2.96725869 | 0.004364136 |
| ENSG00000154127 | *UBASH3B* | 2.964540841 | 0.00121855 |
| ENSG00000073282 | *TP63* | 2.959537616 | 0.012572114 |
| ENSG00000198774 | *RASSF9* | 2.955562175 | 0.001501193 |
| ENSG00000163624 | *CDS1* | 2.953768818 | 0.002554723 |
| ENSG00000259768 | *RP5-991G20.1* | 2.943713575 | 0.002069846 |
| ENSG00000140950 | *TLDC1* | 2.941600246 | 2.35E-05 |
| ENSG00000138271 | *GPR87* | 2.940630215 | 0.003842041 |
| ENSG00000116133 | *DHCR24* | 2.938651275 | 0.009074145 |
| ENSG00000164181 | *ELOVL7* | 2.935895234 | 0.001265223 |
| ENSG00000117399 | *CDC20* | 2.934006137 | 0.001420085 |
| ENSG00000135378 | *PRRG4* | 2.932292016 | 0.005227616 |
| ENSG00000198589 | *LRBA* | 2.928607749 | 0.000432348 |
| ENSG00000115163 | *CENPA* | 2.92130543 | 0.020499968 |
| ENSG00000111206 | *FOXM1* | 2.914100728 | 0.000318175 |
| ENSG00000164930 | *FZD6* | 2.904256724 | 5.33E-05 |
| ENSG00000184731 | *FAM110C* | 2.903280409 | 0.032524703 |
| ENSG00000138180 | *CEP55* | 2.898983196 | 0.001164925 |
| ENSG00000147536 | *GINS4* | 2.896866468 | 0.019998917 |
| ENSG00000164465 | *DCBLD1* | 2.896756852 | 0.000144847 |
| ENSG00000164611 | *PTTG1* | 2.895344012 | 0.002450748 |
| ENSG00000157193 | *LRP8* | 2.893731036 | 0.000221491 |
| ENSG00000133477 | *FAM83F* | 2.888193095 | 0.038349381 |
| ENSG00000182010 | *RTKN2* | 2.885795742 | 0.00256584 |
| ENSG00000120256 | *LRP11* | 2.878287537 | 5.76E-05 |
| ENSG00000185668 | *POU3F1* | 2.877832675 | 0.005526065 |
| ENSG00000121621 | *KIF18A* | 2.875152413 | 0.011635753 |
| ENSG00000160285 | *LSS* | 2.869909128 | 0.020499968 |
| ENSG00000121552 | *CSTA* | 2.868639976 | 4.70E-06 |
| ENSG00000198901 | *PRC1* | 2.867429634 | 2.45E-05 |
| ENSG00000225526 | *MKRN2OS* | 2.865326821 | 0.040851923 |
| ENSG00000186767 | *SPIN4* | 2.863676391 | 0.000308424 |
| ENSG00000168824 | *D4S234E* | 2.860544041 | 0.011189247 |
| ENSG00000168615 | *ADAM9* | 2.853641321 | 4.61E-05 |
| ENSG00000227036 | *LINC00511* | 2.847567773 | 0.035751934 |
| ENSG00000062038 | *CDH3* | 2.847087872 | 0.014225013 |
| ENSG00000169403 | *PTAFR* | 2.839404145 | 0.00262921 |
| ENSG00000151725 | *CENPU* | 2.838134485 | 0.000293549 |
| ENSG00000122861 | *PLAU* | 2.837779207 | 0.00559446 |
| ENSG00000127564 | *PKMYT1* | 2.835169 | 0.02705061 |
| ENSG00000095587 | *TLL2* | 2.834120828 | 0.003476687 |
| ENSG00000188643 | *S100A16* | 2.833011807 | 8.63E-07 |
| ENSG00000074410 | *CA12* | 2.830433119 | 0.000354346 |
| ENSG00000128422 | *KRT17* | 2.818817402 | 0.036140534 |
| ENSG00000168528 | *SERINC2* | 2.81602563 | 0.032445604 |
| ENSG00000143061 | *IGSF3* | 2.814637033 | 0.008987317 |
| ENSG00000134057 | *CCNB1* | 2.813870079 | 0.000189614 |
| ENSG00000140525 | *FANCI* | 2.803709349 | 8.57E-05 |
| ENSG00000066248 | *NGEF* | 2.802358693 | 0.036222378 |
| ENSG00000174939 | *ASPHD1* | 2.798075543 | 0.001594131 |
| ENSG00000276043 | *UHRF1* | 2.795918821 | 0.0006063 |
| ENSG00000186185 | *KIF18B* | 2.791399508 | 0.00300411 |
| ENSG00000246640 | *RP11-1094H24.4* | 2.787643459 | 0.048907505 |
| ENSG00000143228 | *NUF2* | 2.786577847 | 0.014259664 |
| ENSG00000163814 | *CDCP1* | 2.783352877 | 0.018383998 |
| ENSG00000182749 | *PAQR7* | 2.781094933 | 0.00358847 |
| ENSG00000169247 | *SH3TC2* | 2.777334293 | 0.00873425 |
| ENSG00000165244 | *ZNF367* | 2.774701306 | 0.004556097 |
| ENSG00000168672 | *FAM84B* | 2.774009355 | 0.001342252 |
| ENSG00000134690 | *CDCA8* | 2.773871248 | 0.006972146 |
| ENSG00000129474 | *AJUBA* | 2.773546686 | 0.004215157 |
| ENSG00000185164 | *NOMO2* | 2.772472428 | 0.037255929 |
| ENSG00000124429 | *POF1B* | 2.768244524 | 0.030540422 |
| ENSG00000198743 | *SLC5A3* | 2.766536121 | 0.000695626 |
| ENSG00000095539 | *SEMA4G* | 2.766354543 | 0.04856368 |
| ENSG00000041353 | *RAB27B* | 2.760732836 | 0.009087886 |
| ENSG00000156453 | *PCDH1* | 2.760160832 | 0.006353324 |
| ENSG00000170921 | *TANC2* | 2.759994231 | 0.00763493 |
| ENSG00000105011 | *ASF1B* | 2.757898783 | 0.001628093 |
| ENSG00000184564 | *SLITRK6* | 2.757583667 | 5.85E-05 |
| ENSG00000142731 | *PLK4* | 2.757345054 | 0.001785241 |
| ENSG00000091651 | *ORC6* | 2.746247931 | 0.000494864 |
| ENSG00000136542 | *GALNT5* | 2.745874 | 0.011865429 |
| ENSG00000174028 | *FAM3C2* | 2.745443012 | 0.009739141 |
| ENSG00000085999 | *RAD54L* | 2.744685995 | 0.012205107 |
| ENSG00000149548 | *CCDC15* | 2.744375871 | 0.034920248 |
| ENSG00000261116 | *RP3-523K23.2* | 2.743705242 | 0.002069846 |
| ENSG00000145632 | *PLK2* | 2.739233413 | 0.002372699 |
| ENSG00000123485 | *HJURP* | 2.737096331 | 0.003189252 |
| ENSG00000140022 | *STON2* | 2.734901273 | 9.36E-05 |
| ENSG00000145386 | *CCNA2* | 2.732646221 | 0.000521338 |
| ENSG00000063660 | *GPC1* | 2.729309695 | 0.009074145 |
| ENSG00000115363 | *EVA1A* | 2.727391398 | 0.037609217 |
| ENSG00000137804 | *NUSAP1* | 2.721809677 | 8.30E-05 |
| ENSG00000168143 | *FAM83B* | 2.718625659 | 0.027589296 |
| ENSG00000139618 | *BRCA2* | 2.716303613 | 0.00695748 |
| ENSG00000112378 | *PERP* | 2.710130627 | 0.000576801 |
| ENSG00000146670 | *CDCA5* | 2.703488135 | 0.002638214 |
| ENSG00000101003 | *GINS1* | 2.698091426 | 0.000463401 |
| ENSG00000158315 | *RHBDL2* | 2.696462511 | 0.034027704 |
| ENSG00000158125 | *XDH* | 2.695263955 | 0.005003514 |
| ENSG00000242265 | *PEG10* | 2.689820583 | 0.007321076 |
| ENSG00000115339 | *GALNT3* | 2.687093918 | 0.005664652 |
| ENSG00000198142 | *SOWAHC* | 2.679483851 | 6.70E-05 |
| ENSG00000237476 | *XXbac-B135H6.15* | 2.676385944 | 0.024425519 |
| ENSG00000187951 | *ARHGAP11B* | 2.676188042 | 0.012421991 |
| ENSG00000157456 | *CCNB2* | 2.674299536 | 0.004677657 |
| ENSG00000104738 | *MCM4* | 2.671425806 | 0.000116164 |
| ENSG00000161714 | *PLCD3* | 2.670725163 | 0.002929344 |
| ENSG00000187244 | *BCAM* | 2.669417461 | 0.0151385 |
| ENSG00000114346 | *ECT2* | 2.666689501 | 0.000689894 |
| ENSG00000105514 | *RAB3D* | 2.658962459 | 0.007450102 |
| ENSG00000278318 | *ZNF229* | 2.658759864 | 0.009187217 |
| ENSG00000161800 | *RACGAP1* | 2.658409897 | 0.000988351 |
| ENSG00000198554 | *WDHD1* | 2.657061885 | 0.000689894 |
| ENSG00000121207 | *LRAT* | 2.656282541 | 0.045570246 |
| ENSG00000176532 | *PRR15* | 2.649533673 | 0.04556486 |
| ENSG00000139629 | *GALNT6* | 2.64403208 | 0.009638477 |
| ENSG00000183723 | *CMTM4* | 2.638024935 | 0.006884524 |
| ENSG00000151715 | *TMEM45B* | 2.635753224 | 0.044307873 |
| ENSG00000142910 | *TINAGL1* | 2.633100564 | 0.002661863 |
| ENSG00000137962 | *ARHGAP29* | 2.630995043 | 0.001320944 |
| ENSG00000161682 | *FAM171A2* | 2.630712881 | 0.017492482 |
| ENSG00000067167 | *TRAM1* | 2.628215013 | 0.002844114 |
| ENSG00000203727 | *SAMD5* | 2.625584295 | 0.005277218 |
| ENSG00000084731 | *KIF3C* | 2.623874509 | 0.000784957 |
| ENSG00000175832 | *ETV4* | 2.623178664 | 0.010533904 |
| ENSG00000129194 | *SOX15* | 2.621433506 | 0.000744515 |
| ENSG00000123219 | *CENPK* | 2.617509601 | 0.002600603 |
| ENSG00000050405 | *LIMA1* | 2.617289657 | 5.65E-05 |
| ENSG00000143641 | *GALNT2* | 2.616442329 | 0.002987666 |
| ENSG00000073111 | *MCM2* | 2.606931425 | 0.002550626 |
| ENSG00000215458 | *AATBC* | 2.596894927 | 0.005836884 |
| ENSG00000166845 | *C18orf54* | 2.595161934 | 0.006627297 |
| ENSG00000160211 | *G6PD* | 2.588975446 | 0.001196043 |
| ENSG00000138119 | *MYOF* | 2.586590744 | 0.005742387 |
| ENSG00000171346 | *KRT15* | 2.586069507 | 0.033843828 |
| ENSG00000151665 | *PIGF* | 2.582028287 | 0.000320059 |
| ENSG00000091136 | *LAMB1* | 2.580546489 | 0.001030235 |
| ENSG00000072133 | *RPS6KA6* | 2.579717129 | 0.006946425 |
| ENSG00000165895 | *ARHGAP42* | 2.579185121 | 0.007208961 |
| ENSG00000173218 | *VANGL1* | 2.578415758 | 0.001200864 |
| ENSG00000123095 | *BHLHE41* | 2.577784604 | 0.001931172 |
| ENSG00000118242 | *MREG* | 2.576878688 | 0.001105304 |
| ENSG00000140534 | *TICRR* | 2.575431635 | 0.046677579 |
| ENSG00000109667 | *SLC2A9* | 2.562572941 | 0.00323669 |
| ENSG00000157483 | *MYO1E* | 2.558211664 | 0.000308722 |
| ENSG00000178999 | *AURKB* | 2.554396632 | 0.005798532 |
| ENSG00000169118 | *CSNK1G1* | 2.550223252 | 0.001835778 |
| ENSG00000181019 | *NQO1* | 2.54689407 | 0.014244033 |
| ENSG00000106484 | *MEST* | 2.546612868 | 0.001976811 |
| ENSG00000143797 | *MBOAT2* | 2.537951326 | 0.001353378 |
| ENSG00000075218 | *GTSE1* | 2.537068791 | 0.011675482 |
| ENSG00000183762 | *KREMEN1* | 2.535485466 | 0.013617227 |
| ENSG00000111816 | *FRK* | 2.529012909 | 0.005919561 |
| ENSG00000090776 | *EFNB1* | 2.525694116 | 7.95E-05 |
| ENSG00000143324 | *XPR1* | 2.525141673 | 0.000272864 |
| ENSG00000163191 | *S100A11* | 2.524732756 | 3.13E-05 |
| ENSG00000151914 | *DST* | 2.523779363 | 0.002440712 |
| ENSG00000257084 | *U47924.27* | 2.523194607 | 0.002244496 |
| ENSG00000083857 | *FAT1* | 2.519518548 | 0.039986924 |
| ENSG00000268858 | *RP4-591C20.9* | 2.506532134 | 0.029066075 |
| ENSG00000164109 | *MAD2L1* | 2.501950419 | 0.000809376 |
| ENSG00000115221 | *ITGB6* | 2.499008657 | 0.009074145 |
| ENSG00000132698 | *RAB25* | 2.498810058 | 0.018776292 |
| ENSG00000188522 | *FAM83G* | 2.495846893 | 0.017760483 |
| ENSG00000123689 | *G0S2* | 2.489110857 | 0.015532401 |
| ENSG00000171444 | *MCC* | 2.488830622 | 0.009342829 |
| ENSG00000169047 | *IRS1* | 2.485205903 | 0.011990903 |
| ENSG00000060749 | *QSER1* | 2.484215586 | 0.000380065 |
| ENSG00000136114 | *THSD1* | 2.483044289 | 0.006684002 |
| ENSG00000105976 | *MET* | 2.482918328 | 0.002520995 |
| ENSG00000173801 | *JUP* | 2.481979481 | 0.026169337 |
| ENSG00000230937 | *MIR205HG* | 2.477628224 | 0.040505195 |
| ENSG00000173599 | *PC* | 2.475080809 | 0.006500476 |
| ENSG00000198911 | *SREBF2* | 2.474216759 | 0.001796923 |
| ENSG00000049130 | *KITLG* | 2.471701504 | 0.004186596 |
| ENSG00000100139 | *MICALL1* | 2.470297323 | 0.011341471 |
| ENSG00000176619 | *LMNB2* | 2.467736836 | 0.003000444 |
| ENSG00000087586 | *AURKA* | 2.46190165 | 0.001223611 |
| ENSG00000166803 | *KIAA0101* | 2.454480346 | 0.001771265 |
| ENSG00000154102 | *C16orf74* | 2.45397841 | 0.000660737 |
| ENSG00000149212 | *SESN3* | 2.451044681 | 0.000632008 |
| ENSG00000089685 | *BIRC5* | 2.448098569 | 0.007272457 |
| ENSG00000223478 | *RP11-545E17.3* | 2.446379124 | 0.003580366 |
| ENSG00000135048 | *TMEM2* | 2.442323076 | 0.003308491 |
| ENSG00000169991 | *IFFO2* | 2.431022873 | 0.016080513 |
| ENSG00000112559 | *MDFI* | 2.43069689 | 0.027135681 |
| ENSG00000145247 | *OCIAD2* | 2.423268176 | 0.001426776 |
| ENSG00000163629 | *PTPN13* | 2.422750846 | 0.011047805 |
| ENSG00000162757 | *C1orf74* | 2.422151725 | 0.021940417 |
| ENSG00000103485 | *QPRT* | 2.419473826 | 0.001652092 |
| ENSG00000169129 | *AFAP1L2* | 2.418590447 | 0.000776712 |
| ENSG00000070882 | *OSBPL3* | 2.418326842 | 0.036235051 |
| ENSG00000092470 | *WDR76* | 2.415154191 | 0.002680171 |
| ENSG00000176890 | *TYMS* | 2.41462925 | 0.000571315 |
| ENSG00000181467 | *RAP2B* | 2.409363606 | 0.000258019 |
| ENSG00000156463 | *SH3RF2* | 2.405570424 | 0.030562518 |
| ENSG00000131242 | *RAB11FIP4* | 2.404294056 | 0.00858392 |
| ENSG00000108582 | *CPD* | 2.404061352 | 0.000789838 |
| ENSG00000167900 | *TK1* | 2.398580923 | 0.004299914 |
| ENSG00000163393 | *SLC22A15* | 2.396337438 | 0.024916667 |
| ENSG00000170522 | *ELOVL6* | 2.396076884 | 0.00164238 |
| ENSG00000213190 | *MLLT11* | 2.39307126 | 0.00173181 |
| ENSG00000182795 | *C1orf116* | 2.388287989 | 0.048182838 |
| ENSG00000112972 | *HMGCS1* | 2.386387318 | 0.001345501 |
| ENSG00000142657 | *PGD* | 2.385499488 | 0.000139593 |
| ENSG00000169641 | *LUZP1* | 2.385084582 | 0.004532302 |
| ENSG00000153815 | *CMIP* | 2.384721598 | 0.01615584 |
| ENSG00000131188 | *PRR7* | 2.38369247 | 0.048382943 |
| ENSG00000103021 | *CCDC113* | 2.383291598 | 0.001695056 |
| ENSG00000106003 | *LFNG* | 2.381408543 | 0.013403355 |
| ENSG00000170191 | *NANP* | 2.377562044 | 0.003127515 |
| ENSG00000153714 | *LURAP1L* | 2.372925655 | 0.012574081 |
| ENSG00000197566 | *ZNF624* | 2.366247036 | 0.037905361 |
| ENSG00000122966 | *CIT* | 2.361583778 | 0.009580728 |
| ENSG00000139289 | *PHLDA1* | 2.356662602 | 0.003268155 |
| ENSG00000111057 | *KRT18* | 2.348326139 | 0.012909667 |
| ENSG00000144036 | *EXOC6B* | 2.348034178 | 0.000132299 |
| ENSG00000178184 | *PARD6G* | 2.347148235 | 0.000478879 |
| ENSG00000119514 | *GALNT12* | 2.346920014 | 0.002392897 |
| ENSG00000172292 | *CERS6* | 2.344742514 | 0.005476452 |
| ENSG00000131153 | *GINS2* | 2.342639017 | 0.000740827 |
| ENSG00000064115 | *TM7SF3* | 2.339219981 | 0.000927286 |
| ENSG00000125107 | *CNOT1* | 2.335612907 | 0.017494851 |
| ENSG00000140199 | *SLC12A6* | 2.334025282 | 0.027518218 |
| ENSG00000196584 | *XRCC2* | 2.333438813 | 0.041022108 |
| ENSG00000138772 | *ANXA3* | 2.331402649 | 0.012653342 |
| ENSG00000137807 | *KIF23* | 2.330031619 | 0.005798532 |
| ENSG00000180263 | *FGD6* | 2.324215559 | 0.012367699 |
| ENSG00000128487 | *SPECC1* | 2.324160259 | 0.000990076 |
| ENSG00000171056 | *SOX7* | 2.32323422 | 0.044963687 |
| ENSG00000135046 | *ANXA1* | 2.321662548 | 0.000207641 |
| ENSG00000213859 | *KCTD11* | 2.319900758 | 0.002813709 |
| ENSG00000135083 | *CCNJL* | 2.317715518 | 0.01666217 |
| ENSG00000151694 | *ADAM17* | 2.316648546 | 0.001674594 |
| ENSG00000152104 | *PTPN14* | 2.313699102 | 0.023866776 |
| ENSG00000230733 | *AC092171.4* | 2.311748382 | 0.026993797 |
| ENSG00000120437 | *ACAT2* | 2.30539247 | 0.002969728 |
| ENSG00000122641 | *INHBA* | 2.301913878 | 0.043789144 |
| ENSG00000064199 | *SPA17* | 2.298852261 | 0.03071228 |
| ENSG00000147324 | *MFHAS1* | 2.297201021 | 0.000671435 |
| ENSG00000183856 | *IQGAP3* | 2.293672285 | 0.017167304 |
| ENSG00000146386 | *ABRACL* | 2.293392686 | 0.010033274 |
| ENSG00000146918 | *NCAPG2* | 2.290810189 | 0.000145113 |
| ENSG00000187741 | *FANCA* | 2.286874324 | 0.039221037 |
| ENSG00000231365 | *RP11-418J17.1* | 2.286473066 | 0.018776292 |
| ENSG00000102699 | *PARP4* | 2.284466186 | 0.000320615 |
| ENSG00000146263 | *MMS22L* | 2.279817033 | 0.001443169 |
| ENSG00000079459 | *FDFT1* | 2.279003117 | 5.35E-05 |
| ENSG00000099942 | *CRKL* | 2.271429359 | 0.001831954 |
| ENSG00000138594 | *TMOD3* | 2.267965089 | 6.80E-05 |
| ENSG00000129354 | *AP1M2* | 2.266392971 | 0.037304087 |
| ENSG00000078018 | *MAP2* | 2.265436173 | 0.019622216 |
| ENSG00000147231 | *CXorf57* | 2.257569644 | 0.018097679 |
| ENSG00000151632 | *AKR1C2* | 2.250124687 | 0.021702391 |
| ENSG00000186350 | *RXRA* | 2.24974877 | 0.029275069 |
| ENSG00000147044 | *CASK* | 2.248545142 | 0.00012627 |
| ENSG00000091317 | *CMTM6* | 2.248365481 | 5.03E-05 |
| ENSG00000127423 | *AUNIP* | 2.248186674 | 0.048246323 |
| ENSG00000072682 | *P4HA2* | 2.247793624 | 0.000827536 |
| ENSG00000185963 | *BICD2* | 2.246660734 | 0.001051102 |
| ENSG00000119969 | *HELLS* | 2.242774987 | 0.006527319 |
| ENSG00000114529 | *C3orf52* | 2.238724678 | 0.009173755 |
| ENSG00000116661 | *FBXO2* | 2.235920588 | 0.007857685 |
| ENSG00000104356 | *POP1* | 2.234784969 | 0.004978482 |
| ENSG00000164976 | *KIAA1161* | 2.233265427 | 0.008475277 |
| ENSG00000177485 | *ZBTB33* | 2.231571855 | 0.002145198 |
| ENSG00000149554 | *CHEK1* | 2.227869275 | 0.00278429 |
| ENSG00000156675 | *RAB11FIP1* | 2.226113071 | 0.041459535 |
| ENSG00000280527 | *AL031587.1* | 2.225351079 | 0.029620059 |
| ENSG00000074181 | *NOTCH3* | 2.223494493 | 0.030269287 |
| ENSG00000169851 | *PCDH7* | 2.221902071 | 0.000986425 |
| ENSG00000168785 | *TSPAN5* | 2.217387686 | 0.010437984 |
| ENSG00000082701 | *GSK3B* | 2.212535657 | 0.000128686 |
| ENSG00000183943 | *PRKX* | 2.210040238 | 0.024438729 |
| ENSG00000127824 | *TUBA4A* | 2.20688538 | 0.012001769 |
| ENSG00000107185 | *RGP1* | 2.206525107 | 0.017492482 |
| ENSG00000102034 | *ELF4* | 2.205554897 | 0.030068642 |
| ENSG00000169583 | *CLIC3* | 2.205290993 | 0.049603858 |
| ENSG00000277443 | *MARCKS* | 2.204988959 | 0.005774413 |
| ENSG00000156711 | *MAPK13* | 2.202755273 | 0.027579041 |
| ENSG00000112655 | *PTK7* | 2.197953595 | 0.012682845 |
| ENSG00000130164 | *LDLR* | 2.196686531 | 0.000149961 |
| ENSG00000198087 | *CD2AP* | 2.195667128 | 0.001427788 |
| ENSG00000197535 | *MYO5A* | 2.192152553 | 0.001321976 |
| ENSG00000066084 | *DIP2B* | 2.189526121 | 0.001520974 |
| ENSG00000159182 | *PRAC1* | 2.181717767 | 0.00252686 |
| ENSG00000146648 | *EGFR* | 2.179330705 | 0.045816156 |
| ENSG00000165650 | *PDZD8* | 2.177119917 | 0.002569706 |
| ENSG00000129235 | *TXNDC17* | 2.177074081 | 0.000892528 |
| ENSG00000085377 | *PREP* | 2.175065401 | 0.000458573 |
| ENSG00000166949 | *SMAD3* | 2.173609309 | 0.002504506 |
| ENSG00000184117 | *NIPSNAP1* | 2.172945379 | 0.000678489 |
| ENSG00000133026 | *MYH10* | 2.171412619 | 0.008726575 |
| ENSG00000160877 | *NACC1* | 2.168370921 | 0.044123301 |
| ENSG00000104723 | *TUSC3* | 2.168361966 | 0.000506413 |
| ENSG00000149573 | *MPZL2* | 2.167242989 | 0.012472987 |
| ENSG00000213186 | *TRIM59* | 2.166332175 | 0.014846717 |
| ENSG00000162909 | *CAPN2* | 2.16564359 | 5.87E-05 |
| ENSG00000123213 | *NLN* | 2.16497869 | 0.010234074 |
| ENSG00000196743 | *GM2A* | 2.162582124 | 5.65E-05 |
| ENSG00000142945 | *KIF2C* | 2.16184979 | 0.01442025 |
| ENSG00000065923 | *SLC9A7* | 2.161332427 | 0.033163365 |
| ENSG00000077152 | *UBE2T* | 2.158046373 | 0.029764178 |
| ENSG00000253729 | *PRKDC* | 2.156485551 | 0.00695386 |
| ENSG00000060558 | *GNA15* | 2.14964907 | 0.028375143 |
| ENSG00000100364 | *KIAA0930* | 2.148425804 | 0.007752032 |
| ENSG00000103647 | *CORO2B* | 2.142144288 | 0.017912387 |
| ENSG00000173156 | *RHOD* | 2.142023311 | 0.000781575 |
| ENSG00000184992 | *BRI3BP* | 2.140898056 | 0.006618463 |
| ENSG00000106853 | *PTGR1* | 2.140264059 | 0.003741094 |
| ENSG00000278843 | *MMP28* | 2.136567881 | 0.049704831 |
| ENSG00000176148 | *TCP11L1* | 2.132678795 | 0.010811746 |
| ENSG00000214944 | *ARHGEF28* | 2.120263625 | 0.019803295 |
| ENSG00000125834 | *STK35* | 2.111054053 | 0.030544769 |
| ENSG00000176170 | *SPHK1* | 2.104503217 | 0.045365719 |
| ENSG00000099204 | *ABLIM1* | 2.103387318 | 0.000150139 |
| ENSG00000164649 | *CDCA7L* | 2.096095519 | 0.041738803 |
| ENSG00000139174 | *PRICKLE1* | 2.095271356 | 0.033043631 |
| ENSG00000180198 | *RCC1* | 2.094084243 | 0.00291985 |
| ENSG00000185567 | *AHNAK2* | 2.092592151 | 0.00655762 |
| ENSG00000051596 | *THOC3* | 2.0920409 | 0.005947161 |
| ENSG00000144136 | *SLC20A1* | 2.091133345 | 0.001690302 |
| ENSG00000163472 | *TMEM79* | 2.08444663 | 0.042108441 |
| ENSG00000120889 | *TNFRSF10B* | 2.079621986 | 0.005516132 |
| ENSG00000118971 | *CCND2* | 2.075974506 | 0.009889561 |
| ENSG00000131473 | *ACLY* | 2.075910217 | 0.002422011 |
| ENSG00000128578 | *STRIP2* | 2.072575192 | 0.010958254 |
| ENSG00000163507 | *KIAA1524* | 2.072240825 | 0.015051248 |
| ENSG00000148426 | *PROSER2* | 2.072149843 | 0.003771744 |
| ENSG00000002834 | *LASP1* | 2.071202811 | 0.035874604 |
| ENSG00000175970 | *UNC119B* | 2.070636046 | 0.005716143 |
| ENSG00000182199 | *SHMT2* | 2.070185511 | 0.003661299 |
| ENSG00000103966 | *EHD4* | 2.069699624 | 0.001021179 |
| ENSG00000058804 | *NDC1* | 2.06957068 | 0.0025232 |
| ENSG00000071054 | *MAP4K4* | 2.066512749 | 0.000813654 |
| ENSG00000160352 | *ZNF714* | 2.065854814 | 0.029407705 |
| ENSG00000100526 | *CDKN3* | 2.062398249 | 0.028626227 |
| ENSG00000132405 | *TBC1D14* | 2.062264667 | 0.029867471 |
| ENSG00000088387 | *DOCK9* | 2.061307002 | 0.006928956 |
| ENSG00000156110 | *ADK* | 2.058754479 | 0.000775035 |
| ENSG00000145246 | *ATP10D* | 2.058003151 | 0.01456526 |
| ENSG00000130751 | *NPAS1* | 2.056029295 | 0.041517866 |
| ENSG00000109452 | *INPP4B* | 2.054677228 | 0.006720302 |
| ENSG00000174951 | *FUT1* | 2.049466381 | 0.045426205 |
| ENSG00000265972 | *TXNIP* | 2.047887038 | 0.018747712 |
| ENSG00000279010 | *RP5-1039K5.19* | 2.047649195 | 0.0129397 |
| ENSG00000112893 | *MAN2A1* | 2.045455332 | 0.005219602 |
| ENSG00000047365 | *ARAP2* | 2.045014224 | 0.043871817 |
| ENSG00000198146 | *ZNF770* | 2.039477431 | 0.01979817 |
| ENSG00000197956 | *S100A6* | 2.036633375 | 0.028505908 |
| ENSG00000152332 | *UHMK1* | 2.027683166 | 0.004225497 |
| ENSG00000157978 | *LDLRAP1* | 2.026723787 | 0.021252392 |
| ENSG00000069011 | *PITX1* | 2.022463636 | 0.004255785 |
| ENSG00000100994 | *PYGB* | 2.022215878 | 0.005650692 |
| ENSG00000135540 | *NHSL1* | 2.020098337 | 0.041153492 |
| ENSG00000176208 | *ATAD5* | 2.017677488 | 0.034341711 |
| ENSG00000121060 | *TRIM25* | 2.015664923 | 0.0359869 |
| ENSG00000173530 | *TNFRSF10D* | 2.014982727 | 0.009199265 |
| ENSG00000090020 | *SLC9A1* | 2.012676163 | 0.036380792 |
| ENSG00000164045 | *CDC25A* | 2.010628319 | 0.006981394 |
| ENSG00000094916 | *CBX5* | 1.999776041 | 0.008866472 |
| ENSG00000167642 | *SPINT2* | 1.99665931 | 0.027429903 |
| ENSG00000137216 | *TMEM63B* | 1.990172876 | 0.03974137 |
| ENSG00000097021 | *ACOT7* | 1.98526223 | 0.006603319 |
| ENSG00000080819 | *CPOX* | 1.984356445 | 0.005275423 |
| ENSG00000161013 | *MGAT4B* | 1.982400447 | 0.026864952 |
| ENSG00000101412 | *E2F1* | 1.981749996 | 0.00892767 |
| ENSG00000167325 | *RRM1* | 1.981324665 | 0.004407842 |
| ENSG00000166106 | *ADAMTS15* | 1.977029832 | 0.049830636 |
| ENSG00000189159 | *HN1* | 1.974305768 | 0.009052119 |
| ENSG00000111602 | *TIMELESS* | 1.973504485 | 0.016077303 |
| ENSG00000012232 | *EXTL3* | 1.967960134 | 0.003886821 |
| ENSG00000101773 | *RBBP8* | 1.967944138 | 0.001282616 |
| ENSG00000137563 | *GGH* | 1.967450301 | 0.004527672 |
| ENSG00000180530 | *NRIP1* | 1.965005429 | 0.017203914 |
| ENSG00000150054 | *MPP7* | 1.964772014 | 0.020860294 |
| ENSG00000010278 | *CD9* | 1.961011431 | 0.000307007 |
| ENSG00000051128 | *HOMER3* | 1.960601728 | 0.016557146 |
| ENSG00000151090 | *THRB* | 1.959005598 | 0.018253835 |
| ENSG00000160007 | *ARHGAP35* | 1.956555305 | 0.034298488 |
| ENSG00000143179 | *UCK2* | 1.955400756 | 0.011496179 |
| ENSG00000123983 | *ACSL3* | 1.954403998 | 0.005179037 |
| ENSG00000110921 | *MVK* | 1.952670174 | 0.003603634 |
| ENSG00000198648 | *STK39* | 1.952105959 | 0.012602577 |
| ENSG00000010292 | *NCAPD2* | 1.949085917 | 0.030413468 |
| ENSG00000102572 | *STK24* | 1.948642205 | 0.001270573 |
| ENSG00000133119 | *RFC3* | 1.946922675 | 0.025869004 |
| ENSG00000182481 | *KPNA2* | 1.944636922 | 0.019200673 |
| ENSG00000175216 | *CKAP5* | 1.94357804 | 0.003789428 |
| ENSG00000181938 | *GINS3* | 1.940688274 | 0.023300891 |
| ENSG00000130147 | *SH3BP4* | 1.94044786 | 0.01939493 |
| ENSG00000114738 | *MAPKAPK3* | 1.940432976 | 0.000732552 |
| ENSG00000167553 | *TUBA1C* | 1.938341161 | 0.048911089 |
| ENSG00000182606 | *TRAK1* | 1.936343074 | 0.03951258 |
| ENSG00000140575 | *IQGAP1* | 1.936118056 | 0.00165783 |
| ENSG00000206053 | *HN1L* | 1.93519624 | 0.011037819 |
| ENSG00000138413 | *IDH1* | 1.935032694 | 0.002247359 |
| ENSG00000133138 | *TBC1D8B* | 1.933057261 | 0.029501258 |
| ENSG00000127589 | *TUBBP1* | 1.932179722 | 0.036151579 |
| ENSG00000177189 | *RPS6KA3* | 1.926340712 | 0.000451081 |
| ENSG00000107815 | *C10orf2* | 1.920010635 | 0.047324771 |
| ENSG00000095002 | *MSH2* | 1.919079932 | 0.003023168 |
| ENSG00000125266 | *EFNB2* | 1.915181529 | 0.002376107 |
| ENSG00000119314 | *PTBP3* | 1.912850417 | 0.000818963 |
| ENSG00000186493 | *C5orf38* | 1.911694512 | 0.031130496 |
| ENSG00000159228 | *CBR1* | 1.910727227 | 0.002732454 |
| ENSG00000174442 | *ZWILCH* | 1.910120169 | 0.007874871 |
| ENSG00000122483 | *CCDC18* | 1.909767928 | 0.040397674 |
| ENSG00000181827 | *RFX7* | 1.905506633 | 0.017309575 |
| ENSG00000113810 | *SMC4* | 1.905457837 | 0.00532789 |
| ENSG00000162702 | *ZNF281* | 1.904261156 | 0.004115967 |
| ENSG00000096063 | *SRPK1* | 1.901703699 | 0.001259038 |
| ENSG00000123836 | *PFKFB2* | 1.899583106 | 0.029567669 |
| ENSG00000117632 | *STMN1* | 1.898282762 | 0.03037462 |
| ENSG00000033178 | *UBA6* | 1.891651921 | 0.002085468 |
| ENSG00000146733 | *PSPH* | 1.890993133 | 0.007068393 |
| ENSG00000168003 | *SLC3A2* | 1.8904525 | 0.020701077 |
| ENSG00000119979 | *FAM45A* | 1.890093556 | 0.001964803 |
| ENSG00000280798 | *LINC00294* | 1.88848759 | 0.009930597 |
| ENSG00000069956 | *MAPK6* | 1.887067863 | 0.017589979 |
| ENSG00000100441 | *KHNYN* | 1.88696925 | 0.045426205 |
| ENSG00000174307 | *PHLDA3* | 1.886835492 | 0.004520639 |
| ENSG00000182134 | *TDRKH* | 1.885360975 | 0.036318025 |
| ENSG00000133816 | *MICAL2* | 1.883679039 | 0.039035806 |
| ENSG00000084112 | *SSH1* | 1.882845553 | 0.042934627 |
| ENSG00000162783 | *IER5* | 1.882486806 | 0.000904903 |
| ENSG00000118200 | *CAMSAP2* | 1.879430016 | 0.016576506 |
| ENSG00000104689 | *TNFRSF10A* | 1.872229709 | 0.015038707 |
| ENSG00000092621 | *PHGDH* | 1.869212104 | 0.006867044 |
| ENSG00000078399 | *HOXA9* | 1.867690273 | 0.01979817 |
| ENSG00000132321 | *IQCA1* | 1.865984245 | 0.046436132 |
| ENSG00000157985 | *AGAP1* | 1.8639009 | 0.042836544 |
| ENSG00000175643 | *RMI2* | 1.858574382 | 0.017746002 |
| ENSG00000128050 | *PAICS* | 1.858545068 | 0.002256268 |
| ENSG00000166130 | *IKBIP* | 1.8522934 | 0.032926251 |
| ENSG00000186575 | *NF2* | 1.851988053 | 0.010291292 |
| ENSG00000187091 | *PLCD1* | 1.847473243 | 0.042644417 |
| ENSG00000147459 | *DOCK5* | 1.847434252 | 0.014473542 |
| ENSG00000065357 | *DGKA* | 1.840719714 | 0.039671127 |
| ENSG00000120278 | *PLEKHG1* | 1.834881254 | 0.02331518 |
| ENSG00000151746 | *BICD1* | 1.834593561 | 0.044070473 |
| ENSG00000088812 | *ATRN* | 1.829947888 | 0.031128765 |
| ENSG00000172167 | *MTBP* | 1.82949768 | 0.048155794 |
| ENSG00000168496 | *FEN1* | 1.828696184 | 0.037614866 |
| ENSG00000131504 | *DIAPH1* | 1.828643 | 0.024438729 |
| ENSG00000013016 | *EHD3* | 1.82578108 | 0.042042555 |
| ENSG00000247626 | *MARS2* | 1.825625438 | 0.02795003 |
| ENSG00000196233 | *LCOR* | 1.824413178 | 0.024702949 |
| ENSG00000144354 | *CDCA7* | 1.824400428 | 0.006162577 |
| ENSG00000165097 | *KDM1B* | 1.824243155 | 0.010776412 |
| ENSG00000075539 | *FRYL* | 1.82192936 | 0.020557355 |
| ENSG00000107581 | *EIF3A* | 1.821923131 | 0.017956935 |
| ENSG00000166025 | *AMOTL1* | 1.819504925 | 0.012491835 |
| ENSG00000119782 | *FKBP1B* | 1.818237674 | 0.03647551 |
| ENSG00000122952 | *ZWINT* | 1.817701004 | 0.015715739 |
| ENSG00000131462 | *TUBG1* | 1.817370344 | 0.01022482 |
| ENSG00000115464 | *USP34* | 1.817274142 | 0.041549271 |
| ENSG00000163249 | *CCNYL1* | 1.817072022 | 0.006107214 |
| ENSG00000067225 | *PKM* | 1.816951901 | 0.00149755 |
| ENSG00000170779 | *CDCA4* | 1.816772103 | 0.018209062 |
| ENSG00000124145 | *SDC4* | 1.812146767 | 0.003606464 |
| ENSG00000132256 | *TRIM5* | 1.811184072 | 0.007941926 |
| ENSG00000176105 | *YES1* | 1.810726559 | 0.009019256 |
| ENSG00000152749 | *GPR180* | 1.808734864 | 0.008768089 |
| ENSG00000162129 | *CLPB* | 1.806527535 | 0.04318149 |
| ENSG00000196914 | *ARHGEF12* | 1.80583215 | 0.027579041 |
| ENSG00000198369 | *SPRED2* | 1.804533176 | 0.038362148 |
| ENSG00000170017 | *ALCAM* | 1.801736692 | 0.007075978 |
| ENSG00000196972 | *SMIM10L2B* | 1.801471385 | 0.034435184 |
| ENSG00000079691 | *LRRC16A* | 1.798903487 | 0.002440712 |
| ENSG00000197971 | *MBP* | 1.798532057 | 0.030892861 |
| ENSG00000181588 | *MEX3D* | 1.793846395 | 0.046315108 |
| ENSG00000108219 | *TSPAN14* | 1.793563091 | 0.035375534 |
| ENSG00000075151 | *EIF4G3* | 1.790442528 | 0.001105304 |
| ENSG00000135823 | *STX6* | 1.790427134 | 0.002440712 |
| ENSG00000011028 | *MRC2* | 1.7881612 | 0.042960121 |
| ENSG00000105926 | *MPP6* | 1.787693775 | 0.040399864 |
| ENSG00000179859 | *AC025335.1* | 1.78320694 | 0.027797637 |
| ENSG00000166908 | *PIP4K2C* | 1.781416613 | 0.013423961 |
| ENSG00000142192 | *APP* | 1.780946824 | 0.004876409 |
| ENSG00000157227 | *MMP14* | 1.779731193 | 0.036499701 |
| ENSG00000170571 | *EMB* | 1.779426621 | 0.022787041 |
| ENSG00000147874 | *HAUS6* | 1.77671526 | 0.016449112 |
| ENSG00000113300 | *CNOT6* | 1.775869578 | 0.00343317 |
| ENSG00000177565 | *TBL1XR1* | 1.775023558 | 0.00271537 |
| ENSG00000109079 | *TNFAIP1* | 1.774495357 | 0.012540111 |
| ENSG00000198934 | *MAGEE1* | 1.769263513 | 0.026186209 |
| ENSG00000186591 | *UBE2H* | 1.767350174 | 0.003749589 |
| ENSG00000087253 | *LPCAT2* | 1.767179123 | 0.010611571 |
| ENSG00000075391 | *RASAL2* | 1.76710629 | 0.019852402 |
| ENSG00000076248 | *UNG* | 1.766853209 | 0.014251958 |
| ENSG00000000460 | *C1orf112* | 1.766735863 | 0.037905426 |
| ENSG00000179833 | *SERTAD2* | 1.766343241 | 0.002966727 |
| ENSG00000127870 | *RNF6* | 1.76623383 | 0.008126119 |
| ENSG00000103811 | *CTSH* | 1.765499898 | 0.022171534 |
| ENSG00000140598 | *EFTUD1* | 1.763101657 | 0.002646232 |
| ENSG00000110841 | *PPFIBP1* | 1.761833702 | 0.025535775 |
| ENSG00000180667 | *YOD1* | 1.760447369 | 0.048907505 |
| ENSG00000153214 | *TMEM87B* | 1.759678069 | 0.005780095 |
| ENSG00000176454 | *LPCAT4* | 1.756461994 | 0.030824679 |
| ENSG00000158623 | *COPG2* | 1.755321425 | 0.003923777 |
| ENSG00000115295 | *CLIP4* | 1.753113595 | 0.019075058 |
| ENSG00000164904 | *ALDH7A1* | 1.752906996 | 0.00837779 |
| ENSG00000084092 | *NOA1* | 1.751295642 | 0.004867874 |
| ENSG00000170745 | *KCNS3* | 1.750779632 | 0.037440813 |
| ENSG00000134109 | *EDEM1* | 1.74917747 | 0.017015294 |
| ENSG00000118263 | *KLF7* | 1.747847891 | 0.023290337 |
| ENSG00000167693 | *NXN* | 1.746018647 | 0.017114265 |
| ENSG00000100522 | *GNPNAT1* | 1.745301673 | 0.049447783 |
| ENSG00000270696 | *RP11-342K6.1* | 1.744706736 | 0.035636873 |
| ENSG00000128872 | *TMOD2* | 1.74448698 | 0.036099342 |
| ENSG00000112118 | *MCM3* | 1.74393641 | 0.020748534 |
| ENSG00000174151 | *CYB561D1* | 1.742672125 | 0.017956935 |
| ENSG00000116704 | *SLC35D1* | 1.741296737 | 0.045801439 |
| ENSG00000196937 | *FAM3C* | 1.740799241 | 0.009872421 |
| ENSG00000146242 | *TPBG* | 1.740088706 | 0.005621385 |
| ENSG00000128829 | *EIF2AK4* | 1.732956362 | 0.00769501 |
| ENSG00000163558 | *PRKCI* | 1.732402642 | 0.02946722 |
| ENSG00000089902 | *RCOR1* | 1.730386386 | 0.012665775 |
| ENSG00000170113 | *NIPA1* | 1.730110861 | 0.011166515 |
| ENSG00000168040 | *FADD* | 1.72811134 | 0.04257413 |
| ENSG00000089682 | *RBM41* | 1.727844749 | 0.044256434 |
| ENSG00000197102 | *DYNC1H1* | 1.727815143 | 0.020968945 |
| ENSG00000164402 | *8-Sep* | 1.727230478 | 0.03779776 |
| ENSG00000196141 | *SPATS2L* | 1.725777176 | 0.011637692 |
| ENSG00000106692 | *FKTN* | 1.724140235 | 0.033639635 |
| ENSG00000186716 | *BCR* | 1.72285763 | 0.037637792 |
| ENSG00000106537 | *TSPAN13* | 1.713520957 | 0.049175182 |
| ENSG00000072274 | *TFRC* | 1.711838796 | 0.03503583 |
| ENSG00000113407 | *TARS* | 1.711571609 | 0.013936062 |
| ENSG00000164951 | *PDP1* | 1.708018018 | 0.012367699 |
| ENSG00000102024 | *PLS3* | 1.705040961 | 0.032445604 |
| ENSG00000176624 | *MEX3C* | 1.70468169 | 0.009489349 |
| ENSG00000138758 | *11-Sep* | 1.698601609 | 0.006494793 |
| ENSG00000075711 | *DLG1* | 1.696522156 | 0.008016156 |
| ENSG00000167447 | *SMG8* | 1.691872414 | 0.028106176 |
| ENSG00000005020 | *SKAP2* | 1.690592888 | 0.021713607 |
| ENSG00000198363 | *ASPH* | 1.689856867 | 0.009559051 |
| ENSG00000136485 | *DCAF7* | 1.687616922 | 0.029298471 |
| ENSG00000099810 | *MTAP* | 1.68004047 | 0.005917503 |
| ENSG00000187446 | *CHP1* | 1.679518567 | 0.002408965 |
| ENSG00000139926 | *FRMD6* | 1.677129996 | 0.022691702 |
| ENSG00000142867 | *BCL10* | 1.676849147 | 0.022762091 |
| ENSG00000233901 | *LINC01503* | 1.675353539 | 0.043698551 |
| ENSG00000179029 | *TMEM107* | 1.675326891 | 0.019600527 |
| ENSG00000066777 | *ARFGEF1* | 1.668134735 | 0.02735928 |
| ENSG00000196712 | *NF1* | 1.667112648 | 0.02995102 |
| ENSG00000145545 | *SRD5A1* | 1.666647884 | 0.022691702 |
| ENSG00000042445 | *RETSAT* | 1.665938205 | 0.024248136 |
| ENSG00000165732 | *DDX21* | 1.665627138 | 0.014570886 |
| ENSG00000105755 | *ETHE1* | 1.662232858 | 0.016833636 |
| ENSG00000111247 | *RAD51AP1* | 1.657280512 | 0.047142995 |
| ENSG00000106780 | *MEGF9* | 1.655480563 | 0.016200762 |
| ENSG00000111144 | *LTA4H* | 1.655292981 | 0.023842688 |
| ENSG00000156531 | *PHF6* | 1.654614019 | 0.047564558 |
| ENSG00000160691 | *SHC1* | 1.653880382 | 0.038871915 |
| ENSG00000057294 | *PKP2* | 1.649239165 | 0.015014755 |
| ENSG00000259956 | *RBM15B* | 1.649100061 | 0.039442422 |
| ENSG00000134247 | *PTGFRN* | 1.644992187 | 0.030160102 |
| ENSG00000101935 | *AMMECR1* | 1.644972733 | 0.024671588 |
| ENSG00000196943 | *NOP9* | 1.644358832 | 0.043471681 |
| ENSG00000198176 | *TFDP1* | 1.644175896 | 0.005006678 |
| ENSG00000167306 | *MYO5B* | 1.640858021 | 0.02849365 |
| ENSG00000084234 | *APLP2* | 1.640217034 | 0.006987632 |
| ENSG00000167291 | *TBC1D16* | 1.636496367 | 0.047595549 |
| ENSG00000001167 | *NFYA* | 1.634233307 | 0.015362993 |
| ENSG00000112249 | *ASCC3* | 1.632556304 | 0.031488915 |
| ENSG00000134294 | *SLC38A2* | 1.630080796 | 0.040399864 |
| ENSG00000253368 | *TRNP1* | 1.629789195 | 0.017368836 |
| ENSG00000058063 | *ATP11B* | 1.626633027 | 0.023571561 |
| ENSG00000085449 | *WDFY1* | 1.624372138 | 0.02190351 |
| ENSG00000133818 | *RRAS2* | 1.623663166 | 0.048248896 |
| ENSG00000035115 | *SH3YL1* | 1.622199035 | 0.035490629 |
| ENSG00000133657 | *ATP13A3* | 1.619453589 | 0.035398899 |
| ENSG00000120539 | *MASTL* | 1.619300621 | 0.047496594 |
| ENSG00000009335 | *UBE3C* | 1.617320947 | 0.014676695 |
| ENSG00000107789 | *MINPP1* | 1.616556018 | 0.008343825 |
| ENSG00000196072 | *BLOC1S2* | 1.615619703 | 0.02665938 |
| ENSG00000173848 | *NET1* | 1.61130508 | 0.042042555 |
| ENSG00000156299 | *TIAM1* | 1.609667663 | 0.02799086 |
| ENSG00000069020 | *MAST4* | 1.609313351 | 0.049522985 |
| ENSG00000160752 | *FDPS* | 1.608785679 | 0.018938066 |
| ENSG00000075651 | *PLD1* | 1.604634877 | 0.015245238 |
| ENSG00000166471 | *TMEM41B* | 1.599242453 | 0.030774731 |
| ENSG00000137269 | *LRRC1* | 1.597638186 | 0.019420299 |
| ENSG00000007202 | *KIAA0100* | 1.592725709 | 0.017203914 |
| ENSG00000198420 | *TCAF1* | 1.59075979 | 0.043300948 |
| ENSG00000147155 | *EBP* | 1.589795781 | 0.018518542 |
| ENSG00000115762 | *PLEKHB2* | 1.585814575 | 0.008749519 |
| ENSG00000124486 | *USP9X* | 1.580947398 | 0.017039832 |
| ENSG00000124207 | *CSE1L* | 1.576330892 | 0.021702495 |
| ENSG00000111817 | *DSE* | 1.575284464 | 0.021568283 |
| ENSG00000170027 | *YWHAG* | 1.564405061 | 0.015330831 |
| ENSG00000102225 | *CDK16* | 1.562697102 | 0.035375534 |
| ENSG00000166189 | *HPS6* | 1.56255664 | 0.047879044 |
| ENSG00000169398 | *PTK2* | 1.561981789 | 0.011635753 |
| ENSG00000132646 | *PCNA* | 1.561504435 | 0.014829627 |
| ENSG00000100714 | *MTHFD1* | 1.559270984 | 0.019416936 |
| ENSG00000124120 | *TTPAL* | 1.559032164 | 0.029055887 |
| ENSG00000159055 | *MIS18A* | 1.555764724 | 0.041544801 |
| ENSG00000101266 | *CSNK2A1* | 1.555266708 | 0.005836884 |
| ENSG00000197930 | *ERO1L* | 1.554119778 | 0.015392404 |
| ENSG00000158435 | *CNOT11* | 1.551708338 | 0.010160553 |
| ENSG00000135862 | *LAMC1* | 1.551006251 | 0.0437603 |
| ENSG00000136824 | *SMC2* | 1.550488971 | 0.043790972 |
| ENSG00000006634 | *DBF4* | 1.550390657 | 0.048045901 |
| ENSG00000110660 | *SLC35F2* | 1.549645009 | 0.028957854 |
| ENSG00000070756 | *PABPC1* | 1.548083526 | 0.006162577 |
| ENSG00000117528 | *ABCD3* | 1.54769966 | 0.034119538 |
| ENSG00000145907 | *G3BP1* | 1.547134614 | 0.007185563 |
| ENSG00000181191 | *PJA1* | 1.544914802 | 0.033965492 |
| ENSG00000110092 | *CCND1* | 1.544674573 | 0.026352657 |
| ENSG00000138698 | *RAP1GDS1* | 1.5445396 | 0.035059998 |
| ENSG00000141429 | *GALNT1* | 1.54136491 | 0.019727166 |
| ENSG00000067704 | *IARS2* | 1.539807695 | 0.008468228 |
| ENSG00000114573 | *ATP6V1A* | 1.539803345 | 0.013508366 |
| ENSG00000081760 | *AACS* | 1.537158829 | 0.027178135 |
| ENSG00000106367 | *AP1S1* | 1.535896265 | 0.0157754 |
| ENSG00000138604 | *GLCE* | 1.533979945 | 0.025569331 |
| ENSG00000163755 | *HPS3* | 1.531742333 | 0.047287615 |
| ENSG00000132842 | *AP3B1* | 1.531265078 | 0.013811431 |
| ENSG00000117308 | *GALE* | 1.523903614 | 0.043790972 |
| ENSG00000027001 | *MIPEP* | 1.521788803 | 0.027797637 |
| ENSG00000110013 | *SIAE* | 1.521268517 | 0.024657149 |
| ENSG00000188706 | *ZDHHC9* | 1.517829021 | 0.046336957 |
| ENSG00000119541 | *VPS4B* | 1.516774568 | 0.023303663 |
| ENSG00000123352 | *SPATS2* | 1.514913282 | 0.049056739 |
| ENSG00000166477 | *LEO1* | 1.511014187 | 0.016450056 |
| ENSG00000104889 | *RNASEH2A* | 1.510166867 | 0.047461567 |
| ENSG00000101639 | *CEP192* | 1.500897947 | 0.043802611 |
| ENSG00000158195 | *WASF2* | 1.500564382 | 0.037440813 |
| ENSG00000124762 | *CDKN1A* | 1.499968282 | 0.008906325 |
| ENSG00000125686 | *MED1* | 1.493873792 | 0.040554305 |
| ENSG00000083093 | *PALB2* | 1.492232591 | 0.02982024 |
| ENSG00000100596 | *SPTLC2* | 1.492180192 | 0.015906748 |
| ENSG00000182197 | *EXT1* | 1.492006182 | 0.029030709 |
| ENSG00000179562 | *GCC1* | 1.488488124 | 0.042992661 |
| ENSG00000130559 | *CAMSAP1* | 1.484528545 | 0.031804988 |
| ENSG00000117139 | *KDM5B* | 1.479827783 | 0.034051392 |
| ENSG00000164597 | *COG5* | 1.477029177 | 0.020162719 |
| ENSG00000151292 | *CSNK1G3* | 1.476045796 | 0.042108441 |
| ENSG00000176393 | *RNPEP* | 1.474680115 | 0.024844204 |
| ENSG00000078747 | *ITCH* | 1.472491438 | 0.049326623 |
| ENSG00000136261 | *BZW2* | 1.471764593 | 0.018950527 |
| ENSG00000107854 | *TNKS2* | 1.47086738 | 0.024598293 |
| ENSG00000163597 | *SNHG16* | 1.469400489 | 0.038783111 |
| ENSG00000107566 | *ERLIN1* | 1.467581211 | 0.030900685 |
| ENSG00000115652 | *UXS1* | 1.467579865 | 0.030302056 |
| ENSG00000189060 | *H1F0* | 1.466755949 | 0.046470983 |
| ENSG00000025800 | *KPNA6* | 1.46541683 | 0.042836544 |
| ENSG00000139644 | *TMBIM6* | 1.457424707 | 0.010863674 |
| ENSG00000099250 | *NRP1* | 1.454122456 | 0.037546091 |
| ENSG00000159792 | *PSKH1* | 1.451508207 | 0.021739325 |
| ENSG00000160551 | *TAOK1* | 1.451448133 | 0.038645127 |
| ENSG00000137845 | *ADAM10* | 1.447945807 | 0.023622783 |
| ENSG00000141458 | *NPC1* | 1.447330027 | 0.029193053 |
| ENSG00000213853 | *EMP2* | 1.446831454 | 0.040068877 |
| ENSG00000138801 | *PAPSS1* | 1.445234048 | 0.04293124 |
| ENSG00000104687 | *GSR* | 1.44437747 | 0.038584061 |
| ENSG00000170606 | *HSPA4* | 1.441406458 | 0.021193185 |
| ENSG00000136159 | *NUDT15* | 1.437669309 | 0.033548804 |
| ENSG00000100503 | *NIN* | 1.43720796 | 0.015828613 |
| ENSG00000188352 | *FOCAD* | 1.436927106 | 0.028522034 |
| ENSG00000148110 | *HIATL1* | 1.435691224 | 0.016450056 |
| ENSG00000167658 | *EEF2* | 1.434910955 | 0.045951653 |
| ENSG00000164024 | *METAP1* | 1.429875299 | 0.025146271 |
| ENSG00000136628 | *EPRS* | 1.428569224 | 0.015504039 |
| ENSG00000111412 | *C12orf49* | 1.427652083 | 0.026429418 |
| ENSG00000116641 | *DOCK7* | 1.426455315 | 0.03951258 |
| ENSG00000162923 | *WDR26* | 1.419917964 | 0.017693955 |
| ENSG00000165609 | *NUDT5* | 1.419664176 | 0.024546591 |
| ENSG00000164924 | *YWHAZ* | 1.419445012 | 0.045680497 |
| ENSG00000005893 | *LAMP2* | 1.417509676 | 0.049913485 |
| ENSG00000176853 | *FAM91A1* | 1.416191678 | 0.047324771 |
| ENSG00000153113 | *CAST* | 1.415019415 | 0.026186209 |
| ENSG00000244509 | *APOBEC3C* | 1.412719463 | 0.036099342 |
| ENSG00000150403 | *TMCO3* | 1.41232416 | 0.035899905 |
| ENSG00000114354 | *TFG* | 1.408526137 | 0.026669765 |
| ENSG00000047849 | *MAP4* | 1.40737709 | 0.034731191 |
| ENSG00000134684 | *YARS* | 1.401806109 | 0.035059998 |
| ENSG00000106829 | *TLE4* | 1.401533296 | 0.04075514 |
| ENSG00000116489 | *CAPZA1* | 1.393369039 | 0.02841128 |
| ENSG00000063046 | *EIF4B* | 1.38928726 | 0.048911089 |
| ENSG00000108424 | *KPNB1* | 1.387360241 | 0.026437297 |
| ENSG00000170385 | *SLC30A1* | 1.376211765 | 0.043946465 |
| ENSG00000154473 | *BUB3* | 1.361517773 | 0.034416306 |
| ENSG00000161249 | *DMKN* | 1.35906883 | 0.047142995 |
| ENSG00000170873 | *MTSS1* | 1.358987274 | 0.034132398 |
| ENSG00000150753 | *CCT5* | 1.35139871 | 0.041494883 |
| ENSG00000198730 | *CTR9* | 1.348409194 | 0.034275243 |
| ENSG00000047315 | *POLR2B* | 1.344802157 | 0.020464267 |
| ENSG00000029364 | *SLC39A9* | 1.343618991 | 0.0277681 |
| ENSG00000090054 | *SPTLC1* | 1.342765287 | 0.042733348 |
| ENSG00000135250 | *SRPK2* | 1.338172435 | 0.035490629 |
| ENSG00000108828 | *VAT1* | 1.327169801 | 0.037609217 |
| ENSG00000070770 | *CSNK2A2* | 1.32387239 | 0.024502415 |
| ENSG00000196305 | *IARS* | 1.323458514 | 0.0333146 |
| ENSG00000184575 | *XPOT* | 1.319163841 | 0.048246323 |
| ENSG00000115053 | *NCL* | 1.300550874 | 0.030391513 |
| ENSG00000017797 | *RALBP1* | 1.298466649 | 0.030786119 |
| ENSG00000135387 | *CAPRIN1* | 1.298344629 | 0.034416306 |
| ENSG00000139687 | *RB1* | 1.295065874 | 0.045365719 |
| ENSG00000101193 | *GID8* | 1.292426577 | 0.032445604 |
| ENSG00000170035 | *UBE2E3* | 1.273160697 | 0.047595549 |
| ENSG00000044115 | *CTNNA1* | 1.268894555 | 0.030683999 |
| ENSG00000166197 | *NOLC1* | 1.261975548 | 0.049256406 |
| ENSG00000116237 | *ICMT* | 1.261265822 | 0.034291382 |
| ENSG00000103591 | *AAGAB* | 1.261052321 | 0.039752629 |
| ENSG00000184432 | *COPB2* | 1.182557801 | 0.04812124 |
| ENSG00000109046 | *WSB1* | -1.342112961 | 0.033163365 |
| ENSG00000146278 | *PNRC1* | -1.348794701 | 0.044502101 |
| ENSG00000104679 | *R3HCC1* | -1.353757047 | 0.046198085 |
| ENSG00000132275 | *RRP8* | -1.369482895 | 0.037609217 |
| ENSG00000204859 | *ZBTB48* | -1.38258583 | 0.048776777 |
| ENSG00000068024 | *HDAC4* | -1.384960803 | 0.040645293 |
| ENSG00000117616 | *RSRP1* | -1.394993044 | 0.046788924 |
| ENSG00000084676 | *NCOA1* | -1.406615141 | 0.030524517 |
| ENSG00000139636 | *LMBR1L* | -1.407734774 | 0.033754079 |
| ENSG00000051108 | *HERPUD1* | -1.41450645 | 0.04313083 |
| ENSG00000183955 | *SETD8* | -1.415890179 | 0.034416306 |
| ENSG00000116001 | *TIA1* | -1.443852384 | 0.027147543 |
| ENSG00000003756 | *RBM5* | -1.455017317 | 0.014644452 |
| ENSG00000173757 | *STAT5B* | -1.459170819 | 0.025757234 |
| ENSG00000117360 | *PRPF3* | -1.459532334 | 0.013670321 |
| ENSG00000196998 | *WDR45* | -1.470185868 | 0.04554172 |
| ENSG00000174652 | *ZNF266* | -1.471067469 | 0.015955718 |
| ENSG00000182208 | *MOB2* | -1.476530025 | 0.027219854 |
| ENSG00000099622 | *CIRBP* | -1.485722727 | 0.030208975 |
| ENSG00000157036 | *EXOG* | -1.488006944 | 0.047600319 |
| ENSG00000132635 | *PCED1A* | -1.499591061 | 0.029515877 |
| ENSG00000160948 | *VPS28* | -1.51459343 | 0.030824679 |
| ENSG00000246705 | *H2AFJ* | -1.522744065 | 0.041792396 |
| ENSG00000143224 | *PPOX* | -1.526530437 | 0.028626227 |
| ENSG00000124588 | *NQO2* | -1.527174043 | 0.026021377 |
| ENSG00000160908 | *ZNF394* | -1.527482131 | 0.032926251 |
| ENSG00000198105 | *ZNF248* | -1.535270213 | 0.041532491 |
| ENSG00000166313 | *APBB1* | -1.535720687 | 0.035522985 |
| ENSG00000173281 | *PPP1R3B* | -1.538789799 | 0.049056302 |
| ENSG00000120708 | *TGFBI* | -1.539606385 | 0.046797587 |
| ENSG00000215252 | *GOLGA8B* | -1.54275079 | 0.043686746 |
| ENSG00000162852 | *CNST* | -1.544060229 | 0.034731191 |
| ENSG00000148399 | *DPH7* | -1.548809392 | 0.040509976 |
| ENSG00000174886 | *NDUFA11* | -1.54885222 | 0.029136128 |
| ENSG00000148814 | *LRRC27* | -1.551462949 | 0.032135347 |
| ENSG00000130813 | *C19orf66* | -1.554687746 | 0.046390477 |
| ENSG00000101400 | *SNTA1* | -1.559399524 | 0.035859204 |
| ENSG00000145012 | *LPP* | -1.559438794 | 0.017651466 |
| ENSG00000169733 | *RFNG* | -1.574135807 | 0.04123658 |
| ENSG00000212907 | *MT-ND4L* | -1.576812855 | 0.034977233 |
| ENSG00000137871 | *ZNF280D* | -1.577379369 | 0.026669765 |
| ENSG00000273015 | *RP11-352M15.2* | -1.582275141 | 0.049681246 |
| ENSG00000228253 | *MT-ATP8* | -1.585615683 | 0.023432358 |
| ENSG00000176476 | *CCDC101* | -1.600156447 | 0.024916667 |
| ENSG00000166311 | *SMPD1* | -1.602652948 | 0.015853008 |
| ENSG00000163430 | *FSTL1* | -1.607169715 | 0.034993618 |
| ENSG00000117640 | *MTFR1L* | -1.609986106 | 0.032016143 |
| ENSG00000185404 | *SP140L* | -1.610536067 | 0.036131241 |
| ENSG00000170581 | *STAT2* | -1.613829489 | 0.048014971 |
| ENSG00000232859 | *LYRM9* | -1.614281363 | 0.036099342 |
| ENSG00000100342 | *APOL1* | -1.615526825 | 0.039220224 |
| ENSG00000108848 | *LUC7L3* | -1.620090377 | 0.035966893 |
| ENSG00000172922 | *RNASEH2C* | -1.621806725 | 0.046824776 |
| ENSG00000102804 | *TSC22D1* | -1.629086985 | 0.009440427 |
| ENSG00000110811 | *P3H3* | -1.634314407 | 0.014357204 |
| ENSG00000088448 | *ANKRD10* | -1.638732142 | 0.010328997 |
| ENSG00000168826 | *ZBTB49* | -1.639105978 | 0.034709068 |
| ENSG00000133624 | *ZNF767P* | -1.643342363 | 0.045119352 |
| ENSG00000119673 | *ACOT2* | -1.64628307 | 0.04844158 |
| ENSG00000132906 | *CASP9* | -1.649291852 | 0.040245233 |
| ENSG00000105518 | *TMEM205* | -1.65535 | 0.030618923 |
| ENSG00000064995 | *TAF11* | -1.656275331 | 0.042733348 |
| ENSG00000173482 | *PTPRM* | -1.657066424 | 0.040653387 |
| ENSG00000139410 | *SDSL* | -1.662056436 | 0.047142995 |
| ENSG00000144647 | *POMGNT2* | -1.669524606 | 0.048623075 |
| ENSG00000125652 | *ALKBH7* | -1.673897614 | 0.040674613 |
| ENSG00000135916 | *ITM2C* | -1.681601846 | 0.024253287 |
| ENSG00000198873 | *GRK5* | -1.684372714 | 0.032446243 |
| ENSG00000074657 | *ZNF532* | -1.688692155 | 0.032356596 |
| ENSG00000113269 | *RNF130* | -1.691431676 | 0.019084378 |
| ENSG00000125967 | *NECAB3* | -1.693672319 | 0.014503078 |
| ENSG00000126351 | *THRA* | -1.694178819 | 0.00769501 |
| ENSG00000102030 | *NAA10* | -1.695106044 | 0.039210836 |
| ENSG00000148120 | *C9orf3* | -1.695389875 | 0.008546997 |
| ENSG00000026297 | *RNASET2* | -1.702670277 | 0.005804088 |
| ENSG00000171163 | *ZNF692* | -1.706442644 | 0.030786119 |
| ENSG00000172366 | *FAM195A* | -1.709440275 | 0.009031057 |
| ENSG00000103381 | *CPPED1* | -1.710628467 | 0.035252027 |
| ENSG00000158716 | *DUSP23* | -1.712460544 | 0.025374452 |
| ENSG00000164039 | *BDH2* | -1.712872115 | 0.045441076 |
| ENSG00000111752 | *PHC1* | -1.713982583 | 0.04172679 |
| ENSG00000128335 | *APOL2* | -1.716274653 | 0.020028357 |
| ENSG00000075234 | *TTC38* | -1.720227596 | 0.00769501 |
| ENSG00000162542 | *TMCO4* | -1.72762329 | 0.035358724 |
| ENSG00000183718 | *TRIM52* | -1.727961709 | 0.021838293 |
| ENSG00000169592 | *INO80E* | -1.729865442 | 0.025146271 |
| ENSG00000141994 | *DUS3L* | -1.732171515 | 0.03363803 |
| ENSG00000175309 | *PHYKPL* | -1.732829238 | 0.028522034 |
| ENSG00000180628 | *PCGF5* | -1.734125629 | 0.042864907 |
| ENSG00000072195 | *SPEG* | -1.734406336 | 0.032053696 |
| ENSG00000108861 | *DUSP3* | -1.737052327 | 0.039892693 |
| ENSG00000102362 | *SYTL4* | -1.73862449 | 0.033195248 |
| ENSG00000186205 | *1-Mar* | -1.741916557 | 0.02726073 |
| ENSG00000257621 | *PSMA3-AS1* | -1.747034129 | 0.011687679 |
| ENSG00000204054 | *LINC00963* | -1.751473054 | 0.012995034 |
| ENSG00000270055 | *CTD-3092A11.2* | -1.751507733 | 0.020800531 |
| ENSG00000164308 | *ERAP2* | -1.756334189 | 0.01311033 |
| ENSG00000198556 | *ZNF789* | -1.757542193 | 0.008106865 |
| ENSG00000187239 | *FNBP1* | -1.7575571 | 0.00674207 |
| ENSG00000104907 | *TRMT1* | -1.758021891 | 0.008281827 |
| ENSG00000133104 | *SPG20* | -1.76188141 | 0.03951258 |
| ENSG00000090661 | *CERS4* | -1.764162012 | 0.029679852 |
| ENSG00000105393 | *BABAM1* | -1.764474692 | 0.031077309 |
| ENSG00000079819 | *EPB41L2* | -1.764778638 | 0.010197481 |
| ENSG00000167528 | *ZNF641* | -1.766584016 | 0.006057381 |
| ENSG00000257337 | *RP11-983P16.4* | -1.767348676 | 0.011777581 |
| ENSG00000150977 | *RILPL2* | -1.767790148 | 0.008265674 |
| ENSG00000101439 | *CST3* | -1.772340184 | 0.013726877 |
| ENSG00000104957 | *CCDC130* | -1.786963491 | 0.013327629 |
| ENSG00000243335 | *KCTD7* | -1.787606231 | 0.031888368 |
| ENSG00000198478 | *SH3BGRL2* | -1.787830403 | 0.0176816 |
| ENSG00000124074 | *ENKD1* | -1.793696163 | 0.012001769 |
| ENSG00000179743 | *FLJ37453* | -1.795575444 | 0.045739997 |
| ENSG00000158717 | *RNF166* | -1.798566419 | 0.02979206 |
| ENSG00000132323 | *ILKAP* | -1.801298609 | 0.00769501 |
| ENSG00000265491 | *RNF115* | -1.801778714 | 0.042408077 |
| ENSG00000163297 | *ANTXR2* | -1.804015592 | 0.02795003 |
| ENSG00000136514 | *RTP4* | -1.808128953 | 0.048929298 |
| ENSG00000154359 | *LONRF1* | -1.809704912 | 0.043946465 |
| ENSG00000113231 | *PDE8B* | -1.812984652 | 0.049575919 |
| ENSG00000162817 | *C1orf115* | -1.814273271 | 0.040708144 |
| ENSG00000173992 | *CCS* | -1.821993941 | 0.006536503 |
| ENSG00000071073 | *MGAT4A* | -1.825459854 | 0.024842293 |
| ENSG00000099785 | *2-Mar* | -1.829904162 | 0.034831911 |
| ENSG00000184481 | *FOXO4* | -1.831519358 | 0.018278603 |
| ENSG00000168765 | *GSTM4* | -1.832292543 | 0.028991393 |
| ENSG00000124006 | *OBSL1* | -1.832348658 | 0.028859832 |
| ENSG00000258056 | *RP11-644F5.11* | -1.834876771 | 0.032368451 |
| ENSG00000168096 | *ANKS3* | -1.837955638 | 0.008918677 |
| ENSG00000156804 | *FBXO32* | -1.840289564 | 0.005973739 |
| ENSG00000175611 | *LINC00476* | -1.842711873 | 0.048782519 |
| ENSG00000102760 | *RGCC* | -1.843985535 | 0.027439426 |
| ENSG00000158773 | *USF1* | -1.844219571 | 0.010406049 |
| ENSG00000120832 | *MTERF2* | -1.845186247 | 0.021293797 |
| ENSG00000116337 | *AMPD2* | -1.847753703 | 0.039220224 |
| ENSG00000164056 | *SPRY1* | -1.850811037 | 0.041178364 |
| ENSG00000153721 | *CNKSR3* | -1.856554869 | 0.03331402 |
| ENSG00000110237 | *ARHGEF17* | -1.869247243 | 0.030824679 |
| ENSG00000074416 | *MGLL* | -1.872441863 | 0.014532847 |
| ENSG00000185986 | *SDHAP3* | -1.882435349 | 0.038372154 |
| ENSG00000144445 | *KANSL1L* | -1.890943748 | 0.03171893 |
| ENSG00000243943 | *ZNF512* | -1.894387219 | 0.003923777 |
| ENSG00000148339 | *SLC25A25* | -1.897882275 | 0.048744374 |
| ENSG00000142694 | *EVA1B* | -1.898606259 | 0.008396396 |
| ENSG00000198483 | *ANKRD35* | -1.905214273 | 0.043686746 |
| ENSG00000167543 | *TP53I13* | -1.905757726 | 0.032926251 |
| ENSG00000103024 | *NME3* | -1.910427717 | 0.007599303 |
| ENSG00000101849 | *TBL1X* | -1.912031235 | 0.004978482 |
| ENSG00000215375 | *MYL5* | -1.916750784 | 0.008598939 |
| ENSG00000007392 | *LUC7L* | -1.921742372 | 0.002285438 |
| ENSG00000180354 | *MTURN* | -1.922081687 | 0.021294346 |
| ENSG00000114395 | *CYB561D2* | -1.922967182 | 0.032749865 |
| ENSG00000108773 | *KAT2A* | -1.92374172 | 0.004978482 |
| ENSG00000053501 | *USE1* | -1.929773448 | 0.011387726 |
| ENSG00000147526 | *TACC1* | -1.930223722 | 0.004965407 |
| ENSG00000197062 | *ZSCAN26* | -1.942150495 | 0.004543705 |
| ENSG00000132773 | *TOE1* | -1.942155987 | 0.009038076 |
| ENSG00000170271 | *FAXDC2* | -1.946168369 | 0.003900634 |
| ENSG00000160961 | *ZNF333* | -1.946272112 | 0.002123251 |
| ENSG00000152582 | *SPEF2* | -1.949684384 | 0.036182918 |
| ENSG00000280787 | *HERC2P2* | -1.951110934 | 0.049497511 |
| ENSG00000151729 | *SLC25A4* | -1.954681899 | 0.013747862 |
| ENSG00000075826 | *SEC31B* | -1.956509605 | 0.042917505 |
| ENSG00000156398 | *SFXN2* | -1.958218663 | 0.032020953 |
| ENSG00000162066 | *AMDHD2* | -1.964981528 | 0.025757234 |
| ENSG00000136603 | *SKIL* | -1.965455841 | 0.014355915 |
| ENSG00000143013 | *LMO4* | -1.965760774 | 0.020757019 |
| ENSG00000104205 | *SGK3* | -1.966665368 | 0.013884545 |
| ENSG00000196352 | *CD55* | -1.967156497 | 0.028185644 |
| ENSG00000119711 | *ALDH6A1* | -1.96820461 | 0.009223535 |
| ENSG00000188878 | *FBF1* | -1.970545563 | 0.044123301 |
| ENSG00000141933 | *TPGS1* | -1.975654905 | 0.022608925 |
| ENSG00000211459 | *MT-RNR1* | -1.979196785 | 0.042733348 |
| ENSG00000198886 | *MT-ND4* | -1.981219047 | 0.004867022 |
| ENSG00000149177 | *PTPRJ* | -1.984642044 | 0.025910149 |
| ENSG00000148288 | *GBGT1* | -1.99678579 | 0.031077309 |
| ENSG00000172878 | *METAP1D* | -1.998629982 | 0.014779752 |
| ENSG00000120913 | *PDLIM2* | -2.001302653 | 0.038946044 |
| ENSG00000103202 | *NME4* | -2.002472346 | 0.008629868 |
| ENSG00000178922 | *HYI* | -2.004858718 | 0.02538654 |
| ENSG00000144843 | *ADPRH* | -2.009509533 | 0.043756038 |
| ENSG00000113621 | *TXNDC15* | -2.01110025 | 0.006200762 |
| ENSG00000087086 | *FTL* | -2.011347763 | 0.01160498 |
| ENSG00000112096 | *SOD2* | -2.013784433 | 0.007405395 |
| ENSG00000166839 | *ANKDD1A* | -2.018536675 | 0.047564558 |
| ENSG00000196378 | *ZNF34* | -2.01905377 | 0.019116129 |
| ENSG00000076555 | *ACACB* | -2.020305891 | 0.026602815 |
| ENSG00000171105 | *INSR* | -2.021632424 | 0.024694459 |
| ENSG00000138166 | *DUSP5* | -2.027197617 | 0.032749865 |
| ENSG00000114626 | *ABTB1* | -2.027300178 | 0.008926778 |
| ENSG00000167363 | *FN3K* | -2.028123094 | 0.016282327 |
| ENSG00000183617 | *MRPL54* | -2.03224337 | 0.022808598 |
| ENSG00000076706 | *MCAM* | -2.034077682 | 0.026151655 |
| ENSG00000198736 | *MSRB1* | -2.036268048 | 0.019244872 |
| ENSG00000136098 | *NEK3* | -2.037444371 | 0.017309129 |
| ENSG00000010219 | *DYRK4* | -2.039154625 | 0.031229672 |
| ENSG00000139192 | *TAPBPL* | -2.039911742 | 0.028814583 |
| ENSG00000119900 | *OGFRL1* | -2.045367756 | 0.041064445 |
| ENSG00000067191 | *CACNB1* | -2.049553435 | 0.021848259 |
| ENSG00000169740 | *ZNF32* | -2.052443354 | 0.025971135 |
| ENSG00000161544 | *CYGB* | -2.052938198 | 0.01761793 |
| ENSG00000169087 | *HSPBAP1* | -2.054203828 | 0.025913214 |
| ENSG00000083814 | *ZNF671* | -2.055619686 | 0.016674772 |
| ENSG00000148175 | *STOM* | -2.05600078 | 0.006957525 |
| ENSG00000129116 | *PALLD* | -2.057647161 | 0.042108441 |
| ENSG00000215039 | *CD27-AS1* | -2.058848198 | 0.001591948 |
| ENSG00000169604 | *ANTXR1* | -2.060250832 | 0.008774243 |
| ENSG00000237886 | *LINC01573* | -2.064224587 | 0.028074527 |
| ENSG00000167397 | *VKORC1* | -2.065603152 | 0.044638071 |
| ENSG00000139631 | *CSAD* | -2.066774901 | 0.030579016 |
| ENSG00000138821 | *SLC39A8* | -2.068063092 | 0.045907731 |
| ENSG00000134352 | *IL6ST* | -2.070872276 | 0.031455866 |
| ENSG00000152117 | *AC093838.4* | -2.07158007 | 0.035939364 |
| ENSG00000154114 | *TBCEL* | -2.075449518 | 0.032749865 |
| ENSG00000198157 | *HMGN5* | -2.075847757 | 0.031804988 |
| ENSG00000184371 | *CSF1* | -2.07673928 | 0.04172679 |
| ENSG00000267796 | *LIN37* | -2.080290322 | 0.030208975 |
| ENSG00000115526 | *CHST10* | -2.086801378 | 0.01890137 |
| ENSG00000224660 | *SH3BP5-AS1* | -2.0885212 | 0.043946465 |
| ENSG00000109501 | *WFS1* | -2.092562107 | 0.000653719 |
| ENSG00000205763 | *RP9P* | -2.093920138 | 0.020812318 |
| ENSG00000145022 | *TCTA* | -2.094014198 | 0.001056354 |
| ENSG00000185596 | *WASH3P* | -2.094905702 | 0.007475568 |
| ENSG00000175395 | *ZNF25* | -2.096544919 | 0.010648454 |
| ENSG00000184584 | *TMEM173* | -2.101216978 | 0.019586513 |
| ENSG00000105483 | *CARD8* | -2.103558195 | 0.005101066 |
| ENSG00000198899 | *MT-ATP6* | -2.104382612 | 0.002287432 |
| ENSG00000153933 | *DGKE* | -2.105013408 | 0.006060648 |
| ENSG00000170364 | *SETMAR* | -2.109080904 | 0.016499435 |
| ENSG00000169696 | *ASPSCR1* | -2.113043478 | 0.003947192 |
| ENSG00000164674 | *SYTL3* | -2.113913457 | 0.002459121 |
| ENSG00000169249 | *ZRSR2* | -2.116159966 | 0.005826526 |
| ENSG00000004534 | *RBM6* | -2.116766029 | 0.000806574 |
| ENSG00000079337 | *RAPGEF3* | -2.120454944 | 0.001813654 |
| ENSG00000135469 | *COQ10A* | -2.120738787 | 0.007270097 |
| ENSG00000215440 | *NPEPL1* | -2.121906924 | 0.0142063 |
| ENSG00000188677 | *PARVB* | -2.124128074 | 0.026896834 |
| ENSG00000182179 | *UBA7* | -2.125159207 | 0.017343961 |
| ENSG00000146067 | *FAM193B* | -2.125451909 | 0.013970222 |
| ENSG00000277147 | *LINC00869* | -2.125626567 | 0.024522908 |
| ENSG00000092094 | *OSGEP* | -2.127915745 | 0.002783334 |
| ENSG00000173511 | *VEGFB* | -2.128354857 | 0.003274535 |
| ENSG00000176978 | *DPP7* | -2.129140477 | 0.020107676 |
| ENSG00000104870 | *FCGRT* | -2.130228183 | 0.000334289 |
| ENSG00000257704 | *INAFM1* | -2.131123483 | 0.001199325 |
| ENSG00000117625 | *RCOR3* | -2.131153985 | 0.001328654 |
| ENSG00000102901 | *CENPT* | -2.131738256 | 0.000824514 |
| ENSG00000163803 | *PLB1* | -2.132094739 | 0.04640606 |
| ENSG00000013441 | *CLK1* | -2.136218027 | 0.011885422 |
| ENSG00000102897 | *LYRM1* | -2.136274435 | 0.025463672 |
| ENSG00000186088 | *GSAP* | -2.136717422 | 0.014234141 |
| ENSG00000108465 | *CDK5RAP3* | -2.137652622 | 0.011108945 |
| ENSG00000183049 | *CAMK1D* | -2.144110471 | 0.041863949 |
| ENSG00000153208 | *MERTK* | -2.145062484 | 0.0186403 |
| ENSG00000259295 | *CSPG4P12* | -2.147260661 | 0.042934627 |
| ENSG00000163754 | *GYG1* | -2.148136397 | 0.020557355 |
| ENSG00000008128 | *CDK11A* | -2.150646419 | 0.033536102 |
| ENSG00000214174 | *AMZ2P1* | -2.150942285 | 0.03363593 |
| ENSG00000054983 | *GALC* | -2.154000596 | 0.004123551 |
| ENSG00000139508 | *SLC46A3* | -2.154490195 | 0.010300083 |
| ENSG00000111885 | *MAN1A1* | -2.154605082 | 0.031312621 |
| ENSG00000164125 | *FAM198B* | -2.158008122 | 0.005973447 |
| ENSG00000162409 | *PRKAA2* | -2.158517747 | 0.031282016 |
| ENSG00000140398 | *NEIL1* | -2.159422133 | 0.027518218 |
| ENSG00000176293 | *ZNF135* | -2.16259225 | 0.009187217 |
| ENSG00000143344 | *RGL1* | -2.165072557 | 0.004896396 |
| ENSG00000112530 | *PACRG* | -2.168758253 | 0.036433079 |
| ENSG00000225791 | *TRAM2-AS1* | -2.170260597 | 0.035636873 |
| ENSG00000145020 | *AMT* | -2.171442489 | 0.010423687 |
| ENSG00000271601 | *LIX1L* | -2.172599065 | 0.01442025 |
| ENSG00000198933 | *TBKBP1* | -2.17715363 | 0.028867044 |
| ENSG00000058056 | *USP13* | -2.180437515 | 0.002761424 |
| ENSG00000172638 | *EFEMP2* | -2.180668989 | 0.000409517 |
| ENSG00000069667 | *RORA* | -2.183284233 | 0.020884002 |
| ENSG00000116691 | *MIIP* | -2.189390528 | 0.005254242 |
| ENSG00000232931 | *LINC00342* | -2.193471467 | 0.013062144 |
| ENSG00000113971 | *NPHP3* | -2.196230243 | 0.002625943 |
| ENSG00000213339 | *QTRT1* | -2.198547323 | 0.000436048 |
| ENSG00000185022 | *MAFF* | -2.199864206 | 0.045213236 |
| ENSG00000162341 | *TPCN2* | -2.202913791 | 0.005573495 |
| ENSG00000177409 | *SAMD9L* | -2.204358124 | 0.013636715 |
| ENSG00000178764 | *ZHX2* | -2.205868703 | 0.000702052 |
| ENSG00000148362 | *C9orf142* | -2.213844329 | 0.000808171 |
| ENSG00000225663 | *FAM195B* | -2.214864749 | 0.008087993 |
| ENSG00000182484 | *WASH6P* | -2.215972496 | 0.001027151 |
| ENSG00000163637 | *PRICKLE2* | -2.217425658 | 0.011187014 |
| ENSG00000125868 | *DSTN* | -2.219505768 | 0.019089852 |
| ENSG00000158062 | *UBXN11* | -2.220961947 | 0.001427949 |
| ENSG00000082014 | *SMARCD3* | -2.225798146 | 0.000128618 |
| ENSG00000198939 | *ZFP2* | -2.231652775 | 0.037708883 |
| ENSG00000274565 | *CTD-3035K23.7* | -2.23600811 | 0.020017307 |
| ENSG00000148057 | *IDNK* | -2.236789455 | 0.049392207 |
| ENSG00000136040 | *PLXNC1* | -2.237480011 | 0.034810488 |
| ENSG00000163681 | *SLMAP* | -2.23874207 | 0.04556486 |
| ENSG00000248527 | *MTATP6P1* | -2.239123813 | 0.001042806 |
| ENSG00000065989 | *PDE4A* | -2.242911728 | 0.030208975 |
| ENSG00000112759 | *SLC29A1* | -2.243166793 | 0.005338361 |
| ENSG00000104863 | *LIN7B* | -2.245052817 | 0.004677657 |
| ENSG00000176700 | *SCAND2P* | -2.245501013 | 0.01926514 |
| ENSG00000204219 | *TCEA3* | -2.247205152 | 0.01614382 |
| ENSG00000205220 | *PSMB10* | -2.24845441 | 0.020701077 |
| ENSG00000101443 | *WFDC2* | -2.250210155 | 0.036147871 |
| ENSG00000100399 | *CHADL* | -2.251516847 | 0.026377208 |
| ENSG00000146556 | *WASH2P* | -2.252670571 | 0.000494582 |
| ENSG00000069493 | *CLEC2D* | -2.262083842 | 0.018730055 |
| ENSG00000154240 | *CEP112* | -2.262413686 | 0.022391658 |
| ENSG00000123096 | *SSPN* | -2.263875704 | 0.008502174 |
| ENSG00000196814 | *MVB12B* | -2.264102365 | 0.016061873 |
| ENSG00000070495 | *JMJD6* | -2.266126566 | 0.000412976 |
| ENSG00000231889 | *TRAF3IP2-AS1* | -2.267541641 | 0.031760845 |
| ENSG00000119950 | *MXI1* | -2.269171525 | 0.010397643 |
| ENSG00000115594 | *IL1R1* | -2.27243782 | 0.000272906 |
| ENSG00000250722 | *SEPP1* | -2.279008657 | 0.017711011 |
| ENSG00000142089 | *IFITM3* | -2.28206731 | 0.002557831 |
| ENSG00000135842 | *FAM129A* | -2.283278449 | 0.006600385 |
| ENSG00000120675 | *DNAJC15* | -2.283922082 | 0.040488538 |
| ENSG00000125503 | *PPP1R12C* | -2.285842287 | 0.002238316 |
| ENSG00000267519 | *CTD-3252C9.4* | -2.287237888 | 0.002300454 |
| ENSG00000210151 | *MT-TS1* | -2.288483129 | 0.04123951 |
| ENSG00000185745 | *IFIT1* | -2.292179943 | 0.024778296 |
| ENSG00000247746 | *USP51* | -2.295201572 | 0.048350208 |
| ENSG00000179818 | *PCBP1-AS1* | -2.297758976 | 0.000525977 |
| ENSG00000169252 | *ADRB2* | -2.298685985 | 0.018938066 |
| ENSG00000077942 | *FBLN1* | -2.299387959 | 0.020405573 |
| ENSG00000117020 | *AKT3* | -2.300109533 | 0.011145122 |
| ENSG00000111666 | *CHPT1* | -2.30698149 | 0.010153094 |
| ENSG00000119630 | *PGF* | -2.315268062 | 0.019416936 |
| ENSG00000187601 | *MAGEH1* | -2.319793801 | 0.009803272 |
| ENSG00000074211 | *PPP2R2C* | -2.320251035 | 0.040577993 |
| ENSG00000166770 | *ZNF667-AS1* | -2.322155413 | 0.004553568 |
| ENSG00000272341 | *RP1-151F17.2* | -2.323680381 | 0.047856872 |
| ENSG00000134253 | *TRIM45* | -2.328074551 | 0.001591839 |
| ENSG00000111801 | *BTN3A3* | -2.330524879 | 0.012217507 |
| ENSG00000175265 | *GOLGA8A* | -2.332240581 | 0.010480787 |
| ENSG00000166562 | *SEC11C* | -2.332270837 | 0.001747179 |
| ENSG00000213928 | *IRF9* | -2.33474646 | 0.017272749 |
| ENSG00000214765 | *SEPT7P2* | -2.335843608 | 0.004494632 |
| ENSG00000214309 | *MBLAC1* | -2.336076591 | 0.021201893 |
| ENSG00000186994 | *KANK3* | -2.338505884 | 0.031077309 |
| ENSG00000109738 | *GLRB* | -2.340895259 | 0.039608156 |
| ENSG00000163596 | *ICA1L* | -2.3414029 | 0.000434145 |
| ENSG00000134285 | *FKBP11* | -2.34456928 | 0.021904654 |
| ENSG00000264207 | *RP11-196G18.23* | -2.350963103 | 0.014244033 |
| ENSG00000011422 | *PLAUR* | -2.351786075 | 0.014691525 |
| ENSG00000175183 | *CSRP2* | -2.353197693 | 0.004799174 |
| ENSG00000166974 | *MAPRE2* | -2.354954863 | 0.01681473 |
| ENSG00000172594 | *SMPDL3A* | -2.356564002 | 0.004414784 |
| ENSG00000214226 | *C17orf67* | -2.356711708 | 0.030327998 |
| ENSG00000138074 | *SLC5A6* | -2.360776918 | 0.000454402 |
| ENSG00000065717 | *TLE2* | -2.365046535 | 0.006073355 |
| ENSG00000177706 | *FAM20C* | -2.366488446 | 0.000181552 |
| ENSG00000152784 | *PRDM8* | -2.370181557 | 0.017492482 |
| ENSG00000258199 | *RP11-977G19.5* | -2.372466126 | 0.01609741 |
| ENSG00000212866 | *HSPA1B* | -2.375588196 | 0.028708895 |
| ENSG00000219626 | *FAM228B* | -2.377346164 | 0.002440712 |
| ENSG00000270189 | *RP11-258C19.7* | -2.378142756 | 0.002899083 |
| ENSG00000179965 | *ZNF771* | -2.378371182 | 0.013450422 |
| ENSG00000124104 | *SNX21* | -2.381287726 | 0.000207306 |
| ENSG00000117643 | *MAN1C1* | -2.382157544 | 0.002625084 |
| ENSG00000275400 | *RP4-756H11.5* | -2.389273997 | 0.039220224 |
| ENSG00000182310 | *SPACA6P* | -2.390581939 | 0.000329152 |
| ENSG00000115738 | *ID2* | -2.392170281 | 0.037015554 |
| ENSG00000172197 | *MBOAT1* | -2.392524309 | 0.008424463 |
| ENSG00000078687 | *TNRC6C* | -2.393949061 | 0.000135929 |
| ENSG00000231711 | *LINC00899* | -2.395097029 | 0.011750439 |
| ENSG00000143995 | *MEIS1* | -2.396586615 | 0.001959806 |
| ENSG00000184678 | *HIST2H2BE* | -2.397075095 | 0.0279807 |
| ENSG00000147437 | *GNRH1* | -2.399448613 | 0.041479102 |
| ENSG00000169660 | *HEXDC* | -2.399463033 | 0.002468804 |
| ENSG00000166669 | *ATF7IP2* | -2.400112966 | 0.008491938 |
| ENSG00000141480 | *ARRB2* | -2.401342785 | 0.006405073 |
| ENSG00000163328 | *GPR155* | -2.401697511 | 0.002583527 |
| ENSG00000125347 | *IRF1* | -2.40408041 | 0.040158434 |
| ENSG00000137727 | *ARHGAP20* | -2.404792836 | 0.02735928 |
| ENSG00000162426 | *SLC45A1* | -2.405896483 | 0.00427586 |
| ENSG00000151882 | *CCL28* | -2.407846283 | 0.04668272 |
| ENSG00000174928 | *C3orf33* | -2.410838692 | 0.012677042 |
| ENSG00000120458 | *MSANTD2* | -2.411987711 | 0.001866301 |
| ENSG00000118514 | *ALDH8A1* | -2.414634397 | 0.038635097 |
| ENSG00000223547 | *ZNF844* | -2.416973624 | 0.021037754 |
| ENSG00000123609 | *NMI* | -2.417237255 | 0.003148788 |
| ENSG00000130208 | *APOC1* | -2.417310755 | 0.041447603 |
| ENSG00000238083 | *LRRC37A2* | -2.417324801 | 0.020376776 |
| ENSG00000135124 | *P2RX4* | -2.420041282 | 0.007329817 |
| ENSG00000241769 | *LINC00893* | -2.42191844 | 0.029559288 |
| ENSG00000101333 | *PLCB4* | -2.422947478 | 0.037851923 |
| ENSG00000109339 | *MAPK10* | -2.423254804 | 0.000814551 |
| ENSG00000119686 | *FLVCR2* | -2.42582356 | 0.02388214 |
| ENSG00000104177 | *MYEF2* | -2.427283538 | 0.037761139 |
| ENSG00000136425 | *CIB2* | -2.428786992 | 0.002435566 |
| ENSG00000269858 | *EGLN2* | -2.430184335 | 0.000938067 |
| ENSG00000196440 | *ARMCX4* | -2.431312208 | 0.001376788 |
| ENSG00000112769 | *LAMA4* | -2.439169158 | 0.013702283 |
| ENSG00000153179 | *RASSF3* | -2.439672045 | 0.00291985 |
| ENSG00000185885 | *IFITM1* | -2.442122267 | 0.002629646 |
| ENSG00000088543 | *C3orf18* | -2.443020602 | 0.005870529 |
| ENSG00000210135 | *MT-TN* | -2.448952882 | 0.026244801 |
| ENSG00000177606 | *JUN* | -2.449866928 | 0.036099342 |
| ENSG00000272523 | *LINC01023* | -2.460795646 | 0.019803295 |
| ENSG00000132522 | *GPS2* | -2.461053825 | 0.014516494 |
| ENSG00000068079 | *IFI35* | -2.47092762 | 6.35E-05 |
| ENSG00000149489 | *ROM1* | -2.473341494 | 0.010328997 |
| ENSG00000071205 | *ARHGAP10* | -2.473747414 | 0.00125682 |
| ENSG00000276021 | *WDR81* | -2.474359382 | 0.022602324 |
| ENSG00000143434 | *SEMA6C* | -2.477092382 | 0.023013283 |
| ENSG00000235162 | *C12orf75* | -2.477381914 | 0.039364121 |
| ENSG00000111678 | *C12orf57* | -2.477656796 | 0.002079418 |
| ENSG00000164430 | *MB21D1* | -2.481715454 | 0.023243777 |
| ENSG00000268516 | *CTD-3138B18.5* | -2.483096194 | 0.024502415 |
| ENSG00000141505 | *ASGR1* | -2.486630075 | 0.025971135 |
| ENSG00000183621 | *ZNF438* | -2.487550702 | 0.002026177 |
| ENSG00000155957 | *TMBIM4* | -2.495294212 | 0.002224863 |
| ENSG00000212694 | *LINC01089* | -2.498444309 | 0.013956914 |
| ENSG00000165424 | *ZCCHC24* | -2.49881482 | 8.38E-05 |
| ENSG00000229320 | *KRT8P12* | -2.500429115 | 0.009424224 |
| ENSG00000213366 | *GSTM2* | -2.500621293 | 0.000188766 |
| ENSG00000197256 | *KANK2* | -2.501099567 | 0.000768965 |
| ENSG00000113448 | *PDE4D* | -2.507062287 | 0.000347183 |
| ENSG00000271646 | *RP11-326I11.3* | -2.508146588 | 0.021652561 |
| ENSG00000117569 | *PTBP2* | -2.513267333 | 0.003412382 |
| ENSG00000149328 | *GLB1L2* | -2.515628892 | 0.001494038 |
| ENSG00000205090 | *TMEM240* | -2.516739514 | 0.035252027 |
| ENSG00000130830 | *MPP1* | -2.518073213 | 0.000645624 |
| ENSG00000162645 | *GBP2* | -2.518483908 | 0.001076473 |
| ENSG00000199753 | *SNORD104* | -2.522901212 | 0.014516494 |
| ENSG00000230795 | *HLA-K* | -2.52324648 | 0.032926251 |
| ENSG00000271895 | *RP4-635E18.8* | -2.527187837 | 0.015118699 |
| ENSG00000110455 | *ACCS* | -2.527984172 | 0.004443458 |
| ENSG00000186352 | *ANKRD37* | -2.531236396 | 0.005011845 |
| ENSG00000196405 | *EVL* | -2.531330261 | 0.000734491 |
| ENSG00000157110 | *RBPMS* | -2.536131358 | 3.91E-05 |
| ENSG00000182983 | *ZNF662* | -2.539216502 | 0.000351476 |
| ENSG00000131781 | *FMO5* | -2.539902416 | 0.004568056 |
| ENSG00000018869 | *ZNF582* | -2.545189965 | 0.008111282 |
| ENSG00000122786 | *CALD1* | -2.546915515 | 0.009085787 |
| ENSG00000221869 | *CEBPD* | -2.547031944 | 0.0003911 |
| ENSG00000132182 | *NUP210* | -2.54936207 | 0.030097922 |
| ENSG00000154930 | *ACSS1* | -2.549717342 | 0.004041354 |
| ENSG00000133142 | *TCEAL4* | -2.555188555 | 0.001675778 |
| ENSG00000281404 | *LINC01176* | -2.558685786 | 0.046544969 |
| ENSG00000255198 | *SNHG9* | -2.561054788 | 0.007512008 |
| ENSG00000107968 | *MAP3K8* | -2.561771166 | 0.025154915 |
| ENSG00000177096 | *FAM109B* | -2.565470836 | 0.003745432 |
| ENSG00000031081 | *ARHGAP31* | -2.567175187 | 0.00210569 |
| ENSG00000050438 | *SLC4A8* | -2.569095772 | 0.042118717 |
| ENSG00000159899 | *NPR2* | -2.573008442 | 0.000985218 |
| ENSG00000006740 | *ARHGAP44* | -2.573232461 | 0.027014952 |
| ENSG00000240038 | *AMY2B* | -2.574173163 | 0.005541847 |
| ENSG00000081181 | *ARG2* | -2.575486872 | 0.035829564 |
| ENSG00000114631 | *PODXL2* | -2.582450157 | 0.025136611 |
| ENSG00000260563 | *RP13-516M14.1* | -2.582511766 | 0.002808931 |
| ENSG00000071282 | *LMCD1* | -2.583281335 | 0.004583669 |
| ENSG00000120885 | *CLU* | -2.583615822 | 0.010359071 |
| ENSG00000248124 | *RRN3P1* | -2.587307223 | 0.000823559 |
| ENSG00000163412 | *EIF4E3* | -2.590782416 | 0.004826646 |
| ENSG00000111674 | *ENO2* | -2.590958139 | 3.31E-05 |
| ENSG00000168010 | *ATG16L2* | -2.605234958 | 0.007478768 |
| ENSG00000139597 | *N4BP2L1* | -2.609167414 | 0.000950787 |
| ENSG00000104936 | *DMPK* | -2.610215286 | 0.000671991 |
| ENSG00000182963 | *GJC1* | -2.613972628 | 0.003007731 |
| ENSG00000273247 | *RP11-83A24.2* | -2.619850331 | 0.025757234 |
| ENSG00000141519 | *CCDC40* | -2.620095319 | 0.017114265 |
| ENSG00000198938 | *MT-CO3* | -2.621369088 | 0.000794427 |
| ENSG00000223804 | *CH17-472G23.2* | -2.622563997 | 0.010397643 |
| ENSG00000196507 | *TCEAL3* | -2.624051057 | 0.000371895 |
| ENSG00000210140 | *MT-TC* | -2.629092267 | 0.020431937 |
| ENSG00000225630 | *MTND2P28* | -2.629255751 | 9.58E-05 |
| ENSG00000158747 | *NBL1* | -2.62975941 | 9.87E-05 |
| ENSG00000136052 | *SLC41A2* | -2.634197071 | 0.009471372 |
| ENSG00000274012 | *Metazoa_SRP* | -2.634496025 | 0.015271072 |
| ENSG00000182636 | *NDN* | -2.637554466 | 0.006821456 |
| ENSG00000112186 | *CAP2* | -2.650681171 | 0.01905371 |
| ENSG00000140937 | *CDH11* | -2.651474724 | 0.011463504 |
| ENSG00000105472 | *CLEC11A* | -2.651818801 | 0.002452861 |
| ENSG00000137070 | *IL11RA* | -2.652696911 | 5.90E-05 |
| ENSG00000123080 | *CDKN2C* | -2.652979015 | 0.001409038 |
| ENSG00000273619 | *RP5-908M14.9* | -2.655236993 | 0.030544769 |
| ENSG00000197180 | *CH17-340M24.3* | -2.657361276 | 0.015106938 |
| ENSG00000276728 | *AC142472.6* | -2.658379399 | 0.04572034 |
| ENSG00000164920 | *OSR2* | -2.659779742 | 0.002186972 |
| ENSG00000130775 | *THEMIS2* | -2.66250836 | 0.024771259 |
| ENSG00000212123 | *PRR22* | -2.663637017 | 0.029929873 |
| ENSG00000137672 | *TRPC6* | -2.666791999 | 0.011606603 |
| ENSG00000273329 | *RP11-448A19.1* | -2.670895593 | 0.008005412 |
| ENSG00000198744 | *RP5-857K21.11* | -2.673041939 | 0.002240415 |
| ENSG00000204149 | *AGAP6* | -2.679069251 | 0.00033245 |
| ENSG00000125257 | *ABCC4* | -2.679834572 | 0.021077391 |
| ENSG00000078487 | *ZCWPW1* | -2.681061409 | 0.000294517 |
| ENSG00000162946 | *DISC1* | -2.684648159 | 0.046645966 |
| ENSG00000123146 | *ADGRE5* | -2.688252276 | 0.010811746 |
| ENSG00000184271 | *POU6F1* | -2.693816071 | 0.001043044 |
| ENSG00000183508 | *FAM46C* | -2.694743558 | 0.006579901 |
| ENSG00000150764 | *DIXDC1* | -2.696741123 | 0.003265522 |
| ENSG00000134262 | *AP4B1* | -2.704315126 | 0.001162922 |
| ENSG00000137496 | *IL18BP* | -2.706563445 | 0.009317235 |
| ENSG00000196923 | *PDLIM7* | -2.710019564 | 8.16E-05 |
| ENSG00000206341 | *HLA-H* | -2.712841838 | 0.030408353 |
| ENSG00000174326 | *SLC16A11* | -2.716539051 | 0.027178135 |
| ENSG00000155093 | *PTPRN2* | -2.721743767 | 0.000178993 |
| ENSG00000198727 | *MT-CYB* | -2.728908833 | 8.63E-05 |
| ENSG00000174428 | *GTF2IRD2B* | -2.733586514 | 3.20E-05 |
| ENSG00000138642 | *HERC6* | -2.734043285 | 6.20E-05 |
| ENSG00000279457 | *FO538757.2* | -2.735782688 | 0.000371806 |
| ENSG00000165716 | *FAM69B* | -2.736347554 | 0.003828918 |
| ENSG00000128849 | *CGNL1* | -2.737215205 | 0.000792543 |
| ENSG00000106948 | *AKNA* | -2.737343949 | 0.004576883 |
| ENSG00000150540 | *HNMT* | -2.743700029 | 0.002055067 |
| ENSG00000182287 | *AP1S2* | -2.74380695 | 0.026411733 |
| ENSG00000135324 | *MRAP2* | -2.74446337 | 0.009845542 |
| ENSG00000178498 | *DTX3* | -2.7484886 | 6.71E-05 |
| ENSG00000260549 | *MT1L* | -2.74961803 | 0.003589012 |
| ENSG00000134802 | *SLC43A3* | -2.751546573 | 0.00759088 |
| ENSG00000267100 | *ILF3-AS1* | -2.751794745 | 6.49E-05 |
| ENSG00000166979 | *EVA1C* | -2.75235739 | 2.47E-05 |
| ENSG00000180096 | *1-Sep* | -2.754430125 | 0.038170174 |
| ENSG00000159588 | *CCDC17* | -2.755382344 | 0.0455787 |
| ENSG00000163453 | *IGFBP7* | -2.759421218 | 0.000632008 |
| ENSG00000277701 | *RP11-734K23.9* | -2.760156516 | 0.046750735 |
| ENSG00000165030 | *NFIL3* | -2.762229627 | 0.001530491 |
| ENSG00000196275 | *GTF2IRD2* | -2.764143694 | 0.000631215 |
| ENSG00000100307 | *CBX7* | -2.76434288 | 6.15E-06 |
| ENSG00000137094 | *DNAJB5* | -2.766724621 | 0.01245243 |
| ENSG00000232434 | *C9orf172* | -2.767464954 | 0.039035806 |
| ENSG00000177337 | *DLGAP1-AS1* | -2.771959583 | 0.000932615 |
| ENSG00000185201 | *IFITM2* | -2.775990602 | 0.002720303 |
| ENSG00000272579 | *RP11-101E13.5* | -2.783507153 | 0.019511138 |
| ENSG00000250067 | *YJEFN3* | -2.787512941 | 0.019803295 |
| ENSG00000138185 | *ENTPD1* | -2.796165694 | 0.000123841 |
| ENSG00000142227 | *EMP3* | -2.805953118 | 0.006828637 |
| ENSG00000161551 | *ZNF577* | -2.806847042 | 6.75E-05 |
| ENSG00000198189 | *HSD17B11* | -2.807279912 | 0.000391283 |
| ENSG00000121067 | *SPOP* | -2.808819293 | 0.00345819 |
| ENSG00000234769 | *WASH4P* | -2.808877724 | 0.015238122 |
| ENSG00000100068 | *LRP5L* | -2.813597097 | 0.001641544 |
| ENSG00000165874 | *FAM35BP* | -2.814108382 | 0.012814265 |
| ENSG00000157601 | *MX1* | -2.814268643 | 0.000278525 |
| ENSG00000169083 | *AR* | -2.814286183 | 0.027219854 |
| ENSG00000127507 | *ADGRE2* | -2.815030225 | 0.031208754 |
| ENSG00000081803 | *CADPS2* | -2.820522845 | 0.000101727 |
| ENSG00000146197 | *SCUBE3* | -2.821037986 | 0.004439818 |
| ENSG00000185010 | *F8* | -2.825260717 | 0.00581509 |
| ENSG00000182957 | *SPATA13* | -2.826900608 | 0.00434866 |
| ENSG00000101955 | *SRPX* | -2.826939025 | 0.047287615 |
| ENSG00000121310 | *ECHDC2* | -2.829151546 | 4.31E-05 |
| ENSG00000179094 | *PER1* | -2.834139469 | 0.000888847 |
| ENSG00000182621 | *PLCB1* | -2.834419437 | 0.031965423 |
| ENSG00000196296 | *ATP2A1* | -2.83520137 | 0.023962852 |
| ENSG00000237807 | *RP11-400K9.4* | -2.835825952 | 0.018815845 |
| ENSG00000102879 | *CORO1A* | -2.844455349 | 0.039975677 |
| ENSG00000234518 | *PTGES3P1* | -2.844486596 | 0.035179671 |
| ENSG00000137124 | *ALDH1B1* | -2.847521423 | 0.001980319 |
| ENSG00000244274 | *DBNDD2* | -2.849929229 | 0.000744775 |
| ENSG00000033327 | *GAB2* | -2.852062935 | 0.012991477 |
| ENSG00000113594 | *LIFR* | -2.852188998 | 0.032328185 |
| ENSG00000088538 | *DOCK3* | -2.852267122 | 0.004465896 |
| ENSG00000059378 | *PARP12* | -2.852630049 | 0.001161111 |
| ENSG00000004777 | *ARHGAP33* | -2.856006353 | 0.02394271 |
| ENSG00000277715 | *RP11-651L5.3* | -2.860133444 | 0.038386285 |
| ENSG00000198429 | *ZNF69* | -2.86162876 | 0.004677657 |
| ENSG00000158715 | *SLC45A3* | -2.864113778 | 0.045534252 |
| ENSG00000153064 | *BANK1* | -2.873843304 | 0.04001522 |
| ENSG00000274265 | *CH17-189H20.1* | -2.874575193 | 0.027223338 |
| ENSG00000172458 | *IL17D* | -2.874799812 | 0.010611571 |
| ENSG00000121440 | *PDZRN3* | -2.875859565 | 0.003636655 |
| ENSG00000248487 | *ABHD14A* | -2.879813575 | 0.000291468 |
| ENSG00000137941 | *TTLL7* | -2.880981043 | 0.000748464 |
| ENSG00000163995 | *ABLIM2* | -2.886668693 | 0.009184492 |
| ENSG00000147872 | *PLIN2* | -2.887266044 | 0.001148287 |
| ENSG00000204262 | *COL5A2* | -2.890662032 | 0.004257384 |
| ENSG00000150672 | *DLG2* | -2.891754388 | 0.031760845 |
| ENSG00000260296 | *RP11-395I6.3* | -2.891790549 | 0.044408526 |
| ENSG00000174080 | *CTSF* | -2.894225714 | 0.003250048 |
| ENSG00000100311 | *PDGFB* | -2.896421353 | 0.007486114 |
| ENSG00000180089 | *TMEM86B* | -2.896512577 | 0.028814583 |
| ENSG00000213071 | *LPAL2* | -2.898529813 | 0.046250834 |
| ENSG00000256223 | *ZNF10* | -2.90103027 | 0.001742568 |
| ENSG00000147576 | *ADHFE1* | -2.901222375 | 0.000423848 |
| ENSG00000196739 | *COL27A1* | -2.902629061 | 0.004971579 |
| ENSG00000151778 | *SERP2* | -2.903368056 | 0.029509803 |
| ENSG00000167034 | *NKX3-1* | -2.906227353 | 0.004980239 |
| ENSG00000233608 | *TWIST2* | -2.906274306 | 0.000181333 |
| ENSG00000060762 | *MPC1* | -2.906971907 | 0.001320944 |
| ENSG00000260686 | *CTB-36H16.2* | -2.909756427 | 0.022199305 |
| ENSG00000132718 | *SYT11* | -2.912923244 | 0.002587828 |
| ENSG00000187391 | *MAGI2* | -2.917531585 | 0.004817351 |
| ENSG00000173917 | *HOXB2* | -2.919904965 | 0.008035957 |
| ENSG00000178878 | *APOLD1* | -2.922830532 | 0.023216463 |
| ENSG00000167992 | *VWCE* | -2.926794063 | 0.030842677 |
| ENSG00000269821 | *KCNQ1OT1* | -2.936387477 | 0.00587518 |
| ENSG00000059377 | *TBXAS1* | -2.93658204 | 0.000700242 |
| ENSG00000106560 | *GIMAP2* | -2.936937704 | 0.009132986 |
| ENSG00000223891 | *OSER1-AS1* | -2.938090954 | 0.001346576 |
| ENSG00000183401 | *CCDC159* | -2.938256608 | 7.89E-05 |
| ENSG00000267321 | *RP11-1094M14.11* | -2.94738604 | 0.009440427 |
| ENSG00000260708 | *CTA-29F11.1* | -2.952198245 | 0.00320574 |
| ENSG00000087884 | *AAMDC* | -2.954724846 | 0.009013159 |
| ENSG00000066382 | *MPPED2* | -2.955436006 | 0.000642775 |
| ENSG00000184988 | *TMEM106A* | -2.955886017 | 0.004186027 |
| ENSG00000181444 | *ZNF467* | -2.956048506 | 0.011627906 |
| ENSG00000019485 | *PRDM11* | -2.95793159 | 0.001669709 |
| ENSG00000188732 | *FAM221A* | -2.959365625 | 0.007218076 |
| ENSG00000144857 | *BOC* | -2.960904019 | 0.001034835 |
| ENSG00000255248 | *RP11-166D19.1* | -2.963337547 | 0.03146503 |
| ENSG00000153291 | *SLC25A27* | -2.963554434 | 0.001173155 |
| ENSG00000118849 | *RARRES1* | -2.966748995 | 0.037905361 |
| ENSG00000164136 | *IL15* | -2.966778227 | 0.024205066 |
| ENSG00000161647 | *MPP3* | -2.969493626 | 0.000467121 |
| ENSG00000270074 | *RP11-351I21.11* | -2.971717208 | 0.043946465 |
| ENSG00000017483 | *SLC38A5* | -2.973984267 | 0.004515705 |
| ENSG00000236017 | *ASMTL-AS1* | -2.97788682 | 0.008188432 |
| ENSG00000141068 | *KSR1* | -2.981209924 | 0.02426298 |
| ENSG00000159674 | *SPON2* | -2.981290423 | 0.012906037 |
| ENSG00000168404 | *MLKL* | -2.984059517 | 1.44E-06 |
| ENSG00000168016 | *TRANK1* | -2.989213845 | 0.003740284 |
| ENSG00000259891 | *CTA-204B4.2* | -2.992390261 | 0.004876409 |
| ENSG00000118515 | *SGK1* | -2.993138994 | 0.028013574 |
| ENSG00000280032 | *RP11-832A4.7* | -2.993979544 | 0.03066439 |
| ENSG00000196549 | *MME* | -2.994345358 | 0.034435184 |
| ENSG00000099840 | *IZUMO4* | -2.99470665 | 0.002104414 |
| ENSG00000198763 | *MT-ND2* | -2.996377395 | 1.59E-05 |
| ENSG00000228109 | *MFI2-AS1* | -2.996405764 | 0.000152902 |
| ENSG00000203778 | *FAM229B* | -2.996983389 | 0.004621404 |
| ENSG00000105639 | *JAK3* | -2.997962068 | 0.020431937 |
| ENSG00000181404 | *WASH1* | -3.000896735 | 0.000111731 |
| ENSG00000114013 | *CD86* | -3.002389789 | 0.038867677 |
| ENSG00000196511 | *TPK1* | -3.003724798 | 0.015524488 |
| ENSG00000184381 | *PLA2G6* | -3.003826196 | 0.001585546 |
| ENSG00000137098 | *SPAG8* | -3.006673506 | 0.006165693 |
| ENSG00000188185 | *LINC00265* | -3.007811571 | 0.041497863 |
| ENSG00000262580 | *RP11-334C17.5* | -3.010842538 | 0.011187014 |
| ENSG00000171812 | *COL8A2* | -3.014148281 | 0.019848724 |
| ENSG00000278156 | *TSC22D1-AS1* | -3.015663807 | 0.047863649 |
| ENSG00000269293 | *ZSCAN16-AS1* | -3.021386053 | 0.008774243 |
| ENSG00000130518 | *KIAA1683* | -3.021703777 | 0.035718002 |
| ENSG00000134539 | *KLRD1* | -3.022089396 | 0.017944298 |
| ENSG00000218227 | *RP11-889L3.1* | -3.023441053 | 0.00358847 |
| ENSG00000091986 | *CCDC80* | -3.02425857 | 0.007986537 |
| ENSG00000021645 | *NRXN3* | -3.030653389 | 0.000814206 |
| ENSG00000168646 | *AXIN2* | -3.033251525 | 0.003557536 |
| ENSG00000126882 | *FAM78A* | -3.033415306 | 0.010209938 |
| ENSG00000221890 | *NPTXR* | -3.034581114 | 0.007177741 |
| ENSG00000184304 | *PRKD1* | -3.035446645 | 0.001219859 |
| ENSG00000073756 | *PTGS2* | -3.03616107 | 0.018317593 |
| ENSG00000120820 | *GLT8D2* | -3.040639203 | 0.028360709 |
| ENSG00000064989 | *CALCRL* | -3.041419275 | 0.005541847 |
| ENSG00000144152 | *FBLN7* | -3.046633919 | 0.020888022 |
| ENSG00000273686 | *B2M* | -3.047868626 | 0.003465319 |
| ENSG00000140682 | *TGFB1I1* | -3.048078171 | 2.19E-05 |
| ENSG00000166710 | *B2M* | -3.048783777 | 1.21E-05 |
| ENSG00000278376 | *RP11-158I9.8* | -3.050430146 | 0.011613315 |
| ENSG00000155657 | *TTN* | -3.051880981 | 0.039005826 |
| ENSG00000138685 | *FGF2* | -3.052325751 | 0.002419324 |
| ENSG00000168026 | *TTC21A* | -3.05474225 | 2.62E-05 |
| ENSG00000026950 | *BTN3A1* | -3.057686221 | 0.001693289 |
| ENSG00000162639 | *HENMT1* | -3.059676902 | 0.00207847 |
| ENSG00000084636 | *COL16A1* | -3.05987606 | 0.000207817 |
| ENSG00000198888 | *MT-ND1* | -3.060708849 | 9.91E-05 |
| ENSG00000168481 | *LGI3* | -3.062824857 | 0.009605133 |
| ENSG00000129749 | *CHRNA10* | -3.068096922 | 0.04328573 |
| ENSG00000184060 | *ADAP2* | -3.071056365 | 0.006931817 |
| ENSG00000272645 | *RP11-504P24.8* | -3.071742916 | 0.000398842 |
| ENSG00000275888 | *RP13-516M14.10* | -3.08574588 | 0.044377767 |
| ENSG00000167604 | *NFKBID* | -3.086757378 | 0.000494582 |
| ENSG00000117479 | *SLC19A2* | -3.087250192 | 0.001161111 |
| ENSG00000174808 | *BTC* | -3.093626414 | 0.013308402 |
| ENSG00000145569 | *FAM105A* | -3.095764977 | 0.000987675 |
| ENSG00000231160 | *KLF3-AS1* | -3.097328051 | 0.009424518 |
| ENSG00000186470 | *BTN3A2* | -3.097458955 | 0.000567712 |
| ENSG00000158887 | *MPZ* | -3.099961149 | 0.016317039 |
| ENSG00000204131 | *NHSL2* | -3.101278194 | 0.01905371 |
| ENSG00000134986 | *NREP* | -3.105516379 | 0.002285438 |
| ENSG00000183963 | *SMTN* | -3.107037977 | 1.59E-05 |
| ENSG00000172379 | *ARNT2* | -3.107434744 | 0.019586513 |
| ENSG00000152078 | *TMEM56* | -3.109102341 | 0.023066067 |
| ENSG00000078804 | *TP53INP2* | -3.110051323 | 0.002447421 |
| ENSG00000183281 | *PLGLB1* | -3.110355739 | 0.001785241 |
| ENSG00000214548 | *MEG3* | -3.110510317 | 1.72E-08 |
| ENSG00000234585 | *CCT6P3* | -3.118983477 | 2.22E-05 |
| ENSG00000266967 | *AARSD1* | -3.123098652 | 0.047620828 |
| ENSG00000082512 | *TRAF5* | -3.123698163 | 2.33E-05 |
| ENSG00000079150 | *FKBP7* | -3.124512008 | 0.002625055 |
| ENSG00000198840 | *MT-ND3* | -3.125623559 | 5.96E-05 |
| ENSG00000029534 | *ANK1* | -3.126296529 | 0.01593727 |
| ENSG00000100968 | *NFATC4* | -3.130031258 | 6.59E-08 |
| ENSG00000206559 | *ZCWPW2* | -3.130380733 | 0.036041157 |
| ENSG00000146070 | *PLA2G7* | -3.132493767 | 0.019899241 |
| ENSG00000112425 | *EPM2A* | -3.133328914 | 9.54E-05 |
| ENSG00000164776 | *PHKG1* | -3.140691078 | 0.021137622 |
| ENSG00000136895 | *GARNL3* | -3.143294608 | 2.56E-05 |
| ENSG00000100292 | *HMOX1* | -3.148680323 | 0.00320574 |
| ENSG00000178685 | *PARP10* | -3.148688587 | 0.004115242 |
| ENSG00000157514 | *TSC22D3* | -3.148864067 | 0.000475516 |
| ENSG00000159884 | *CCDC107* | -3.160178516 | 0.001320944 |
| ENSG00000172465 | *TCEAL1* | -3.160856771 | 0.000597068 |
| ENSG00000092841 | *MYL6* | -3.162830339 | 0.000807984 |
| ENSG00000150556 | *LYPD6B* | -3.167099711 | 0.025709005 |
| ENSG00000134072 | *CAMK1* | -3.167441291 | 0.001250426 |
| ENSG00000181045 | *SLC26A11* | -3.171858956 | 4.32E-05 |
| ENSG00000196209 | *SIRPB2* | -3.178500624 | 0.034577798 |
| ENSG00000141337 | *ARSG* | -3.18611008 | 0.003847697 |
| ENSG00000135596 | *MICAL1* | -3.18616849 | 0.001109679 |
| ENSG00000106070 | *GRB10* | -3.196150677 | 0.002040093 |
| ENSG00000113555 | *PCDH12* | -3.196601961 | 0.000818963 |
| ENSG00000261353 | *CTA-14H9.5* | -3.197202228 | 0.046751025 |
| ENSG00000226396 | *RP5-1056L3.3* | -3.198891523 | 0.031766317 |
| ENSG00000113108 | *APBB3* | -3.202722363 | 4.31E-05 |
| ENSG00000066185 | *ZMYND12* | -3.211625089 | 0.018659893 |
| ENSG00000135407 | *AVIL* | -3.212885989 | 0.002512151 |
| ENSG00000082196 | *C1QTNF3* | -3.212950705 | 0.0081891 |
| ENSG00000124713 | *GNMT* | -3.214383744 | 0.047102162 |
| ENSG00000159496 | *RGL4* | -3.216019971 | 0.048111581 |
| ENSG00000162461 | *SLC25A34* | -3.222729528 | 0.037012103 |
| ENSG00000204815 | *TTC25* | -3.224145379 | 0.017621878 |
| ENSG00000100116 | *GCAT* | -3.225822491 | 1.02E-06 |
| ENSG00000136883 | *KIF12* | -3.227367273 | 0.030562518 |
| ENSG00000115325 | *DOK1* | -3.228481213 | 0.001866301 |
| ENSG00000143341 | *HMCN1* | -3.237036151 | 0.038635097 |
| ENSG00000169122 | *FAM110B* | -3.237969693 | 0.007272457 |
| ENSG00000159176 | *CSRP1* | -3.241034701 | 0.000244336 |
| ENSG00000171385 | *KCND3* | -3.241410306 | 3.76E-05 |
| ENSG00000109794 | *FAM149A* | -3.244056838 | 0.00065159 |
| ENSG00000143416 | *SELENBP1* | -3.244551748 | 8.38E-05 |
| ENSG00000136147 | *PHF11* | -3.246140644 | 5.74E-07 |
| ENSG00000225285 | *RP4-758J18.10* | -3.251133524 | 0.010680751 |
| ENSG00000172426 | *RSPH9* | -3.255367661 | 0.042462582 |
| ENSG00000176485 | *PLA2G16* | -3.257274539 | 0.010327139 |
| ENSG00000220201 | *ZGLP1* | -3.257418521 | 0.012814265 |
| ENSG00000272143 | *FGF14-AS2* | -3.258575799 | 0.011007595 |
| ENSG00000174804 | *FZD4* | -3.261033504 | 5.61E-06 |
| ENSG00000228299 | *HLA-C* | -3.265723999 | 2.03E-05 |
| ENSG00000213398 | *LCAT* | -3.267851225 | 1.15E-05 |
| ENSG00000165113 | *GKAP1* | -3.270383959 | 0.000732929 |
| ENSG00000174125 | *TLR1* | -3.271194591 | 0.003826188 |
| ENSG00000074706 | *IPCEF1* | -3.272138593 | 0.027014952 |
| ENSG00000056998 | *GYG2* | -3.272356824 | 0.000384583 |
| ENSG00000157554 | *ERG* | -3.273372672 | 4.73E-05 |
| ENSG00000215481 | *BCRP3* | -3.274050999 | 0.027579041 |
| ENSG00000246985 | *SOCS2-AS1* | -3.274130179 | 0.000626995 |
| ENSG00000108830 | *RND2* | -3.277561034 | 0.000664325 |
| ENSG00000171533 | *MAP6* | -3.281515981 | 0.000252033 |
| ENSG00000168060 | *NAALADL1* | -3.283941385 | 0.002442899 |
| ENSG00000063127 | *SLC6A16* | -3.290001091 | 0.000682797 |
| ENSG00000182109 | *RP11-69E11.4* | -3.29181472 | 0.015371384 |
| ENSG00000196159 | *FAT4* | -3.293714775 | 0.002638646 |
| ENSG00000107242 | *PIP5K1B* | -3.295799055 | 0.018704483 |
| ENSG00000183615 | *FAM167B* | -3.296140608 | 0.000174057 |
| ENSG00000135929 | *CYP27A1* | -3.300508316 | 0.000143953 |
| ENSG00000167549 | *CORO6* | -3.305484486 | 0.000533429 |
| ENSG00000204397 | *CARD16* | -3.306364909 | 0.013855325 |
| ENSG00000134871 | *COL4A2* | -3.307316673 | 0.010648454 |
| ENSG00000069399 | *BCL3* | -3.308253487 | 0.021201439 |
| ENSG00000144724 | *PTPRG* | -3.31297301 | 0.00805742 |
| ENSG00000239569 | *KMT2E-AS1* | -3.31360093 | 0.000326449 |
| ENSG00000120658 | *ENOX1* | -3.317243171 | 0.005932956 |
| ENSG00000267534 | *S1PR2* | -3.318296946 | 0.001537678 |
| ENSG00000228409 | *CCT6P1* | -3.322039254 | 0.000953816 |
| ENSG00000048052 | *HDAC9* | -3.322639727 | 0.000871926 |
| ENSG00000077782 | *FGFR1* | -3.32509343 | 7.76E-06 |
| ENSG00000114698 | *PLSCR4* | -3.33009115 | 1.88E-05 |
| ENSG00000165028 | *NIPSNAP3B* | -3.330489028 | 0.002820947 |
| ENSG00000175928 | *LRRN1* | -3.333471403 | 0.000671991 |
| ENSG00000276141 | *WHAMMP3* | -3.33463022 | 0.003444581 |
| ENSG00000241839 | *PLEKHO2* | -3.339816251 | 1.92E-05 |
| ENSG00000206561 | *COLQ* | -3.339865669 | 0.00355336 |
| ENSG00000179909 | *ZNF154* | -3.341650239 | 0.001179822 |
| ENSG00000025708 | *TYMP* | -3.344141647 | 0.017316388 |
| ENSG00000092758 | *COL9A3* | -3.344553198 | 0.021160565 |
| ENSG00000196843 | *ARID5A* | -3.348175108 | 7.07E-05 |
| ENSG00000025039 | *RRAGD* | -3.350120627 | 0.002676242 |
| ENSG00000247516 | *MIR4458HG* | -3.350874256 | 0.001532611 |
| ENSG00000205517 | *RGL3* | -3.353957217 | 0.008477931 |
| ENSG00000136383 | *ALPK3* | -3.354828375 | 0.012856133 |
| ENSG00000106991 | *ENG* | -3.356007044 | 5.58E-07 |
| ENSG00000119946 | *CNNM1* | -3.357765095 | 0.001077358 |
| ENSG00000140416 | *TPM1* | -3.358224068 | 0.001130467 |
| ENSG00000069702 | *TGFBR3* | -3.358708435 | 0.00665341 |
| ENSG00000150995 | *ITPR1* | -3.362746145 | 0.000130371 |
| ENSG00000196167 | *COLCA1* | -3.364555798 | 0.019848724 |
| ENSG00000079308 | *TNS1* | -3.367092579 | 0.000896341 |
| ENSG00000236333 | *TRHDE-AS1* | -3.36931924 | 0.020800531 |
| ENSG00000151376 | *ME3* | -3.369391063 | 6.69E-07 |
| ENSG00000272870 | *RP11-798M19.6* | -3.372768675 | 0.001032939 |
| ENSG00000181634 | *TNFSF15* | -3.37380599 | 0.02133329 |
| ENSG00000134020 | *PEBP4* | -3.382945441 | 0.00117874 |
| ENSG00000243696 | *RP5-966M1.6* | -3.383046876 | 0.011300826 |
| ENSG00000048740 | *CELF2* | -3.383151532 | 5.68E-06 |
| ENSG00000261584 | *RP11-457M11.5* | -3.385582115 | 0.005071976 |
| ENSG00000077684 | *JADE1* | -3.393009891 | 0.000224116 |
| ENSG00000167315 | *ACAA2* | -3.394117012 | 0.000199555 |
| ENSG00000061918 | *GUCY1B3* | -3.395637312 | 0.004826646 |
| ENSG00000088756 | *ARHGAP28* | -3.397742706 | 0.024856141 |
| ENSG00000266865 | *RP11-848P1.9* | -3.405143956 | 0.004450834 |
| ENSG00000203814 | *HIST2H2BF* | -3.40616581 | 0.02326174 |
| ENSG00000176971 | *FIBIN* | -3.410304936 | 0.002253635 |
| ENSG00000008226 | *DLEC1* | -3.411088531 | 0.030871949 |
| ENSG00000122986 | *HVCN1* | -3.413065734 | 0.002553926 |
| ENSG00000102003 | *SYP* | -3.415724146 | 0.027178135 |
| ENSG00000232611 | *RP11-1114A5.4* | -3.417165583 | 0.002470337 |
| ENSG00000231721 | *LINC-PINT* | -3.419693606 | 1.03E-06 |
| ENSG00000155970 | *MICU3* | -3.419774992 | 0.001018147 |
| ENSG00000186204 | *CYP4F12* | -3.425029718 | 0.028626227 |
| ENSG00000168899 | *VAMP5* | -3.425237266 | 0.000621462 |
| ENSG00000174514 | *MFSD4* | -3.425352459 | 0.009263945 |
| ENSG00000133321 | *RARRES3* | -3.430995196 | 0.000896233 |
| ENSG00000186998 | *EMID1* | -3.435488381 | 0.000626995 |
| ENSG00000197355 | *UAP1L1* | -3.436544906 | 6.78E-05 |
| ENSG00000101347 | *SAMHD1* | -3.438738493 | 5.51E-07 |
| ENSG00000166920 | *C15orf48* | -3.442501466 | 0.021838293 |
| ENSG00000115604 | *IL18R1* | -3.444279747 | 0.004257009 |
| ENSG00000198417 | *MT1F* | -3.447592467 | 0.000589292 |
| ENSG00000165449 | *SLC16A9* | -3.44805465 | 0.010811746 |
| ENSG00000159713 | *TPPP3* | -3.450224855 | 0.000191636 |
| ENSG00000272821 | *CTA-384D8.36* | -3.450398621 | 0.023052857 |
| ENSG00000229481 | *CTD-2554C21.3* | -3.452122738 | 0.022996293 |
| ENSG00000096060 | *FKBP5* | -3.454492057 | 0.007969826 |
| ENSG00000078814 | *MYH7B* | -3.458696158 | 0.00769501 |
| ENSG00000282670 | *RP11-932O9.9* | -3.467946789 | 0.041064445 |
| ENSG00000175471 | *MCTP1* | -3.471447822 | 0.01209139 |
| ENSG00000134508 | *CABLES1* | -3.472793876 | 0.020197836 |
| ENSG00000078114 | *NEBL* | -3.472831542 | 0.002041192 |
| ENSG00000147408 | *CSGALNACT1* | -3.473833707 | 0.000909816 |
| ENSG00000251141 | *RP11-53O19.1* | -3.474731936 | 0.046544969 |
| ENSG00000107099 | *DOCK8* | -3.479679321 | 0.000244901 |
| ENSG00000102886 | *GDPD3* | -3.480684821 | 0.000353097 |
| ENSG00000179698 | *KIAA1875* | -3.48117518 | 0.043300948 |
| ENSG00000272221 | *XXbac-BPG181B23.7* | -3.482495525 | 0.014920132 |
| ENSG00000135636 | *DYSF* | -3.485397546 | 0.00352591 |
| ENSG00000104953 | *TLE6* | -3.485923676 | 0.004290418 |
| ENSG00000008311 | *AASS* | -3.486867414 | 0.00021188 |
| ENSG00000115935 | *WIPF1* | -3.488701754 | 2.03E-05 |
| ENSG00000175040 | *CHST2* | -3.493818247 | 0.013190829 |
| ENSG00000135218 | *CD36* | -3.497319409 | 0.005300949 |
| ENSG00000128714 | *HOXD13* | -3.498610604 | 0.018974243 |
| ENSG00000205085 | *FAM71F2* | -3.503304773 | 0.023571561 |
| ENSG00000089041 | *P2RX7* | -3.505808833 | 0.000365207 |
| ENSG00000182916 | *TCEAL7* | -3.50774657 | 0.006962715 |
| ENSG00000183833 | *MAATS1* | -3.509288699 | 0.000212437 |
| ENSG00000220563 | *PKMP3* | -3.510270252 | 0.049908381 |
| ENSG00000134627 | *PIWIL4* | -3.510642349 | 0.001427788 |
| ENSG00000203797 | *DDO* | -3.514936997 | 0.001155745 |
| ENSG00000164197 | *RNF180* | -3.517089512 | 0.005571854 |
| ENSG00000163762 | *TM4SF18* | -3.520479471 | 0.025132858 |
| ENSG00000172889 | *EGFL7* | -3.524657095 | 8.70E-07 |
| ENSG00000214274 | *ANG* | -3.526014957 | 0.000149597 |
| ENSG00000272669 | *RP3-508I15.21* | -3.527419856 | 0.018125273 |
| ENSG00000224596 | *ZMIZ1-AS1* | -3.529923749 | 0.003697231 |
| ENSG00000187796 | *CARD9* | -3.533386716 | 0.000689624 |
| ENSG00000197054 | *ZNF763* | -3.534317923 | 0.023376838 |
| ENSG00000206417 | *H1FX-AS1* | -3.534947605 | 0.000734158 |
| ENSG00000126878 | *AIF1L* | -3.542773906 | 0.000468785 |
| ENSG00000167984 | *NLRC3* | -3.543255929 | 0.00094237 |
| ENSG00000162595 | *DIRAS3* | -3.544366454 | 0.017025785 |
| ENSG00000151617 | *EDNRA* | -3.546413655 | 0.000413356 |
| ENSG00000004399 | *PLXND1* | -3.547843716 | 1.47E-06 |
| ENSG00000177694 | *NAALADL2* | -3.553022111 | 5.18E-05 |
| ENSG00000077157 | *PPP1R12B* | -3.553543101 | 5.01E-09 |
| ENSG00000169885 | *CALML6* | -3.560208284 | 0.005333177 |
| ENSG00000134245 | *WNT2B* | -3.561497694 | 5.47E-05 |
| ENSG00000150637 | *CD226* | -3.563532502 | 0.045539165 |
| ENSG00000101210 | *EEF1A2* | -3.564853898 | 0.026346432 |
| ENSG00000128016 | *ZFP36* | -3.565630001 | 4.88E-05 |
| ENSG00000259658 | *RP11-89K11.1* | -3.566777491 | 0.005185864 |
| ENSG00000249242 | *TMEM150C* | -3.567672804 | 0.033843828 |
| ENSG00000272462 | *U91328.19* | -3.569900395 | 0.000327503 |
| ENSG00000153404 | *PLEKHG4B* | -3.571091263 | 0.047289418 |
| ENSG00000165121 | *RP11-213G2.3* | -3.5757289 | 5.10E-05 |
| ENSG00000133874 | *RNF122* | -3.582038257 | 0.000436048 |
| ENSG00000128298 | *BAIAP2L2* | -3.58351399 | 0.048619998 |
| ENSG00000170074 | *FAM153A* | -3.594610729 | 0.000107008 |
| ENSG00000101187 | *SLCO4A1* | -3.598211775 | 0.010484951 |
| ENSG00000124191 | *TOX2* | -3.604841307 | 0.00032608 |
| ENSG00000173705 | *SUSD5* | -3.606287094 | 0.048799082 |
| ENSG00000184226 | *PCDH9* | -3.611799296 | 0.015220221 |
| ENSG00000156265 | *MAP3K7CL* | -3.613701727 | 0.004894174 |
| ENSG00000184205 | *TSPYL2* | -3.613814302 | 3.40E-09 |
| ENSG00000119714 | *GPR68* | -3.616953801 | 0.001051102 |
| ENSG00000162572 | *SCNN1D* | -3.619653516 | 0.000372244 |
| ENSG00000237187 | *NR2F1-AS1* | -3.620429598 | 0.037274274 |
| ENSG00000226237 | *RP11-276H19.1* | -3.629465394 | 0.0186403 |
| ENSG00000249992 | *TMEM158* | -3.634888421 | 1.27E-05 |
| ENSG00000279821 | *RP11-1334A24.5* | -3.634972906 | 0.003731817 |
| ENSG00000120327 | *PCDHB14* | -3.63635814 | 0.046311641 |
| ENSG00000143878 | *RHOB* | -3.64382002 | 4.32E-06 |
| ENSG00000281650 | *CH17-64J14.5* | -3.645143037 | 0.014584241 |
| ENSG00000161265 | *U2AF1L4* | -3.647914493 | 1.88E-05 |
| ENSG00000151789 | *ZNF385D* | -3.650621364 | 0.000213506 |
| ENSG00000277449 | *CEBPB-AS1* | -3.664663826 | 0.006086721 |
| ENSG00000133067 | *LGR6* | -3.667618777 | 0.036664474 |
| ENSG00000260572 | *RP11-16N11.2* | -3.678831611 | 0.016863834 |
| ENSG00000133246 | *PRAM1* | -3.686192575 | 0.002186972 |
| ENSG00000115318 | *LOXL3* | -3.687141229 | 0.000346642 |
| ENSG00000227953 | *LINC01341* | -3.689056529 | 0.036808979 |
| ENSG00000179598 | *PLD6* | -3.689312936 | 1.73E-05 |
| ENSG00000095794 | *CREM* | -3.690899527 | 2.20E-10 |
| ENSG00000007944 | *MYLIP* | -3.691286775 | 8.74E-06 |
| ENSG00000183578 | *TNFAIP8L3* | -3.692348739 | 0.001582983 |
| ENSG00000102524 | *TNFSF13B* | -3.69259452 | 0.004149109 |
| ENSG00000178623 | *GPR35* | -3.694625217 | 0.006266172 |
| ENSG00000036672 | *USP2* | -3.696473549 | 0.000206858 |
| ENSG00000170345 | *FOS* | -3.697567633 | 0.002467281 |
| ENSG00000187608 | *ISG15* | -3.704733659 | 0.000155507 |
| ENSG00000177989 | *ODF3B* | -3.70508842 | 0.00032928 |
| ENSG00000162804 | *SNED1* | -3.70840584 | 0.000107226 |
| ENSG00000272086 | *CTD-2186M15.3* | -3.708712291 | 0.009424518 |
| ENSG00000112208 | *BAG2* | -3.709453955 | 8.14E-05 |
| ENSG00000116157 | *GPX7* | -3.711338625 | 0.000734491 |
| ENSG00000231367 | *AC016995.3* | -3.715041009 | 0.030786119 |
| ENSG00000198754 | *OXCT2* | -3.715995315 | 0.044070473 |
| ENSG00000151702 | *FLI1* | -3.717628257 | 0.004609882 |
| ENSG00000103196 | *CRISPLD2* | -3.719253696 | 1.01E-07 |
| ENSG00000100060 | *MFNG* | -3.721517193 | 0.000327503 |
| ENSG00000162944 | *RFTN2* | -3.725312482 | 0.005013652 |
| ENSG00000168734 | *PKIG* | -3.72949122 | 8.95E-05 |
| ENSG00000111077 | *TNS2* | -3.730417675 | 4.15E-07 |
| ENSG00000112320 | *SOBP* | -3.732111744 | 2.60E-07 |
| ENSG00000262877 | *RP11-1055B8.4* | -3.736804083 | 0.019084378 |
| ENSG00000108960 | *MMD* | -3.740737338 | 0.005643633 |
| ENSG00000198948 | *MFAP3L* | -3.7430324 | 0.002743391 |
| ENSG00000275367 | *RP11-266K4.14* | -3.744095689 | 0.000628055 |
| ENSG00000138356 | *AOX1* | -3.747391417 | 0.000293976 |
| ENSG00000100599 | *RIN3* | -3.749243768 | 1.14E-05 |
| ENSG00000231856 | *RP11-327P2.5* | -3.752188676 | 0.034669952 |
| ENSG00000263874 | *LINC00672* | -3.753562093 | 0.01258841 |
| ENSG00000126016 | *AMOT* | -3.754269001 | 0.000136306 |
| ENSG00000113140 | *SPARC* | -3.755060478 | 0.00022028 |
| ENSG00000271869 | *RP11-51J9.5* | -3.762026508 | 0.037980514 |
| ENSG00000267414 | *RP11-456K23.1* | -3.762675903 | 0.009432907 |
| ENSG00000235790 | *RP11-73M7.6* | -3.763785068 | 0.027579041 |
| ENSG00000205403 | *CFI* | -3.765463641 | 0.000172655 |
| ENSG00000138639 | *ARHGAP24* | -3.769619739 | 0.000689467 |
| ENSG00000168405 | *CMAHP* | -3.78198026 | 1.06E-06 |
| ENSG00000160460 | *SPTBN4* | -3.782961196 | 0.000534994 |
| ENSG00000116117 | *PARD3B* | -3.784595148 | 0.00034223 |
| ENSG00000117228 | *GBP1* | -3.785576978 | 5.27E-07 |
| ENSG00000154620 | *TMSB4Y* | -3.788323839 | 0.003426904 |
| ENSG00000164972 | *C9orf24* | -3.791162848 | 0.011777581 |
| ENSG00000196154 | *S100A4* | -3.799131212 | 0.000665615 |
| ENSG00000165072 | *MAMDC2* | -3.803087693 | 0.001751613 |
| ENSG00000121316 | *PLBD1* | -3.803439385 | 0.000693504 |
| ENSG00000225880 | *LINC00115* | -3.803559543 | 0.015014755 |
| ENSG00000184986 | *TMEM121* | -3.803966774 | 0.000928426 |
| ENSG00000035862 | *TIMP2* | -3.805074442 | 9.22E-08 |
| ENSG00000112394 | *SLC16A10* | -3.806189734 | 0.018332493 |
| ENSG00000137198 | *GMPR* | -3.807738371 | 1.23E-07 |
| ENSG00000102385 | *DRP2* | -3.812810638 | 0.002665005 |
| ENSG00000177685 | *CRACR2B* | -3.813055513 | 0.004368557 |
| ENSG00000244968 | *LIFR-AS1* | -3.81971082 | 0.041459535 |
| ENSG00000047597 | *XK* | -3.822872084 | 0.01979817 |
| ENSG00000198838 | *RYR3* | -3.823807787 | 0.000566994 |
| ENSG00000159433 | *STARD9* | -3.825690177 | 5.08E-05 |
| ENSG00000143882 | *ATP6V1C2* | -3.832073368 | 0.018253835 |
| ENSG00000144655 | *CSRNP1* | -3.834563897 | 4.73E-05 |
| ENSG00000172183 | *ISG20* | -3.838172114 | 0.00228271 |
| ENSG00000149201 | *CCDC81* | -3.850999085 | 0.027679975 |
| ENSG00000124249 | *KCNK15* | -3.851116466 | 0.019912781 |
| ENSG00000271851 | *RP11-565F19.2* | -3.852789533 | 0.025348236 |
| ENSG00000251661 | *RP11-326C3.11* | -3.855099096 | 0.002425767 |
| ENSG00000226124 | *FTCDNL1* | -3.857603648 | 0.035004923 |
| ENSG00000166257 | *SCN3B* | -3.861655852 | 0.030840113 |
| ENSG00000196730 | *DAPK1* | -3.862229869 | 0.004803395 |
| ENSG00000181804 | *SLC9A9* | -3.864306913 | 8.54E-06 |
| ENSG00000170577 | *SIX2* | -3.865896084 | 0.005045127 |
| ENSG00000153814 | *JAZF1* | -3.868635746 | 4.86E-05 |
| ENSG00000073670 | *ADAM11* | -3.872096617 | 0.002614834 |
| ENSG00000184922 | *FMNL1* | -3.872741661 | 0.000638536 |
| ENSG00000142494 | *SLC47A1* | -3.873599804 | 0.000338107 |
| ENSG00000196793 | *ZNF239* | -3.876405559 | 0.003784988 |
| ENSG00000206077 | *ZDHHC11B* | -3.877257487 | 0.029913955 |
| ENSG00000197558 | *SSPO* | -3.877331226 | 0.023701413 |
| ENSG00000183960 | *KCNH8* | -3.878741734 | 0.033037226 |
| ENSG00000135905 | *DOCK10* | -3.878748312 | 0.030562518 |
| ENSG00000231793 | *DOC2GP* | -3.878798827 | 0.023013283 |
| ENSG00000261884 | *CTC-479C5.12* | -3.878821388 | 0.001345213 |
| ENSG00000102878 | *HSF4* | -3.879387649 | 1.07E-05 |
| ENSG00000273802 | *HIST1H2BG* | -3.881276227 | 0.000679771 |
| ENSG00000130844 | *ZNF331* | -3.887480466 | 9.92E-08 |
| ENSG00000104967 | *NOVA2* | -3.887608767 | 0.034796045 |
| ENSG00000274137 | *MYOM2* | -3.903363746 | 0.027219854 |
| ENSG00000166405 | *RIC3* | -3.904104644 | 6.76E-05 |
| ENSG00000165795 | *NDRG2* | -3.90795521 | 3.95E-05 |
| ENSG00000167995 | *BEST1* | -3.910214315 | 0.000139753 |
| ENSG00000129946 | *SHC2* | -3.919758206 | 0.012001769 |
| ENSG00000111348 | *ARHGDIB* | -3.923093296 | 0.000548358 |
| ENSG00000163121 | *NEURL3* | -3.935521785 | 0.046257139 |
| ENSG00000254995 | *STX16-NPEPL1* | -3.935904548 | 0.022200906 |
| ENSG00000220785 | *MTMR9LP* | -3.937503744 | 1.23E-06 |
| ENSG00000159761 | *C16orf86* | -3.937586614 | 0.001485064 |
| ENSG00000091972 | *CD200* | -3.937995307 | 4.48E-05 |
| ENSG00000101460 | *MAP1LC3A* | -3.943079268 | 1.93E-08 |
| ENSG00000112137 | *PHACTR1* | -3.946508346 | 0.001127057 |
| ENSG00000165410 | *CFL2* | -3.946978617 | 0.000217925 |
| ENSG00000167588 | *GPD1* | -3.947532324 | 0.026068845 |
| ENSG00000197467 | *COL13A1* | -3.947551868 | 4.57E-05 |
| ENSG00000146674 | *IGFBP3* | -3.954583369 | 1.46E-05 |
| ENSG00000121895 | *TMEM156* | -3.957840463 | 0.047449131 |
| ENSG00000198673 | *FAM19A2* | -3.961424566 | 0.025649776 |
| ENSG00000118432 | *CNR1* | -3.965890836 | 0.031543714 |
| ENSG00000245105 | *A2M-AS1* | -3.966194534 | 0.004584703 |
| ENSG00000177721 | *ANXA2R* | -3.970003893 | 0.000436048 |
| ENSG00000056558 | *TRAF1* | -3.973180865 | 1.08E-05 |
| ENSG00000253304 | *TMEM200B* | -3.973775532 | 1.00E-05 |
| ENSG00000112149 | *CD83* | -3.976242622 | 6.10E-05 |
| ENSG00000011590 | *ZBTB32* | -3.977263763 | 0.046677579 |
| ENSG00000132205 | *EMILIN2* | -3.978288328 | 0.000927286 |
| ENSG00000250303 | *RP11-356J5.12* | -3.98380063 | 0.001244964 |
| ENSG00000187498 | *COL4A1* | -3.984100939 | 0.004882808 |
| ENSG00000156218 | *ADAMTSL3* | -3.986930253 | 1.61E-07 |
| ENSG00000103489 | *XYLT1* | -3.988853844 | 6.65E-05 |
| ENSG00000232855 | *AF131217.1* | -3.989028112 | 0.004318531 |
| ENSG00000128606 | *LRRC17* | -3.998306736 | 0.001202824 |
| ENSG00000010610 | *CD4* | -3.99974398 | 0.018769075 |
| ENSG00000184313 | *MROH7* | -4.002773155 | 0.0064739 |
| ENSG00000259605 | *AC074212.5* | -4.00634599 | 0.002636406 |
| ENSG00000279369 | *RP11-51F16.1* | -4.006573979 | 0.020372295 |
| ENSG00000180447 | *GAS1* | -4.009075683 | 0.000348645 |
| ENSG00000134769 | *DTNA* | -4.016395686 | 0.017329794 |
| ENSG00000251143 | *RP11-849H4.4* | -4.016551494 | 0.029136128 |
| ENSG00000172247 | *C1QTNF4* | -4.017153869 | 0.002028722 |
| ENSG00000128709 | *HOXD9* | -4.030720261 | 0.000130057 |
| ENSG00000170955 | *PRKCDBP* | -4.032343258 | 4.11E-06 |
| ENSG00000076864 | *RAP1GAP* | -4.03253846 | 0.00769501 |
| ENSG00000111249 | *CUX2* | -4.03394923 | 0.036099342 |
| ENSG00000163531 | *NFASC* | -4.033957003 | 1.35E-06 |
| ENSG00000105643 | *ARRDC2* | -4.035439747 | 6.07E-09 |
| ENSG00000100593 | *ISM2* | -4.035977591 | 0.028957854 |
| ENSG00000235568 | *NFAM1* | -4.036788163 | 0.000758627 |
| ENSG00000154822 | *PLCL2* | -4.036946847 | 0.007830577 |
| ENSG00000223532 | *HLA-B* | -4.038082717 | 0.000157052 |
| ENSG00000101605 | *MYOM1* | -4.038692068 | 0.010650349 |
| ENSG00000177943 | *MAMDC4* | -4.039245086 | 0.002669225 |
| ENSG00000263155 | *MYZAP* | -4.042758097 | 0.000328316 |
| ENSG00000146094 | *DOK3* | -4.045670552 | 0.001756865 |
| ENSG00000280402 | *RP11-20I23.10* | -4.046542659 | 0.044238367 |
| ENSG00000047648 | *ARHGAP6* | -4.046822908 | 0.000333606 |
| ENSG00000085514 | *PILRA* | -4.052142633 | 6.53E-05 |
| ENSG00000141497 | *ZMYND15* | -4.052324377 | 0.008301388 |
| ENSG00000138670 | *RASGEF1B* | -4.061120442 | 0.000221762 |
| ENSG00000152804 | *HHEX* | -4.063323579 | 0.003240894 |
| ENSG00000213144 | *RP11-64B16.2* | -4.063498914 | 0.004389031 |
| ENSG00000224713 | *AC025165.8* | -4.064913909 | 0.009719154 |
| ENSG00000214212 | *C19orf38* | -4.065387938 | 0.02311078 |
| ENSG00000163072 | *NOSTRIN* | -4.078020327 | 0.035588599 |
| ENSG00000261496 | *RP13-514E23.1* | -4.078231297 | 0.010880221 |
| ENSG00000186377 | *CYP4X1* | -4.078518114 | 0.013980454 |
| ENSG00000087245 | *MMP2* | -4.083475913 | 1.49E-08 |
| ENSG00000136717 | *BIN1* | -4.09330043 | 2.05E-09 |
| ENSG00000161638 | *ITGA5* | -4.097374491 | 3.11E-06 |
| ENSG00000183023 | *SLC8A1* | -4.108183044 | 8.48E-05 |
| ENSG00000122574 | *WIPF3* | -4.108655071 | 7.06E-08 |
| ENSG00000057657 | *PRDM1* | -4.113198861 | 0.005407565 |
| ENSG00000214694 | *ARHGEF33* | -4.116710511 | 0.036433079 |
| ENSG00000188385 | *JAKMIP3* | -4.122706972 | 0.018704483 |
| ENSG00000276075 | *CTD-2012K14.8* | -4.129874034 | 0.011333765 |
| ENSG00000009790 | *TRAF3IP3* | -4.131252535 | 0.003473771 |
| ENSG00000151062 | *CACNA2D4* | -4.136152163 | 0.004586148 |
| ENSG00000104432 | *IL7* | -4.140803287 | 0.005664652 |
| ENSG00000162894 | *FCMR* | -4.141042558 | 0.003845492 |
| ENSG00000244486 | *SCARF2* | -4.142385064 | 3.87E-07 |
| ENSG00000198932 | *GPRASP1* | -4.143834233 | 4.10E-09 |
| ENSG00000171119 | *NRTN* | -4.145179381 | 0.000807984 |
| ENSG00000164742 | *ADCY1* | -4.15021241 | 0.018383998 |
| ENSG00000005513 | *SOX8* | -4.155062537 | 0.002242531 |
| ENSG00000101194 | *SLC17A9* | -4.156706141 | 0.0081891 |
| ENSG00000080493 | *SLC4A4* | -4.158104739 | 0.010089044 |
| ENSG00000241170 | *RP11-147I3.1* | -4.161135224 | 0.00436307 |
| ENSG00000129675 | *ARHGEF6* | -4.167618402 | 1.46E-07 |
| ENSG00000021300 | *PLEKHB1* | -4.168121646 | 0.000501834 |
| ENSG00000164946 | *FREM1* | -4.168567113 | 0.002378398 |
| ENSG00000198467 | *TPM2* | -4.171009523 | 2.33E-05 |
| ENSG00000158555 | *GDPD5* | -4.171145832 | 0.000109054 |
| ENSG00000126562 | *WNK4* | -4.172914358 | 0.002181022 |
| ENSG00000137474 | *MYO7A* | -4.177334694 | 0.005804088 |
| ENSG00000171227 | *TMEM37* | -4.180999426 | 1.29E-05 |
| ENSG00000157404 | *KIT* | -4.185570781 | 0.001167377 |
| ENSG00000072657 | *TRHDE* | -4.190596278 | 0.011696928 |
| ENSG00000187486 | *KCNJ11* | -4.193388494 | 0.010811746 |
| ENSG00000115602 | *IL1RL1* | -4.196236019 | 0.032434772 |
| ENSG00000162366 | *PDZK1IP1* | -4.200285254 | 0.013719771 |
| ENSG00000223756 | *TSSC2* | -4.203093696 | 0.001474196 |
| ENSG00000185437 | *SH3BGR* | -4.205471851 | 0.000722099 |
| ENSG00000162614 | *NEXN* | -4.205869096 | 0.002186972 |
| ENSG00000260807 | *RP11-161M6.2* | -4.216492011 | 0.000347723 |
| ENSG00000184012 | *TMPRSS2* | -4.219468638 | 0.003705204 |
| ENSG00000263812 | *LINC00908* | -4.220972161 | 0.001263832 |
| ENSG00000142959 | *BEST4* | -4.23009665 | 0.018170142 |
| ENSG00000129535 | *NRL* | -4.253151911 | 0.013508366 |
| ENSG00000122691 | *TWIST1* | -4.253250781 | 0.01979817 |
| ENSG00000279117 | *CTD-2562J17.6* | -4.25719067 | 0.002638379 |
| ENSG00000115138 | *POMC* | -4.258348454 | 0.023126523 |
| ENSG00000108622 | *ICAM2* | -4.258608821 | 0.000135125 |
| ENSG00000137558 | *PI15* | -4.259101931 | 7.57E-05 |
| ENSG00000127954 | *STEAP4* | -4.260357662 | 0.00074773 |
| ENSG00000272505 | *RP11-981G7.6* | -4.264746316 | 0.039220224 |
| ENSG00000106078 | *COBL* | -4.266565022 | 5.83E-08 |
| ENSG00000239697 | *TNFSF12* | -4.267318649 | 3.12E-07 |
| ENSG00000185339 | *TCN2* | -4.270711033 | 1.31E-06 |
| ENSG00000235475 | *LINC01372* | -4.271815256 | 0.016337783 |
| ENSG00000148219 | *ASTN2* | -4.272787676 | 9.91E-07 |
| ENSG00000153956 | *CACNA2D1* | -4.277808449 | 0.00076769 |
| ENSG00000168243 | *GNG4* | -4.27875732 | 0.000979524 |
| ENSG00000272927 | *RP11-1191J2.5* | -4.27985111 | 0.024916667 |
| ENSG00000187824 | *TMEM220* | -4.281147493 | 0.000385675 |
| ENSG00000135363 | *LMO2* | -4.282880321 | 1.86E-05 |
| ENSG00000262222 | *RP11-876N24.4* | -4.283034249 | 0.006180836 |
| ENSG00000078596 | *ITM2A* | -4.290334268 | 0.000127289 |
| ENSG00000131378 | *RFTN1* | -4.293792834 | 0.00086744 |
| ENSG00000143494 | *VASH2* | -4.295401198 | 0.012936276 |
| ENSG00000183098 | *GPC6* | -4.299188908 | 0.001017049 |
| ENSG00000233098 | *CCDC144NL-AS1* | -4.30279372 | 0.000814598 |
| ENSG00000165511 | *C10orf25* | -4.303658058 | 0.006500476 |
| ENSG00000187193 | *MT1X* | -4.303659602 | 3.49E-05 |
| ENSG00000152137 | *HSPB8* | -4.308957897 | 2.22E-07 |
| ENSG00000168993 | *CPLX1* | -4.312160817 | 5.60E-05 |
| ENSG00000235501 | *RP4-639F20.1* | -4.314982609 | 1.01E-05 |
| ENSG00000107611 | *CUBN* | -4.317100231 | 0.000242145 |
| ENSG00000110777 | *POU2AF1* | -4.317150325 | 0.010493194 |
| ENSG00000130413 | *STK33* | -4.322984125 | 0.000129675 |
| ENSG00000185499 | *MUC1* | -4.328260401 | 0.000734036 |
| ENSG00000240694 | *PNMA2* | -4.329463468 | 0.035104865 |
| ENSG00000244119 | *PDCL3P4* | -4.332423024 | 0.001250881 |
| ENSG00000184557 | *SOCS3* | -4.333037023 | 4.94E-05 |
| ENSG00000170558 | *CDH2* | -4.335426406 | 0.004461844 |
| ENSG00000239828 | *RP11-446H18.5* | -4.337600695 | 0.036012019 |
| ENSG00000126561 | *STAT5A* | -4.340968749 | 1.09E-08 |
| ENSG00000099860 | *GADD45B* | -4.342133628 | 2.31E-06 |
| ENSG00000092929 | *UNC13D* | -4.351411108 | 1.30E-05 |
| ENSG00000119699 | *TGFB3* | -4.357433675 | 6.64E-14 |
| ENSG00000130201 | *EXOC3L2* | -4.362947298 | 0.003214915 |
| ENSG00000165507 | *C10orf10* | -4.366615533 | 4.92E-06 |
| ENSG00000164619 | *BMPER* | -4.370716593 | 0.002996378 |
| ENSG00000128567 | *PODXL* | -4.371280229 | 4.66E-05 |
| ENSG00000185924 | *RTN4RL1* | -4.37196783 | 0.008782237 |
| ENSG00000226833 | *AC097724.3* | -4.372236662 | 0.000451047 |
| ENSG00000279198 | *CTD-2231E14.2* | -4.377764754 | 0.033163365 |
| ENSG00000249700 | *SRD5A3-AS1* | -4.380499425 | 0.044273153 |
| ENSG00000086289 | *EPDR1* | -4.383003881 | 3.51E-06 |
| ENSG00000143333 | *RGS16* | -4.38891487 | 0.000536903 |
| ENSG00000259207 | *ITGB3* | -4.390229142 | 0.008400163 |
| ENSG00000152377 | *SPOCK1* | -4.393901131 | 0.001186467 |
| ENSG00000188820 | *FAM26F* | -4.393991268 | 0.000964206 |
| ENSG00000254535 | *PABPC4L* | -4.394298022 | 0.001145485 |
| ENSG00000162676 | *GFI1* | -4.39552399 | 0.021018085 |
| ENSG00000165338 | *HECTD2* | -4.397448943 | 5.34E-09 |
| ENSG00000104324 | *CPQ* | -4.401567475 | 5.17E-07 |
| ENSG00000144031 | *ANKRD53* | -4.403962929 | 0.006054732 |
| ENSG00000130948 | *HSD17B3* | -4.412772521 | 0.033043631 |
| ENSG00000176490 | *DIRAS1* | -4.414981449 | 2.18E-06 |
| ENSG00000130038 | *CRACR2A* | -4.418035298 | 0.001242146 |
| ENSG00000176402 | *GJC3* | -4.41893488 | 0.030068642 |
| ENSG00000138207 | *RBP4* | -4.419179856 | 0.014185391 |
| ENSG00000186517 | *ARHGAP30* | -4.427700691 | 0.000445299 |
| ENSG00000138735 | *PDE5A* | -4.432315632 | 4.96E-06 |
| ENSG00000117425 | *PTCH2* | -4.437087142 | 0.00205878 |
| ENSG00000267280 | *TBX2-AS1* | -4.437772316 | 0.001475342 |
| ENSG00000160255 | *ITGB2* | -4.437874245 | 3.76E-05 |
| ENSG00000254750 | *CASP1P2* | -4.439922905 | 0.029679861 |
| ENSG00000231437 | *RP11-88H9.2* | -4.442119974 | 0.001593581 |
| ENSG00000185338 | *SOCS1* | -4.442248168 | 8.68E-07 |
| ENSG00000146205 | *ANO7* | -4.442553231 | 0.000607778 |
| ENSG00000171791 | *BCL2* | -4.447402544 | 1.78E-08 |
| ENSG00000177374 | *HIC1* | -4.45831967 | 3.32E-06 |
| ENSG00000176884 | *GRIN1* | -4.46194063 | 0.023929033 |
| ENSG00000134470 | *IL15RA* | -4.463441172 | 6.90E-06 |
| ENSG00000169439 | *SDC2* | -4.466888631 | 1.43E-08 |
| ENSG00000198959 | *TGM2* | -4.472632388 | 3.77E-11 |
| ENSG00000140749 | *IGSF6* | -4.476027982 | 0.000146455 |
| ENSG00000162882 | *HAAO* | -4.478917794 | 2.95E-10 |
| ENSG00000103472 | *RRN3P2* | -4.481843015 | 0.000151226 |
| ENSG00000130635 | *COL5A1* | -4.483672918 | 5.89E-06 |
| ENSG00000152953 | *STK32B* | -4.485426044 | 0.024378329 |
| ENSG00000165617 | *DACT1* | -4.488078275 | 1.43E-06 |
| ENSG00000160145 | *KALRN* | -4.489621445 | 0.000291246 |
| ENSG00000260428 | *SCX* | -4.490305887 | 0.000635075 |
| ENSG00000254851 | *RP11-109L13.1* | -4.495913481 | 0.025874981 |
| ENSG00000137965 | *IFI44* | -4.497441874 | 7.19E-05 |
| ENSG00000153012 | *LGI2* | -4.501355166 | 0.017426817 |
| ENSG00000214872 | *SMTNL1* | -4.502302127 | 0.038871915 |
| ENSG00000130307 | *USHBP1* | -4.505852357 | 0.00373165 |
| ENSG00000279384 | *RP11-635L1.2* | -4.512114983 | 0.0437603 |
| ENSG00000242282 | *AC108488.4* | -4.512775541 | 0.000357467 |
| ENSG00000197594 | *ENPP1* | -4.519921562 | 5.11E-06 |
| ENSG00000132329 | *RAMP1* | -4.530255168 | 4.97E-07 |
| ENSG00000186462 | *NAP1L2* | -4.53394454 | 0.000634343 |
| ENSG00000080854 | *IGSF9B* | -4.536700365 | 0.003247247 |
| ENSG00000280219 | *RP11-752L20.3* | -4.537609769 | 0.035520946 |
| ENSG00000117477 | *CCDC181* | -4.548121674 | 0.002662868 |
| ENSG00000175745 | *NR2F1* | -4.549075157 | 0.000155712 |
| ENSG00000116039 | *ATP6V1B1* | -4.550962189 | 0.011675482 |
| ENSG00000185527 | *PDE6G* | -4.55627062 | 0.02774546 |
| ENSG00000181856 | *SLC2A4* | -4.556724108 | 1.68E-08 |
| ENSG00000173068 | *BNC2* | -4.557566517 | 0.000151226 |
| ENSG00000174672 | *BRSK2* | -4.562157007 | 0.004369542 |
| ENSG00000163644 | *PPM1K* | -4.564935128 | 1.40E-08 |
| ENSG00000224383 | *PRR29* | -4.567190747 | 0.009973898 |
| ENSG00000162687 | *KCNT2* | -4.577097754 | 0.004057105 |
| ENSG00000186648 | *LRRC16B* | -4.592080602 | 0.041946769 |
| ENSG00000089692 | *LAG3* | -4.592640114 | 0.00091334 |
| ENSG00000174807 | *CD248* | -4.605592511 | 0.001300027 |
| ENSG00000135439 | *AGAP2* | -4.613925666 | 0.0015087 |
| ENSG00000259976 | *RP11-553L6.5* | -4.615973696 | 8.12E-08 |
| ENSG00000168306 | *ACOX2* | -4.619851703 | 1.97E-05 |
| ENSG00000237940 | *AC093642.3* | -4.621259567 | 0.034542777 |
| ENSG00000240771 | *ARHGEF25* | -4.621565092 | 1.80E-09 |
| ENSG00000142347 | *MYO1F* | -4.624519005 | 2.17E-06 |
| ENSG00000204936 | *CD177* | -4.625936631 | 0.014503078 |
| ENSG00000109610 | *SOD3* | -4.633778446 | 4.19E-07 |
| ENSG00000132386 | *SERPINF1* | -4.635360048 | 8.81E-09 |
| ENSG00000206435 | *HLA-C* | -4.655058863 | 0.039602428 |
| ENSG00000120262 | *CCDC170* | -4.661422897 | 0.00038408 |
| ENSG00000125144 | *MT1G* | -4.663287097 | 6.69E-07 |
| ENSG00000136490 | *LIMD2* | -4.664692279 | 0.000429308 |
| ENSG00000103534 | *TMC5* | -4.669392962 | 0.006618463 |
| ENSG00000203697 | *CAPN8* | -4.672932388 | 0.013105778 |
| ENSG00000279140 | *RP11-477I4.4* | -4.676288145 | 0.009716996 |
| ENSG00000247982 | *LINC00926* | -4.678135777 | 0.000604983 |
| ENSG00000113083 | *LOX* | -4.683336136 | 0.000271905 |
| ENSG00000228221 | *LINC00578* | -4.683769837 | 0.010448519 |
| ENSG00000172164 | *SNTB1* | -4.684400618 | 3.87E-06 |
| ENSG00000111186 | *WNT5B* | -4.688417319 | 1.33E-11 |
| ENSG00000147251 | *DOCK11* | -4.69222532 | 1.85E-06 |
| ENSG00000166823 | *MESP1* | -4.692335222 | 0.000593438 |
| ENSG00000055118 | *KCNH2* | -4.69328052 | 0.000990076 |
| ENSG00000170681 | *MURC* | -4.695627201 | 0.0309346 |
| ENSG00000105538 | *RASIP1* | -4.695659379 | 3.61E-07 |
| ENSG00000130653 | *PNPLA7* | -4.697484381 | 3.72E-05 |
| ENSG00000263934 | *SNORD3A* | -4.697786276 | 0.000101684 |
| ENSG00000162512 | *SDC3* | -4.698196979 | 8.65E-06 |
| ENSG00000105137 | *SYDE1* | -4.700331149 | 5.79E-05 |
| ENSG00000276533 | *RP11-139H15.5* | -4.703324137 | 0.040552063 |
| ENSG00000104043 | *ATP8B4* | -4.705860961 | 8.09E-05 |
| ENSG00000120693 | *SMAD9* | -4.706688364 | 3.29E-06 |
| ENSG00000223561 | *AC003090.1* | -4.707620132 | 0.003008547 |
| ENSG00000170500 | *LONRF2* | -4.722327795 | 2.80E-07 |
| ENSG00000164116 | *GUCY1A3* | -4.722471747 | 0.000164921 |
| ENSG00000049768 | *FOXP3* | -4.728719115 | 0.014473542 |
| ENSG00000255389 | *C6orf3* | -4.736633194 | 0.000853482 |
| ENSG00000116574 | *RHOU* | -4.747315322 | 6.69E-06 |
| ENSG00000184908 | *CLCNKB* | -4.749955101 | 0.013185964 |
| ENSG00000198208 | *RPS6KL1* | -4.75909151 | 1.13E-05 |
| ENSG00000188305 | *C19orf35* | -4.75954531 | 0.032434772 |
| ENSG00000137486 | *ARRB1* | -4.759703657 | 2.25E-06 |
| ENSG00000159167 | *STC1* | -4.766262138 | 0.001685056 |
| ENSG00000072818 | *ACAP1* | -4.776833116 | 1.50E-05 |
| ENSG00000167371 | *PRRT2* | -4.782004008 | 0.003845492 |
| ENSG00000138400 | *MDH1B* | -4.784617572 | 0.000289519 |
| ENSG00000179761 | *PIPOX* | -4.785773514 | 1.67E-05 |
| ENSG00000103056 | *SMPD3* | -4.787315812 | 2.29E-06 |
| ENSG00000109927 | *TECTA* | -4.790309409 | 0.000312959 |
| ENSG00000132185 | *FCRLA* | -4.793286883 | 0.036981115 |
| ENSG00000106069 | *CHN2* | -4.796397339 | 0.001356803 |
| ENSG00000250056 | *LINC01018* | -4.801123627 | 0.004107276 |
| ENSG00000183091 | *NEB* | -4.805198383 | 0.003475387 |
| ENSG00000178031 | *ADAMTSL1* | -4.806014028 | 2.48E-05 |
| ENSG00000107954 | *NEURL1* | -4.814967207 | 0.004083931 |
| ENSG00000170743 | *SYT9* | -4.81741897 | 0.047290916 |
| ENSG00000260230 | *FRRS1L* | -4.819543098 | 2.74E-05 |
| ENSG00000167100 | *SAMD14* | -4.820368112 | 0.00038531 |
| ENSG00000139193 | *CD27* | -4.820573384 | 0.002879643 |
| ENSG00000185482 | *STAC3* | -4.822058703 | 0.000716405 |
| ENSG00000149131 | *SERPING1* | -4.82523627 | 4.00E-06 |
| ENSG00000250328 | *MGC32805* | -4.825413853 | 0.043059879 |
| ENSG00000073712 | *FERMT2* | -4.829244198 | 1.03E-07 |
| ENSG00000214140 | *PRCD* | -4.834333388 | 0.000299359 |
| ENSG00000163219 | *ARHGAP25* | -4.834999254 | 0.000334755 |
| ENSG00000233170 | *RP11-863K10.4* | -4.83777094 | 0.028738316 |
| ENSG00000168062 | *BATF2* | -4.853632049 | 0.000287143 |
| ENSG00000267060 | *PTGES3L* | -4.863453355 | 0.03951258 |
| ENSG00000169442 | *CD52* | -4.870191545 | 0.000320615 |
| ENSG00000196092 | *PAX5* | -4.872821908 | 0.047485151 |
| ENSG00000013725 | *CD6* | -4.878001029 | 0.000385675 |
| ENSG00000233593 | *RP4-665J23.1* | -4.881016952 | 0.000140827 |
| ENSG00000185133 | *INPP5J* | -4.882115869 | 0.0015087 |
| ENSG00000117013 | *KCNQ4* | -4.884645829 | 9.84E-05 |
| ENSG00000079841 | *RIMS1* | -4.886920291 | 0.042408077 |
| ENSG00000273771 | *RP11-236L14.2* | -4.893902102 | 0.001820729 |
| ENSG00000196338 | *NLGN3* | -4.895746461 | 1.08E-06 |
| ENSG00000162415 | *ZSWIM5* | -4.898401156 | 9.24E-05 |
| ENSG00000112299 | *VNN1* | -4.904262428 | 0.02701742 |
| ENSG00000141431 | *ASXL3* | -4.906365224 | 0.001378124 |
| ENSG00000088882 | *CPXM1* | -4.907554141 | 2.14E-11 |
| ENSG00000204677 | *FAM153C* | -4.907569338 | 0.000385675 |
| ENSG00000133106 | *EPSTI1* | -4.907927197 | 2.81E-05 |
| ENSG00000138646 | *HERC5* | -4.909719577 | 1.57E-06 |
| ENSG00000261616 | *RP11-6O2.3* | -4.910362505 | 0.001329695 |
| ENSG00000225691 | *HLA-C* | -4.912675526 | 0.000368269 |
| ENSG00000132622 | *HSPA12B* | -4.921912353 | 1.88E-05 |
| ENSG00000188487 | *INSC* | -4.932953042 | 0.041698526 |
| ENSG00000157613 | *CREB3L1* | -4.934906438 | 0.000696581 |
| ENSG00000177990 | *DPY19L2* | -4.935519516 | 3.77E-10 |
| ENSG00000175463 | *TBC1D10C* | -4.941285961 | 0.00037953 |
| ENSG00000042832 | *TG* | -4.946319353 | 0.011300826 |
| ENSG00000158186 | *MRAS* | -4.950648212 | 2.18E-06 |
| ENSG00000272720 | *CTA-228A9.3* | -4.951318949 | 0.00419813 |
| ENSG00000042062 | *FAM65C* | -4.955627585 | 1.71E-08 |
| ENSG00000172476 | *RAB40A* | -4.956698852 | 0.002431266 |
| ENSG00000136404 | *TM6SF1* | -4.958305208 | 0.003475025 |
| ENSG00000125730 | *C3* | -4.96486451 | 9.98E-05 |
| ENSG00000254838 | *GVINP1* | -4.976339011 | 1.38E-05 |
| ENSG00000113532 | *ST8SIA4* | -4.979263083 | 0.005892133 |
| ENSG00000260577 | *RP11-615I2.2* | -4.98329415 | 0.004108973 |
| ENSG00000189134 | *NKAPL* | -4.983606142 | 0.027518218 |
| ENSG00000151623 | *NR3C2* | -4.989596535 | 0.000445299 |
| ENSG00000158104 | *HPD* | -4.990804734 | 0.002638087 |
| ENSG00000189292 | *FAM150B* | -4.998127519 | 0.00959712 |
| ENSG00000001561 | *ENPP4* | -5.00689191 | 8.77E-05 |
| ENSG00000133063 | *CHIT1* | -5.011489899 | 0.005071976 |
| ENSG00000155465 | *SLC7A7* | -5.012302479 | 2.59E-05 |
| ENSG00000214456 | *PLIN5* | -5.028785247 | 2.61E-05 |
| ENSG00000215452 | *ZNF663P* | -5.033533568 | 5.33E-05 |
| ENSG00000259495 | *RP11-210M15.2* | -5.034617686 | 0.022562657 |
| ENSG00000171291 | *ZNF439* | -5.035342216 | 2.95E-09 |
| ENSG00000167874 | *TMEM88* | -5.052785847 | 0.000227405 |
| ENSG00000172159 | *FRMD3* | -5.054832863 | 0.000653931 |
| ENSG00000197635 | *DPP4* | -5.060859887 | 0.000166842 |
| ENSG00000101977 | *MCF2* | -5.062996883 | 0.027147543 |
| ENSG00000262655 | *SPON1* | -5.065875986 | 1.51E-08 |
| ENSG00000077264 | *PAK3* | -5.069007517 | 0.000298249 |
| ENSG00000167680 | *SEMA6B* | -5.071561552 | 2.47E-05 |
| ENSG00000122176 | *FMOD* | -5.074899339 | 1.37E-12 |
| ENSG00000214189 | *ZNF788* | -5.076499992 | 0.000434656 |
| ENSG00000260911 | *RP11-196G11.2* | -5.081343554 | 0.020062322 |
| ENSG00000253250 | *C8orf88* | -5.081531194 | 0.000272906 |
| ENSG00000206450 | *HLA-B* | -5.085283776 | 9.72E-06 |
| ENSG00000215861 | *WI2-1896O14.1* | -5.087995889 | 0.001652092 |
| ENSG00000167311 | *ART5* | -5.088478485 | 0.020028357 |
| ENSG00000197405 | *C5AR1* | -5.097491945 | 7.94E-07 |
| ENSG00000255587 | *RAB44* | -5.097821375 | 0.027754912 |
| ENSG00000158163 | *DZIP1L* | -5.099134208 | 2.53E-07 |
| ENSG00000182771 | *GRID1* | -5.099603528 | 0.043698551 |
| ENSG00000233820 | *RP11-535M15.2* | -5.104292201 | 0.049349645 |
| ENSG00000091513 | *TF* | -5.108449749 | 5.13E-05 |
| ENSG00000152931 | *PART1* | -5.108759845 | 4.67E-06 |
| ENSG00000230082 | *PRRT3-AS1* | -5.110152268 | 0.026669765 |
| ENSG00000142661 | *MYOM3* | -5.111313836 | 0.016672073 |
| ENSG00000172602 | *RND1* | -5.119458358 | 0.002694943 |
| ENSG00000198771 | *RCSD1* | -5.120041319 | 2.90E-06 |
| ENSG00000182511 | *FES* | -5.120415957 | 2.09E-10 |
| ENSG00000109063 | *MYH3* | -5.123910615 | 0.000338107 |
| ENSG00000106125 | *FAM188B* | -5.126784718 | 0.008453474 |
| ENSG00000170989 | *S1PR1* | -5.130818911 | 6.93E-05 |
| ENSG00000116544 | *DLGAP3* | -5.130943735 | 0.023379384 |
| ENSG00000160963 | *COL26A1* | -5.132457847 | 0.006462757 |
| ENSG00000197614 | *MFAP5* | -5.133532968 | 0.001030368 |
| ENSG00000278934 | *CTD-2006M22.2* | -5.146433382 | 0.029021468 |
| ENSG00000178033 | *FAM26E* | -5.151847286 | 0.002808807 |
| ENSG00000261490 | *RP11-448G15.3* | -5.160632082 | 2.99E-07 |
| ENSG00000077616 | *NAALAD2* | -5.165524711 | 0.000239511 |
| ENSG00000225492 | *GBP1P1* | -5.170092994 | 5.27E-07 |
| ENSG00000162407 | *PPAP2B* | -5.171402401 | 4.19E-13 |
| ENSG00000163132 | *MSX1* | -5.172532675 | 0.000525288 |
| ENSG00000126217 | *MCF2L* | -5.175622936 | 0.0001001 |
| ENSG00000102802 | *MEDAG* | -5.177053347 | 0.001834079 |
| ENSG00000023902 | *PLEKHO1* | -5.178885752 | 1.32E-08 |
| ENSG00000273437 | *RP11-434H6.7* | -5.179005085 | 0.002489337 |
| ENSG00000126709 | *IFI6* | -5.181207823 | 1.05E-08 |
| ENSG00000129993 | *CBFA2T3* | -5.182154391 | 0.000585252 |
| ENSG00000129244 | *ATP1B2* | -5.190006727 | 0.000367785 |
| ENSG00000135454 | *B4GALNT1* | -5.191934474 | 2.83E-05 |
| ENSG00000126246 | *IGFLR1* | -5.202697291 | 0.015621773 |
| ENSG00000165917 | *RAPSN* | -5.203545847 | 0.025869004 |
| ENSG00000152495 | *CAMK4* | -5.205710166 | 8.99E-10 |
| ENSG00000119138 | *KLF9* | -5.206736534 | 1.11E-07 |
| ENSG00000181790 | *ADGRB1* | -5.209189363 | 0.001973477 |
| ENSG00000248144 | *ADH1C* | -5.210227113 | 0.001173613 |
| ENSG00000064201 | *TSPAN32* | -5.217713308 | 0.00105405 |
| ENSG00000010295 | *IFFO1* | -5.220887411 | 9.68E-10 |
| ENSG00000156127 | *BATF* | -5.2209781 | 0.000258837 |
| ENSG00000105419 | *MEIS3* | -5.228377769 | 7.27E-08 |
| ENSG00000246022 | *ALDH1L1-AS2* | -5.228876322 | 0.027014952 |
| ENSG00000205045 | *SLFN12L* | -5.228987999 | 0.047529147 |
| ENSG00000139914 | *FITM1* | -5.236420543 | 0.011470915 |
| ENSG00000168071 | *CCDC88B* | -5.236839123 | 1.87E-05 |
| ENSG00000232126 | *HLA-B* | -5.238572974 | 6.71E-06 |
| ENSG00000124749 | *COL21A1* | -5.242104677 | 7.85E-05 |
| ENSG00000170458 | *CD14* | -5.242318874 | 9.28E-08 |
| ENSG00000229056 | *AC020571.3* | -5.245696613 | 0.032768442 |
| ENSG00000170482 | *SLC23A1* | -5.250025128 | 0.020238856 |
| ENSG00000079931 | *MOXD1* | -5.263993105 | 7.15E-05 |
| ENSG00000183018 | *SPNS2* | -5.268387283 | 1.57E-05 |
| ENSG00000162576 | *MXRA8* | -5.269599541 | 1.59E-11 |
| ENSG00000122420 | *PTGFR* | -5.269775988 | 0.042118717 |
| ENSG00000105737 | *GRIK5* | -5.277418529 | 8.53E-07 |
| ENSG00000081041 | *CXCL2* | -5.278862795 | 0.000334953 |
| ENSG00000101082 | *SLA2* | -5.279209282 | 0.019600527 |
| ENSG00000106333 | *PCOLCE* | -5.292081507 | 3.65E-15 |
| ENSG00000159640 | *ACE* | -5.292101301 | 6.21E-05 |
| ENSG00000137573 | *SULF1* | -5.295908525 | 2.69E-08 |
| ENSG00000268089 | *GABRQ* | -5.299840695 | 0.030813953 |
| ENSG00000272511 | *RP11-180N14.1* | -5.301028936 | 0.028074527 |
| ENSG00000107551 | *RASSF4* | -5.305088009 | 2.44E-12 |
| ENSG00000086730 | *LAT2* | -5.308095151 | 8.76E-06 |
| ENSG00000116254 | *CHD5* | -5.310150616 | 0.001949177 |
| ENSG00000116741 | *RGS2* | -5.310598413 | 5.45E-08 |
| ENSG00000259969 | *RP11-999E24.3* | -5.310878901 | 0.001508223 |
| ENSG00000114200 | *BCHE* | -5.316634232 | 0.001504822 |
| ENSG00000158815 | *FGF17* | -5.3201769 | 0.000865912 |
| ENSG00000163131 | *CTSS* | -5.321873005 | 2.32E-08 |
| ENSG00000104899 | *AMH* | -5.327926056 | 0.024673667 |
| ENSG00000158473 | *CD1D* | -5.328984977 | 0.000205339 |
| ENSG00000272894 | *RP5-1159O4.1* | -5.330760442 | 1.15E-05 |
| ENSG00000117245 | *KIF17* | -5.332586433 | 0.00358847 |
| ENSG00000174099 | *MSRB3* | -5.334298359 | 2.78E-05 |
| ENSG00000145147 | *SLIT2* | -5.343851175 | 1.32E-07 |
| ENSG00000183873 | *SCN5A* | -5.344224616 | 0.006398969 |
| ENSG00000053918 | *KCNQ1* | -5.345810894 | 0.000385751 |
| ENSG00000272501 | *XXbac-BPG299F13.17* | -5.351617288 | 0.001847827 |
| ENSG00000110876 | *SELPLG* | -5.353047079 | 3.47E-06 |
| ENSG00000267121 | *CTD-2020K17.1* | -5.371587149 | 8.48E-05 |
| ENSG00000182853 | *VMO1* | -5.375771135 | 0.000153157 |
| ENSG00000060709 | *RIMBP2* | -5.378176351 | 0.013019081 |
| ENSG00000218336 | *TENM3* | -5.380228337 | 9.52E-06 |
| ENSG00000129467 | *ADCY4* | -5.383377898 | 3.32E-13 |
| ENSG00000188338 | *SLC38A3* | -5.38622004 | 0.014510997 |
| ENSG00000163661 | *PTX3* | -5.386758097 | 0.009543756 |
| ENSG00000198019 | *FCGR1B* | -5.389100496 | 0.019081927 |
| ENSG00000102962 | *CCL22* | -5.38914954 | 0.003955082 |
| ENSG00000143318 | *CASQ1* | -5.394957149 | 0.002021328 |
| ENSG00000146555 | *SDK1* | -5.395938991 | 0.000791264 |
| ENSG00000007237 | *GAS7* | -5.41200131 | 0.000858619 |
| ENSG00000131196 | *NFATC1* | -5.415258773 | 0.000187884 |
| ENSG00000095370 | *SH2D3C* | -5.416345086 | 9.31E-10 |
| ENSG00000176907 | *C8orf4* | -5.417630767 | 6.91E-08 |
| ENSG00000176919 | *C8G* | -5.418281332 | 0.042617878 |
| ENSG00000111664 | *GNB3* | -5.419851767 | 0.000320615 |
| ENSG00000162772 | *ATF3* | -5.423062626 | 7.15E-05 |
| ENSG00000167077 | *MEI1* | -5.427118898 | 0.000269853 |
| ENSG00000247774 | *PCED1B-AS1* | -5.43212931 | 0.000191138 |
| ENSG00000228214 | *LINC00693* | -5.440843477 | 0.010153842 |
| ENSG00000099994 | *SUSD2* | -5.444096461 | 4.60E-05 |
| ENSG00000161649 | *CD300LG* | -5.449920567 | 0.035485037 |
| ENSG00000259663 | *CTD-2314G24.2* | -5.462904038 | 1.73E-05 |
| ENSG00000131018 | *SYNE1* | -5.463879826 | 5.75E-17 |
| ENSG00000144488 | *ESPNL* | -5.465313616 | 0.002793469 |
| ENSG00000280153 | *RP11-876N24.3* | -5.467661966 | 1.16E-05 |
| ENSG00000115461 | *IGFBP5* | -5.468367007 | 1.72E-05 |
| ENSG00000148541 | *FAM13C* | -5.470004866 | 1.08E-05 |
| ENSG00000112183 | *RBM24* | -5.471801474 | 0.00038408 |
| ENSG00000157103 | *SLC6A1* | -5.474931271 | 0.027147543 |
| ENSG00000162654 | *GBP4* | -5.480766936 | 8.79E-06 |
| ENSG00000236532 | *AL035610.2* | -5.485715364 | 0.035737043 |
| ENSG00000172794 | *RAB37* | -5.486423374 | 8.56E-06 |
| ENSG00000100079 | *LGALS2* | -5.487318252 | 0.004965407 |
| ENSG00000165269 | *AQP7* | -5.494807138 | 0.035059998 |
| ENSG00000137673 | *MMP7* | -5.498455745 | 8.23E-07 |
| ENSG00000263400 | *TMEM220-AS1* | -5.499320519 | 0.006694677 |
| ENSG00000148408 | *CACNA1B* | -5.500627754 | 0.014130974 |
| ENSG00000166676 | *TVP23A* | -5.500708367 | 1.61E-06 |
| ENSG00000002745 | *WNT16* | -5.502294779 | 0.009074145 |
| ENSG00000142156 | *COL6A1* | -5.503663752 | 6.00E-19 |
| ENSG00000130558 | *OLFM1* | -5.507707571 | 3.74E-07 |
| ENSG00000204584 | *RP11-304F15.3* | -5.512940454 | 0.002965568 |
| ENSG00000090376 | *IRAK3* | -5.515259979 | 2.46E-07 |
| ENSG00000260805 | *RP11-61J19.5* | -5.519176411 | 0.002708803 |
| ENSG00000224608 | *HLA-B* | -5.522116774 | 3.38E-06 |
| ENSG00000160111 | *CPAMD8* | -5.52306666 | 1.08E-05 |
| ENSG00000049089 | *COL9A2* | -5.524545283 | 1.82E-05 |
| ENSG00000083067 | *TRPM3* | -5.524968327 | 0.011145122 |
| ENSG00000139910 | *NOVA1* | -5.526574568 | 6.38E-06 |
| ENSG00000116852 | *KIF21B* | -5.527982793 | 0.00406834 |
| ENSG00000094963 | *FMO2* | -5.531162591 | 0.007366941 |
| ENSG00000171476 | *HOPX* | -5.535188996 | 3.62E-08 |
| ENSG00000188404 | *SELL* | -5.537598925 | 0.000716405 |
| ENSG00000143127 | *ITGA10* | -5.537679545 | 1.26E-05 |
| ENSG00000080573 | *COL5A3* | -5.543053494 | 8.95E-09 |
| ENSG00000260077 | *RP11-254F7.2* | -5.548977332 | 9.23E-07 |
| ENSG00000139220 | *PPFIA2* | -5.553073823 | 0.000986183 |
| ENSG00000203685 | *C1orf95* | -5.557137715 | 6.87E-07 |
| ENSG00000171408 | *PDE7B* | -5.557580441 | 5.73E-05 |
| ENSG00000178752 | *FAM132B* | -5.559781416 | 0.001852102 |
| ENSG00000197536 | *C5orf56* | -5.561484141 | 6.74E-10 |
| ENSG00000110448 | *CD5* | -5.575850486 | 0.000587841 |
| ENSG00000153563 | *CD8A* | -5.579466626 | 9.40E-08 |
| ENSG00000046653 | *GPM6B* | -5.583840666 | 9.31E-06 |
| ENSG00000186417 | *GLDN* | -5.602283623 | 2.57E-05 |
| ENSG00000225746 | *SNHG23* | -5.602830667 | 0.012245721 |
| ENSG00000163687 | *DNASE1L3* | -5.606830606 | 0.004701357 |
| ENSG00000198300 | *PEG3* | -5.607254794 | 0.000113691 |
| ENSG00000197142 | *ACSL5* | -5.614098836 | 7.34E-07 |
| ENSG00000130303 | *BST2* | -5.616694995 | 3.46E-07 |
| ENSG00000104814 | *MAP4K1* | -5.618252914 | 2.14E-05 |
| ENSG00000158528 | *PPP1R9A* | -5.62374084 | 1.69E-07 |
| ENSG00000118292 | *C1orf54* | -5.628614547 | 5.37E-09 |
| ENSG00000022267 | *FHL1* | -5.630095125 | 4.73E-07 |
| ENSG00000177875 | *CCDC184* | -5.632699258 | 0.019852402 |
| ENSG00000067798 | *NAV3* | -5.632855135 | 9.82E-06 |
| ENSG00000124701 | *APOBEC2* | -5.638525688 | 0.011187014 |
| ENSG00000156414 | *TDRD9* | -5.639914186 | 0.048246323 |
| ENSG00000154655 | *L3MBTL4* | -5.645230898 | 4.15E-05 |
| ENSG00000125931 | *CITED1* | -5.647801762 | 0.005045127 |
| ENSG00000166341 | *DCHS1* | -5.658000908 | 2.11E-08 |
| ENSG00000100285 | *NEFH* | -5.66536943 | 0.000339338 |
| ENSG00000232907 | *DLGAP4-AS1* | -5.66649806 | 0.038726232 |
| ENSG00000205181 | *LINC00654* | -5.673835458 | 0.001414623 |
| ENSG00000278126 | *RP11-454E5.4* | -5.674570723 | 0.000166525 |
| ENSG00000036530 | *CYP46A1* | -5.680292196 | 0.002228154 |
| ENSG00000153234 | *NR4A2* | -5.684464925 | 2.19E-06 |
| ENSG00000126353 | *CCR7* | -5.686457976 | 0.002392897 |
| ENSG00000113924 | *HGD* | -5.69214092 | 0.000110245 |
| ENSG00000118777 | *ABCG2* | -5.695572336 | 1.25E-05 |
| ENSG00000250508 | *RP11-757G1.6* | -5.697965211 | 0.034794939 |
| ENSG00000144596 | *GRIP2* | -5.702130666 | 0.000189358 |
| ENSG00000144810 | *COL8A1* | -5.703066954 | 3.09E-05 |
| ENSG00000255150 | *EID3* | -5.703764371 | 9.37E-05 |
| ENSG00000087076 | *HSD17B14* | -5.706138857 | 7.40E-12 |
| ENSG00000233038 | *AC011899.9* | -5.708480067 | 0.03120268 |
| ENSG00000122035 | *RASL11A* | -5.709770298 | 1.84E-09 |
| ENSG00000197043 | *ANXA6* | -5.710308231 | 6.21E-10 |
| ENSG00000241112 | *RPL29P14* | -5.713597403 | 0.044963687 |
| ENSG00000115252 | *PDE1A* | -5.729270835 | 0.000153157 |
| ENSG00000267640 | *CTD-2554C21.2* | -5.732173391 | 0.009494684 |
| ENSG00000111913 | *FAM65B* | -5.733371184 | 6.79E-11 |
| ENSG00000215217 | *C5orf49* | -5.738523383 | 0.00077567 |
| ENSG00000168772 | *CXXC4* | -5.7409527 | 0.041880945 |
| ENSG00000234840 | *LINC01239* | -5.743883806 | 0.013345012 |
| ENSG00000160321 | *ZNF208* | -5.746453066 | 0.008062364 |
| ENSG00000198286 | *CARD11* | -5.748556916 | 0.001105304 |
| ENSG00000140795 | *MYLK3* | -5.749897731 | 0.017512113 |
| ENSG00000124019 | *FAM124B* | -5.751807287 | 0.022904732 |
| ENSG00000111729 | *CLEC4A* | -5.759128829 | 0.000153157 |
| ENSG00000171016 | *PYGO1* | -5.761071859 | 1.25E-06 |
| ENSG00000108176 | *DNAJC12* | -5.76584585 | 0.000422948 |
| ENSG00000184785 | *SMIM10* | -5.769893956 | 3.13E-07 |
| ENSG00000113319 | *RASGRF2* | -5.772572399 | 7.68E-11 |
| ENSG00000138722 | *MMRN1* | -5.781498387 | 0.000537028 |
| ENSG00000182676 | *PPP1R27* | -5.783187671 | 0.002542617 |
| ENSG00000213514 | *RP11-428P16.2* | -5.789308774 | 0.035179545 |
| ENSG00000102935 | *ZNF423* | -5.789758752 | 5.84E-07 |
| ENSG00000152270 | *PDE3B* | -5.793292526 | 1.72E-07 |
| ENSG00000104894 | *CD37* | -5.798089265 | 1.37E-05 |
| ENSG00000146151 | *HMGCLL1* | -5.800846741 | 0.002201398 |
| ENSG00000117115 | *PADI2* | -5.814287306 | 0.001420391 |
| ENSG00000167483 | *FAM129C* | -5.816786515 | 0.002800951 |
| ENSG00000236699 | *ARHGEF38* | -5.817273405 | 0.001463642 |
| ENSG00000134533 | *RERG* | -5.820387236 | 2.24E-10 |
| ENSG00000164591 | *MYOZ3* | -5.828548062 | 0.000632555 |
| ENSG00000157766 | *ACAN* | -5.836760519 | 0.044502101 |
| ENSG00000258647 | *LINC00930* | -5.839858039 | 0.020263269 |
| ENSG00000228784 | *LINC00954* | -5.843237395 | 0.002112898 |
| ENSG00000156689 | *GLYATL2* | -5.845484312 | 0.015602565 |
| ENSG00000196684 | *HSH2D* | -5.85107171 | 0.000867949 |
| ENSG00000179362 | *HMGN2P46* | -5.867980948 | 7.31E-06 |
| ENSG00000112041 | *TULP1* | -5.871437006 | 0.038772203 |
| ENSG00000159403 | *C1R* | -5.87149603 | 1.92E-17 |
| ENSG00000267082 | *CTC-510F12.2* | -5.87562969 | 5.14E-05 |
| ENSG00000128284 | *APOL3* | -5.87791482 | 3.61E-12 |
| ENSG00000259158 | *ADAM20P1* | -5.888934438 | 0.028465853 |
| ENSG00000154556 | *SORBS2* | -5.894250991 | 6.26E-11 |
| ENSG00000164318 | *EGFLAM* | -5.894660992 | 0.000625212 |
| ENSG00000127903 | *ZNF835* | -5.894846124 | 0.012692747 |
| ENSG00000250138 | *RP11-848G14.5* | -5.899550992 | 0.003699119 |
| ENSG00000064886 | *CHI3L2* | -5.908980555 | 0.000202061 |
| ENSG00000169116 | *PARM1* | -5.909490089 | 1.01E-06 |
| ENSG00000138135 | *CH25H* | -5.911606459 | 0.000906873 |
| ENSG00000255986 | *MT1JP* | -5.912458197 | 0.003247976 |
| ENSG00000136859 | *ANGPTL2* | -5.914162412 | 2.81E-20 |
| ENSG00000272168 | *CASC15* | -5.920500155 | 0.000239708 |
| ENSG00000196358 | *NTNG2* | -5.929923622 | 3.54E-05 |
| ENSG00000161929 | *SCIMP* | -5.945403861 | 0.002309125 |
| ENSG00000143110 | *C1orf162* | -5.948504785 | 3.24E-08 |
| ENSG00000165349 | *SLC7A3* | -5.955202632 | 0.0359869 |
| ENSG00000168913 | *ENHO* | -5.957714359 | 0.046162271 |
| ENSG00000081818 | *PCDHB4* | -5.962034688 | 0.00036419 |
| ENSG00000165071 | *TMEM71* | -5.973149768 | 0.002059476 |
| ENSG00000253636 | *RP11-531A24.5* | -5.974213428 | 0.000689789 |
| ENSG00000143387 | *CTSK* | -5.976595243 | 1.41E-13 |
| ENSG00000253379 | *RP11-1102P16.1* | -5.978236368 | 0.042087657 |
| ENSG00000180539 | *C9orf139* | -5.984173676 | 0.019307614 |
| ENSG00000174611 | *KY* | -5.987896101 | 0.011734004 |
| ENSG00000169026 | *MFSD7* | -5.989641367 | 1.47E-05 |
| ENSG00000164161 | *HHIP* | -6.017992898 | 1.38E-05 |
| ENSG00000128596 | *CCDC136* | -6.018761948 | 1.57E-10 |
| ENSG00000132530 | *XAF1* | -6.021875858 | 4.82E-05 |
| ENSG00000184838 | *PRR16* | -6.023309714 | 1.77E-06 |
| ENSG00000108387 | *4-Sep* | -6.026156621 | 1.82E-05 |
| ENSG00000167207 | *NOD2* | -6.026739461 | 0.011541412 |
| ENSG00000099954 | *CECR2* | -6.04577018 | 0.010417609 |
| ENSG00000117594 | *HSD11B1* | -6.045825655 | 1.69E-06 |
| ENSG00000128266 | *GNAZ* | -6.046089749 | 9.97E-08 |
| ENSG00000125869 | *LAMP5* | -6.046189032 | 0.02995588 |
| ENSG00000110852 | *CLEC2B* | -6.049380375 | 3.81E-12 |
| ENSG00000152213 | *ARL11* | -6.057093748 | 0.00070746 |
| ENSG00000266714 | *MYO15B* | -6.059866161 | 8.43E-11 |
| ENSG00000168497 | *SDPR* | -6.060586839 | 1.70E-08 |
| ENSG00000130822 | *PNCK* | -6.066071522 | 2.97E-17 |
| ENSG00000168004 | *HRASLS5* | -6.066178785 | 0.011681887 |
| ENSG00000075340 | *ADD2* | -6.067191263 | 0.037637792 |
| ENSG00000179583 | *CIITA* | -6.075048776 | 3.56E-06 |
| ENSG00000108381 | *ASPA* | -6.076447367 | 1.80E-07 |
| ENSG00000275830 | *RP11-403A3.3* | -6.079077976 | 0.048358726 |
| ENSG00000126264 | *HCST* | -6.082387288 | 0.000186557 |
| ENSG00000027869 | *SH2D2A* | -6.087643372 | 3.19E-05 |
| ENSG00000075073 | *TACR2* | -6.088165305 | 4.95E-05 |
| ENSG00000120129 | *DUSP1* | -6.089369474 | 3.20E-06 |
| ENSG00000167895 | *TMC8* | -6.089962491 | 5.34E-07 |
| ENSG00000265142 | *MIR133A1HG* | -6.099240053 | 0.013386888 |
| ENSG00000157502 | *MUM1L1* | -6.100730308 | 0.000588271 |
| ENSG00000122694 | *GLIPR2* | -6.101720748 | 8.25E-09 |
| ENSG00000278743 | *RP11-707G18.1* | -6.106265747 | 0.048911089 |
| ENSG00000121797 | *CCRL2* | -6.106415506 | 0.014645651 |
| ENSG00000074370 | *ATP2A3* | -6.126159045 | 1.72E-05 |
| ENSG00000159212 | *CLIC6* | -6.127968895 | 1.41E-07 |
| ENSG00000108821 | *COL1A1* | -6.129742673 | 9.07E-13 |
| ENSG00000188906 | *LRRK2* | -6.13082276 | 1.25E-09 |
| ENSG00000154262 | *ABCA6* | -6.133078053 | 2.08E-05 |
| ENSG00000020633 | *RUNX3* | -6.133303519 | 9.14E-05 |
| ENSG00000141314 | *RHBDL3* | -6.13404369 | 0.002586316 |
| ENSG00000168621 | *GDNF* | -6.135857892 | 0.006824898 |
| ENSG00000131386 | *GALNT15* | -6.143836978 | 0.002069176 |
| ENSG00000274536 | *RP6-159A1.4* | -6.1518504 | 0.024336106 |
| ENSG00000178162 | *FAR2P2* | -6.15547554 | 7.96E-07 |
| ENSG00000145861 | *C1QTNF2* | -6.162591117 | 0.000134483 |
| ENSG00000108001 | *EBF3* | -6.162749144 | 0.011401145 |
| ENSG00000187260 | *WDR86* | -6.164484066 | 0.001027661 |
| ENSG00000177791 | *MYOZ1* | -6.171093866 | 0.000371895 |
| ENSG00000182132 | *KCNIP1* | -6.172530182 | 0.036235051 |
| ENSG00000254427 | *RP11-430H10.1* | -6.172572817 | 0.037546091 |
| ENSG00000231920 | *NEBL-AS1* | -6.172954765 | 0.025183909 |
| ENSG00000150627 | *WDR17* | -6.174617151 | 0.000506891 |
| ENSG00000041515 | *MYO16* | -6.174659658 | 0.00837779 |
| ENSG00000169085 | *C8orf46* | -6.175134137 | 1.29E-09 |
| ENSG00000059804 | *SLC2A3* | -6.180035891 | 1.07E-09 |
| ENSG00000206452 | *HLA-C* | -6.181746957 | 0.000954692 |
| ENSG00000153976 | *HS3ST3A1* | -6.18560652 | 3.19E-06 |
| ENSG00000015285 | *WAS* | -6.187066312 | 2.42E-06 |
| ENSG00000197361 | *FBXL22* | -6.19090027 | 6.32E-12 |
| ENSG00000123570 | *RAB9B* | -6.193246434 | 5.09E-08 |
| ENSG00000056487 | *PHF21B* | -6.194528831 | 0.000827956 |
| ENSG00000213706 | *AL590762.7* | -6.195368938 | 0.030544769 |
| ENSG00000156076 | *WIF1* | -6.201095753 | 0.005436167 |
| ENSG00000136235 | *GPNMB* | -6.203454867 | 3.10E-16 |
| ENSG00000172985 | *SH3RF3* | -6.205614908 | 5.12E-12 |
| ENSG00000164949 | *GEM* | -6.208817523 | 1.35E-05 |
| ENSG00000147255 | *IGSF1* | -6.214684446 | 0.008265674 |
| ENSG00000166825 | *ANPEP* | -6.219363969 | 1.69E-10 |
| ENSG00000143248 | *RGS5* | -6.220032927 | 6.64E-14 |
| ENSG00000186479 | *RGS7BP* | -6.229063065 | 0.000534385 |
| ENSG00000128253 | *RFPL2* | -6.231275338 | 0.024956918 |
| ENSG00000261560 | *RP11-166B2.3* | -6.234828598 | 0.026492449 |
| ENSG00000185905 | *C16orf54* | -6.234948169 | 0.003193729 |
| ENSG00000069535 | *MAOB* | -6.234996352 | 8.22E-11 |
| ENSG00000173110 | *HSPA6* | -6.240468139 | 1.25E-05 |
| ENSG00000162896 | *PIGR* | -6.244841504 | 0.000146455 |
| ENSG00000273483 | *RP4-671G15.2* | -6.248550283 | 0.049603858 |
| ENSG00000211772 | *TRBC2* | -6.249013767 | 0.036235051 |
| ENSG00000134545 | *KLRC1* | -6.249382069 | 0.029226538 |
| ENSG00000141338 | *ABCA8* | -6.257423366 | 1.21E-12 |
| ENSG00000280339 | *RP11-736K20.4* | -6.265575506 | 0.010832387 |
| ENSG00000023445 | *BIRC3* | -6.274810631 | 2.87E-10 |
| ENSG00000132688 | *NES* | -6.2792059 | 8.06E-10 |
| ENSG00000123119 | *NECAB1* | -6.280885767 | 4.32E-05 |
| ENSG00000196169 | *KIF19* | -6.281248609 | 0.049965799 |
| ENSG00000119535 | *CSF3R* | -6.28788978 | 1.53E-05 |
| ENSG00000108950 | *FAM20A* | -6.288727404 | 1.37E-05 |
| ENSG00000165810 | *BTNL9* | -6.291171915 | 0.000224874 |
| ENSG00000130294 | *KIF1A* | -6.298895192 | 0.003794081 |
| ENSG00000254507 | *RP11-481A20.10* | -6.300876788 | 0.046544969 |
| ENSG00000077274 | *CAPN6* | -6.302874818 | 5.11E-06 |
| ENSG00000164128 | *NPY1R* | -6.303201824 | 0.01979817 |
| ENSG00000139055 | *ERP27* | -6.304472946 | 0.000283244 |
| ENSG00000182492 | *BGN* | -6.310395304 | 1.64E-10 |
| ENSG00000139970 | *RTN1* | -6.310835806 | 5.76E-06 |
| ENSG00000215386 | *MIR99AHG* | -6.311865795 | 3.98E-10 |
| ENSG00000108515 | *ENO3* | -6.315212658 | 4.77E-07 |
| ENSG00000188511 | *C22orf34* | -6.320516002 | 0.039423087 |
| ENSG00000160469 | *BRSK1* | -6.328342271 | 4.85E-07 |
| ENSG00000073910 | *FRY* | -6.328370806 | 1.24E-07 |
| ENSG00000040731 | *CDH10* | -6.328673483 | 0.00223987 |
| ENSG00000169282 | *KCNAB1* | -6.343985652 | 3.22E-18 |
| ENSG00000171954 | *CYP4F22* | -6.345272126 | 0.039781267 |
| ENSG00000155966 | *AFF2* | -6.345832335 | 0.048050531 |
| ENSG00000230018 | *RP11-481H12.1* | -6.34955202 | 0.022054431 |
| ENSG00000170369 | *CST2* | -6.357770738 | 0.04075514 |
| ENSG00000144668 | *ITGA9* | -6.360606059 | 1.78E-15 |
| ENSG00000260249 | *RP11-401P9.5* | -6.364702276 | 0.047790252 |
| ENSG00000158560 | *DYNC1I1* | -6.370076852 | 1.98E-08 |
| ENSG00000169783 | *LINGO1* | -6.377018215 | 0.016182111 |
| ENSG00000165178 | *NCF1C* | -6.380197431 | 0.000299673 |
| ENSG00000100678 | *SLC8A3* | -6.383492332 | 0.048776777 |
| ENSG00000166147 | *FBN1* | -6.384133339 | 3.44E-09 |
| ENSG00000062524 | *LTK* | -6.389243979 | 0.000508535 |
| ENSG00000006638 | *TBXA2R* | -6.392632097 | 0.000149106 |
| ENSG00000142102 | *ATHL1* | -6.393131518 | 8.47E-14 |
| ENSG00000204335 | *SP5* | -6.39395478 | 0.008620021 |
| ENSG00000110799 | *VWF* | -6.41173678 | 7.14E-11 |
| ENSG00000126895 | *AVPR2* | -6.412206675 | 0.02589662 |
| ENSG00000113212 | *PCDHB7* | -6.420632781 | 0.002952204 |
| ENSG00000160191 | *PDE9A* | -6.421471692 | 4.09E-08 |
| ENSG00000239975 | *IGKV1D-33* | -6.425851185 | 0.022660543 |
| ENSG00000123685 | *BATF3* | -6.429842041 | 0.000137884 |
| ENSG00000175356 | *SCUBE2* | -6.437025576 | 2.18E-08 |
| ENSG00000066629 | *EML1* | -6.437417054 | 6.41E-12 |
| ENSG00000089820 | *ARHGAP4* | -6.441485208 | 2.92E-09 |
| ENSG00000275325 | *PDCD6IPP1* | -6.447459011 | 0.027822351 |
| ENSG00000168702 | *LRP1B* | -6.451769541 | 0.000151226 |
| ENSG00000180353 | *HCLS1* | -6.454376408 | 1.62E-08 |
| ENSG00000196569 | *LAMA2* | -6.455269235 | 2.13E-13 |
| ENSG00000162543 | *UBXN10* | -6.457983002 | 5.77E-06 |
| ENSG00000166741 | *NNMT* | -6.459059675 | 0.011581905 |
| ENSG00000123572 | *NRK* | -6.460706028 | 7.89E-05 |
| ENSG00000189409 | *MMP23B* | -6.472794176 | 4.86E-05 |
| ENSG00000163710 | *PCOLCE2* | -6.473668488 | 7.45E-07 |
| ENSG00000164440 | *TXLNB* | -6.481528826 | 0.000345528 |
| ENSG00000171502 | *COL24A1* | -6.486809319 | 0.000605804 |
| ENSG00000228157 | *AC007952.5* | -6.493706119 | 0.021294346 |
| ENSG00000111879 | *FAM184A* | -6.497784679 | 1.08E-06 |
| ENSG00000106038 | *EVX1* | -6.498357303 | 0.000228121 |
| ENSG00000235997 | *AC109642.1* | -6.498672434 | 0.023999645 |
| ENSG00000161381 | *PLXDC1* | -6.502487111 | 2.73E-11 |
| ENSG00000243649 | *CFB* | -6.504022772 | 0.011662148 |
| ENSG00000162892 | *IL24* | -6.504045047 | 0.007745467 |
| ENSG00000102245 | *CD40LG* | -6.504725971 | 0.028943497 |
| ENSG00000187699 | *C2orf88* | -6.517097025 | 4.88E-10 |
| ENSG00000128917 | *DLL4* | -6.523670953 | 5.18E-08 |
| ENSG00000163873 | *GRIK3* | -6.526438423 | 0.015990708 |
| ENSG00000177238 | *TRIM72* | -6.527526902 | 0.01077012 |
| ENSG00000087250 | *MT3* | -6.53121559 | 0.010725122 |
| ENSG00000224397 | *LINC01272* | -6.536275331 | 0.006073355 |
| ENSG00000215018 | *COL28A1* | -6.537416799 | 0.001509138 |
| ENSG00000181074 | *OR52N4* | -6.537635322 | 0.029933584 |
| ENSG00000259834 | *RP11-284N8.3* | -6.53901103 | 0.017867926 |
| ENSG00000141449 | *GREB1L* | -6.541846187 | 3.31E-05 |
| ENSG00000166448 | *TMEM130* | -6.541851307 | 0.00172781 |
| ENSG00000169894 | *MUC3A* | -6.54838559 | 4.00E-05 |
| ENSG00000279805 | *CTA-212A2.1* | -6.554664794 | 0.020615677 |
| ENSG00000172243 | *CLEC7A* | -6.556471467 | 4.86E-05 |
| ENSG00000065534 | *MYLK* | -6.563801197 | 1.64E-09 |
| ENSG00000149633 | *KIAA1755* | -6.56431093 | 2.20E-09 |
| ENSG00000244306 | *LINC01296* | -6.576429457 | 0.029337932 |
| ENSG00000165995 | *CACNB2* | -6.582779897 | 7.60E-11 |
| ENSG00000149150 | *SLC43A1* | -6.587409029 | 2.05E-09 |
| ENSG00000159337 | *PLA2G4D* | -6.590718074 | 0.00164979 |
| ENSG00000260816 | *RP11-319G9.3* | -6.592164173 | 0.005400515 |
| ENSG00000240505 | *TNFRSF13B* | -6.595756986 | 0.029942344 |
| ENSG00000256262 | *USP30-AS1* | -6.595944722 | 0.02852191 |
| ENSG00000181143 | *MUC16* | -6.596777903 | 0.022901492 |
| ENSG00000277734 | *TRAC* | -6.603284468 | 4.11E-06 |
| ENSG00000182575 | *NXPH3* | -6.607845299 | 1.21E-08 |
| ENSG00000248801 | *C8orf34-AS1* | -6.610059362 | 0.043946465 |
| ENSG00000260244 | *RP11-588K22.2* | -6.610984036 | 1.28E-07 |
| ENSG00000148948 | *LRRC4C* | -6.611521573 | 0.029620059 |
| ENSG00000110680 | *CALCA* | -6.612438985 | 0.02201631 |
| ENSG00000196711 | *FAM150A* | -6.617643927 | 0.019334166 |
| ENSG00000142149 | *HUNK* | -6.624330887 | 0.000296094 |
| ENSG00000139572 | *GPR84* | -6.626301679 | 0.024271723 |
| ENSG00000226180 | *RP11-278A23.1* | -6.62646592 | 0.03951258 |
| ENSG00000184730 | *APOBR* | -6.628003946 | 3.10E-05 |
| ENSG00000163083 | *INHBB* | -6.629197681 | 0.001938431 |
| ENSG00000179256 | *SMCO3* | -6.631937803 | 0.019912781 |
| ENSG00000131097 | *HIGD1B* | -6.639117349 | 0.003696588 |
| ENSG00000076344 | *RGS11* | -6.643314403 | 2.87E-12 |
| ENSG00000266378 | *RP11-214O1.3* | -6.649632217 | 0.038584061 |
| ENSG00000074047 | *GLI2* | -6.658076936 | 8.16E-07 |
| ENSG00000258314 | *CTD-2314B22.1* | -6.658421111 | 0.02024768 |
| ENSG00000162241 | *SLC25A45* | -6.671403866 | 2.74E-07 |
| ENSG00000266964 | *FXYD1* | -6.672605279 | 0.013924971 |
| ENSG00000185532 | *PRKG1* | -6.676114778 | 8.77E-10 |
| ENSG00000131401 | *NAPSB* | -6.677316659 | 1.31E-05 |
| ENSG00000255026 | *RP11-326C3.2* | -6.681896979 | 0.003206987 |
| ENSG00000173918 | *C1QTNF1* | -6.689039042 | 1.38E-10 |
| ENSG00000177839 | *PCDHB9* | -6.695727494 | 0.013884545 |
| ENSG00000143512 | *HHIPL2* | -6.696752895 | 0.027178135 |
| ENSG00000269516 | *CYP4F23P* | -6.698217251 | 0.016576506 |
| ENSG00000170370 | *EMX2* | -6.698377678 | 0.035013984 |
| ENSG00000065413 | *ANKRD44* | -6.698752017 | 2.01E-10 |
| ENSG00000005379 | *BZRAP1* | -6.699611533 | 1.13E-10 |
| ENSG00000102837 | *OLFM4* | -6.699890212 | 0.022904732 |
| ENSG00000173421 | *CCDC36* | -6.703033688 | 0.014663859 |
| ENSG00000100985 | *MMP9* | -6.7047056 | 0.021483107 |
| ENSG00000018280 | *SLC11A1* | -6.706827704 | 8.39E-10 |
| ENSG00000151692 | *RNF144A* | -6.707236529 | 3.90E-10 |
| ENSG00000250510 | *GPR162* | -6.709190352 | 9.93E-06 |
| ENSG00000087258 | *GNAO1* | -6.727300538 | 2.62E-06 |
| ENSG00000270640 | *RP11-373D23.2* | -6.727851314 | 0.011475312 |
| ENSG00000096088 | *PGC* | -6.729802601 | 0.004360119 |
| ENSG00000131730 | *CKMT2* | -6.730062964 | 2.52E-06 |
| ENSG00000277496 | *RP11-93B14.9* | -6.731904658 | 0.003760662 |
| ENSG00000235688 | *AC116614.1* | -6.732940964 | 0.030306315 |
| ENSG00000108551 | *RASD1* | -6.741313133 | 0.011177136 |
| ENSG00000154736 | *ADAMTS5* | -6.743808284 | 1.24E-06 |
| ENSG00000196104 | *SPOCK3* | -6.743962327 | 1.10E-10 |
| ENSG00000134321 | *RSAD2* | -6.74748273 | 1.59E-05 |
| ENSG00000141837 | *CACNA1A* | -6.753884118 | 0.006070811 |
| ENSG00000127533 | *F2RL3* | -6.755263924 | 0.024309856 |
| ENSG00000131634 | *TMEM204* | -6.756597714 | 2.24E-10 |
| ENSG00000162711 | *NLRP3* | -6.75761247 | 7.96E-05 |
| ENSG00000138615 | *CILP* | -6.759331265 | 4.06E-05 |
| ENSG00000165495 | *PKNOX2* | -6.75990175 | 1.78E-08 |
| ENSG00000146215 | *CRIP3* | -6.760537663 | 9.73E-06 |
| ENSG00000159753 | *RLTPR* | -6.76308469 | 7.02E-05 |
| ENSG00000120903 | *CHRNA2* | -6.765288156 | 0.012990092 |
| ENSG00000142552 | *RCN3* | -6.766715496 | 1.95E-17 |
| ENSG00000224729 | *PCOLCE-AS1* | -6.76959028 | 0.021102099 |
| ENSG00000162373 | *BEND5* | -6.772017878 | 2.91E-06 |
| ENSG00000227136 | *LINC00595* | -6.774488332 | 0.004717307 |
| ENSG00000246375 | *RP11-10L7.1* | -6.776507749 | 0.032750669 |
| ENSG00000140092 | *FBLN5* | -6.777539125 | 4.56E-09 |
| ENSG00000229619 | *MBNL1-AS1* | -6.790566911 | 4.58E-09 |
| ENSG00000273733 | *CTC-510F12.7* | -6.790774739 | 0.000523147 |
| ENSG00000080007 | *DDX43* | -6.791651046 | 0.041842011 |
| ENSG00000121769 | *FABP3* | -6.80050329 | 5.67E-12 |
| ENSG00000152785 | *BMP3* | -6.804928037 | 0.008749519 |
| ENSG00000144847 | *IGSF11* | -6.805007959 | 0.030327998 |
| ENSG00000152760 | *TCTEX1D1* | -6.80583668 | 0.040488538 |
| ENSG00000178175 | *ZNF366* | -6.805985796 | 0.015818624 |
| ENSG00000163508 | *EOMES* | -6.806461056 | 0.01240885 |
| ENSG00000125910 | *S1PR4* | -6.807467264 | 0.000139593 |
| ENSG00000157680 | *DGKI* | -6.812087829 | 0.036235051 |
| ENSG00000019582 | *CD74* | -6.812198813 | 2.62E-13 |
| ENSG00000152207 | *CYSLTR2* | -6.819968169 | 0.012323077 |
| ENSG00000188916 | *FAM196A* | -6.823563944 | 0.011395441 |
| ENSG00000157782 | *CABP1* | -6.825690357 | 0.001157478 |
| ENSG00000156113 | *KCNMA1* | -6.827743919 | 8.10E-28 |
| ENSG00000251573 | *CTD-2089N3.2* | -6.83231564 | 0.041517866 |
| ENSG00000205362 | *MT1A* | -6.833211049 | 5.33E-06 |
| ENSG00000169507 | *SLC38A11* | -6.834164772 | 2.36E-06 |
| ENSG00000254415 | *SIGLEC14* | -6.83477867 | 0.003886821 |
| ENSG00000278301 | *GRAMD4P3* | -6.838772092 | 0.017422878 |
| ENSG00000165215 | *CLDN3* | -6.839702407 | 0.01734436 |
| ENSG00000087085 | *ACHE* | -6.840876442 | 8.62E-07 |
| ENSG00000111796 | *KLRB1* | -6.845355488 | 2.61E-05 |
| ENSG00000069122 | *ADGRF5* | -6.845652729 | 9.74E-08 |
| ENSG00000143028 | *SYPL2* | -6.845817614 | 9.73E-06 |
| ENSG00000100884 | *CPNE6* | -6.845982034 | 0.001030235 |
| ENSG00000122025 | *FLT3* | -6.847014793 | 0.007167398 |
| ENSG00000183317 | *EPHA10* | -6.854312324 | 0.016200828 |
| ENSG00000225756 | *DBH-AS1* | -6.858094344 | 0.012496129 |
| ENSG00000160013 | *PTGIR* | -6.859892295 | 0.01826813 |
| ENSG00000188848 | *BEND4* | -6.859926393 | 0.012572114 |
| ENSG00000164691 | *TAGAP* | -6.871806659 | 2.04E-05 |
| ENSG00000007314 | *SCN4A* | -6.878629051 | 0.014846717 |
| ENSG00000116329 | *OPRD1* | -6.878882577 | 0.049056302 |
| ENSG00000268555 | *RP11-678G14.3* | -6.878992268 | 0.045385308 |
| ENSG00000204851 | *PNMAL2* | -6.886050162 | 0.00163552 |
| ENSG00000079112 | *CDH17* | -6.886712919 | 0.029246942 |
| ENSG00000175229 | *GAL3ST3* | -6.893065659 | 0.010965678 |
| ENSG00000237499 | *RP11-356I2.4* | -6.893294921 | 0.003779455 |
| ENSG00000245812 | *RP11-175K6.1* | -6.894340261 | 0.035417217 |
| ENSG00000187922 | *LCN10* | -6.898098135 | 0.005573495 |
| ENSG00000152689 | *RASGRP3* | -6.902127625 | 5.85E-07 |
| ENSG00000267506 | *RP11-13K12.1* | -6.910917104 | 0.047220151 |
| ENSG00000154654 | *NCAM2* | -6.911082878 | 0.003451846 |
| ENSG00000143185 | *XCL2* | -6.91208181 | 0.029193053 |
| ENSG00000172156 | *CCL11* | -6.912990065 | 0.025496375 |
| ENSG00000160097 | *FNDC5* | -6.91322576 | 2.76E-07 |
| ENSG00000148677 | *ANKRD1* | -6.917487004 | 0.033590656 |
| ENSG00000182230 | *FAM153B* | -6.920884214 | 1.19E-10 |
| ENSG00000171659 | *GPR34* | -6.921374573 | 0.000119776 |
| ENSG00000255007 | *CTD-2589M5.4* | -6.924966924 | 0.013370868 |
| ENSG00000145708 | *CRHBP* | -6.926249646 | 0.029458227 |
| ENSG00000172260 | *NEGR1* | -6.927609914 | 3.34E-07 |
| ENSG00000234290 | *AC116366.6* | -6.928411809 | 0.011223137 |
| ENSG00000113248 | *PCDHB15* | -6.931023636 | 6.54E-06 |
| ENSG00000135077 | *HAVCR2* | -6.935948553 | 7.65E-08 |
| ENSG00000167851 | *CD300A* | -6.93598266 | 4.59E-08 |
| ENSG00000109846 | *CRYAB* | -6.936880392 | 1.05E-07 |
| ENSG00000253405 | *EVX1-AS* | -6.940297947 | 0.009443176 |
| ENSG00000149403 | *GRIK4* | -6.944949971 | 0.020224806 |
| ENSG00000204103 | *MAFB* | -6.947575074 | 3.71E-06 |
| ENSG00000166501 | *PRKCB* | -6.951624362 | 5.12E-08 |
| ENSG00000150893 | *FREM2* | -6.955085253 | 0.001582136 |
| ENSG00000259881 | *RP11-830F9.5* | -6.956618423 | 0.003307996 |
| ENSG00000245025 | *RP11-875O11.1* | -6.957627953 | 0.020524161 |
| ENSG00000123360 | *PDE1B* | -6.960264551 | 0.001551529 |
| ENSG00000134571 | *MYBPC3* | -6.962915574 | 0.043698551 |
| ENSG00000130222 | *GADD45G* | -6.963393554 | 2.11E-07 |
| ENSG00000259863 | *SH3RF3-AS1* | -6.964744834 | 0.010484951 |
| ENSG00000186265 | *BTLA* | -6.964819591 | 0.009966814 |
| ENSG00000143171 | *RXRG* | -6.965280531 | 0.034970235 |
| ENSG00000171777 | *RASGRP4* | -6.966984706 | 0.003089674 |
| ENSG00000142611 | *PRDM16* | -6.969576958 | 0.00929931 |
| ENSG00000184293 | *CLECL1* | -6.970243429 | 0.021483107 |
| ENSG00000091879 | *ANGPT2* | -6.971143795 | 0.006458568 |
| ENSG00000167994 | *RAB3IL1* | -6.973064215 | 6.08E-09 |
| ENSG00000234883 | *MIR155HG* | -6.977129275 | 0.009187754 |
| ENSG00000137507 | *LRRC32* | -6.981708709 | 1.21E-12 |
| ENSG00000149781 | *FERMT3* | -6.982611639 | 9.70E-10 |
| ENSG00000198108 | *CHSY3* | -6.98311173 | 0.031124712 |
| ENSG00000160282 | *FTCD* | -6.984985361 | 0.001004769 |
| ENSG00000253520 | *RP11-798K23.5* | -6.987970591 | 0.002536083 |
| ENSG00000272971 | *RP11-284F21.11* | -6.988718063 | 0.002783334 |
| ENSG00000137571 | *SLCO5A1* | -6.989589571 | 0.005988358 |
| ENSG00000158477 | *CD1A* | -6.992911258 | 0.046544969 |
| ENSG00000198832 | *SELM* | -7.007109747 | 4.02E-14 |
| ENSG00000221866 | *PLXNA4* | -7.008456558 | 0.009179531 |
| ENSG00000162669 | *HFM1* | -7.011933347 | 0.002730979 |
| ENSG00000103313 | *MEFV* | -7.0131945 | 0.034544954 |
| ENSG00000181234 | *TMEM132C* | -7.013359514 | 0.001216133 |
| ENSG00000146250 | *PRSS35* | -7.02014376 | 0.011108945 |
| ENSG00000115590 | *IL1R2* | -7.02151126 | 0.002363908 |
| ENSG00000258733 | *CTD-2341M24.1* | -7.02549549 | 0.038644711 |
| ENSG00000113657 | *DPYSL3* | -7.025501903 | 1.18E-09 |
| ENSG00000133048 | *CHI3L1* | -7.030226659 | 7.49E-08 |
| ENSG00000255197 | *RP11-750H9.5* | -7.030827621 | 0.022854571 |
| ENSG00000145428 | *RNF175* | -7.030830765 | 5.88E-06 |
| ENSG00000206306 | *HLA-DRB1* | -7.032530166 | 0.029501258 |
| ENSG00000267206 | *LCN6* | -7.033105297 | 0.040645293 |
| ENSG00000176083 | *ZNF683* | -7.034606912 | 0.043698551 |
| ENSG00000008277 | *ADAM22* | -7.04013593 | 3.32E-17 |
| ENSG00000183662 | *FAM19A1* | -7.044242031 | 0.024133584 |
| ENSG00000130600 | *H19* | -7.04664026 | 0.007252536 |
| ENSG00000281759 | *CTD-2007N20.1* | -7.04721436 | 0.034204518 |
| ENSG00000174944 | *P2RY14* | -7.054713362 | 0.003612502 |
| ENSG00000274956 | *UG0898H09* | -7.055870491 | 0.020463684 |
| ENSG00000136167 | *LCP1* | -7.059812704 | 0.006962715 |
| ENSG00000149380 | *P4HA3* | -7.070910761 | 2.26E-07 |
| ENSG00000181036 | *FCRL6* | -7.07281383 | 0.012527986 |
| ENSG00000168961 | *LGALS9* | -7.082104984 | 8.79E-06 |
| ENSG00000000938 | *FGR* | -7.088108647 | 2.51E-13 |
| ENSG00000196196 | *HRCT1* | -7.090477371 | 4.17E-05 |
| ENSG00000213949 | *ITGA1* | -7.092097208 | 9.54E-11 |
| ENSG00000282556 | *RP11-304C12.5* | -7.096803544 | 0.025186538 |
| ENSG00000080709 | *KCNN2* | -7.100692406 | 0.001659478 |
| ENSG00000081052 | *COL4A4* | -7.101231731 | 3.16E-09 |
| ENSG00000233093 | *LINC00892* | -7.101392153 | 0.012724853 |
| ENSG00000178789 | *CD300LB* | -7.117815304 | 0.027550356 |
| ENSG00000235621 | *LINC00494* | -7.118425564 | 0.011750439 |
| ENSG00000184682 | *PRR33* | -7.123115348 | 0.01860204 |
| ENSG00000183783 | *KCTD8* | -7.123983374 | 0.012217507 |
| ENSG00000262003 | *RP11-676J12.7* | -7.12404421 | 0.012206155 |
| ENSG00000162881 | *OXER1* | -7.128003833 | 0.000115609 |
| ENSG00000166105 | *GLB1L3* | -7.133306897 | 1.96E-08 |
| ENSG00000103316 | *CRYM* | -7.140979583 | 0.024205066 |
| ENSG00000150510 | *FAM124A* | -7.141679428 | 4.18E-08 |
| ENSG00000198821 | *CD247* | -7.144100698 | 4.19E-06 |
| ENSG00000175538 | *KCNE3* | -7.151872117 | 1.03E-07 |
| ENSG00000224041 | *IGKV3D-15* | -7.157556433 | 0.00913822 |
| ENSG00000265206 | *RP5-1171I10.5* | -7.157964684 | 0.006946425 |
| ENSG00000007933 | *FMO3* | -7.162558059 | 0.004443458 |
| ENSG00000167850 | *CD300C* | -7.168033721 | 0.01273689 |
| ENSG00000178562 | *CD28* | -7.169766775 | 0.003520465 |
| ENSG00000154198 | *CYP4Z2P* | -7.171702585 | 0.025531334 |
| ENSG00000153823 | *PID1* | -7.173413069 | 1.21E-08 |
| ENSG00000169248 | *CXCL11* | -7.173459214 | 0.003699119 |
| ENSG00000226674 | *TEX41* | -7.178279582 | 0.03004489 |
| ENSG00000179772 | *FOXS1* | -7.1786498 | 0.0176816 |
| ENSG00000086288 | *NME8* | -7.178961865 | 0.011848559 |
| ENSG00000180616 | *SSTR2* | -7.180075415 | 4.87E-07 |
| ENSG00000280285 | *RP11-630A13.4* | -7.181301665 | 0.034577798 |
| ENSG00000181374 | *CCL13* | -7.189141228 | 0.040152153 |
| ENSG00000223403 | *MEG9* | -7.191346511 | 0.001018435 |
| ENSG00000153283 | *CD96* | -7.19956975 | 6.45E-07 |
| ENSG00000048462 | *TNFRSF17* | -7.200101579 | 0.014130974 |
| ENSG00000120324 | *PCDHB10* | -7.201117922 | 0.002541589 |
| ENSG00000189184 | *PCDH18* | -7.20490385 | 1.57E-12 |
| ENSG00000167476 | *JSRP1* | -7.208518582 | 0.000644929 |
| ENSG00000264727 | *RP11-680C21.1* | -7.211012808 | 0.039451774 |
| ENSG00000126950 | *TMEM35* | -7.214689214 | 1.15E-16 |
| ENSG00000197540 | *GZMM* | -7.215395952 | 0.003026882 |
| ENSG00000166006 | *KCNC2* | -7.217368578 | 0.018682435 |
| ENSG00000164434 | *FABP7* | -7.218136019 | 0.003009167 |
| ENSG00000276305 | *AC106788.1* | -7.220687229 | 0.014421862 |
| ENSG00000280543 | *ASAP1-IT2* | -7.221749159 | 0.01456526 |
| ENSG00000178796 | *RIIAD1* | -7.228758017 | 0.032434772 |
| ENSG00000274624 | *RP11-49G2.3* | -7.229071727 | 0.042587898 |
| ENSG00000161055 | *SCGB3A1* | -7.229489121 | 2.03E-06 |
| ENSG00000228252 | *COL6A4P2* | -7.23098768 | 0.014271558 |
| ENSG00000180061 | *TMEM150B* | -7.237054437 | 0.011689322 |
| ENSG00000169994 | *MYO7B* | -7.237853461 | 8.71E-05 |
| ENSG00000182183 | *FAM159A* | -7.238370779 | 0.00309654 |
| ENSG00000168952 | *STXBP6* | -7.23974666 | 7.58E-08 |
| ENSG00000196381 | *ZNF781* | -7.242550448 | 0.00865022 |
| ENSG00000055955 | *ITIH4* | -7.242691979 | 0.011187014 |
| ENSG00000138080 | *EMILIN1* | -7.244820688 | 6.36E-24 |
| ENSG00000205056 | *RP11-693J15.5* | -7.24732363 | 0.014430542 |
| ENSG00000279508 | *AL589743.1* | -7.248349233 | 0.00333458 |
| ENSG00000185739 | *SRL* | -7.24965143 | 0.045874662 |
| ENSG00000203883 | *SOX18* | -7.249708236 | 4.72E-10 |
| ENSG00000100626 | *GALNT16* | -7.262175087 | 8.90E-07 |
| ENSG00000105697 | *HAMP* | -7.262238817 | 0.013105778 |
| ENSG00000124216 | *SNAI1* | -7.263182654 | 0.001365305 |
| ENSG00000248668 | *OXCT1-AS1* | -7.265331077 | 0.003445487 |
| ENSG00000061455 | *PRDM6* | -7.26558984 | 1.15E-08 |
| ENSG00000272529 | *RP11-415F23.4* | -7.267982747 | 0.018564161 |
| ENSG00000206195 | *DUXAP8* | -7.268226008 | 0.005275449 |
| ENSG00000253123 | *RP11-527N22.1* | -7.271388557 | 0.037609217 |
| ENSG00000272491 | *RP5-1024N4.4* | -7.272489807 | 0.012995034 |
| ENSG00000168229 | *PTGDR* | -7.273472161 | 0.009227217 |
| ENSG00000169224 | *GCSAML* | -7.273795585 | 0.012321503 |
| ENSG00000130052 | *STARD8* | -7.275904927 | 3.42E-12 |
| ENSG00000198354 | *DCAF12L2* | -7.278278947 | 0.011007595 |
| ENSG00000101098 | *RIMS4* | -7.280675404 | 0.001030235 |
| ENSG00000136634 | *IL10* | -7.282489937 | 0.015504039 |
| ENSG00000147570 | *DNAJC5B* | -7.28286358 | 0.010765894 |
| ENSG00000148483 | *TMEM236* | -7.285008655 | 0.029674675 |
| ENSG00000107796 | *ACTA2* | -7.293886611 | 5.59E-21 |
| ENSG00000159495 | *TGM7* | -7.295223142 | 0.027679975 |
| ENSG00000135424 | *ITGA7* | -7.296984479 | 8.13E-19 |
| ENSG00000147166 | *ITGB1BP2* | -7.299247044 | 2.30E-06 |
| ENSG00000265531 | *FCGR1C* | -7.302914357 | 0.01153272 |
| ENSG00000167244 | *IGF2* | -7.305841298 | 2.87E-07 |
| ENSG00000150048 | *CLEC1A* | -7.310969698 | 0.000678489 |
| ENSG00000163273 | *NPPC* | -7.314975773 | 0.018256818 |
| ENSG00000134042 | *MRO* | -7.315466525 | 0.025341438 |
| ENSG00000259370 | *RP11-1069G10.1* | -7.318017247 | 0.006001574 |
| ENSG00000158517 | *NCF1* | -7.31997862 | 5.86E-05 |
| ENSG00000068615 | *REEP1* | -7.320142555 | 4.67E-08 |
| ENSG00000154928 | *EPHB1* | -7.326280176 | 0.000468899 |
| ENSG00000273650 | *CTD-3193K9.11* | -7.338942611 | 0.021842906 |
| ENSG00000118407 | *FILIP1* | -7.339706351 | 2.62E-07 |
| ENSG00000081138 | *CDH7* | -7.340335601 | 0.01641939 |
| ENSG00000230537 | *RP11-305L7.1* | -7.343292984 | 0.001260323 |
| ENSG00000198643 | *FAM3D* | -7.344813654 | 0.035648922 |
| ENSG00000109684 | *CLNK* | -7.34675968 | 0.009598564 |
| ENSG00000080224 | *EPHA6* | -7.347800664 | 0.008922904 |
| ENSG00000134853 | *PDGFRA* | -7.34816199 | 2.10E-10 |
| ENSG00000128482 | *RNF112* | -7.348954882 | 1.12E-13 |
| ENSG00000205436 | *EXOC3L4* | -7.349956682 | 1.51E-06 |
| ENSG00000270093 | *AP000473.8* | -7.351632294 | 0.002051596 |
| ENSG00000280237 | *MIR4697HG* | -7.352329771 | 1.24E-09 |
| ENSG00000228278 | *ORM2* | -7.356110223 | 0.003741094 |
| ENSG00000197057 | *DTHD1* | -7.356470642 | 0.004568056 |
| ENSG00000153930 | *ANKFN1* | -7.356714213 | 0.006389061 |
| ENSG00000154258 | *ABCA9* | -7.36127928 | 0.000196915 |
| ENSG00000068831 | *RASGRP2* | -7.363324092 | 2.47E-11 |
| ENSG00000186235 | *AC016757.3* | -7.365849743 | 0.014133595 |
| ENSG00000231156 | *AC093702.1* | -7.36820127 | 0.018141389 |
| ENSG00000224132 | *AC112715.2* | -7.368670579 | 0.026111895 |
| ENSG00000258498 | *DIO3OS* | -7.368691257 | 6.36E-12 |
| ENSG00000280222 | *RP11-174G17.3* | -7.369070957 | 0.0359869 |
| ENSG00000125735 | *TNFSF14* | -7.377772651 | 0.003518194 |
| ENSG00000171101 | *SIGLEC17P* | -7.380834439 | 0.00769501 |
| ENSG00000164035 | *EMCN* | -7.382219816 | 9.50E-09 |
| ENSG00000245869 | *RP11-158I9.5* | -7.387793671 | 0.021400271 |
| ENSG00000187323 | *DCC* | -7.388096769 | 0.026366343 |
| ENSG00000128573 | *FOXP2* | -7.39092468 | 1.19E-06 |
| ENSG00000164488 | *DACT2* | -7.393385486 | 0.001122674 |
| ENSG00000140853 | *NLRC5* | -7.393537114 | 2.98E-13 |
| ENSG00000107719 | *PALD1* | -7.39464023 | 7.44E-11 |
| ENSG00000162174 | *ASRGL1* | -7.394931384 | 3.00E-06 |
| ENSG00000249007 | *RP11-510N19.5* | -7.397328099 | 0.004963355 |
| ENSG00000230798 | *FOXD3-AS1* | -7.398225824 | 0.03639231 |
| ENSG00000267369 | *RP11-1094M14.8* | -7.398716754 | 0.007830577 |
| ENSG00000106952 | *TNFSF8* | -7.399886896 | 0.002554723 |
| ENSG00000126759 | *CFP* | -7.401535697 | 7.64E-05 |
| ENSG00000076662 | *ICAM3* | -7.402660204 | 0.008749519 |
| ENSG00000181778 | *TMEM252* | -7.406519242 | 0.001564436 |
| ENSG00000213996 | *TM6SF2* | -7.409747269 | 0.013321452 |
| ENSG00000151572 | *ANO4* | -7.416490336 | 0.002441522 |
| ENSG00000174640 | *SLCO2A1* | -7.416703943 | 1.01E-09 |
| ENSG00000102970 | *CCL17* | -7.418491519 | 0.008188477 |
| ENSG00000281852 | *LINC00891* | -7.428093797 | 0.010197481 |
| ENSG00000164344 | *KLKB1* | -7.43060517 | 0.001179072 |
| ENSG00000262410 | *RP11-388C12.8* | -7.43164367 | 0.003391255 |
| ENSG00000007171 | *NOS2* | -7.433383674 | 0.017662453 |
| ENSG00000189233 | *NUGGC* | -7.439557211 | 0.008302101 |
| ENSG00000144230 | *GPR17* | -7.440333264 | 0.004169579 |
| ENSG00000180785 | *OR51E1* | -7.441617714 | 0.022261334 |
| ENSG00000132702 | *HAPLN2* | -7.442577428 | 1.08E-13 |
| ENSG00000168546 | *GFRA2* | -7.444846472 | 0.003214915 |
| ENSG00000182326 | *C1S* | -7.444871879 | 3.03E-20 |
| ENSG00000121933 | *ADORA3* | -7.445865868 | 0.006950252 |
| ENSG00000225400 | *RAB28P5* | -7.44977857 | 0.006253281 |
| ENSG00000173714 | *WFIKKN2* | -7.451689702 | 0.013336572 |
| ENSG00000197588 | *KLKP1* | -7.461989981 | 0.005822192 |
| ENSG00000253730 | *RP11-893F2.13* | -7.463078546 | 0.017492482 |
| ENSG00000197705 | *KLHL14* | -7.465189965 | 0.00125682 |
| ENSG00000137273 | *FOXF2* | -7.469115535 | 0.011439288 |
| ENSG00000226306 | *NPY6R* | -7.476311363 | 0.000465497 |
| ENSG00000224003 | *YES1P1* | -7.476448096 | 0.005033349 |
| ENSG00000100055 | *CYTH4* | -7.47651698 | 3.98E-10 |
| ENSG00000166086 | *JAM3* | -7.476661984 | 1.92E-17 |
| ENSG00000120328 | *PCDHB12* | -7.484681225 | 0.004168933 |
| ENSG00000178199 | *ZC3H12D* | -7.486173144 | 0.000541998 |
| ENSG00000183571 | *PGPEP1L* | -7.493352026 | 0.007314826 |
| ENSG00000162706 | *CADM3* | -7.49819457 | 0.002380538 |
| ENSG00000105499 | *PLA2G4C* | -7.49907655 | 5.00E-08 |
| ENSG00000244953 | *RP11-613D13.8* | -7.49996301 | 0.011689802 |
| ENSG00000197599 | *CCDC154* | -7.500186226 | 0.000915077 |
| ENSG00000168280 | *KIF5C* | -7.506442635 | 7.93E-06 |
| ENSG00000168754 | *FAM178B* | -7.510652446 | 0.002384522 |
| ENSG00000186297 | *GABRA5* | -7.514967292 | 0.040511418 |
| ENSG00000204165 | *CXorf65* | -7.517426748 | 0.012001769 |
| ENSG00000172215 | *CXCR6* | -7.518210863 | 0.000351336 |
| ENSG00000121068 | *TBX2* | -7.518551459 | 1.35E-09 |
| ENSG00000231346 | *LINC01160* | -7.521133258 | 0.000265005 |
| ENSG00000237181 | *AC147651.4* | -7.522994861 | 0.009973898 |
| ENSG00000130433 | *CACNG6* | -7.52410724 | 0.049578116 |
| ENSG00000086991 | *NOX4* | -7.525367755 | 0.013809592 |
| ENSG00000166428 | *PLD4* | -7.526903058 | 0.000536688 |
| ENSG00000113088 | *GZMK* | -7.52753887 | 0.00363526 |
| ENSG00000183813 | *CCR4* | -7.528252301 | 0.002263916 |
| ENSG00000164483 | *SAMD3* | -7.531722904 | 0.007640594 |
| ENSG00000172346 | *CSDC2* | -7.532970985 | 8.02E-11 |
| ENSG00000027644 | *INSRR* | -7.534963458 | 0.00959712 |
| ENSG00000075035 | *WSCD2* | -7.537021768 | 4.65E-07 |
| ENSG00000211653 | *IGLV1-40* | -7.537274304 | 0.018278603 |
| ENSG00000091137 | *SLC26A4* | -7.541188087 | 4.19E-06 |
| ENSG00000206432 | *TMEM200C* | -7.543840618 | 0.00665341 |
| ENSG00000115353 | *TACR1* | -7.54528407 | 0.002967956 |
| ENSG00000197380 | *DACT3* | -7.553242919 | 2.10E-16 |
| ENSG00000176533 | *GNG7* | -7.554442917 | 1.74E-18 |
| ENSG00000137726 | *FXYD6* | -7.555477379 | 3.77E-10 |
| ENSG00000205038 | *PKHD1L1* | -7.562275548 | 0.006527319 |
| ENSG00000255794 | *RMST* | -7.565132455 | 0.003295119 |
| ENSG00000163568 | *AIM2* | -7.57360464 | 0.002783334 |
| ENSG00000196664 | *TLR7* | -7.574098074 | 0.002031539 |
| ENSG00000146013 | *GFRA3* | -7.577001114 | 0.00151536 |
| ENSG00000131471 | *AOC3* | -7.579796051 | 5.57E-22 |
| ENSG00000276644 | *DACH1* | -7.580046095 | 0.000704883 |
| ENSG00000148516 | *ZEB1* | -7.580996742 | 3.85E-12 |
| ENSG00000134193 | *REG4* | -7.582383794 | 0.022133671 |
| ENSG00000181631 | *P2RY13* | -7.585533489 | 0.001871118 |
| ENSG00000113396 | *SLC27A6* | -7.593879107 | 1.58E-07 |
| ENSG00000150594 | *ADRA2A* | -7.597573345 | 0.000162895 |
| ENSG00000152910 | *CNTNAP4* | -7.597886736 | 0.04708394 |
| ENSG00000135144 | *DTX1* | -7.601583841 | 0.000225612 |
| ENSG00000153902 | *LGI4* | -7.601863563 | 5.72E-12 |
| ENSG00000165300 | *SLITRK5* | -7.60537383 | 4.37E-08 |
| ENSG00000122223 | *CD244* | -7.605576524 | 0.005828213 |
| ENSG00000177301 | *KCNA2* | -7.606714428 | 0.001325371 |
| ENSG00000227039 | *ITGB2-AS1* | -7.60897883 | 1.43E-05 |
| ENSG00000263639 | *MSMB* | -7.609184003 | 0.011502973 |
| ENSG00000131055 | *COX4I2* | -7.609276505 | 0.000704883 |
| ENSG00000120729 | *MYOT* | -7.609293331 | 0.026476871 |
| ENSG00000151388 | *ADAMTS12* | -7.610202137 | 0.001751613 |
| ENSG00000172733 | *PURG* | -7.61596421 | 0.012909667 |
| ENSG00000255240 | *RP11-142C4.6* | -7.617607206 | 0.003451846 |
| ENSG00000236625 | *C4B* | -7.61820644 | 0.021465217 |
| ENSG00000165323 | *FAT3* | -7.621976444 | 8.59E-09 |
| ENSG00000170382 | *LRRN2* | -7.622838381 | 0.000494189 |
| ENSG00000214787 | *MS4A4E* | -7.625032962 | 0.005828213 |
| ENSG00000037280 | *FLT4* | -7.62719752 | 3.76E-05 |
| ENSG00000211689 | *TRGC1* | -7.630703659 | 0.00304289 |
| ENSG00000278468 | *MUC4* | -7.637602854 | 0.02924062 |
| ENSG00000082397 | *EPB41L3* | -7.637926139 | 7.83E-10 |
| ENSG00000251301 | *RP11-81H14.2* | -7.643133443 | 0.013563671 |
| ENSG00000110076 | *NRXN2* | -7.646661296 | 0.000211201 |
| ENSG00000123358 | *NR4A1* | -7.648294999 | 3.97E-10 |
| ENSG00000115896 | *PLCL1* | -7.650041256 | 1.54E-10 |
| ENSG00000148735 | *PLEKHS1* | -7.656018565 | 0.000110277 |
| ENSG00000115468 | *EFHD1* | -7.667701732 | 1.40E-11 |
| ENSG00000039537 | *C6* | -7.67218543 | 0.010250426 |
| ENSG00000241163 | *LINC00877* | -7.672536606 | 0.007512899 |
| ENSG00000144868 | *TMEM108* | -7.673807073 | 0.003360525 |
| ENSG00000068976 | *PYGM* | -7.679434867 | 3.30E-12 |
| ENSG00000244682 | *FCGR2C* | -7.680275567 | 0.000232055 |
| ENSG00000225194 | *LINC00092* | -7.680508361 | 0.003274076 |
| ENSG00000261625 | *RP11-554A11.4* | -7.680669841 | 0.001691905 |
| ENSG00000168995 | *SIGLEC7* | -7.695204148 | 0.002429772 |
| ENSG00000177464 | *GPR4* | -7.695788536 | 0.000847092 |
| ENSG00000130224 | *LRCH2* | -7.696635178 | 2.67E-10 |
| ENSG00000272674 | *PCDHB16* | -7.69791248 | 0.000241718 |
| ENSG00000232977 | *LINC00327* | -7.708465722 | 0.00666263 |
| ENSG00000174407 | *MIR1-1HG* | -7.708496718 | 0.006962715 |
| ENSG00000166592 | *RRAD* | -7.711481745 | 1.25E-08 |
| ENSG00000197977 | *ELOVL2* | -7.715113144 | 0.00053682 |
| ENSG00000127129 | *EDN2* | -7.715881376 | 5.37E-07 |
| ENSG00000073861 | *TBX21* | -7.717615232 | 0.002790385 |
| ENSG00000224982 | *TMEM233* | -7.7217995 | 0.012188088 |
| ENSG00000198796 | *ALPK2* | -7.721915815 | 0.013386888 |
| ENSG00000141433 | *ADCYAP1* | -7.722519574 | 0.022304471 |
| ENSG00000268505 | *RP11-805I24.3* | -7.723523819 | 0.029341959 |
| ENSG00000171115 | *GIMAP8* | -7.726099062 | 1.43E-06 |
| ENSG00000272463 | *RP11-532F6.3* | -7.729322061 | 0.003193729 |
| ENSG00000278243 | *TSTA3* | -7.740893029 | 0.000798805 |
| ENSG00000127324 | *TSPAN8* | -7.74208036 | 9.78E-10 |
| ENSG00000248596 | *RP11-844P9.2* | -7.742333188 | 1.85E-06 |
| ENSG00000163600 | *ICOS* | -7.74268424 | 0.004024227 |
| ENSG00000182836 | *PLCXD3* | -7.74835683 | 0.002504506 |
| ENSG00000146192 | *FGD2* | -7.754811983 | 5.45E-11 |
| ENSG00000197406 | *DIO3* | -7.755228966 | 0.00085707 |
| ENSG00000245164 | *LINC00861* | -7.760903106 | 0.00269165 |
| ENSG00000225978 | *HAR1A* | -7.763645965 | 0.004515705 |
| ENSG00000084674 | *APOB* | -7.773834769 | 0.011019036 |
| ENSG00000242600 | *MBL1P* | -7.777736882 | 0.000137415 |
| ENSG00000277351 | *RP11-325L12.6* | -7.781458863 | 0.008003115 |
| ENSG00000255545 | *RP11-627G23.1* | -7.782319226 | 0.03037462 |
| ENSG00000163909 | *HEYL* | -7.786188813 | 3.40E-09 |
| ENSG00000187479 | *C11orf96* | -7.795182194 | 1.46E-10 |
| ENSG00000255051 | *BCAS2P1* | -7.801225217 | 0.011815299 |
| ENSG00000182557 | *SPNS3* | -7.804172078 | 0.002213633 |
| ENSG00000204930 | *FAM221B* | -7.805729578 | 0.007018214 |
| ENSG00000211751 | *TRBV25-1* | -7.807171903 | 0.002224143 |
| ENSG00000161944 | *ASGR2* | -7.810209837 | 0.010988488 |
| ENSG00000272668 | *RP11-190A12.8* | -7.812319381 | 0.00038347 |
| ENSG00000078053 | *AMPH* | -7.820956395 | 0.000189614 |
| ENSG00000154721 | *JAM2* | -7.821270991 | 2.86E-11 |
| ENSG00000145087 | *STXBP5L* | -7.826977338 | 0.000233986 |
| ENSG00000188803 | *SHISA6* | -7.828313427 | 0.001959495 |
| ENSG00000232414 | *CYP21A2* | -7.828651747 | 0.014027872 |
| ENSG00000196376 | *SLC35F1* | -7.829359758 | 0.003633772 |
| ENSG00000204071 | *TCEAL6* | -7.832395183 | 0.014244033 |
| ENSG00000276849 | *TRBC2* | -7.832483681 | 0.001265223 |
| ENSG00000211659 | *IGLV3-25* | -7.83772394 | 0.002644316 |
| ENSG00000282784 | *AC245015.2* | -7.840401736 | 0.026186209 |
| ENSG00000275990 | *NCF4* | -7.843551962 | 0.008276692 |
| ENSG00000105967 | *TFEC* | -7.84711771 | 0.000211677 |
| ENSG00000121898 | *CPXM2* | -7.849272762 | 7.70E-11 |
| ENSG00000280977 | *Z93930.1* | -7.854364141 | 0.000493546 |
| ENSG00000161940 | *BCL6B* | -7.855467479 | 4.55E-05 |
| ENSG00000184845 | *DRD1* | -7.856636563 | 0.000722099 |
| ENSG00000253522 | *CTC-231O11.1* | -7.859315248 | 0.009529119 |
| ENSG00000139874 | *SSTR1* | -7.864275414 | 0.004764225 |
| ENSG00000179855 | *GIPC3* | -7.870377988 | 0.000417464 |
| ENSG00000123610 | *TNFAIP6* | -7.871319945 | 0.006501435 |
| ENSG00000196329 | *GIMAP5* | -7.876216933 | 0.000529952 |
| ENSG00000242142 | *SERBP1P3* | -7.87724861 | 0.001569273 |
| ENSG00000172349 | *IL16* | -7.881906147 | 2.04E-09 |
| ENSG00000122585 | *NPY* | -7.882770289 | 0.000498043 |
| ENSG00000127920 | *GNG11* | -7.884356955 | 1.02E-08 |
| ENSG00000172348 | *RCAN2* | -7.884588319 | 2.88E-17 |
| ENSG00000189058 | *APOD* | -7.890954601 | 4.61E-31 |
| ENSG00000182866 | *LCK* | -7.894147893 | 9.13E-07 |
| ENSG00000175857 | *GAPT* | -7.895769739 | 0.001182837 |
| ENSG00000233117 | *LINC00702* | -7.897712905 | 0.003171841 |
| ENSG00000113494 | *PRLR* | -7.900286817 | 0.00172781 |
| ENSG00000129596 | *CDO1* | -7.904311369 | 3.86E-09 |
| ENSG00000205502 | *C2CD4B* | -7.920002894 | 2.29E-06 |
| ENSG00000092051 | *JPH4* | -7.924168692 | 8.06E-10 |
| ENSG00000228203 | *RNF144A-AS1* | -7.925366862 | 0.005365325 |
| ENSG00000213203 | *GIMAP1* | -7.92537736 | 6.15E-05 |
| ENSG00000160654 | *CD3G* | -7.931444838 | 0.001038624 |
| ENSG00000231246 | *RP5-965F6.2* | -7.931918594 | 0.004927355 |
| ENSG00000233705 | *SLC26A4-AS1* | -7.932197665 | 0.002195719 |
| ENSG00000142185 | *TRPM2* | -7.932383612 | 2.26E-06 |
| ENSG00000144891 | *AGTR1* | -7.938702772 | 0.005802229 |
| ENSG00000196361 | *ELAVL3* | -7.94089663 | 0.006237663 |
| ENSG00000173991 | *TCAP* | -7.942725174 | 0.002058371 |
| ENSG00000151322 | *NPAS3* | -7.945972835 | 0.001289582 |
| ENSG00000258469 | *CHMP4BP1* | -7.947493913 | 0.002367623 |
| ENSG00000231817 | *LINC01198* | -7.953354321 | 0.024942884 |
| ENSG00000125740 | *FOSB* | -7.953598775 | 0.001736825 |
| ENSG00000270547 | *LINC01235* | -7.956883338 | 0.001202824 |
| ENSG00000172322 | *CLEC12A* | -7.957379833 | 0.000697815 |
| ENSG00000265465 | *MIR4768* | -7.960270202 | 0.004082873 |
| ENSG00000163154 | *TNFAIP8L2* | -7.975775207 | 0.000164865 |
| ENSG00000197520 | *FAM177B* | -7.976007994 | 0.000924914 |
| ENSG00000146285 | *SCML4* | -7.976731356 | 0.000323084 |
| ENSG00000104490 | *NCALD* | -7.977768943 | 2.49E-09 |
| ENSG00000151490 | *PTPRO* | -7.978587693 | 0.001104045 |
| ENSG00000188778 | *ADRB3* | -7.980724511 | 0.014244033 |
| ENSG00000184524 | *CEND1* | -7.982928859 | 7.37E-08 |
| ENSG00000276886 | *GREM1* | -7.98815874 | 0.002459484 |
| ENSG00000183844 | *FAM3B* | -7.98914034 | 5.75E-05 |
| ENSG00000133116 | *KL* | -7.989864638 | 0.000137426 |
| ENSG00000167434 | *CA4* | -7.991048567 | 0.001122721 |
| ENSG00000151650 | *VENTX* | -7.993771404 | 0.002204209 |
| ENSG00000149970 | *CNKSR2* | -7.994885143 | 0.00052982 |
| ENSG00000148655 | *C10orf11* | -7.999381868 | 1.10E-08 |
| ENSG00000189056 | *RELN* | -8.005841278 | 0.00146031 |
| ENSG00000174332 | *GLIS1* | -8.007309835 | 0.012221115 |
| ENSG00000196208 | *GREB1* | -8.009034018 | 6.53E-07 |
| ENSG00000090659 | *CD209* | -8.010919413 | 0.00018337 |
| ENSG00000277022 | *RP3-453C12.15* | -8.011155961 | 0.004503273 |
| ENSG00000173175 | *ADCY5* | -8.013089359 | 8.13E-17 |
| ENSG00000162733 | *DDR2* | -8.014404267 | 6.10E-25 |
| ENSG00000278309 | *RP11-102K13.5* | -8.016664204 | 0.000276928 |
| ENSG00000170837 | *GPR27* | -8.025431625 | 8.80E-05 |
| ENSG00000118946 | *PCDH17* | -8.026856044 | 0.001486448 |
| ENSG00000225706 | *PTPRD-AS1* | -8.02771306 | 0.035242493 |
| ENSG00000127743 | *IL17B* | -8.032281388 | 8.37E-05 |
| ENSG00000189129 | *PLAC9* | -8.034994858 | 1.31E-09 |
| ENSG00000115607 | *IL18RAP* | -8.036632192 | 0.000909162 |
| ENSG00000120251 | *GRIA2* | -8.043839375 | 0.004322056 |
| ENSG00000241666 | *RP3-455J7.4* | -8.047870072 | 0.002395467 |
| ENSG00000162383 | *SLC1A7* | -8.048993698 | 0.000478635 |
| ENSG00000115361 | *ACADL* | -8.050898061 | 1.01E-09 |
| ENSG00000112303 | *VNN2* | -8.05174915 | 3.76E-05 |
| ENSG00000128918 | *ALDH1A2* | -8.054408253 | 4.38E-15 |
| ENSG00000248771 | *LINC01207* | -8.058253536 | 0.002972101 |
| ENSG00000211890 | *IGHA2* | -8.067092365 | 0.00014568 |
| ENSG00000137078 | *SIT1* | -8.071452763 | 0.000837181 |
| ENSG00000170801 | *HTRA3* | -8.073415858 | 3.53E-11 |
| ENSG00000126860 | *EVI2A* | -8.076959492 | 6.75E-05 |
| ENSG00000035720 | *STAP1* | -8.086223234 | 0.002188672 |
| ENSG00000079263 | *SP140* | -8.08872165 | 0.000313144 |
| ENSG00000134830 | *C5AR2* | -8.089737274 | 0.004123551 |
| ENSG00000253308 | *RP1-170O19.17* | -8.09008609 | 0.000232055 |
| ENSG00000007264 | *MATK* | -8.095261969 | 0.000197952 |
| ENSG00000121577 | *POPDC2* | -8.095572035 | 2.25E-14 |
| ENSG00000007908 | *SELE* | -8.101183559 | 4.09E-10 |
| ENSG00000123838 | *C4BPA* | -8.102782745 | 0.007599303 |
| ENSG00000155962 | *CLIC2* | -8.103926362 | 2.93E-07 |
| ENSG00000158714 | *SLAMF8* | -8.11208428 | 1.94E-08 |
| ENSG00000086967 | *MYBPC2* | -8.11521028 | 0.046047433 |
| ENSG00000173702 | *MUC13* | -8.115444807 | 0.00262921 |
| ENSG00000155011 | *DKK2* | -8.115924843 | 0.000385356 |
| ENSG00000149599 | *DUSP15* | -8.119179908 | 4.86E-05 |
| ENSG00000159307 | *SCUBE1* | -8.120208073 | 0.000773236 |
| ENSG00000064692 | *SNCAIP* | -8.13291945 | 2.30E-07 |
| ENSG00000103569 | *AQP9* | -8.134103369 | 0.004257384 |
| ENSG00000143195 | *ILDR2* | -8.138855917 | 1.15E-05 |
| ENSG00000227191 | *TRGC2* | -8.140809481 | 0.000206899 |
| ENSG00000101134 | *DOK5* | -8.142222724 | 0.001829378 |
| ENSG00000100351 | *GRAP2* | -8.143983509 | 0.00014059 |
| ENSG00000078589 | *P2RY10* | -8.144322363 | 0.002024144 |
| ENSG00000116748 | *AMPD1* | -8.147860075 | 0.001680746 |
| ENSG00000006071 | *ABCC8* | -8.157309836 | 0.000235884 |
| ENSG00000169291 | *SHE* | -8.16447921 | 2.67E-05 |
| ENSG00000280726 | *PCAT19* | -8.169159935 | 0.003198819 |
| ENSG00000154589 | *LY96* | -8.175507431 | 0.000109461 |
| ENSG00000095637 | *SORBS1* | -8.176304049 | 1.07E-24 |
| ENSG00000176049 | *JAKMIP2* | -8.181545238 | 0.001608237 |
| ENSG00000090382 | *LYZ* | -8.182853583 | 2.11E-09 |
| ENSG00000152672 | *CLEC4F* | -8.184265753 | 0.000320337 |
| ENSG00000259884 | *RP11-1100L3.8* | -8.187214724 | 0.000525501 |
| ENSG00000133083 | *DCLK1* | -8.188335285 | 8.74E-11 |
| ENSG00000185046 | *ANKS1B* | -8.191384173 | 0.00024724 |
| ENSG00000141639 | *MAPK4* | -8.1917309 | 3.52E-05 |
| ENSG00000211664 | *IGLV2-18* | -8.193208327 | 0.008502174 |
| ENSG00000250360 | *CTD-2089N3.1* | -8.193346308 | 0.000404768 |
| ENSG00000185652 | *NTF3* | -8.197217854 | 0.000723627 |
| ENSG00000225670 | *CADM3-AS1* | -8.199470646 | 0.000830624 |
| ENSG00000167641 | *PPP1R14A* | -8.213884643 | 1.33E-15 |
| ENSG00000198223 | *CSF2RA* | -8.214466344 | 1.58E-07 |
| ENSG00000228798 | *AP000473.5* | -8.22197681 | 0.001931172 |
| ENSG00000198400 | *NTRK1* | -8.222478798 | 0.000367495 |
| ENSG00000184005 | *ST6GALNAC3* | -8.232640491 | 0.001661763 |
| ENSG00000168675 | *LDLRAD4* | -8.239488878 | 9.92E-19 |
| ENSG00000166091 | *CMTM5* | -8.246508199 | 0.000892504 |
| ENSG00000263961 | *C1orf186* | -8.247661297 | 7.51E-05 |
| ENSG00000152969 | *JAKMIP1* | -8.251511081 | 0.000426406 |
| ENSG00000206190 | *ATP10A* | -8.252269621 | 8.25E-09 |
| ENSG00000085563 | *ABCB1* | -8.257363408 | 9.80E-06 |
| ENSG00000262370 | *RP11-473M20.9* | -8.260635537 | 0.000266638 |
| ENSG00000127074 | *RGS13* | -8.261273831 | 0.002181022 |
| ENSG00000116774 | *OLFML3* | -8.261448485 | 2.72E-13 |
| ENSG00000111728 | *ST8SIA1* | -8.262550687 | 2.16E-11 |
| ENSG00000167083 | *GNGT2* | -8.262814411 | 0.000460343 |
| ENSG00000132639 | *SNAP25* | -8.262928989 | 1.45E-05 |
| ENSG00000179915 | *NRXN1* | -8.265625123 | 0.000856978 |
| ENSG00000161896 | *IP6K3* | -8.266287649 | 8.94E-08 |
| ENSG00000134917 | *ADAMTS8* | -8.269975356 | 2.74E-12 |
| ENSG00000123342 | *MMP19* | -8.27467628 | 3.23E-09 |
| ENSG00000089012 | *SIRPG* | -8.277118062 | 0.000535438 |
| ENSG00000282608 | *ADORA3* | -8.282822894 | 0.000649546 |
| ENSG00000160185 | *UBASH3A* | -8.284256733 | 0.000508535 |
| ENSG00000138395 | *CDK15* | -8.288626411 | 0.001251574 |
| ENSG00000265542 | *RP11-60A24.3* | -8.289015176 | 0.0019773 |
| ENSG00000121807 | *CCR2* | -8.290079778 | 0.000238685 |
| ENSG00000001626 | *CFTR* | -8.292288372 | 8.37E-07 |
| ENSG00000188859 | *FAM78B* | -8.305752107 | 0.000137415 |
| ENSG00000204442 | *FAM155A* | -8.308612617 | 0.000271526 |
| ENSG00000124203 | *ZNF831* | -8.309820568 | 0.000341201 |
| ENSG00000251321 | *PCAT4* | -8.310080799 | 0.003268155 |
| ENSG00000101331 | *CCM2L* | -8.310324164 | 8.08E-05 |
| ENSG00000174576 | *NPAS4* | -8.310717667 | 0.000167438 |
| ENSG00000167261 | *DPEP2* | -8.313614634 | 2.72E-05 |
| ENSG00000182487 | *NCF1B* | -8.317360745 | 0.001348043 |
| ENSG00000129450 | *SIGLEC9* | -8.317982245 | 0.000150904 |
| ENSG00000127329 | *PTPRB* | -8.318834683 | 1.26E-08 |
| ENSG00000164867 | *NOS3* | -8.321984139 | 2.44E-05 |
| ENSG00000234336 | *JAZF1-AS1* | -8.327559237 | 0.001000043 |
| ENSG00000281103 | *TRG-AS1* | -8.335086841 | 8.81E-05 |
| ENSG00000108018 | *SORCS1* | -8.337847095 | 0.000134714 |
| ENSG00000173114 | *LRRN3* | -8.341527951 | 0.000332401 |
| ENSG00000179542 | *SLITRK4* | -8.341947303 | 0.00304499 |
| ENSG00000229847 | *EMX2OS* | -8.341955805 | 0.002637329 |
| ENSG00000105246 | *EBI3* | -8.345888657 | 3.95E-05 |
| ENSG00000223382 | *RP1-65J11.1* | -8.353053073 | 0.001968578 |
| ENSG00000110665 | *C11orf21* | -8.370543646 | 0.00078877 |
| ENSG00000112280 | *COL9A1* | -8.373011793 | 0.002685067 |
| ENSG00000271811 | *RP1-79C4.4* | -8.378328036 | 0.000217127 |
| ENSG00000232057 | *AC093390.1* | -8.389600695 | 0.005526065 |
| ENSG00000157570 | *TSPAN18* | -8.390496317 | 5.33E-12 |
| ENSG00000082482 | *KCNK2* | -8.3943975 | 0.000649546 |
| ENSG00000211685 | *IGLC7* | -8.394843821 | 0.00055391 |
| ENSG00000186827 | *TNFRSF4* | -8.396155884 | 5.73E-05 |
| ENSG00000130988 | *RGN* | -8.399034094 | 6.11E-13 |
| ENSG00000134242 | *PTPN22* | -8.400003942 | 0.000325547 |
| ENSG00000010282 | *HHATL* | -8.403805438 | 0.003307193 |
| ENSG00000152092 | *ASTN1* | -8.40411787 | 0.000784957 |
| ENSG00000166509 | *CLEC3A* | -8.405372826 | 0.007348444 |
| ENSG00000113361 | *CDH6* | -8.421118086 | 0.000380535 |
| ENSG00000162267 | *ITIH3* | -8.421425625 | 0.000693363 |
| ENSG00000160219 | *GAB3* | -8.428190748 | 1.13E-05 |
| ENSG00000174946 | *GPR171* | -8.434510418 | 0.000275362 |
| ENSG00000214578 | *HMGN2P15* | -8.434976018 | 0.000313144 |
| ENSG00000244116 | *IGKV2-28* | -8.436148397 | 0.001747881 |
| ENSG00000026751 | *SLAMF7* | -8.440414895 | 7.26E-07 |
| ENSG00000143603 | *KCNN3* | -8.444967439 | 2.21E-05 |
| ENSG00000178860 | *MSC* | -8.450015615 | 0.000266153 |
| ENSG00000122862 | *SRGN* | -8.450799828 | 1.47E-17 |
| ENSG00000183918 | *SH2D1A* | -8.451029612 | 0.000546133 |
| ENSG00000149564 | *ESAM* | -8.452608893 | 6.27E-09 |
| ENSG00000167912 | *RP11-25K19.1* | -8.45404452 | 0.000689624 |
| ENSG00000178828 | *RNF186* | -8.454108073 | 0.036166751 |
| ENSG00000279377 | *AC003973.3* | -8.458484013 | 0.000286534 |
| ENSG00000250208 | *FZD10-AS1* | -8.460197255 | 7.56E-06 |
| ENSG00000184588 | *PDE4B* | -8.460206976 | 5.93E-10 |
| ENSG00000275575 | *PTP4A3* | -8.466805756 | 0.000102396 |
| ENSG00000134532 | *SOX5* | -8.467036218 | 0.000290026 |
| ENSG00000206305 | *HLA-DQA1* | -8.467872772 | 0.000410391 |
| ENSG00000014257 | *ACPP* | -8.468579737 | 1.24E-10 |
| ENSG00000279484 | *KLHL30-AS1* | -8.478802098 | 0.002154774 |
| ENSG00000168427 | *KLHL30* | -8.479121023 | 0.00024981 |
| ENSG00000197415 | *VEPH1* | -8.481770218 | 0.000849134 |
| ENSG00000121361 | *KCNJ8* | -8.483404438 | 1.56E-10 |
| ENSG00000158270 | *COLEC12* | -8.484374569 | 3.66E-06 |
| ENSG00000158488 | *CD1E* | -8.487605558 | 0.000308085 |
| ENSG00000088320 | *REM1* | -8.491106245 | 6.37E-05 |
| ENSG00000004776 | *HSPB6* | -8.49191729 | 1.61E-15 |
| ENSG00000216490 | *IFI30* | -8.493882962 | 0.001481231 |
| ENSG00000248309 | *MEF2C-AS1* | -8.498297285 | 0.001431842 |
| ENSG00000108405 | *P2RX1* | -8.510092853 | 7.91E-06 |
| ENSG00000142583 | *SLC2A5* | -8.510162555 | 1.89E-10 |
| ENSG00000153303 | *FRMD1* | -8.516273001 | 0.010779332 |
| ENSG00000279496 | *CTC-251I16.1* | -8.51924197 | 4.46E-05 |
| ENSG00000241641 | *RPS23P6* | -8.521974715 | 0.001699599 |
| ENSG00000128655 | *PDE11A* | -8.530362477 | 1.48E-05 |
| ENSG00000164616 | *FBXL21* | -8.530577515 | 0.000817166 |
| ENSG00000227418 | *PCGEM1* | -8.53329498 | 0.000660881 |
| ENSG00000230500 | *MKX-AS1* | -8.534447074 | 0.000470259 |
| ENSG00000122224 | *LY9* | -8.540594089 | 0.000242116 |
| ENSG00000154269 | *ENPP3* | -8.541942607 | 0.001249675 |
| ENSG00000171596 | *NMUR1* | -8.542034292 | 0.000118596 |
| ENSG00000018625 | *ATP1A2* | -8.546173527 | 1.58E-18 |
| ENSG00000078328 | *RBFOX1* | -8.546605406 | 0.000854592 |
| ENSG00000121871 | *SLITRK3* | -8.546673323 | 0.000927159 |
| ENSG00000115523 | *GNLY* | -8.551921207 | 0.000306685 |
| ENSG00000162692 | *VCAM1* | -8.55491601 | 8.01E-09 |
| ENSG00000148357 | *HMCN2* | -8.560315663 | 1.84E-06 |
| ENSG00000186198 | *SLC51B* | -8.568772941 | 0.000405203 |
| ENSG00000169760 | *NLGN1* | -8.574531886 | 0.000433471 |
| ENSG00000261269 | *RP11-389C8.2* | -8.584082359 | 7.42E-05 |
| ENSG00000050030 | *KIAA2022* | -8.586481171 | 7.56E-05 |
| ENSG00000186469 | *GNG2* | -8.588062275 | 3.16E-10 |
| ENSG00000205002 | *AARD* | -8.588448286 | 0.002468804 |
| ENSG00000248587 | *GDNF-AS1* | -8.588744495 | 6.37E-05 |
| ENSG00000170276 | *HSPB2* | -8.593627105 | 0.001804923 |
| ENSG00000170396 | *ZNF804A* | -8.596619705 | 0.000156227 |
| ENSG00000235531 | *MSC-AS1* | -8.600743351 | 0.000261335 |
| ENSG00000140030 | *GPR65* | -8.60633336 | 6.89E-05 |
| ENSG00000162878 | *PKDCC* | -8.608175435 | 7.68E-11 |
| ENSG00000250423 | *KIAA1210* | -8.624226145 | 0.032724546 |
| ENSG00000105851 | *PIK3CG* | -8.625956127 | 0.000954692 |
| ENSG00000053328 | *METTL24* | -8.631604804 | 0.000595549 |
| ENSG00000114654 | *EFCC1* | -8.634185575 | 1.61E-05 |
| ENSG00000120280 | *CXorf21* | -8.634365488 | 0.000687715 |
| ENSG00000277494 | *GPIHBP1* | -8.634736297 | 0.000185333 |
| ENSG00000156427 | *FGF18* | -8.636300586 | 8.66E-05 |
| ENSG00000102383 | *ZDHHC15* | -8.641666038 | 0.000196741 |
| ENSG00000004799 | *PDK4* | -8.642490215 | 6.78E-08 |
| ENSG00000142303 | *ADAMTS10* | -8.644421887 | 6.94E-09 |
| ENSG00000170476 | *MZB1* | -8.644494715 | 0.000689624 |
| ENSG00000106538 | *RARRES2* | -8.654968655 | 7.19E-16 |
| ENSG00000101335 | *MYL9* | -8.656876851 | 1.64E-20 |
| ENSG00000093134 | *VNN3* | -8.659256562 | 0.010487379 |
| ENSG00000197245 | *FAM110D* | -8.659677355 | 2.77E-05 |
| ENSG00000140459 | *CYP11A1* | -8.662924985 | 0.000148254 |
| ENSG00000269113 | *TRABD2B* | -8.663439189 | 1.12E-05 |
| ENSG00000158022 | *TRIM63* | -8.667054853 | 0.000155089 |
| ENSG00000135502 | *SLC26A10* | -8.674912503 | 1.87E-05 |
| ENSG00000179954 | *SSC5D* | -8.675078032 | 8.43E-09 |
| ENSG00000249751 | *ECSCR* | -8.67788246 | 0.000291585 |
| ENSG00000150681 | *RGS18* | -8.681817807 | 0.00010633 |
| ENSG00000101440 | *ASIP* | -8.682625531 | 0.000921035 |
| ENSG00000198846 | *TOX* | -8.685464413 | 6.48E-06 |
| ENSG00000132554 | *RGS22* | -8.686390521 | 1.97E-05 |
| ENSG00000114854 | *TNNC1* | -8.68721718 | 0.001661763 |
| ENSG00000198795 | *ZNF521* | -8.690569975 | 2.61E-05 |
| ENSG00000183837 | *PNMA3* | -8.695541393 | 2.64E-05 |
| ENSG00000183798 | *EMILIN3* | -8.696944442 | 4.75E-06 |
| ENSG00000010671 | *BTK* | -8.706241477 | 2.21E-05 |
| ENSG00000103522 | *IL21R* | -8.708438533 | 0.000380065 |
| ENSG00000135094 | *SDS* | -8.710122191 | 5.04E-05 |
| ENSG00000231672 | *DIRC3* | -8.711457227 | 0.00021167 |
| ENSG00000172116 | *CD8B* | -8.712678029 | 7.24E-05 |
| ENSG00000165379 | *LRFN5* | -8.714570645 | 1.44E-05 |
| ENSG00000140368 | *PSTPIP1* | -8.718101933 | 7.37E-08 |
| ENSG00000253829 | *RP11-723D22.3* | -8.722553137 | 5.60E-05 |
| ENSG00000118473 | *SGIP1* | -8.724247733 | 7.05E-06 |
| ENSG00000274993 | *RP11-395B7.2* | -8.726658132 | 0.000865508 |
| ENSG00000146122 | *DAAM2* | -8.73163782 | 1.23E-10 |
| ENSG00000069431 | *ABCC9* | -8.733715627 | 7.34E-11 |
| ENSG00000143768 | *LEFTY2* | -8.73587052 | 0.001475348 |
| ENSG00000101336 | *HCK* | -8.741436474 | 1.48E-09 |
| ENSG00000110446 | *SLC15A3* | -8.746181901 | 1.37E-08 |
| ENSG00000109099 | *PMP22* | -8.748162162 | 4.29E-17 |
| ENSG00000155816 | *FMN2* | -8.758795046 | 1.89E-05 |
| ENSG00000175489 | *LRRC25* | -8.762693303 | 5.04E-06 |
| ENSG00000203710 | *CR1* | -8.769742864 | 0.000358991 |
| ENSG00000088992 | *TESC* | -8.773054676 | 1.00E-05 |
| ENSG00000163827 | *LRRC2* | -8.774720563 | 0.00035086 |
| ENSG00000065609 | *SNAP91* | -8.775475108 | 0.000187437 |
| ENSG00000167281 | *RBFOX3* | -8.778912164 | 4.36E-15 |
| ENSG00000274827 | *LINC01297* | -8.782851254 | 6.63E-05 |
| ENSG00000198756 | *COLGALT2* | -8.784648011 | 3.44E-09 |
| ENSG00000104213 | *PDGFRL* | -8.785999405 | 3.75E-05 |
| ENSG00000198502 | *HLA-DRB5* | -8.805970906 | 3.97E-05 |
| ENSG00000101916 | *TLR8* | -8.80828239 | 0.000494189 |
| ENSG00000110324 | *IL10RA* | -8.812048861 | 6.49E-12 |
| ENSG00000144619 | *CNTN4* | -8.812748937 | 9.54E-05 |
| ENSG00000160539 | *PPAPDC3* | -8.815560512 | 3.31E-06 |
| ENSG00000106819 | *ASPN* | -8.82277377 | 1.88E-05 |
| ENSG00000185477 | *GPRIN3* | -8.828442619 | 2.21E-05 |
| ENSG00000171873 | *ADRA1D* | -8.829912709 | 3.29E-06 |
| ENSG00000072694 | *FCGR2B* | -8.830227703 | 1.62E-05 |
| ENSG00000067840 | *PDZD4* | -8.830384503 | 1.37E-11 |
| ENSG00000186074 | *CD300LF* | -8.834549643 | 5.00E-05 |
| ENSG00000225783 | *MIAT* | -8.842056623 | 3.39E-05 |
| ENSG00000164303 | *ENPP6* | -8.843022925 | 1.67E-05 |
| ENSG00000104951 | *IL4I1* | -8.847918645 | 6.56E-05 |
| ENSG00000163564 | *PYHIN1* | -8.849064823 | 0.000159769 |
| ENSG00000125675 | *GRIA3* | -8.850183816 | 0.000296104 |
| ENSG00000116031 | *CD207* | -8.854097925 | 7.95E-05 |
| ENSG00000275326 | *NOSTRIN* | -8.861436039 | 0.000909816 |
| ENSG00000175445 | *LPL* | -8.861511055 | 6.29E-06 |
| ENSG00000180525 | *PRR26* | -8.866584869 | 6.71E-05 |
| ENSG00000183346 | *C10orf107* | -8.866597456 | 3.88E-06 |
| ENSG00000078549 | *ADCYAP1R1* | -8.871138794 | 5.54E-05 |
| ENSG00000184905 | *TCEAL2* | -8.871251584 | 4.58E-10 |
| ENSG00000171189 | *GRIK1* | -8.873599803 | 0.000345922 |
| ENSG00000230463 | *HLA-DRB3* | -8.87568835 | 4.06E-05 |
| ENSG00000130528 | *HRC* | -8.877688334 | 0.033686228 |
| ENSG00000161835 | *GRASP* | -8.892962185 | 1.33E-15 |
| ENSG00000106823 | *ECM2* | -8.896025629 | 5.48E-10 |
| ENSG00000116833 | *NR5A2* | -8.897416064 | 7.08E-05 |
| ENSG00000168685 | *IL7R* | -8.911538733 | 4.26E-11 |
| ENSG00000154864 | *PIEZO2* | -8.917579684 | 1.01E-06 |
| ENSG00000108370 | *RGS9* | -8.925323835 | 2.61E-10 |
| ENSG00000198848 | *CES1* | -8.929604128 | 3.39E-05 |
| ENSG00000100427 | *MLC1* | -8.933141576 | 3.87E-05 |
| ENSG00000147113 | *CXorf36* | -8.93907302 | 3.83E-07 |
| ENSG00000075884 | *ARHGAP15* | -8.947982711 | 3.66E-06 |
| ENSG00000276480 | *MYH11* | -8.949207892 | 9.31E-06 |
| ENSG00000105383 | *CD33* | -8.962377947 | 1.73E-05 |
| ENSG00000256508 | *MRGPRF-AS1* | -8.964325084 | 1.69E-05 |
| ENSG00000206549 | *PRSS50* | -8.968397086 | 2.15E-05 |
| ENSG00000206052 | *DOK6* | -8.969730725 | 3.02E-05 |
| ENSG00000198732 | *SMOC1* | -8.974153512 | 1.36E-20 |
| ENSG00000140807 | *NKD1* | -8.9826207 | 5.50E-07 |
| ENSG00000166148 | *AVPR1A* | -8.98312471 | 9.35E-06 |
| ENSG00000162367 | *TAL1* | -8.986183999 | 8.11E-06 |
| ENSG00000117090 | *SLAMF1* | -8.987821435 | 6.35E-05 |
| ENSG00000141744 | *PNMT* | -8.99095359 | 0.00072029 |
| ENSG00000158445 | *KCNB1* | -8.992109243 | 1.49E-06 |
| ENSG00000137809 | *ITGA11* | -8.99470349 | 4.49E-06 |
| ENSG00000189350 | *FAM179A* | -9.007632287 | 1.18E-05 |
| ENSG00000064205 | *WISP2* | -9.010803446 | 1.36E-06 |
| ENSG00000271605 | *MILR1* | -9.012423848 | 1.69E-05 |
| ENSG00000171840 | *NINJ2* | -9.013728625 | 9.13E-06 |
| ENSG00000008118 | *CAMK1G* | -9.01385195 | 1.24E-05 |
| ENSG00000117069 | *ST6GALNAC5* | -9.015508234 | 0.00048834 |
| ENSG00000136869 | *TLR4* | -9.017636224 | 2.32E-06 |
| ENSG00000133392 | *MYH11* | -9.017709435 | 3.89E-06 |
| ENSG00000198075 | *SULT1C4* | -9.018610944 | 9.10E-06 |
| ENSG00000122188 | *LAX1* | -9.018637485 | 0.000120768 |
| ENSG00000122367 | *LDB3* | -9.02070863 | 7.13E-23 |
| ENSG00000168079 | *SCARA5* | -9.030318415 | 8.55E-05 |
| ENSG00000171714 | *ANO5* | -9.036184834 | 9.10E-13 |
| ENSG00000093072 | *CECR1* | -9.039341511 | 1.08E-11 |
| ENSG00000187474 | *FPR3* | -9.042183077 | 4.69E-06 |
| ENSG00000227954 | *TARID* | -9.044142364 | 5.82E-05 |
| ENSG00000174837 | *ADGRE1* | -9.044426889 | 0.000472642 |
| ENSG00000058866 | *DGKG* | -9.053932456 | 1.06E-06 |
| ENSG00000118004 | *COLEC11* | -9.055031564 | 2.29E-05 |
| ENSG00000196932 | *TMEM26* | -9.055093256 | 5.27E-06 |
| ENSG00000169896 | *ITGAM* | -9.058028995 | 9.32E-06 |
| ENSG00000112782 | *CLIC5* | -9.074517893 | 1.18E-05 |
| ENSG00000267532 | *MIR497HG* | -9.075210935 | 2.66E-05 |
| ENSG00000142449 | *FBN3* | -9.076592389 | 7.84E-05 |
| ENSG00000105369 | *CD79A* | -9.080646176 | 0.004318531 |
| ENSG00000184160 | *ADRA2C* | -9.081031475 | 3.75E-05 |
| ENSG00000224367 | *OACYLP* | -9.101802673 | 0.00065524 |
| ENSG00000163563 | *MNDA* | -9.102243931 | 7.23E-06 |
| ENSG00000214313 | *AZGP1P1* | -9.116846134 | 6.37E-05 |
| ENSG00000205277 | *MUC12* | -9.117732201 | 8.30E-06 |
| ENSG00000186510 | *CLCNKA* | -9.12045512 | 0.000407996 |
| ENSG00000044524 | *EPHA3* | -9.120682121 | 5.16E-23 |
| ENSG00000101463 | *SYNDIG1* | -9.134959451 | 4.81E-06 |
| ENSG00000004468 | *CD38* | -9.142648732 | 1.88E-06 |
| ENSG00000147588 | *PMP2* | -9.143694745 | 0.000892528 |
| ENSG00000140678 | *ITGAX* | -9.148363387 | 6.80E-11 |
| ENSG00000198851 | *CD3E* | -9.14841402 | 3.04E-06 |
| ENSG00000138792 | *ENPEP* | -9.15577634 | 2.52E-05 |
| ENSG00000107518 | *ATRNL1* | -9.168504376 | 7.57E-06 |
| ENSG00000174279 | *EVX2* | -9.169401196 | 9.99E-05 |
| ENSG00000198597 | *ZNF536* | -9.169812789 | 0.000143682 |
| ENSG00000128262 | *POM121L9P* | -9.173121677 | 3.10E-05 |
| ENSG00000163884 | *KLF15* | -9.175533222 | 1.98E-09 |
| ENSG00000172005 | *MAL* | -9.179367869 | 6.98E-06 |
| ENSG00000095917 | *TPSD1* | -9.179677818 | 1.18E-05 |
| ENSG00000197471 | *SPN* | -9.181424036 | 9.41E-05 |
| ENSG00000138944 | *KIAA1644* | -9.196507929 | 2.46E-06 |
| ENSG00000152402 | *GUCY1A2* | -9.196547703 | 1.75E-05 |
| ENSG00000156219 | *ART3* | -9.198927727 | 8.72E-05 |
| ENSG00000184343 | *SRPK3* | -9.200754838 | 6.38E-09 |
| ENSG00000169218 | *RSPO1* | -9.206759986 | 4.02E-06 |
| ENSG00000163823 | *CCR1* | -9.212895219 | 1.05E-05 |
| ENSG00000163815 | *CLEC3B* | -9.217165395 | 2.52E-06 |
| ENSG00000143194 | *MAEL* | -9.221473344 | 4.46E-05 |
| ENSG00000240219 | *RP11-430C7.5* | -9.223997648 | 3.39E-05 |
| ENSG00000135426 | *TESPA1* | -9.228829645 | 1.89E-05 |
| ENSG00000169418 | *NPR1* | -9.229538119 | 9.81E-07 |
| ENSG00000163599 | *CTLA4* | -9.229567061 | 2.35E-05 |
| ENSG00000267107 | *PCAT19* | -9.243401864 | 1.46E-05 |
| ENSG00000239911 | *PRKAG2-AS1* | -9.245080085 | 2.31E-06 |
| ENSG00000160808 | *MYL3* | -9.247612752 | 0.037137679 |
| ENSG00000101096 | *NFATC2* | -9.249679469 | 1.34E-06 |
| ENSG00000186642 | *PDE2A* | -9.250476854 | 1.46E-07 |
| ENSG00000071991 | *CDH19* | -9.253714003 | 0.000151226 |
| ENSG00000050555 | *LAMC3* | -9.256135516 | 1.84E-05 |
| ENSG00000160791 | *CCR5* | -9.256952474 | 2.84E-05 |
| ENSG00000164692 | *COL1A2* | -9.263558962 | 4.35E-23 |
| ENSG00000100368 | *CSF2RB* | -9.268268323 | 3.44E-05 |
| ENSG00000149591 | *TAGLN* | -9.274942671 | 9.94E-21 |
| ENSG00000128656 | *CHN1* | -9.277777654 | 3.85E-12 |
| ENSG00000169245 | *CXCL10* | -9.284627183 | 2.36E-05 |
| ENSG00000280429 | *AF001548.3* | -9.288654763 | 1.15E-05 |
| ENSG00000128591 | *FLNC* | -9.289371951 | 8.90E-40 |
| ENSG00000005249 | *PRKAR2B* | -9.29047769 | 8.95E-10 |
| ENSG00000240583 | *AQP1* | -9.296361248 | 1.92E-28 |
| ENSG00000214491 | *SEC14L6* | -9.302177702 | 6.04E-06 |
| ENSG00000136630 | *HLX* | -9.302183805 | 2.01E-06 |
| ENSG00000005102 | *MEOX1* | -9.306520648 | 3.77E-05 |
| ENSG00000211448 | *DIO2* | -9.308350704 | 4.91E-13 |
| ENSG00000106066 | *CPVL* | -9.310437456 | 9.93E-15 |
| ENSG00000211669 | *IGLV3-10* | -9.310500893 | 0.000267652 |
| ENSG00000135744 | *AGT* | -9.312738821 | 1.71E-06 |
| ENSG00000104415 | *WISP1* | -9.325181123 | 3.02E-06 |
| ENSG00000104903 | *LYL1* | -9.326840634 | 3.15E-06 |
| ENSG00000136999 | *NOV* | -9.326979412 | 4.27E-08 |
| ENSG00000117154 | *IGSF21* | -9.330770072 | 2.86E-05 |
| ENSG00000162998 | *FRZB* | -9.350091623 | 3.02E-16 |
| ENSG00000163239 | *TDRD10* | -9.354879955 | 4.32E-06 |
| ENSG00000124772 | *CPNE5* | -9.367948475 | 2.93E-06 |
| ENSG00000134061 | *CD180* | -9.368949653 | 1.69E-05 |
| ENSG00000128052 | *KDR* | -9.373433151 | 8.55E-09 |
| ENSG00000123338 | *NCKAP1L* | -9.37407194 | 3.56E-10 |
| ENSG00000205221 | *VIT* | -9.37450626 | 2.75E-05 |
| ENSG00000169554 | *ZEB2* | -9.376416398 | 1.54E-12 |
| ENSG00000020181 | *ADGRA2* | -9.378073184 | 1.54E-13 |
| ENSG00000250978 | *RP11-357D18.1* | -9.379708871 | 0.000216138 |
| ENSG00000211640 | *IGLV6-57* | -9.385530411 | 0.033184126 |
| ENSG00000183486 | *MX2* | -9.392374486 | 3.73E-06 |
| ENSG00000134326 | *CMPK2* | -9.393033683 | 2.86E-06 |
| ENSG00000162745 | *OLFML2B* | -9.407519585 | 4.15E-07 |
| ENSG00000141293 | *SKAP1* | -9.425328566 | 6.31E-06 |
| ENSG00000150337 | *FCGR1A* | -9.433687359 | 1.59E-05 |
| ENSG00000038945 | *MSR1* | -9.435562244 | 3.20E-07 |
| ENSG00000140538 | *NTRK3* | -9.43775157 | 5.49E-07 |
| ENSG00000282633 | *IGHA1* | -9.443421736 | 0.035964735 |
| ENSG00000165084 | *C8orf34* | -9.44699721 | 0.000216133 |
| ENSG00000172543 | *CTSW* | -9.450386873 | 1.31E-05 |
| ENSG00000154133 | *ROBO4* | -9.45125744 | 1.18E-06 |
| ENSG00000129682 | *FGF13* | -9.454623095 | 3.43E-07 |
| ENSG00000122756 | *CNTFR* | -9.454972744 | 1.03E-05 |
| ENSG00000184709 | *LRRC26* | -9.455816849 | 0.017492482 |
| ENSG00000170390 | *DCLK2* | -9.457005881 | 1.37E-07 |
| ENSG00000110934 | *BIN2* | -9.460185739 | 1.20E-06 |
| ENSG00000144481 | *TRPM8* | -9.465372611 | 4.02E-06 |
| ENSG00000180644 | *PRF1* | -9.466528421 | 2.52E-05 |
| ENSG00000012124 | *CD22* | -9.469545431 | 8.65E-06 |
| ENSG00000166292 | *TMEM100* | -9.474543922 | 3.36E-06 |
| ENSG00000102755 | *FLT1* | -9.477365606 | 2.07E-06 |
| ENSG00000133878 | *DUSP26* | -9.478781574 | 2.43E-05 |
| ENSG00000163380 | *LMOD3* | -9.481889441 | 0.041687262 |
| ENSG00000092009 | *CMA1* | -9.483674465 | 0.000428064 |
| ENSG00000166928 | *MS4A14* | -9.488213059 | 1.62E-05 |
| ENSG00000182253 | *SYNM* | -9.491454474 | 4.86E-30 |
| ENSG00000170891 | *CYTL1* | -9.494088908 | 3.41E-06 |
| ENSG00000280623 | *PCAT14* | -9.500647 | 2.17E-06 |
| ENSG00000111052 | *LIN7A* | -9.511376147 | 1.62E-05 |
| ENSG00000143851 | *PTPN7* | -9.513949477 | 3.93E-06 |
| ENSG00000141750 | *STAC2* | -9.516062317 | 4.99E-06 |
| ENSG00000087116 | *ADAMTS2* | -9.517652696 | 5.63E-08 |
| ENSG00000280143 | *AP000892.6* | -9.518323037 | 3.42E-16 |
| ENSG00000099985 | *OSM* | -9.519699086 | 2.33E-05 |
| ENSG00000096996 | *IL12RB1* | -9.520143179 | 6.12E-06 |
| ENSG00000164764 | *SBSPON* | -9.526948968 | 8.52E-14 |
| ENSG00000177675 | *CD163L1* | -9.526953282 | 7.43E-06 |
| ENSG00000180139 | *ACTA2-AS1* | -9.5396768 | 2.34E-13 |
| ENSG00000156049 | *GNA14* | -9.539762685 | 3.49E-07 |
| ENSG00000087303 | *NID2* | -9.546268413 | 5.68E-07 |
| ENSG00000070193 | *FGF10* | -9.546289151 | 6.35E-05 |
| ENSG00000151812 | *SLC35F4* | -9.550062662 | 8.60E-06 |
| ENSG00000183807 | *FAM162B* | -9.553002516 | 5.88E-06 |
| ENSG00000100450 | *GZMH* | -9.558038416 | 1.22E-05 |
| ENSG00000180875 | *GREM2* | -9.561863353 | 8.12E-07 |
| ENSG00000148053 | *NTRK2* | -9.563818816 | 6.82E-11 |
| ENSG00000050628 | *PTGER3* | -9.564632013 | 2.03E-06 |
| ENSG00000182870 | *GALNT9* | -9.564842152 | 0.000224116 |
| ENSG00000086205 | *FOLH1* | -9.565073421 | 1.53E-05 |
| ENSG00000230006 | *ANKRD36BP2* | -9.572861793 | 0.024838635 |
| ENSG00000079102 | *RUNX1T1* | -9.574126457 | 4.91E-07 |
| ENSG00000219438 | *FAM19A5* | -9.576723862 | 9.39E-07 |
| ENSG00000025423 | *HSD17B6* | -9.579292955 | 5.62E-15 |
| ENSG00000143125 | *PROK1* | -9.585718368 | 5.38E-06 |
| ENSG00000116194 | *ANGPTL1* | -9.588466056 | 6.84E-06 |
| ENSG00000108342 | *CSF3* | -9.621842942 | 6.68E-06 |
| ENSG00000102683 | *SGCG* | -9.631932604 | 9.19E-06 |
| ENSG00000126218 | *F10* | -9.636776595 | 9.46E-08 |
| ENSG00000197291 | *RAMP2-AS1* | -9.638345705 | 2.77E-06 |
| ENSG00000172399 | *MYOZ2* | -9.647837251 | 0.013848461 |
| ENSG00000167037 | *SGSM1* | -9.665653049 | 1.41E-05 |
| ENSG00000131831 | *RAI2* | -9.665692928 | 7.63E-12 |
| ENSG00000173200 | *PARP15* | -9.666953825 | 5.77E-06 |
| ENSG00000137959 | *IFI44L* | -9.672820361 | 5.09E-08 |
| ENSG00000163106 | *HPGDS* | -9.674678173 | 1.79E-05 |
| ENSG00000120156 | *TEK* | -9.678890403 | 2.46E-06 |
| ENSG00000134460 | *IL2RA* | -9.682210049 | 1.84E-06 |
| ENSG00000145362 | *ANK2* | -9.686919553 | 5.45E-11 |
| ENSG00000112214 | *FHL5* | -9.691389562 | 0.000158955 |
| ENSG00000174004 | *NRROS* | -9.69740675 | 2.62E-06 |
| ENSG00000181847 | *TIGIT* | -9.70162748 | 0.017135726 |
| ENSG00000100453 | *GZMB* | -9.705873067 | 0.02589662 |
| ENSG00000185818 | *NAT8L* | -9.717468744 | 1.26E-05 |
| ENSG00000211897 | *IGHG3* | -9.720188555 | 0.002188265 |
| ENSG00000158481 | *CD1C* | -9.72194979 | 8.07E-06 |
| ENSG00000127528 | *KLF2* | -9.745938333 | 1.09E-09 |
| ENSG00000266524 | *GDF10* | -9.746021479 | 6.54E-05 |
| ENSG00000228080 | *HLA-DRB1* | -9.749347116 | 4.37E-06 |
| ENSG00000230630 | *DNM3OS* | -9.758022604 | 6.39E-06 |
| ENSG00000066056 | *TIE1* | -9.760603612 | 7.08E-08 |
| ENSG00000137841 | *PLCB2* | -9.765310066 | 7.98E-10 |
| ENSG00000187848 | *P2RX2* | -9.77161643 | 5.78E-07 |
| ENSG00000149596 | *JPH2* | -9.783185234 | 4.27E-20 |
| ENSG00000168539 | *CHRM1* | -9.796135699 | 1.47E-05 |
| ENSG00000177363 | *LRRN4CL* | -9.801582432 | 6.59E-07 |
| ENSG00000205358 | *MT1H* | -9.803163923 | 0.018317593 |
| ENSG00000109956 | *B3GAT1* | -9.812072861 | 1.46E-05 |
| ENSG00000182162 | *P2RY8* | -9.832888368 | 1.10E-05 |
| ENSG00000235505 | *RP11-693N9.2* | -9.83378688 | 1.22E-06 |
| ENSG00000106018 | *VIPR2* | -9.836559124 | 2.61E-06 |
| ENSG00000276231 | *PIK3R6* | -9.839409525 | 5.14E-06 |
| ENSG00000179639 | *FCER1A* | -9.84152484 | 8.18E-07 |
| ENSG00000101445 | *PPP1R16B* | -9.846296181 | 0.005541847 |
| ENSG00000162551 | *ALPL* | -9.846790595 | 1.13E-07 |
| ENSG00000154553 | *PDLIM3* | -9.84984403 | 2.91E-18 |
| ENSG00000196616 | *ADH1B* | -9.864587132 | 2.10E-05 |
| ENSG00000066294 | *CD84* | -9.874470421 | 5.04E-07 |
| ENSG00000106236 | *NPTX2* | -9.884231009 | 7.96E-05 |
| ENSG00000172995 | *ARPP21* | -9.889890848 | 1.33E-06 |
| ENSG00000166960 | *CCDC178* | -9.892901847 | 1.88E-05 |
| ENSG00000167208 | *SNX20* | -9.893489867 | 2.27E-05 |
| ENSG00000234638 | *AC053503.6* | -9.902506894 | 2.38E-07 |
| ENSG00000188536 | *HBA2* | -9.916050702 | 6.84E-06 |
| ENSG00000109819 | *PPARGC1A* | -9.934253501 | 8.75E-07 |
| ENSG00000153446 | *C16orf89* | -9.946196161 | 1.32E-07 |
| ENSG00000133687 | *TMTC1* | -9.949293699 | 3.98E-08 |
| ENSG00000279526 | *AC011239.2* | -9.960046747 | 3.31E-06 |
| ENSG00000204065 | *TCEAL5* | -9.966591214 | 1.08E-05 |
| ENSG00000149968 | *MMP3* | -9.974237051 | 0.03495058 |
| ENSG00000100473 | *COCH* | -9.976233202 | 1.27E-08 |
| ENSG00000167332 | *OR51E2* | -9.981867666 | 7.16E-07 |
| ENSG00000163638 | *ADAMTS9* | -9.990152063 | 6.88E-07 |
| ENSG00000181195 | *PENK* | -9.990397492 | 2.03E-06 |
| ENSG00000132840 | *BHMT2* | -9.992827997 | 9.33E-09 |
| ENSG00000237949 | *LINC00844* | -10.00179104 | 1.08E-05 |
| ENSG00000169031 | *COL4A3* | -10.00992387 | 9.05E-08 |
| ENSG00000120279 | *MYCT1* | -10.01527446 | 9.39E-07 |
| ENSG00000241351 | *IGKV3-11* | -10.01642755 | 0.020522586 |
| ENSG00000239951 | *IGKV3-20* | -10.01657936 | 0.047620828 |
| ENSG00000146966 | *DENND2A* | -10.01797098 | 1.68E-08 |
| ENSG00000102445 | *KIAA0226L* | -10.0267046 | 6.67E-07 |
| ENSG00000162739 | *SLAMF6* | -10.03147817 | 3.74E-07 |
| ENSG00000154678 | *PDE1C* | -10.03152607 | 6.32E-07 |
| ENSG00000255399 | *TBX5-AS1* | -10.03216911 | 1.25E-05 |
| ENSG00000267505 | *CTC-296K1.3* | -10.03311809 | 7.40E-08 |
| ENSG00000261685 | *RP11-401P9.4* | -10.03368002 | 3.19E-08 |
| ENSG00000172403 | *SYNPO2* | -10.03622067 | 9.76E-32 |
| ENSG00000164879 | *CA3* | -10.04937844 | 0.000855228 |
| ENSG00000277016 | *IGHG4* | -10.05561486 | 0.026985635 |
| ENSG00000182168 | *UNC5C* | -10.05588502 | 1.03E-06 |
| ENSG00000230708 | *HLA-DPB1* | -10.05782133 | 0.000708255 |
| ENSG00000254510 | *RP11-867G23.10* | -10.05807893 | 6.67E-07 |
| ENSG00000147027 | *TMEM47* | -10.06135013 | 2.88E-16 |
| ENSG00000184113 | *CLDN5* | -10.06164584 | 1.39E-11 |
| ENSG00000196126 | *HLA-DRB1* | -10.07176911 | 9.48E-08 |
| ENSG00000166523 | *CLEC4E* | -10.07940383 | 5.32E-07 |
| ENSG00000077420 | *APBB1IP* | -10.09029151 | 2.45E-06 |
| ENSG00000165186 | *PTCHD1* | -10.09059889 | 7.75E-08 |
| ENSG00000113263 | *ITK* | -10.09368346 | 6.54E-07 |
| ENSG00000187068 | *C3orf70* | -10.10383373 | 8.25E-08 |
| ENSG00000188488 | *SERPINA5* | -10.1045054 | 1.97E-05 |
| ENSG00000272789 | *RP11-286H15.1* | -10.10663405 | 8.62E-07 |
| ENSG00000140287 | *HDC* | -10.11112594 | 3.20E-07 |
| ENSG00000124785 | *NRN1* | -10.11513682 | 7.30E-06 |
| ENSG00000172572 | *PDE3A* | -10.12058933 | 4.56E-14 |
| ENSG00000128815 | *WDFY4* | -10.12168998 | 0.011777905 |
| ENSG00000133110 | *POSTN* | -10.13274132 | 6.48E-08 |
| ENSG00000124256 | *ZBP1* | -10.13548101 | 0.016406705 |
| ENSG00000169126 | *ARMC4* | -10.14204779 | 2.30E-07 |
| ENSG00000124205 | *EDN3* | -10.14221309 | 0.02735928 |
| ENSG00000168421 | *RHOH* | -10.14430618 | 5.26E-07 |
| ENSG00000131203 | *IDO1* | -10.15167684 | 1.24E-06 |
| ENSG00000153707 | *PTPRD* | -10.15891491 | 6.07E-07 |
| ENSG00000198844 | *ARHGEF15* | -10.16213948 | 1.16E-08 |
| ENSG00000085741 | *WNT11* | -10.18170962 | 6.71E-08 |
| ENSG00000257542 | *OR7E47P* | -10.18834518 | 1.78E-07 |
| ENSG00000176435 | *CLEC14A* | -10.19011126 | 4.83E-08 |
| ENSG00000131459 | *GFPT2* | -10.19039908 | 1.25E-08 |
| ENSG00000135472 | *FAIM2* | -10.1939476 | 6.57E-08 |
| ENSG00000204136 | *GGTA1P* | -10.19484218 | 9.91E-08 |
| ENSG00000136573 | *BLK* | -10.19627197 | 0.037478946 |
| ENSG00000185052 | *SLC24A3* | -10.22229654 | 2.92E-09 |
| ENSG00000144837 | *PLA1A* | -10.22289131 | 1.78E-08 |
| ENSG00000185862 | *EVI2B* | -10.24342561 | 1.69E-07 |
| ENSG00000187513 | *GJA4* | -10.24473981 | 2.02E-07 |
| ENSG00000280323 | *AC053503.12* | -10.25076496 | 5.98E-08 |
| ENSG00000123243 | *ITIH5* | -10.25222718 | 1.31E-24 |
| ENSG00000123496 | *IL13RA2* | -10.25535416 | 3.32E-07 |
| ENSG00000042980 | *ADAM28* | -10.26175696 | 1.98E-06 |
| ENSG00000149534 | *MS4A2* | -10.26595244 | 7.84E-07 |
| ENSG00000258274 | *RP11-887P2.5* | -10.27332302 | 0.012282302 |
| ENSG00000167286 | *CD3D* | -10.27529793 | 5.56E-07 |
| ENSG00000102032 | *RENBP* | -10.2963235 | 3.98E-08 |
| ENSG00000127241 | *MASP1* | -10.29767383 | 1.05E-20 |
| ENSG00000171051 | *FPR1* | -10.30456359 | 1.03E-06 |
| ENSG00000197859 | *ADAMTSL2* | -10.31022842 | 1.88E-06 |
| ENSG00000186310 | *NAP1L3* | -10.32328587 | 2.22E-07 |
| ENSG00000106772 | *PRUNE2* | -10.32430438 | 6.14E-23 |
| ENSG00000017427 | *IGF1* | -10.33235013 | 1.71E-07 |
| ENSG00000085265 | *FCN1* | -10.33520769 | 0.002547886 |
| ENSG00000145649 | *GZMA* | -10.33959179 | 1.27E-06 |
| ENSG00000133107 | *TRPC4* | -10.34007433 | 1.92E-07 |
| ENSG00000123329 | *ARHGAP9* | -10.34367813 | 0.000608964 |
| ENSG00000109906 | *ZBTB16* | -10.34602579 | 2.20E-06 |
| ENSG00000179144 | *GIMAP7* | -10.34621376 | 3.58E-08 |
| ENSG00000077984 | *CST7* | -10.34755053 | 5.72E-07 |
| ENSG00000112799 | *LY86* | -10.35051194 | 3.16E-07 |
| ENSG00000260802 | *LINC00890* | -10.35206541 | 3.06E-07 |
| ENSG00000007312 | *CD79B* | -10.35432886 | 9.51E-07 |
| ENSG00000164330 | *EBF1* | -10.35680781 | 1.95E-06 |
| ENSG00000141506 | *PIK3R5* | -10.35962943 | 1.56E-07 |
| ENSG00000160801 | *PTH1R* | -10.36134137 | 2.76E-08 |
| ENSG00000173762 | *CD7* | -10.36289495 | 2.22E-06 |
| ENSG00000152049 | *KCNE4* | -10.36577781 | 3.12E-08 |
| ENSG00000028137 | *TNFRSF1B* | -10.366333 | 2.30E-11 |
| ENSG00000154783 | *FGD5* | -10.37104282 | 1.98E-08 |
| ENSG00000120907 | *ADRA1A* | -10.38661215 | 3.71E-08 |
| ENSG00000174175 | *SELP* | -10.38784332 | 3.98E-08 |
| ENSG00000117091 | *CD48* | -10.39292232 | 4.26E-07 |
| ENSG00000214402 | *LCNL1* | -10.39368945 | 1.32E-06 |
| ENSG00000158859 | *ADAMTS4* | -10.40110421 | 2.89E-15 |
| ENSG00000090539 | *CHRD* | -10.40886767 | 9.05E-09 |
| ENSG00000165973 | *NELL1* | -10.40937446 | 9.31E-08 |
| ENSG00000167654 | *ATCAY* | -10.41443922 | 1.96E-08 |
| ENSG00000138755 | *CXCL9* | -10.42025116 | 1.50E-06 |
| ENSG00000099998 | *GGT5* | -10.42329537 | 1.29E-09 |
| ENSG00000118729 | *CASQ2* | -10.43011908 | 7.85E-09 |
| ENSG00000005844 | *ITGAL* | -10.43060168 | 2.75E-07 |
| ENSG00000160883 | *HK3* | -10.44394855 | 1.62E-06 |
| ENSG00000169347 | *GP2* | -10.45163869 | 0.033233489 |
| ENSG00000110848 | *CD69* | -10.45782835 | 2.98E-08 |
| ENSG00000108798 | *ABI3* | -10.46025567 | 1.40E-08 |
| ENSG00000007062 | *PROM1* | -10.46863968 | 2.33E-07 |
| ENSG00000139567 | *ACVRL1* | -10.47588757 | 3.33E-09 |
| ENSG00000133561 | *GIMAP6* | -10.47890059 | 1.10E-08 |
| ENSG00000241684 | *ADAMTS9-AS2* | -10.49865005 | 1.24E-07 |
| ENSG00000180209 | *MYLPF* | -10.50115261 | 0.001032939 |
| ENSG00000089225 | *TBX5* | -10.50536251 | 7.44E-07 |
| ENSG00000265107 | *GJA5* | -10.50731242 | 5.82E-07 |
| ENSG00000229295 | *HLA-DPB1* | -10.51366158 | 1.78E-08 |
| ENSG00000124126 | *PREX1* | -10.52378629 | 2.89E-08 |
| ENSG00000173406 | *DAB1* | -10.52409858 | 2.52E-07 |
| ENSG00000188176 | *SMTNL2* | -10.52464689 | 0.002969728 |
| ENSG00000187688 | *TRPV2* | -10.52901204 | 5.86E-08 |
| ENSG00000124479 | *NDP* | -10.54264598 | 2.67E-06 |
| ENSG00000144278 | *GALNT13* | -10.54634326 | 6.45E-07 |
| ENSG00000048540 | *LMO3* | -10.55254908 | 9.93E-10 |
| ENSG00000198626 | *RYR2* | -10.55464665 | 1.04E-07 |
| ENSG00000155307 | *SAMSN1* | -10.5560381 | 3.36E-07 |
| ENSG00000125384 | *PTGER2* | -10.56546241 | 7.87E-09 |
| ENSG00000127083 | *OMD* | -10.56606142 | 7.22E-07 |
| ENSG00000160862 | *AZGP1* | -10.58817651 | 5.70E-17 |
| ENSG00000105374 | *NKG7* | -10.59800844 | 0.001445229 |
| ENSG00000136160 | *EDNRB* | -10.59835842 | 6.56E-11 |
| ENSG00000034971 | *MYOC* | -10.60227454 | 0.00718355 |
| ENSG00000204161 | *C10orf128* | -10.60288525 | 6.85E-08 |
| ENSG00000242534 | *IGKV2D-28* | -10.61025968 | 0.008473718 |
| ENSG00000164188 | *RANBP3L* | -10.61668061 | 1.27E-06 |
| ENSG00000167749 | *KLK4* | -10.61902864 | 3.43E-08 |
| ENSG00000150625 | *GPM6A* | -10.61989117 | 4.56E-08 |
| ENSG00000185811 | *IKZF1* | -10.62581314 | 3.10E-08 |
| ENSG00000105122 | *RASAL3* | -10.62589719 | 1.07E-07 |
| ENSG00000127951 | *FGL2* | -10.62730184 | 1.55E-09 |
| ENSG00000144681 | *STAC* | -10.63013444 | 7.25E-08 |
| ENSG00000179776 | *CDH5* | -10.63641914 | 1.91E-09 |
| ENSG00000203747 | *FCGR3A* | -10.64088545 | 2.36E-08 |
| ENSG00000172987 | *HPSE2* | -10.64612437 | 2.10E-07 |
| ENSG00000177455 | *CD19* | -10.64697331 | 0.004200785 |
| ENSG00000269404 | *SPIB* | -10.6536192 | 0.007722048 |
| ENSG00000118526 | *TCF21* | -10.65458914 | 1.45E-08 |
| ENSG00000151892 | *GFRA1* | -10.67468437 | 7.36E-08 |
| ENSG00000144218 | *AFF3* | -10.67654231 | 8.22E-09 |
| ENSG00000019169 | *MARCO* | -10.68406222 | 5.90E-07 |
| ENSG00000100365 | *NCF4* | -10.68902596 | 2.65E-07 |
| ENSG00000162989 | *KCNJ3* | -10.69739874 | 3.19E-06 |
| ENSG00000142512 | *SIGLEC10* | -10.70034117 | 9.50E-09 |
| ENSG00000178538 | *CA8* | -10.70062131 | 1.18E-07 |
| ENSG00000142173 | *COL6A2* | -10.70068747 | 4.89E-31 |
| ENSG00000133574 | *GIMAP4* | -10.70377151 | 5.85E-09 |
| ENSG00000166482 | *MFAP4* | -10.70565045 | 1.75E-26 |
| ENSG00000206240 | *HLA-DRB1* | -10.71114935 | 1.14E-08 |
| ENSG00000088827 | *SIGLEC1* | -10.71211619 | 2.10E-08 |
| ENSG00000163618 | *CADPS* | -10.71889921 | 1.81E-07 |
| ENSG00000046889 | *PREX2* | -10.75078695 | 1.14E-08 |
| ENSG00000161405 | *IKZF3* | -10.75327945 | 1.27E-07 |
| ENSG00000151067 | *CACNA1C* | -10.76796832 | 9.50E-10 |
| ENSG00000139329 | *LUM* | -10.77447747 | 1.02E-17 |
| ENSG00000136960 | *ENPP2* | -10.77778908 | 1.98E-09 |
| ENSG00000116824 | *CD2* | -10.77957375 | 2.54E-08 |
| ENSG00000135333 | *EPHA7* | -10.78190594 | 1.96E-08 |
| ENSG00000082175 | *PGR* | -10.78299019 | 1.29E-09 |
| ENSG00000009694 | *TENM1* | -10.78349815 | 8.01E-09 |
| ENSG00000108924 | *HLF* | -10.79302016 | 1.05E-09 |
| ENSG00000122679 | *RAMP3* | -10.79385147 | 1.19E-09 |
| ENSG00000162618 | *ADGRL4* | -10.79598434 | 1.40E-08 |
| ENSG00000078295 | *ADCY2* | -10.80332852 | 3.93E-08 |
| ENSG00000005108 | *THSD7A* | -10.8142776 | 3.56E-08 |
| ENSG00000165168 | *CYBB* | -10.81470359 | 1.48E-08 |
| ENSG00000145703 | *IQGAP2* | -10.82170002 | 1.92E-09 |
| ENSG00000167157 | *PRRX2* | -10.82522933 | 4.58E-09 |
| ENSG00000092054 | *MYH7* | -10.83648679 | 0.019357357 |
| ENSG00000125810 | *CD93* | -10.84298097 | 1.98E-09 |
| ENSG00000054938 | *CHRDL2* | -10.85372569 | 1.27E-08 |
| ENSG00000211893 | *IGHG2* | -10.85916327 | 0.02639796 |
| ENSG00000188282 | *RUFY4* | -10.86387546 | 1.37E-06 |
| ENSG00000165457 | *FOLR2* | -10.86538088 | 2.29E-08 |
| ENSG00000174600 | *CMKLR1* | -10.87969575 | 1.65E-08 |
| ENSG00000267405 | *CTC-296K1.4* | -10.89403915 | 5.23E-08 |
| ENSG00000155629 | *PIK3AP1* | -10.90310025 | 4.09E-08 |
| ENSG00000170624 | *SGCD* | -10.90766869 | 8.55E-09 |
| ENSG00000155849 | *ELMO1* | -10.90844279 | 2.58E-09 |
| ENSG00000154451 | *GBP5* | -10.91400981 | 2.56E-07 |
| ENSG00000157445 | *CACNA2D3* | -10.91517836 | 6.04E-09 |
| ENSG00000136244 | *IL6* | -10.91949767 | 9.39E-07 |
| ENSG00000100448 | *CTSG* | -10.92647625 | 4.45E-07 |
| ENSG00000225986 | *UBXN10-AS1* | -10.94080799 | 1.36E-07 |
| ENSG00000111452 | *ADGRD1* | -10.95225549 | 1.91E-09 |
| ENSG00000122122 | *SASH3* | -10.95676055 | 0.00091334 |
| ENSG00000164736 | *SOX17* | -10.96051526 | 5.63E-08 |
| ENSG00000163359 | *COL6A3* | -10.9728125 | 6.56E-33 |
| ENSG00000143297 | *FCRL5* | -10.99467956 | 0.025971135 |
| ENSG00000115165 | *CYTIP* | -10.99503472 | 2.04E-08 |
| ENSG00000225217 | *HSPA7* | -11.02110514 | 1.70E-08 |
| ENSG00000162630 | *B3GALT2* | -11.02411341 | 3.15E-08 |
| ENSG00000169508 | *GPR183* | -11.02809313 | 5.85E-09 |
| ENSG00000156738 | *MS4A1* | -11.03917708 | 0.007895583 |
| ENSG00000169744 | *LDB2* | -11.04615328 | 3.44E-09 |
| ENSG00000112936 | *C7* | -11.04929828 | 2.24E-13 |
| ENSG00000177575 | *CD163* | -11.05172888 | 1.10E-10 |
| ENSG00000101470 | *TNNC2* | -11.05931986 | 0.000238466 |
| ENSG00000147443 | *DOK2* | -11.06307489 | 3.22E-08 |
| ENSG00000143119 | *CD53* | -11.0702426 | 3.04E-09 |
| ENSG00000121075 | *TBX4* | -11.07077206 | 0.0013913 |
| ENSG00000130592 | *LSP1* | -11.08741587 | 3.60E-09 |
| ENSG00000010327 | *STAB1* | -11.09093479 | 3.28E-23 |
| ENSG00000101265 | *RASSF2* | -11.0936608 | 6.91E-08 |
| ENSG00000146352 | *CLVS2* | -11.09438476 | 3.50E-08 |
| ENSG00000185274 | *WBSCR17* | -11.10277156 | 1.64E-09 |
| ENSG00000137077 | *CCL21* | -11.10500581 | 0.000937144 |
| ENSG00000091482 | *SMPX* | -11.11031025 | 0.035190292 |
| ENSG00000164530 | *PI16* | -11.11312407 | 1.46E-12 |
| ENSG00000136250 | *AOAH* | -11.16900827 | 1.24E-08 |
| ENSG00000136286 | *MYO1G* | -11.17537342 | 8.08E-08 |
| ENSG00000081189 | *MEF2C* | -11.17981894 | 3.86E-09 |
| ENSG00000105989 | *WNT2* | -11.18553772 | 4.19E-08 |
| ENSG00000171860 | *C3AR1* | -11.19148203 | 2.01E-09 |
| ENSG00000169413 | *RNASE6* | -11.1917932 | 1.85E-09 |
| ENSG00000182578 | *CSF1R* | -11.20143189 | 4.69E-10 |
| ENSG00000174059 | *CD34* | -11.21526523 | 1.58E-10 |
| ENSG00000173376 | *NDNF* | -11.21591261 | 6.49E-10 |
| ENSG00000277893 | *SRD5A2* | -11.23242087 | 6.41E-09 |
| ENSG00000140968 | *IRF8* | -11.25517033 | 0.000297154 |
| ENSG00000198336 | *MYL4* | -11.26858632 | 0.028955299 |
| ENSG00000118785 | *SPP1* | -11.27845471 | 9.49E-09 |
| ENSG00000123560 | *PLP1* | -11.29701869 | 5.40E-08 |
| ENSG00000106624 | *AEBP1* | -11.31769951 | 9.36E-37 |
| ENSG00000106483 | *SFRP4* | -11.32749395 | 6.09E-09 |
| ENSG00000134516 | *DOCK2* | -11.33098991 | 7.28E-09 |
| ENSG00000177519 | *RPRM* | -11.3318407 | 2.57E-09 |
| ENSG00000183801 | *OLFML1* | -11.35009288 | 3.32E-10 |
| ENSG00000162511 | *LAPTM5* | -11.35013233 | 4.24E-20 |
| ENSG00000215218 | *UBE2QL1* | -11.35034434 | 4.35E-10 |
| ENSG00000172935 | *MRGPRF* | -11.38252579 | 1.31E-10 |
| ENSG00000130700 | *GATA5* | -11.39044821 | 0.003634352 |
| ENSG00000132514 | *CLEC10A* | -11.41132328 | 1.74E-09 |
| ENSG00000130300 | *PLVAP* | -11.41688199 | 2.64E-09 |
| ENSG00000160307 | *S100B* | -11.47917832 | 5.85E-09 |
| ENSG00000136099 | *PCDH8* | -11.48184029 | 6.45E-07 |
| ENSG00000019991 | *HGF* | -11.5194991 | 1.20E-09 |
| ENSG00000145423 | *SFRP2* | -11.52798655 | 3.91E-11 |
| ENSG00000110079 | *MS4A4A* | -11.52923338 | 6.61E-10 |
| ENSG00000147168 | *IL2RG* | -11.53128795 | 0.000256786 |
| ENSG00000243466 | *IGKV1-5* | -11.53881163 | 0.026346432 |
| ENSG00000244734 | *HBB* | -11.55897385 | 0.002688989 |
| ENSG00000183160 | *TMEM119* | -11.56262168 | 6.38E-09 |
| ENSG00000131477 | *RAMP2* | -11.56427903 | 1.09E-09 |
| ENSG00000253755 | *IGHGP* | -11.56484821 | 0.009281792 |
| ENSG00000112175 | *BMP5* | -11.56825418 | 0.001082131 |
| ENSG00000115085 | *ZAP70* | -11.57564113 | 1.15E-08 |
| ENSG00000231286 | *HLA-DQB1* | -11.58899367 | 7.08E-09 |
| ENSG00000107562 | *CXCL12* | -11.59508965 | 1.87E-10 |
| ENSG00000146374 | *RSPO3* | -11.59800819 | 0.000689894 |
| ENSG00000166831 | *RBPMS2* | -11.62502037 | 2.79E-11 |
| ENSG00000170153 | *RNF150* | -11.63300829 | 5.17E-11 |
| ENSG00000115956 | *PLEK* | -11.65200593 | 0.000158027 |
| ENSG00000185565 | *LSAMP* | -11.65329145 | 4.30E-11 |
| ENSG00000142515 | *KLK3* | -11.67171955 | 6.98E-20 |
| ENSG00000163145 | *C1QTNF7* | -11.68002247 | 1.83E-10 |
| ENSG00000211648 | *IGLV1-47* | -11.70715052 | 0.017322454 |
| ENSG00000187955 | *COL14A1* | -11.71178121 | 4.67E-11 |
| ENSG00000138964 | *PARVG* | -11.72165966 | 8.66E-10 |
| ENSG00000135447 | *PPP1R1A* | -11.72170099 | 2.64E-11 |
| ENSG00000184613 | *NELL2* | -11.72178466 | 2.07E-08 |
| ENSG00000198125 | *MB* | -11.73158859 | 0.001463556 |
| ENSG00000136546 | *SCN7A* | -11.73265937 | 1.16E-08 |
| ENSG00000164106 | *SCRG1* | -11.73772259 | 1.21E-08 |
| ENSG00000119508 | *NR4A3* | -11.76200056 | 4.85E-11 |
| ENSG00000140285 | *FGF7* | -11.7677086 | 8.38E-10 |
| ENSG00000141052 | *MYOCD* | -11.81558769 | 9.01E-11 |
| ENSG00000260314 | *MRC1* | -11.83986167 | 5.28E-10 |
| ENSG00000089472 | *HEPH* | -11.86135313 | 1.28E-11 |
| ENSG00000171303 | *KCNK3* | -11.8646912 | 9.70E-10 |
| ENSG00000241158 | *ADAMTS9-AS1* | -11.86978932 | 4.30E-10 |
| ENSG00000043462 | *LCP2* | -11.8711988 | 1.98E-10 |
| ENSG00000066336 | *SPI1* | -11.88715944 | 1.55E-09 |
| ENSG00000002933 | *TMEM176A* | -11.93206447 | 7.70E-11 |
| ENSG00000170962 | *PDGFD* | -11.93272312 | 1.18E-09 |
| ENSG00000158869 | *FCER1G* | -11.96848684 | 3.31E-10 |
| ENSG00000213088 | *ACKR1* | -11.98364771 | 5.46E-11 |
| ENSG00000077522 | *ACTN2* | -11.98915448 | 0.010844112 |
| ENSG00000144339 | *TMEFF2* | -12.00269331 | 1.61E-11 |
| ENSG00000166927 | *MS4A7* | -12.01337195 | 2.97E-11 |
| ENSG00000166432 | *ZMAT1* | -12.02895968 | 2.77E-10 |
| ENSG00000172724 | *CCL19* | -12.03721322 | 0.00038347 |
| ENSG00000133800 | *LYVE1* | -12.06194923 | 6.80E-09 |
| ENSG00000137491 | *SLCO2B1* | -12.06430658 | 2.98E-12 |
| ENSG00000155659 | *VSIG4* | -12.08488123 | 1.76E-11 |
| ENSG00000049540 | *ELN* | -12.09107053 | 6.63E-06 |
| ENSG00000167751 | *KLK2* | -12.09435888 | 4.20E-18 |
| ENSG00000143226 | *FCGR2A* | -12.11346828 | 1.72E-11 |
| ENSG00000136842 | *TMOD1* | -12.11633228 | 1.19E-11 |
| ENSG00000130598 | *TNNI2* | -12.13084254 | 0.000415288 |
| ENSG00000197629 | *MPEG1* | -12.14177941 | 8.77E-10 |
| ENSG00000147655 | *RSPO2* | -12.14654133 | 0.000703449 |
| ENSG00000130755 | *GMFG* | -12.15793095 | 6.06E-10 |
| ENSG00000155926 | *SLA* | -12.16239449 | 7.63E-12 |
| ENSG00000072163 | *LIMS2* | -12.1764576 | 1.80E-21 |
| ENSG00000174403 | *C20orf166-AS1* | -12.18598179 | 6.94E-12 |
| ENSG00000119865 | *CNRIP1* | -12.18619956 | 4.88E-11 |
| ENSG00000239474 | *KLHL41* | -12.21476036 | 0.023263515 |
| ENSG00000081237 | *PTPRC* | -12.21749394 | 1.04E-09 |
| ENSG00000165966 | *PDZRN4* | -12.22785733 | 1.02E-10 |
| ENSG00000160593 | *AMICA1* | -12.31391424 | 2.56E-11 |
| ENSG00000164122 | *ASB5* | -12.33622459 | 0.0001568 |
| ENSG00000240045 | *RP11-451G4.2* | -12.35153507 | 0.043501075 |
| ENSG00000165633 | *VSTM4* | -12.35533756 | 6.95E-13 |
| ENSG00000163751 | *CPA3* | -12.36055154 | 1.50E-10 |
| ENSG00000249307 | *LINC01088* | -12.37082738 | 1.78E-08 |
| ENSG00000124440 | *HIF3A* | -12.37532764 | 2.98E-12 |
| ENSG00000119147 | *C2orf40* | -12.37665271 | 7.63E-12 |
| ENSG00000282094 | *IGHGP* | -12.39534988 | 0.002191473 |
| ENSG00000211895 | *IGHA1* | -12.40717788 | 0.000169823 |
| ENSG00000113721 | *PDGFRB* | -12.41688611 | 1.79E-12 |
| ENSG00000172236 | *TPSAB1* | -12.42653597 | 1.47E-11 |
| ENSG00000112562 | *SMOC2* | -12.43727567 | 1.02E-13 |
| ENSG00000127472 | *PLA2G5* | -12.44448064 | 4.85E-11 |
| ENSG00000106809 | *OGN* | -12.461527 | 6.38E-12 |
| ENSG00000227993 | *HLA-DRA* | -12.48540909 | 1.94E-12 |
| ENSG00000231679 | *HLA-DRB3* | -12.49086082 | 1.95E-09 |
| ENSG00000154188 | *ANGPT1* | -12.52374923 | 5.43E-11 |
| ENSG00000124491 | *F13A1* | -12.57574946 | 2.16E-11 |
| ENSG00000104879 | *CKM* | -12.60208696 | 7.42E-05 |
| ENSG00000228987 | *HLA-DRA* | -12.61553908 | 0.018785153 |
| ENSG00000205364 | *MT1M* | -12.62463637 | 2.74E-12 |
| ENSG00000134201 | *GSTM5* | -12.65196251 | 3.61E-12 |
| ENSG00000149294 | *NCAM1* | -12.66644997 | 1.24E-12 |
| ENSG00000133055 | *MYBPH* | -12.69335696 | 0.038831993 |
| ENSG00000106565 | *TMEM176B* | -12.70414201 | 4.10E-12 |
| ENSG00000154096 | *THY1* | -12.71997337 | 4.30E-11 |
| ENSG00000163431 | *LMOD1* | -12.72637791 | 3.74E-32 |
| ENSG00000211598 | *IGKV4-1* | -12.74427194 | 0.008183619 |
| ENSG00000174348 | *PODN* | -12.74530042 | 8.75E-14 |
| ENSG00000204291 | *COL15A1* | -12.75382725 | 3.32E-13 |
| ENSG00000170419 | *VSTM2A* | -12.82653451 | 0.036235051 |
| ENSG00000104313 | *EYA1* | -12.83189795 | 1.26E-11 |
| ENSG00000156234 | *CXCL13* | -12.8376459 | 0.000814598 |
| ENSG00000154330 | *PGM5* | -12.90192639 | 1.35E-29 |
| ENSG00000211679 | *IGLC3* | -12.92099596 | 1.59E-05 |
| ENSG00000249669 | *MIR143HG* | -12.93341717 | 2.68E-13 |
| ENSG00000138650 | *PCDH10* | -12.95546332 | 3.57E-13 |
| ENSG00000277633 | *IGHG1* | -12.97357138 | 0.001421519 |
| ENSG00000161281 | *COX7A1* | -12.98697464 | 7.95E-12 |
| ENSG00000150051 | *MKX* | -12.99503335 | 7.79E-11 |
| ENSG00000188783 | *PRELP* | -12.99619711 | 1.76E-14 |
| ENSG00000188257 | *PLA2G2A* | -13.00682466 | 8.27E-12 |
| ENSG00000108823 | *SGCA* | -13.01148425 | 6.56E-13 |
| ENSG00000072952 | *MRVI1* | -13.01955458 | 9.07E-13 |
| ENSG00000124212 | *PTGIS* | -13.03098609 | 1.57E-13 |
| ENSG00000261371 | *PECAM1* | -13.05981161 | 8.65E-06 |
| ENSG00000110077 | *MS4A6A* | -13.14058061 | 9.12E-14 |
| ENSG00000196091 | *MYBPC1* | -13.15660444 | 7.52E-12 |
| ENSG00000136732 | *GYPC* | -13.22061841 | 1.18E-12 |
| ENSG00000103710 | *RASL12* | -13.24293182 | 1.51E-14 |
| ENSG00000132465 | *JCHAIN* | -13.24954027 | 6.18E-06 |
| ENSG00000274497 | *IGHG2* | -13.25366443 | 0.002735888 |
| ENSG00000106034 | *CPED1* | -13.26602665 | 2.24E-13 |
| ENSG00000011600 | *TYROBP* | -13.2758281 | 3.66E-13 |
| ENSG00000197253 | *TPSB2* | -13.36491282 | 9.47E-12 |
| ENSG00000173641 | *HSPB7* | -13.48584891 | 4.21E-12 |
| ENSG00000121966 | *CXCR4* | -13.51079161 | 6.30E-14 |
| ENSG00000196557 | *CACNA1H* | -13.52145185 | 2.83E-06 |
| ENSG00000211666 | *IGLV2-14* | -13.52619137 | 0.00953132 |
| ENSG00000103241 | *FOXF1* | -13.5441731 | 9.04E-15 |
| ENSG00000130595 | *TNNT3* | -13.54939672 | 1.19E-05 |
| ENSG00000163017 | *ACTG2* | -13.63722463 | 1.26E-45 |
| ENSG00000108691 | *CCL2* | -13.67782354 | 2.55E-12 |
| ENSG00000168542 | *COL3A1* | -13.68153007 | 1.81E-25 |
| ENSG00000116132 | *PRRX1* | -13.69574708 | 1.13E-14 |
| ENSG00000129009 | *ISLR* | -13.72268987 | 6.65E-14 |
| ENSG00000241644 | *INMT* | -13.77838649 | 9.15E-14 |
| ENSG00000143196 | *DPT* | -13.81301236 | 5.77E-13 |
| ENSG00000268388 | *FENDRR* | -13.82750511 | 1.85E-16 |
| ENSG00000224958 | *PGM5-AS1* | -13.83952597 | 2.93E-06 |
| ENSG00000129538 | *RNASE1* | -13.9746905 | 1.59E-13 |
| ENSG00000143632 | *ACTA1* | -14.00870546 | 0.001117256 |
| ENSG00000159251 | *ACTC1* | -14.02110495 | 0.000759363 |
| ENSG00000159189 | *C1QC* | -14.03955856 | 2.16E-15 |
| ENSG00000090104 | *RGS1* | -14.04144087 | 3.23E-16 |
| ENSG00000198523 | *PLN* | -14.23043526 | 8.76E-06 |
| ENSG00000105894 | *PTN* | -14.4100207 | 1.23E-14 |
| ENSG00000168309 | *FAM107A* | -14.43421487 | 1.42E-15 |
| ENSG00000130176 | *CNN1* | -14.45106276 | 8.17E-32 |
| ENSG00000107317 | *PTGDS* | -14.51569936 | 2.89E-15 |
| ENSG00000101951 | *PAGE4* | -14.53748249 | 0.000120816 |
| ENSG00000145936 | *KCNMB1* | -14.6512689 | 6.32E-17 |
| ENSG00000211896 | *IGHG1* | -14.65178552 | 4.43E-05 |
| ENSG00000173369 | *C1QB* | -14.68030326 | 6.35E-17 |
| ENSG00000276173 | *IGHA2* | -14.71627446 | 0.000614311 |
| ENSG00000175899 | *A2M* | -14.90868141 | 7.43E-08 |
| ENSG00000011465 | *DCN* | -14.94472152 | 3.91E-15 |
| ENSG00000149451 | *ADAM33* | -14.96972763 | 2.28E-18 |
| ENSG00000111341 | *MGP* | -15.01169551 | 6.09E-19 |
| ENSG00000269936 | *RP11-394O4.5* | -15.01804022 | 2.09E-17 |
| ENSG00000173372 | *C1QA* | -15.10144604 | 1.05E-16 |
| ENSG00000152583 | *SPARCL1* | -15.12242334 | 5.83E-26 |
| ENSG00000103175 | *WFDC1* | -15.12798569 | 1.92E-17 |
| ENSG00000175084 | *DES* | -15.21096673 | 8.61E-65 |
| ENSG00000077943 | *ITGA8* | -15.21640326 | 6.35E-17 |
| ENSG00000101938 | *CHRDL1* | -15.40599456 | 1.85E-17 |
| ENSG00000211592 | *IGKC* | -15.78528352 | 2.78E-06 |
| ENSG00000211677 | *IGLC2* | -15.99467995 | 7.31E-06 |
| ENSG00000183036 | *PCP4* | -16.50940177 | 5.62E-18 |
| ENSG00000012223 | *LTF* | -16.61149589 | 9.65E-07 |
| ENSG00000100721 | *TCL1A* | -20.52019168 | 0.000236611 |
| ENSG00000282399 | *IGHV1-69-2* | -21.51014555 | 0.0001001 |
| ENSG00000132704 | *FCRL2* | -21.8263062 | 7.57E-05 |
| ENSG00000211642 | *IGLV10-54* | -22.10085912 | 5.94E-05 |
| ENSG00000197893 | *NRAP* | -22.4452823 | 4.36E-05 |
| ENSG00000163092 | *XIRP2* | -22.95035813 | 2.72E-05 |
| ENSG00000186526 | *CYP4F8* | -22.98717546 | 2.63E-05 |
| ENSG00000156885 | *COX6A2* | -23.15019667 | 2.27E-05 |
| ENSG00000111046 | *MYF6* | -23.38352636 | 1.82E-05 |
| ENSG00000138100 | *TRIM54* | -23.40871107 | 1.78E-05 |
| ENSG00000233209 | *HLA-DQB1* | -23.89972954 | 1.13E-05 |
| ENSG00000117215 | *PLA2G2D* | -23.9295592 | 1.10E-05 |
| ENSG00000170807 | *LMOD2* | -24.22662962 | 8.27E-06 |
| ENSG00000211955 | *IGHV3-33* | -24.41577434 | 6.84E-06 |
| ENSG00000211660 | *IGLV2-23* | -24.93522999 | 4.07E-06 |
| ENSG00000122180 | *MYOG* | -24.95465593 | 4.00E-06 |
| ENSG00000170290 | *SLN* | -25.39978909 | 2.54E-06 |
| ENSG00000186439 | *TRDN* | -26.22592093 | 1.08E-06 |
| ENSG00000111245 | *MYL2* | -27.03052387 | 4.65E-07 |
| ENSG00000168530 | *MYL1* | -28.09309578 | 1.39E-07 |
